# Supplementary material for: Use of the Hospital Survey of Patient Safety Culture in Norwegian Hospitals: A Systematic Review
Source: Int J Environ Res Public Health. 2021 Jun 17;18(12):6518. doi: 10.3390/ijerph18126518 (PMC8296424; doi:10.3390/ijerph18126518)
Supplement: Supplementary file 1 [file ijerph-18-06518-s001.zip › ijerph-1231785-supplementary.pdf]

International Journal of
Environmental Research and Public Health

MDPI

Table S1. All sources.

ollection Name
123Library
1findr
AAPG/Datapages
AASLH Publications
Free Full-Text Journals in Chemistry
abc.es
ABC-CLIO eBooks
African American Experience (Academic)
American Government (Academic)
American History (Academic)
American Indian Experience (Academic)
Daily Life through History(Academic)
History Reference Online
Issues(Academic)
Issues: Understanding Controversy and Society
Latino American Experience(Academic)
Modern Genocide
Pop Culture Universe
Popular Culture Universe(Academic)
World At War Understanding Conflict and Society(Academic)
World Geography (Academic)
World History: Ancient and Medieval Eras
World History: The Modern Era (Academic)
World Religions
Calames
Theses.fr
Thèses.fr
Statistica Sinica
Academic Journals Free
Academy of Management
Accessible Archives Complete
African American Newspapers: Part I
African American Newspapers: Part II
African American Newspapers: Part III
African American Newspapers: Part IV
African American Newspapers: Part IX

African American Newspapers: Part V
African American Newspapers: Part VI
African American Newspapers: Part VII
African American Newspapers: Part VIII
African American Newspapers: Part X
African American Newspapers: Part XI
African American Newspapers: Part XII
African-American Newspapers: The 19th Century
Civil War Collection
County Histories
Frank Leslie's Illustrated Newspaper
Godey's Lady's Book 1830-1898
National Anti-Slavery Standard
National Citizen and Ballot Box
Scenes in the life of Harriet Tubman
South Carolina Newspapers 1732-1780
The Civil War: Part I: A Newspaper Perspective
The Liberator 1831-1865
The Lily
The Pennsylvania Gazette 1728-1800
The Pennsylvania Genealogical Catalogue Chester County 1809-1870
The Pennsylvania Newspaper Record Delaware County 1819-1870
The Revolution
Virginia Gazette, Part 2: 1746-1755
Virginia Gazette, Part I: 1736-1745
Virginia Gazette, Part III: 1756-1765
Virginia Gazette, Part IV: 1766-1775
Virginia Gazette, Part V: 1776-1780
ACLS Humanities eBook
ACM Digital Library Complete
KB+ BIBSAM Association For Computing Machinery Digital Library 2015-2018
KB+ Bibsam Association for Computing Machinery Digital Library Journals 2020
KB+ JISC Collections Association For Computing Machinery Digital Library 2017-2019
ACSESS Digital Library
American Society of Agronomy
Ad Parnassum
African American Communities
Age of Exploration
American Consumer Culture, 1935-1965
American History Module I: Settlement, Commerce, Revolution and Reform: 1493-1859

American History Module II: Civil War, Reconstruction and the Modern Era: 1860-1945
American Indian Histories and Culture
American West
Apartheid South Africa, 1948-1980
Archives Direct
Area Studies: China and Southeast Asia
Area Studies: India
Area Studies: Japan
China, America and the Pacific
China: Culture and Society
China: The Wason Pamphlet Collection
China: Trade, Politics and Culture, 1793-1980
Church Missionary Society Periodicals Module 1: Global Missions and Contemporary Encounters, 1804-2009
Church Missionary Society Periodicals Module 2: Medical Journals, Asian Missions and The Historical Record, 1816-1986
Church Missionary Society Periodicals: Module I Global Missions and Contemporary Encounters, 1804-2006
Colonial America
Colonial America Module 1: Early Settlement, Expansion and Rivalries
Colonial America Module 2: Towards Revolution
Colonial America Module 3: The American Revolution
Colonial America Module 4: Legislation and Politics in the Colonies
Confidential Print: Africa, 1834-1966
Confidential Print: Latin America, 1833-1969
Confidential Print: Middle East, 1839-1969
Confidential Print: North America, 1824-1961
Defining Gender, 1450-1910
East India Company
East India Company Module 2: Factory Records for South Asia and South East Asia
Eighteenth Century Drama
Eighteenth Century Journals: A Portal to Newspapers and Periodicals, 1685-1815
Eighteenth Century Journals: Module I Newspapers and Periodicals, 1693-1790
Eighteenth Century Journals: Module II Newspapers and Periodicals, 1699-1809
Eighteenth Century Journals: Module III Newspapers and Periodicals, 1680-1813
Eighteenth Century Journals: Module IV Newspapers and Periodicals, 1708-1817
Eighteenth Century Journals: Module V The Lady's Magazine and other titles, 1712-1832
Empire Online
Empire Online (DFG Nationallizenzen)
Empire Online Section 1
Empire Online Section 2
Empire Online Section 3
Empire Online Section 4

Empire Online Section 5
Everyday Life and Women in America, c.1800-1920
First World War Portal: Module I Personal Experiences
First World War Portal: Module II Propaganda & Recruitment
First World War Portal: Module III Visual Perspectives and Narratives
First World War: A Global Conflict
Foreign Office Files for China, 1919-1948
Foreign Office Files for China, 1919-1980
Foreign Office Files for China, 1949-1980
Foreign Office Files for India, Pakistan and Afghanistan, 1947-1980
Foreign Office Files for Japan
Foreign Office Files for Japan, 1919-1952: Occupation of Japan, 1946-1952
Foreign Office Files for the Middle East, 1971-1974: The 1973 Arab-Israeli War and the Oil Crisis
Foreign Office Files for the Middle East, 1975-1978: The Lebanese Civil War and the Camp David Accords
Foreign Office Files for the Middle East, 1979-1981: The Iranian Revolution and the Iran-Iraq War
Frontier Life
Gender: Identity and Social Change
Global Commodities: Trade, Exploration and Cultural Exchange
History of Mass Tourism
India, Raj and Empire
J. Walter Thompson: Advertising America
Jewish Life in America, c1654-1954: Sources from the American Jewish Historical Society, New York
Literary Manuscripts from the Berg Collection of the New York Public Library
Literary Manuscripts from the Brotherton Library
Literary Print Culture
London Low Life
Macmillan Cabinet Papers (1957-1963) (DFG Nationallizenzen)
Macmillan Cabinet Papers, 1957-1963
Mass Observation Online
Mass Observation Online: Module I
Mass Observation Online: Module II
Mass Observation Online: Module III
Mass Observation Online: Module IV
Medical Services and Warfare
Medieval Family Life: The Paston, Cely, Plumpton, Stonor and Armburgh Papers
Medieval Travel Writing
Meiji Japan: The Edward Sylvester Morse Collection from the Peabody Essex Museum, Salem
Migration to New Worlds Module 1: The Century of Immigration
Migration to New Worlds Module 2: The Modern Era
Migration to New Worlds: Module I The Century of Immigration

Nixon Years, 1969-1974
Perdita Manuscripts, 1500-1700
Popular Culture in Britain and America 1950-1975: Module I
Popular Culture in Britain and America 1950-1975: Module II
Popular Medicine in America, 1800-1900
Race Relations in America
Romanticism: Life, Literature and Landscape
Service Newspapers of World War Two
Shakespeare in Performance
Socialism on Film: Module I Wars & Revolutions
Socialism on Film: Module II Newsreels & Magazines
Socialism on Film: Module III Culture & Society
Socialism on Film: The Cold War and International Propaganda
The Grand Tour
The Police Gazette
Trade Catalogues and the American Home
Travel Writing, Spectacle and World History
Victorian Popular Culture: Circuses, Sideshows and Freaks
Victorian Popular Culture: Moving Pictures, Optical Entertainments and the Advent of Cinema
Victorian Popular Culture: Music Hall, Theatre and Popular Entertainment
Victorian Popular Culture: Spiritualism, Sensation and Magic
Virginia Company Archives
Women in the National Archives
Worlds Fairs
Africa Through Western Eyes
Cabinet Papers (1957-1964)
China Through Western Eyes
Church Missionary Society Archives
Crown Servants
East India Company Factory Records
Foreign Office Files for Japan and the Far East
Foreign Office Files: United States of America
Gilbert and Sullivan, Part 1: The Correspondence, Diaries, Literary Manuscripts and Prompt Copies of W. S. Gilbert (1836-1911) from the British Library, London
Japan Through Western Eyes
Keats, Leigh Hunt and Shelley
Plantation life in the Caribbean. Part 1, Jamaica, c1765-1848
Slave Trade Journals and Papers
Slavery, Abolition and Social Justice, 1490-2007
The History of Science and Technology. Series one, the Papers of Sir Hans Sloane, 1660-1753, from the British Library, London

AOSIS OpenJournals
AgEcon Search Free
AgriKnowledge Free
AgriKnowledge（アグリナレッジ） (A&I)
AIAA Aerospace Research Central Journals Archive
AIAA Conference Proceedings
AIP Conference Proceedings (American Institute of Physics)
AIP Digital Archive
AIP Journals (American Institute of Physics)
AIP Open Access Journals
AIP Scitation Acoustical Society of America
AIP Scitation American Association of Physics Teachers
AIP Scitation AVS the Science and Technology Society
AIP Scitation Journals Complete
AIP Scitation Laser Institute
AIP Scitation Society of Rheology
ASCE Research Library Proceedings
KB+ BIBSAM AIP Journals 2015-2017
KB+ BIBSAM AIP Other Society Titles 2015-2018
KB+ BIBSAM American Institute Of Physics AIP Journals 2018
KB+ BIBSAM American Institute Of Physics Other Society Titles 2018
KB+ BIBSAM American Institute Of Physics Other Society Titles 2019-2021
KB+ JISC Collections American Institute Of Physics Complete Collection 2014-2016
KB+ JISC Collections American Institute of Physics Complete Collection 2017-2019 (35 titles)
KB+ JISC Collections American Institute of Physics Complete Collection with extended backfile 2017-2019
KB+ Jisc Collections American Institute of Physics Journals Agreement 2020
KB+ Jisc Collections American Institute of Physics Journals Agreement Optional Title 2020
KB+ JISC Collections American Institute Of Physics With Extended Backfile 2014-2016
NESLi2 AIP Complete Standard Access
NESLi2 AIP Complete With Extended Backfiles
Chinese Electronic Periodical Service Conference Proceedings (Airiti Library)
Chinese Electronic Periodical Services (CEPS)
Chinese Electronic Periodical Services Basic & Applied Sciences
Chinese Electronic Periodical Services Bio Agriculture
Chinese Electronic Periodical Services Engineering
Chinese Electronic Periodical Services Health & Medical Care
Chinese Electronic Periodical Services Humanities
CJTD中國大陸期刊與會議論文
African Journals Online (Open Access)
Akademiai Kiado

Al Manhal eBook Collection
Al Manhal eDissertations Collection
Al Manhal eJournal Collection - Humanities & Social Sciences
Al Manhal eReports Collection
ALA ACRL Association of College and Research Libraries
Albertus Magnus College LibGuides
60 Minutes: 1997-2014
Academic Video Online: Eastern Edition
Academic Video Online: Premium - Australia
Academic Video Online: Premium - Canada
Academic Video Online: Premium - Japan
Academic Video Online: Premium - Outside North America
Academic Video Online: Premium - United States
African Diaspora, 1860-Present (Text) - All Titles
African Diaspora, 1860-Present (Video) - All Titles
African Diaspora, 1860-Present (Video) - Outside North America
Alexander Street Drama
Alexander Street Literature
America Latina en Video - Canada
America Latina en Video - China
America Latina en Video - Outside North America
America Latina en Video - United States
American History in Video - Australia
American History in Video - Canada
American History in Video - China
American History in Video - Outside North America
American History in Video - United States
American Newsreels in Video
Anthropological Fieldwork Online
Anthropology Online
APA PsycTherapy®
Art and Architecture in Video - Australia
Art and Architecture in Video - Canada
Art and Architecture in Video - Outside North America
Art and Architecture in Video - United States
Asian American Drama
Asian Film Online, Volume 1 - Australia
Asian Film Online, Volume 1 - Canada
Asian Film Online, Volume 1 - Outside North America
Asian Film Online, Volume 1 - United States

Asian Film Online, Volume 2 - Australia
Asian Film Online, Volume 2 - Canada
Asian Film Online, Volume 2 - Outside North America
Asian Film Online, Volume 2 - United States
Audio Drama: The L.A. Theatre Works Collection
Australasian Literature: Australia, New Zealand, and the Pacific Islands - Australia
Australasian Literature: Australia, New Zealand, and the Pacific Islands - Canada
Australasian Literature: Australia, New Zealand, and the Pacific Islands - Outside North America
Australasian Literature: Australia, New Zealand, and the Pacific Islands - United States
Australasian Video Online - Australia
Australasian Video Online - Canada
Australasian Video Online - Outside North America
Australasian Video Online - United States
BBC Horizon Collection
BBC Landmark Video Collection: First Edition
BBC Landmark Video Collection: Second Edition
BBC Literary Adaptations in Video
BBC Video Collection - Australia
BBC Video Collection - Canada
BBC Video Collection - Outside North America
BBC Video Collection - United States
Behavioral and Mental Health Online (Text)
Behavioral and Mental Health Online (Video) - Canada
Behavioral and Mental Health Online (Video) - China
Behavioral and Mental Health Online (Video) - Outside North America
Behavioral and Mental Health Online (Video) - United States
Black Drama: First Edition
Black Drama: Second Edition
Black Drama: Third Edition
Black Studies in Video - Australia
Black Studies in Video - Canada
Black Studies in Video - China
Black Studies in Video - Middle East
Black Studies in Video - Outside North America
Black Studies in Video - United States
Black Thought and Culture
Border and Migration Studies Online (Text)
Border and Migration Studies Online (Video) - Australia
Border and Migration Studies Online (Video) - Canada
Border and Migration Studies Online (Video) - Outside North America

Border and Migration Studies Online (Video) - United States
British and Irish Women's Letters and Diaries: First Edition (Pre-January 2009 Purchasers Only)
British and Irish Women's Letters and Diaries: Second Edition
BroadwayHD Collection
Business Ebooks Online
Business Education in Video - North America
Business Education in Video - Outside North America
Caribbean Studies in Video: The Banyan Archive - Australia
Caribbean Studies in Video: The Banyan Archive - Canada
Caribbean Studies in Video: The Banyan Archive - Outside North America
Caribbean Studies in Video: The Banyan Archive - United States
Civil War Letters and Diaries
CNN Video Collection
Contemporary World Drama
Counseling and Psychotherapy Transcripts, Volume 1
Counseling and Psychotherapy Transcripts, Volume 2
Counseling and Therapy in Video, Classic
Counseling and Therapy in Video: Volume 1
Counseling and Therapy in Video: Volume 2
Counseling and Therapy in Video: Volume 3
Counseling and Therapy in Video: Volume 4 (Text)
Counseling and Therapy in Video: Volume 4 (Video) - Australia
Counseling and Therapy in Video: Volume 4 (Video) - Canada
Counseling and Therapy in Video: Volume 4 (Video) - Outside North America
Counseling and Therapy in Video: Volume 4 (Video) - United States
Counseling and Therapy in Video: Volume 5
Counseling: John Whiteley Collection
Criminal Justice and Public Safety in Video - North America
Criminal Justice and Public Safety in Video - Outside North America
Criterion Collection - All Titles
Criterion Collection - Canada
Criterion Collection - United States
Current Affairs in Video - Australia
Current Affairs in Video - Canada
Current Affairs in Video - Outside North America
Current Affairs in Video - United States
Dance Online: Dance in Video, Volume 1
Dance Online: Dance in Video, Volume 2 - Australia
Dance Online: Dance in Video, Volume 2 - Canada
Dance Online: Dance in Video, Volume 2 - Outside North America

Dance Online: Dance in Video, Volume 2 - United States
Dance Online: Dance Studies Collection
Demand-Driven Acquisition
Demand-Driven Acquisition, Feature Film - Australia
Demand-Driven Acquisition, Feature Film - Canada
Demand-Driven Acquisition, Feature Film - China
Demand-Driven Acquisition, Feature Film - Outside North America
Demand-Driven Acquisition, Feature Film - United States
Demand-Driven Acquisition, Select - Australia
Demand-Driven Acquisition, Select - Canada
Demand-Driven Acquisition, Select - China
Demand-Driven Acquisition, Select - Outside North America
Demand-Driven Acquisition, Select - United States
Dental Education in Video
DER Essential
DER Expanded
DER New Releases
DER Premium
Diagnosing Mental Disorders: DSM-5™ and ICD-10
Digital Karl Barth Library
Digital Library of Classic Protestant Texts
Digital Library of the Catholic Reformation
Disability in the Modern World (Text)
Disability in the Modern World (Video)
Docuseek2 Complete Collection - Australia
Docuseek2 Complete Collection - Canada
Docuseek2 Complete Collection - Outside North America
Docuseek2 Complete Collection - United States
Docuseek2 Complete Collection: Second Edition - Australia
Docuseek2 Complete Collection: Second Edition - Canada
Docuseek2 Complete Collection: Second Edition - Outside North America
Docuseek2 Complete Collection: Second Edition - United States
Docuseek2 Essential Collection - Australia
Docuseek2 Essential Collection - Canada
Docuseek2 Essential Collection - Outside North America
Docuseek2 Essential Collection - United States
Docuseek2 International Collection - All Titles
Drama Texts Collection
Dutch Newsreels in Video
Early Encounters in North America

Early Experiences in Australasia: Primary Sources and Personal Narratives
Education in Video, Volume 1 - North America
Education in Video, Volume 1 - Outside North America
Education in Video, Volume 2 - Australia
Education in Video, Volume 2 - Canada
Education in Video, Volume 2 - Outside North America
Education in Video, Volume 2 - United States
Engineering Case Studies Online (Text)
Engineering Case Studies Online (Video) - Australia
Engineering Case Studies Online (Video) - Canada
Engineering Case Studies Online (Video) - China
Engineering Case Studies Online (Video) - Outside North America
Engineering Case Studies Online (Video) - United States
Environmental Issues Online (Text)
Environmental Issues Online (Video) - Australia
Environmental Issues Online (Video) - Canada
Environmental Issues Online (Video) - Outside North America
Environmental Issues Online (Video) - United States
Environmental Studies in Video - Australia
Environmental Studies in Video - Canada
Environmental Studies in Video - Outside North America
Environmental Studies in Video - United States
Ethnographic Sound Archives Online
Ethnographic Video Online, Teaching Edition (Text)
Ethnographic Video Online, Teaching Edition (Video)
Ethnographic Video Online, Volume 1 - Australia
Ethnographic Video Online, Volume 1 - Canada
Ethnographic Video Online, Volume 1 - China
Ethnographic Video Online, Volume 1 - Malaysia
Ethnographic Video Online, Volume 1 - Middle East
Ethnographic Video Online, Volume 1 - Outside North America
Ethnographic Video Online, Volume 1 - United States
Ethnographic Video Online, Volume 1 (Purchasers/Subscribers Post Sept. 2015) - Australia
Ethnographic Video Online, Volume 1 (Purchasers/Subscribers Post Sept. 2015) - Canada
Ethnographic Video Online, Volume 1 (Purchasers/Subscribers Post Sept. 2015) - Outside North America
Ethnographic Video Online, Volume 1 (Purchasers/Subscribers Post Sept. 2015) - United States
Ethnographic Video Online, Volume 2 - Australia
Ethnographic Video Online, Volume 2 - Canada
Ethnographic Video Online, Volume 2 - Outside North America
Ethnographic Video Online, Volume 2 - United States

Ethnographic Video Online, Volume 2 (Purchasers/Subscribers Post Sept. 2015) - Australia
Ethnographic Video Online, Volume 2 (Purchasers/Subscribers Post Sept. 2015) - Canada
Ethnographic Video Online, Volume 2 (Purchasers/Subscribers Post Sept. 2015) - Outside North America
Ethnographic Video Online, Volume 2 (Purchasers/Subscribers Post Sept. 2015) - United States
Ethnographic Video Online, Volume 3 - Australia
Ethnographic Video Online, Volume 3 - Canada
Ethnographic Video Online, Volume 3 - China
Ethnographic Video Online, Volume 3 - Outside North America
Ethnographic Video Online, Volume 3 - United States
Ethnographic Video Online, Volume 4 - Australia
Ethnographic Video Online, Volume 4 - Canada
Ethnographic Video Online, Volume 4 - Outside North America
Ethnographic Video Online, Volume 4 - United States
Evidence Based Acquisition
Fashion Studies Online: The Videofashion Library
Film Platform Collection - Canada
Film Platform Collection - Outside North America
Film Platform Collection - United States
Film Scripts Online, Volume 1
Film Scripts Online, Volume 2
Filmakers Library Online, Second Edition - North America
Filmakers Library Online, Second Edition - Outside North America
Filmakers Library Online, Volume 1 - North America
Filmakers Library Online, Volume 1 - Outside North America
Filmakers Library Online, Volume 2 - North America
Filmakers Library Online, Volume 2 - Outside North America
Filmakers Library Online, Volume 3
Filmakers Library Online, Volume 4 - Canada
Filmakers Library Online, Volume 4 - Outside North America
Filmakers Library Online, Volume 4 - United States
Food Studies Online (Text)
Food Studies Online (Video) - Australia
Food Studies Online (Video) - Canada
Food Studies Online (Video) - China
Food Studies Online (Video) - Outside North America
Food Studies Online (Video) - United States
French Newsreels in Video
Gilded Age
Harper's Weekly: 1857-1912
Health and Society in Video - Australia

Health and Society in Video - Canada
Health and Society in Video - Outside North America
Health and Society in Video - United States
Human Resource Management Online (Text)
Human Resource Management Online (Video) - Australia
Human Resource Management Online (Video) - Canada
Human Resource Management Online (Video) - Outside North America
Human Resource Management Online (Video) - United States
Human Rights Studies Online (Text)
Human Rights Studies Online (Video) - Australia
Human Rights Studies Online (Video) - Canada
Human Rights Studies Online (Video) - Outside North America
Human Rights Studies Online (Video) - United States
Illustrated Civil War Newspapers and Magazines
Images of America
Images of the American Civil War
Independent World Cinema: Classic and Contemporary Film - Canada
Independent World Cinema: Classic and Contemporary Film - Outside North America
Independent World Cinema: Classic and Contemporary Film - United States
International Business Online (Text)
International Business Online (Video) - All Titles
International Business Online (Video) - Australia
Japanese Newsreels in Video
Latin American Drama
LGBT Studies in Video - Australia
LGBT Studies in Video - Canada
LGBT Studies in Video - China
LGBT Studies in Video - Outside North America
LGBT Studies in Video - United States
LGBT Thought and Culture
Manuscript Women's Letters and Diaries
March of Time
Mass Incarceration and Prison Studies (Text)
Mass Incarceration and Prison Studies (Video)
Medical Imaging in Video
Meet the Press
Music Online: African American Music Reference
Music Online: American Music - Australia
Music Online: American Music - Canada
Music Online: American Music - Japan

Music Online: American Music - Outside North America
Music Online: American Music - United States
Music Online: Classical Music in Video
Music Online: Classical Music Library - Australia
Music Online: Classical Music Library - Canada
Music Online: Classical Music Library - Japan
Music Online: Classical Music Library - Outside North America
Music Online: Classical Music Library - United States
Music Online: Classical Music Reference Library
Music Online: Classical Performance In Video
Music Online: Classical Scores Library, Volume 1
Music Online: Classical Scores Library, Volume 1 - DFG
Music Online: Classical Scores Library, Volume 2
Music Online: Classical Scores Library, Volume 3
Music Online: Classical Scores Library, Volume 4
Music Online: Contemporary World Music - All Titles
Music Online: Contemporary World Music - Outside North America
Music Online: Jazz Music Library - Australia
Music Online: Jazz Music Library - Canada
Music Online: Jazz Music Library - Japan
Music Online: Jazz Music Library - Outside North America
Music Online: Jazz Music Library - United States
Music Online: Music Periodicals of the 19th Century
Music Online: Opera in Video - All Titles
Music Online: The Garland Encyclopedia of World Music
Music Video Collection - Canada
Music Video Collection - China
Music Video Collection - Outside North America
Music Video Collection - United States
National Theatre Collection
New World Cinema: Independent Features & Shorts, 1990-Present - Canada
New World Cinema: Independent Features & Shorts, 1990-Present - United States
North American Immigrant Letters, Diaries and Oral Histories
North American Indian Drama: First Edition (Pre-October 2012 Purchasers Only)
North American Indian Drama: Second Edition
North American Indian Thought and Culture
North American Women's Drama
North American Women's Drama: Second Edition
North American Women's Letters And Diaries
Nursing and Mental Health in Video: A Symptom Media Collection

Nursing Assessment in Video
Nursing Assistant Education in Video
Nursing Education in Video - All Titles
Nursing Education in Video, 2013
Nursing Education in Video, 2014
Nursing Education in Video, 2015
Nursing Education in Video: Second Edition
Nursing Education in Video: Third Edition
Occupational Therapy: Physical Modalities
Oral History Online
PBS Video Collection: Fourth Edition
PBS Video Collection: Second Edition - All Titles
PBS Video Collection: Third Edition - All Titles
Performance Design Archive Online
Popular Music Library - All Titles
Psychological Experiments Online (Text)
Psychological Experiments Online (Video) - Australia
Psychological Experiments Online (Video) - Canada
Psychological Experiments Online (Video) - China
Psychological Experiments Online (Video) - Outside North America
Psychological Experiments Online (Video) - United States
Psychotherapy.net Collection
Public Library Video Online: Premium
Rehabilitation Therapy in Video
Revolution and Protest Online (Text)
Revolution and Protest Online (Video) - Australia
Revolution and Protest Online (Video) - Canada
Revolution and Protest Online (Video) - Outside North America
Revolution and Protest Online (Video) - United States
Romantic Era Redefined
Romanticism Redefined: Pickering & Chatto and The Wordsworth
Royal Shakespeare Company Collection (Video)
Security Issues Online (Text)
Security Issues Online (Video) - All Titles
Security Issues Online (Video) - Australia
Security Issues Online (Video) - Outside North America
Silent Film Online - All Titles
Silent Film Online - Canada
Silent Film Online - United States
Sixties: Primary Documents and Personal Narratives 1960-1974 (Text)

Sixties: Primary Documents and Personal Narratives 1960-1974 (Video)
Smithsonian Global Sound for Libraries
Social Theory: First Edition (Pre-April 2014 Purchasers Only)
Social Theory: Second Edition
Social Work Online (Text)
Social Work Online (Video) - Australia
Social Work Online (Video) - Canada
Social Work Online (Video) - Outside North America
Social Work Online (Video) - United States
Sports Medicine and Exercise Science in Video, Volume 1
Sports Medicine and Exercise Science in Video, Volume 2
Teachers TV from Education in Video
Theatre in Context Collection
Theatre in Video - CRKN
Theatre in Video, BBC
Theatre in Video, Supplement
Theatre in Video, Volume 1 - North America
Theatre in Video, Volume 1 - North America (Pre-June 2011 Purchasers Only)
Theatre in Video, Volume 1 - Outside North America
Theatre in Video, Volume 2 - Australia
Theatre in Video, Volume 2 - Canada
Theatre in Video, Volume 2 - Outside North America
Theatre in Video, Volume 2 - United States
Twentieth Century Advice Literature
Twentieth Century Drama, Second Edition
Twentieth Century North American Drama: First Edition (Pre-October 2012 Purchasers Only)
Twentieth Century North American Drama: Second Edition
Twentieth Century Religious Thought (with The Digital Karl Barth Library)
Twentieth Century Religious Thought, Volume 1: Christianity - All Titles
Twentieth Century Religious Thought, Volume 2: Islam - All Titles
Twentieth Century Religious Thought, Volume 3: Judaism - All Titles
Twentieth Century Religious Thought, Volume 4: Eastern Religions - All Titles
Underground and Independent Comics, Comix, and Graphic Novels: Volume 1
Underground and Independent Comics, Comix, and Graphic Novels: Volume 2
Veterinary Education in Video
Video Journal of Counseling and Therapy
Video Journal of Counseling and Therapy, 2014
Women and Social Movements in Modern Empires since 1820 (Text)
Women and Social Movements in Modern Empires since 1820 (Video)
Women and Social Movements in the United States, 1600-2000

Women and Social Movements in the United States, 1600-2000, edited by Judy Tzu-Chun Wu and Rebecca Plant, Fall 2019-Spring 2020
Women and Social Movements, International
Women and Social Movements, International (Video)
World History in Video - Australia
World History in Video - Canada
World History in Video - Outside North America
World History in Video - United States
World Newsreels Online: 1929-1966
World of Archie Comics Archive
Youth and Popular Culture Magazine Archive
Alfred Wegener Institute IR (ePIC)
Alfred Wegener Institute IR (ePIC)
Allegheny College Library Catalog
Allen Press American Accounting Association
Allen Press Miscellaneous
Ambrose Video 2.0
BBC Shakespeare Plays
American Academy of Implant Dentistry Publications
American Academy of Pediatrics
American Academy of Pediatrics (AAP) eBooks
American Academy of Periodontology
American Association for Artificial Intelligence publications
American Association for Laboratory Animal Science Publications
Respiratory Care
American Association for the Advancement of Science
KB+ JISC Collections American Association For The Advancement Of Science 2018-2020
American Association of Immunologists Publications
American Association of Neurological Surgeons Publications
American Association of School Administrators
American Association on Intellectual and Developmental Disabilities
American Bar Association Online Journals
American Ceramic Society
ACS Academic Core Plus
ACS Guide to Scholarly Communication
American Chemical Society (ACS) Open Access
American Chemical Society Advances in Chemistry Series
American Chemical Society Journals
American Chemical Society Legacy Archive
American Chemical Society Symposium Series
BACON - American Chemical Society ACS - COUPERIN_FRONTLIST

CRKN American Chemical Society Journals
DRAA ACS Journals
JUSTICE American Chemical Society All Publication 2016
JUSTICE American Chemical Society All Publication 2020
KB+ BIBSAM American Chemical Society All 2014-2016
KB+ Jisc Collections American Chemical Society Web Editions 2015
Nesli2 American Chemical Society
BizJournals
American College of Physicians-ASIM
American Concrete Institute Journals
American Concrete Institute Online Journal Archives
American Economic Association
American Fisheries Society
American Health Consultants
American Health Lawyers Association Publications
American Institute of Aeronautics and Astronautics Publications
AIAA Journal Papers
DIPPR Project 801
American Institute of Mathematical Sciences (AIMS)
American Digital Archive of the Jewish Experience
American Judges Association Publications
ALA Techsource
American Library Association
ChoiceReviews online
American Marketing Association
American Mathematical Society eBooks
American Mathematical Society Journals
American Mathematical Society Publications (Freely Accessible)
AMS Books Online (Freely Accessible)
e-Contemporary Mathematics
KB+ Eduserv American Mathematical Society 2014-2016
KB+ Eduserv American Mathematical Society 2017-2019
mathjournals org
MathSciNet
Student Mathematical Library - 2017
University Lecture Series Backfile
American Medical Association Backfiles
American Medical Association Journals
JAMA Network Open Access
American Memory: The 19th Century in Print

American Mental Health Counselors Association Publications
American Meteorological Society
American Museum of Natural History Research Library
American Musical Instrument Society Publications
American Occupational Therapy Association Publications
American Physical Society [PROLA]
American Physical Society Journals
KB+ BIBSAM American Physical Society APS Journals 2017-2018
KB+ Bibsam American Physical Society APS Journals 2020
KB+ BIBSAM American Physical Society Journals 2015-2016
American Physiological Society
American Phytopathological Society Journal Back Issues
American Phytopathological Society Journals
DSM-5 Library
PsychiatryOnline Premium Package
DSM Premium
APA Books E-Collection 2010
APA Books E-Collection 2012
APA Books E-Collection 2013
APA Books E-Collection 2020
APA Handbook of Research Methods in Psychology
APA PsycBooks
APA PsycNET
PsycARTICLES
American Public Health Association
American Real Estate Society
American Roentgen Ray Society
American School Counselor Association
American Society for Bone and Mineral Research
American Society for Cell Biology
American Society for Clinical Investigation
American Society for Engineering Education Publications
American Society for Microbiology (ASM) Science
ASTM Standards
American Society of Agricultural and Biological Engineers
American Society of Animal Science
American Society of Civil Engineers:Jisc Collections:Journals:2015
American Society Of Civil Engineers ASCE eBooks
American Society of Civil Engineers ASCE E-books and Standards
American Society Of Civil Engineers ASCE Journals

American Society Of Civil Engineers ASCE Proceedings
American Society Of Civil Engineers ASCE Standards
American Society of Civil Engineers:NESLI2:Journals:2014
KB+ JISC Collections American Society of Civil Engineers Journals 2016-2017
KB+ Jisc Collections American Society of Civil Engineers Journals 2020
American Society of Clinical Oncology Journals
ASHRAE Publications
American Society of Hematology
Copeia Online
ASM Online Journals
American Speech Language Hearing Association
American Statistical Association Publications
American Thoracic Society Journals
AUC DAR Repository (Digital Archive and Research Repository)
American Veterinary Medical Association
ACP Journal Club PLUS
American Society for Microbiology eBooks 2015
American Society for Microbiology eBooks 2016
American Society for Microbiology eBooks 2017
American Society for Microbiology eBooks Frontlist
Ammons Scientific
Amsterdam UMC - Vrije Universiteit Amsterdam
Analysis and Policy Observatory - APO
Annual Reviews
Annual Reviews Back Volume Collection
Annual Reviews Economics Collection
Annual Reviews Open Access
Annual Reviews:Electronic Back Volume Collection:2013 - NESLI2
KB+ Jisc Collections Annual Reviews Agriculture Collection 2018-2020 Post 2008
KB+ Jisc Collections Annual Reviews Agriculture Collection 2018-2020 Pre 2008
KB+ Jisc Collections Annual Reviews Biomedical Collection 2018-2020 Post 2008
KB+ Jisc Collections Annual Reviews Biomedical Collection 2018-2020 Pre 2008
KB+ JISC Collections Annual Reviews Biomedical Life Sciences 2015-2017 Post 2008 (29 titles)
KB+ JISC Collections Annual Reviews Biomedical Life Sciences 2015-2017 Pre 2008
KB+ Jisc Collections Annual Reviews Complete Collection 2018-2020 Post 2008
KB+ Jisc Collections Annual Reviews Complete Collection 2018-2020 Pre 2008
KB+ JISC Collections Annual Reviews Economics 2015-2017 Post 2008
KB+ JISC Collections Annual Reviews Economics 2015-2017 Pre 2008
KB+ Jisc Collections Annual Reviews Economics Collections 2018-2020 Post 2008
KB+ Jisc Collections Annual Reviews Economics Collections 2018-2020 Pre 2008

KB+ Jisc Collections Annual Reviews Life Sciences Collection 2018-2020 Post 2008
KB+ Jisc Collections Annual Reviews Life Sciences Collection 2018-2020 Pre 2008
KB+ JISC Collections Annual Reviews Physical Sciences 2015-2017 Post 2008
KB+ JISC Collections Annual Reviews Physical Sciences 2015-2017 Pre 2008
KB+ Jisc Collections Annual Reviews Physical Sciences Collection 2018-2020 Post 2008
KB+ Jisc Collections Annual Reviews Physical Sciences Collection 2018-2020 Pre 2008
KB+ JISC Collections Annual Reviews Sciences 2015-2017 Post 2008
KB+ JISC Collections Annual Reviews Sciences 2015-2017 Pre 2008
KB+ JISC Collections Annual Reviews Sciences 2016-2017 Post 2008
KB+ JISC Collections Annual Reviews Sciences 2016-2017 Pre 2008
KB+ JISC Collections Annual Reviews Sciences Collection 2015-2017 Post 2008
KB+ JISC Collections Annual Reviews Sciences Collection 2015-2017 Pre 2008
KB+ Jisc Collections Annual Reviews Sciences Collection 2018-2020 Post 2008
KB+ Jisc Collections Annual Reviews Sciences Collection 2018-2020 Pre 2008
KB+ JISC Collections Annual Reviews Social Sciences 2015-2017 Post 2008
KB+ JISC Collections Annual Reviews Social Sciences 2015-2017 Pre 2008
KB+ Jisc Collections Annual Reviews Social Sciences Collections 2018-2020 Post 2008
KB+ Jisc Collections Annual Reviews Social Sciences Collections 2018-2020 Pre 2008
NESLi2 Annual Reviews
NESLi2 Annual Reviews Back Volume Collection
NESLi2 Sciences Collection of 34 Annual Reviews
ANSI Journals
DRAM (Database of Recorded American Music)
AnthroSource
ANU Press eTEXT
APA PsycCritiques
A-R Editions Music Publications
ARAN (Access to Research at NUI Galway)
Archaeology Data Service Journals, Series and Books
Archives of African Journals Free
ArchNet Digital Library
Ariez Tijdschriften
Armand-Colin journals
Ars Aequi Libri eBoeken
Ars Aequi maandblad- en KwartaalSignaalartikelen
Ars Aequi maandblad- en KwartaalSignaaledities
Rechtspraak Vreemdelingenrecht jurisprudentie
Uitgaven van Ars Aequi Libri als webboek
Artech House
ArtikelSök

Archaeology Data Service
CERN Document Server
Design Online
ARTstor
Association for Research in Vision and Ophthalmology
ADB Online Publications
L & B Digital Library
ASM Alloy Center
ASM Alloy Phase Diagram Center
ASM Corrosion Analysis Network
ASM Failure Analysis Center
ASM Handbooks Online
ASM Medical Materials Database
ASM Micrograph Center
EcoSal-Escherichia coli and Salmonella: Cellular and Molecular Biology
ASME Digital Collection Conference Proceedings
ASME Digital Collection eBooks
ASME Digital Collection Journals
ASME Digital Collection Journals Archive
Association for Learning Technology Publications
Association for Science Education Journals
Association for the Sciences of Limnology and Oceanography
Association for the Study of African American Life and History
AAMC Journals
Association of Departments of English (ADE)
Association of Energy Engineers Online Journals
ARL Digital Publications
ARL Publications
Public Health Reports
Association for Physical Education Member Journals
American Society for Testing and Materials: ASTM Journals
ASTM Manuals
ASTM Symposia Papers
AUB ScholarWorks
AES Electronic Library
AES Publications
Austlii Journal
Australian Mathematical Society
Research Data Australia (RDA)
Research Data Australia (RDA) Abstract

Research Data Australia (RDA) Full Text
ANU E Press
Austrian Newspapers Online (ANNO)
Arab World Geographer
Digital Commons
BMI Research
Ball State University Cardinal Scholar Institutional Repository
Ball State University Libguides
Bangladesh Journals Online
Bank of England
Munich DigitiZation Center (MDZ)
BC Decker
BCC Research
Beck eBibliothek Books
Begell eBooks Platform
Behavior Analyst Online
Baishideng "World Journal of" online journals
Beit Ariela Index of Daily Newspapers - יומית לעיתונות אריאלה בית מפתח
Bentham Subscription Based Books
Bentham Subscription Based Journals
KB+ Eduserv Bentham Science Chemistry Collection 2015-2017
KB+ Eduserv Bentham Science CMedical PubMed Collection 2015-2017
KB+ Eduserv Bentham Science Complete Collection 2015-2017
KB+ Eduserv Bentham Science Drug Design and Discovery Collection 2015-2017
KB+ Eduserv Bentham Science High Impact Collection 2015-2017
KB+ Eduserv Bentham Science Patents Collection 2015-2017
KB+ Eduserv Bentham Science Pharmacy Collection 2015-2017
Berghahn Journals
Jahresberichte für deutsche Geschichte
Biblical Archaeology Society
BiblicalStudies.org.uk
Bibliographie der deutschen Sprach - und Literaturwissenschaft
African American History
BiblioBoard Access (Open Access)
BiblioBoard Core Module
British Library 19th Century Module
EBooks Minnesota
Folk and Americana
Open Research Library (Open Access)
Spanish Language History and Literature

DONum
Gallica - All content.
Gallica - Open Access
Gallica Ebooks
Bibliothèques Virtuelles Humanistes
Biodiversity Heritage Library
Bioline International
Bacon Bioone Global Openaccess
BioOne 1
BioOne Complete
KB+ JISC Collections BioOne 2016-2018
Biophysical Society
BioScientifica
Society for Endocrinology
BJu Legal
BJu Tijdschriften Online
Blackwell Reference Online Complete
Bloomberg BNA Online Journals
Bloomsbury Academic: Screen Studies
Bloomsbury Design Library
Bloomsbury Fairchild Library
Churchill Archive
Bloomsbury Academic Collections series: Biblical Studies Landmark Texts
Bloomsbury Academic Collections series: Literary Criticism Landmark Texts
Bloomsbury Academic Collections series: Philosophy Landmark Texts
Bloomsbury Applied Visual Arts
Bloomsbury Architecture Library
Bloomsbury Collections All Titles
Bloomsbury Collections Anthropology 2017
Bloomsbury Collections Anthropology 2018
Bloomsbury Collections Biblical Studies 2017
Bloomsbury Collections Biblical Studies 2017
Bloomsbury Collections Classical Studies And Archaeology 2017
Bloomsbury Collections Classical Studies And Archaeology 2018
Bloomsbury Collections Education 2017
Bloomsbury Collections Education 2018
Bloomsbury Collections Film And Media Studies 2017
Bloomsbury Collections Film And Media Studies 2018
Bloomsbury Collections Hart Publishing 2017
Bloomsbury Collections Hart Publishing 2018

Bloomsbury Collections History 2017
Bloomsbury Collections History 2018
Bloomsbury Collections Linguistics 2017
Bloomsbury Collections Linguistics 2018
Bloomsbury Collections Literary Studies 2018
Bloomsbury Collections Philosophy 2017
Bloomsbury Collections Philosophy 2018
Bloomsbury Collections Religious Studies 2017
Bloomsbury Collections Religious Studies 2018
Bloomsbury Collections Theology 2017
Bloomsbury Collections: 18th- and 19th-Century Literature Archive 2006-2012
Bloomsbury Collections: Ancient Commentators on Aristotle
Bloomsbury Collections: Ancient Philosophy Archive 1984-2012
Bloomsbury Collections: Applied Linguistics Archive 2000-2012
Bloomsbury Collections: Biblical Studies 2015
Bloomsbury Collections: Biblical Studies 2016
Bloomsbury Collections: Business & Management
Bloomsbury Collections: Christian Doctrines Archive 1988-2013
Bloomsbury Collections: Christology Archive 1982-2011
Bloomsbury Collections: Classical Literature Archive 1994-2012
Bloomsbury Collections: Classical Studies & Archaeology 2013
Bloomsbury Collections: Classical Studies & Archaeology 2014
Bloomsbury Collections: Classical Studies & Archaeology 2015
Bloomsbury Collections: Classical Studies & Archaeology 2016
Bloomsbury Collections: Comparative Law, Legal History and Legal Studies
Bloomsbury Collections: Competition Law
Bloomsbury Collections: Constitutional and Administrative Law
Bloomsbury Collections: Contemporary Writing, Theory and Culture Archive 2006-2012
Bloomsbury Collections: Continuing Professional Development
Bloomsbury Collections: Contract, Tort and Restitution
Bloomsbury Collections: Corporate and Financial Law
Bloomsbury Collections: Criminal Law and Justice
Bloomsbury Collections: Early Modern History Archive 1971-2013
Bloomsbury Collections: Education 2013
Bloomsbury Collections: Education 2015
Bloomsbury Collections: Education 2016
Bloomsbury Collections: Education Around the World
Bloomsbury Collections: Energy, Environmental & Natural Resources Law
Bloomsbury Collections: Essential Histories (Osprey Publishing)
Bloomsbury Collections: European Law

Bloomsbury Collections: Family and Social Law
Bloomsbury Collections: Film Studies Archive 2000-2013
Bloomsbury Collections: Geographers Biobibliographical Studies
Bloomsbury Collections: Hart Publishing 2014
Bloomsbury Collections: Hart Publishing 2015
Bloomsbury Collections: Hart Publishing 2016
Bloomsbury Collections: History 2014
Bloomsbury Collections: Human Rights Law
Bloomsbury Collections: Intellectual Property Law
Bloomsbury Collections: International Critical Commentary 1901-2014
Bloomsbury Collections: International Law
Bloomsbury Collections: International Relations Archive 1998-2013
Bloomsbury Collections: Labour & Discrimination Law
Bloomsbury Collections: Legal Philosophy
Bloomsbury Collections: Linguistics 2013
Bloomsbury Collections: Linguistics 2014
Bloomsbury Collections: Linguistics 2015
Bloomsbury Collections: Literary Studies 2013
Bloomsbury Collections: Literary Studies 2014
Bloomsbury Collections: Literary Studies 2015
Bloomsbury Collections: Litigation & Civil Procedure
Bloomsbury Collections: Media Studies Archive 2008-2013
Bloomsbury Collections: Medical Law & Ethics
Bloomsbury Collections: Medieval History Archive 1981-2013
Bloomsbury Collections: Modernism Archive 2005-2012
Bloomsbury Collections: Pauline Studies Archive 1984-2012
Bloomsbury Collections: Philosophy 2013
Bloomsbury Collections: Philosophy 2014
Bloomsbury Collections: Philosophy 2015
Bloomsbury Collections: Philosophy of Education Archive 2008-2012
Bloomsbury Collections: Political Theory and Philosophy Archive 1998-2013
Bloomsbury Collections: Politics & International Relations 2015
Bloomsbury Collections: Politics and International Relations 2014
Bloomsbury Collections: Politics Archive 1993-2013
Bloomsbury Collections: Poyser Monographs
Bloomsbury Collections: Religious Studies 2014
Bloomsbury Collections: Religious Studies 2015
Bloomsbury Collections: Religious Studies Archive 2008-2012
Bloomsbury Collections: Second Language Acquisition Archive 2004-2012
Bloomsbury Collections: Shakespeare Archive 2003-2012

Bloomsbury Collections: The Churchill Collection
Bloomsbury Collections: The Law of Property and Trusts
Bloomsbury Collections: Theology 2015
Bloomsbury Collections: Theology 2016
Bloomsbury Cultural History 2018-19 Collection
Bloomsbury Drama Online Core Collection UK
Bloomsbury Drama Online: The RSC Live Collection 1 (2013-17)
Bloomsbury Encyclopedia of Philosophers
Bloomsbury Fashion Video Archive
Bloomsbury Food Library
Bloomsbury Human Kinetics Library
Bloomsbury Open Access
Bloomsbury Popular Music
Arcadian Library Online
Berg Fashion Library
BMJ Clinical Evidence
BMJ Journals
BMJ Journals (EIRA)
BMJ Journals Online Archive
BMJ Open Access Journals
DRAA British Medical Journals (BMJ)
EZB-NALGM-00452 BMJ Journals Archive NL
KB+ BIBSAM British Medical Journals 2017-2019
KB+ BIBSAM British Medical Journals 2020
KB+ BIBSAM British Medical Journals Complete 2017-2019
KB+ BIBSAM British Medical Journals Complete 2020
KB+ JISC Collections British Medical Journal BMJ Journals Online Collection 2019-2020
KB+ JISC Collections British Medical Journal Journal Collection 2015-2016
KB+ JISC Collections British Medical Journal Journal Collection 2017-2018
KB+ JISC Collections British Medical Journal Optional Access Journals 2015-2016
KB+ JISC Collections British Medical Journal Optional Access Journals 2017-2018
KB+ JISC Collections British Medical Journal Optional Additional Titles 2019-2020
Best Practice
BMJ Best Practice
BNI
Böhlau Verlag OpenAccess
50 Lessons
Books24x7 BusinessPro
Books24x7 EngPro
Books24x7 ExecBlueprint

Books24x7 ExecSummaries
Books24x7 FinancePro
Books24x7 GovEssentials
Books24x7 ITPro
Books24x7 OfficeEssentials
Books24x7 Well-BeingEssentials
Skillsoft IT and Desktop Videos
Book Up
Boston College Open Access Journals
Boston University on behalf of the Center for Psychiatric Rehabilitation
BGSU Digital Resource Commons
Brage Consortium Repository
Année philologique
Brepols Books
Brepols journals
Année Philologique
Brepols Miscellanea Online - Collection 2016
Dictionnaire d'histoire et de géographie ecclésiastiques (DHGE)
Bridgeman Education
Bridgeman Education (Bridgeman Art Library)
Archives of the Church of Uganda Online
Art Sales Catalogue Online
Art Sales Catalogues Online (DFG Nationallizenzen)
Book Sales Catalogues Online
Brill - Recueil des cours de l''Académie de droit international de La Haye
Brill Biology Journal Collection 2018
Brill Biology Journal Collection 2019
Brill Encyclopedia of Early Christianity Online
Brill Humanities & Social Sciences Journal Collection 2019
Brill International Law & Human Rights Journal Collection 2019
Brill Journal Archives Online Part 1: Humanities & Social Sciences Collection
Brill Journal Archives Online Part 2: Humanities & Social Sciences Collection
Brill Middle East & Islamic Studies Journal Collection 2018
Brill Online Books and Journals
Brill Religious Studies Journal Collection 2019
Brill Research Perspectives Journal Collection 2018
Brill Research Perspectives Journal Collection 2019
Brill Shared Ebook Collection - SCELC
Brill.com
Brill's Africa Yearbook Online

Brill''s New Pauly
Chinese Film and Newsreel Scripts from the Cultural Revolution Online
Classic Mexican Cinema
Codices Vossiani Latini Online
Cuban Culture and Cultural Relations
Early Modern Pamphlets Online (TEMPO) - Dutch Pamphlets
Early Western Korans
Encyclopaedia of Islam
Encyclopaedia of Islam THREE
Encyclopedia of Hebrew Language and Linguistics
Ephraim Deinard (1846-1930) Online
Human Rights and Humanitarian Law E-Books Online, Collection 2017
Human Rights Documents Online
Japan Chronicle
Jewish Theater under Stalinism
Lithographed Editions of Firdawsi's Shahnamah Online
Manchuria Daily News Online
Mass Media in Russia, 1908-1918
Middle Eastern Manuscripts Online 2: The Ottoman Legacy of Levinus Warner
Missionary Archives from Lesotho, 1832 - 2006
Mobilizing East Asia Online
Muslims in Russia
North China Daily News Online
North China Standard Online
Prize Papers Online 1: American Revolutionary War and Fourth Anglo-Dutch War
Prize Papers Online 2: Seven Years’ War and War of the Austrian Succession
Prize Papers Online 3: First, Second and Third Anglo-Dutch War and War of the Spanish Succession
Prize Papers Online: Atlas
Publications of the Hague Academy of International Law
Religion in Geschichte und Gegenwart
Religion Past and Present
Russian Anarchist Periodicals of the Early 20th Century
Russian Military Intelligence on Asia: Archive Series 1651-1917
Russian Military Intelligence on Asia: Secret Prints 1883-1914
Sacramentum Mundi Online
Sephardic Editions, 1550-1820 Online
Soviet Cinema. Film Periodicals 1918-1942:Parts 1 (Journals) and 2 (Newspapers)
Soviet Cinema: Archival Documents from RGALI 1923-1935
Studies on the Texts of the Desert of Judah
Textual History of the Bible

The Guatemala Collection
U.S. Intelligence on the Middle East, 1945-2009
US Intelligence on Europe, 1945-1995
Weapons of Mass Destruction
World of Children – Artek Pioneer Camp Archives, 1944-1967 Online
Brill Online reference works
New Pauly Online
Bacon Brill France Istexjournals
Bacon Brill France Istexradi
Brill - All E-Book Titles
Brill Martinus Nijhoff E-Books International Law Collection
Brill Online E-Books Anti-Calvin Online
Brill Online E-Books Archives Of The Church In North India: Archival Collection
Brill Online E-Books Archives Of The Church In North India: Monograph Collection
Brill Online E-Books Archives Of The Presbyterian Church Of Cuba Online
Brill Online E-Books Asian Studies 2007
Brill Online E-Books Asian Studies 2008
Brill Online E-Books Asian Studies 2009
Brill Online E-Books Asian Studies 2010
Brill Online E-Books Asian Studies 2011
Brill Online E-Books Asian Studies 2012
Brill Online E-Books Asian Studies 2013
Brill Online E-Books Asian Studies 2014
Brill Online E-Books Asian Studies 2015
Brill Online E-Books Asian Studies 2016
Brill Online E-Books Asian Studies 2017
Brill Online E-Books Asian Studies 2018
Brill Online E-Books Biblical Studies, Ancient Near East and Early Christianity 2007
Brill Online E-Books Biblical Studies, Ancient Near East and Early Christianity 2008
Brill Online E-Books Biblical Studies, Ancient Near East and Early Christianity 2009
Brill Online E-Books Biblical Studies, Ancient Near East and Early Christianity 2010
Brill Online E-Books Biblical Studies, Ancient Near East and Early Christianity 2011
Brill Online E-Books Biblical Studies, Ancient Near East and Early Christianity 2012
Brill Online E-Books Biblical Studies, Ancient Near East and Early Christianity 2013
Brill Online E-Books Biblical Studies, Ancient Near East and Early Christianity 2015
Brill Online E-Books Biblical Studies, Ancient Near East and Early Christianity 2016
Brill Online E-Books Biblical Studies, Ancient Near East and Early Christianity 2017
Brill Online E-Books Biblical Studies, Ancient Near East and Early Christianity 2018
Brill Online E-Books Biblical Studies, Ancient Near East and Early Christianity 2019
Brill Online E-Books Biblical Studies, Ancient Near East and Early Christianity Collection

Brill Online E-Books Biology 2007-2010
Brill Online E-Books Biology 2011
Brill Online E-Books Biology 2012
Brill Online E-Books Biology 2013
Brill Online E-Books Biology 2014
Brill Online E-Books Biology 2015
Brill Online E-Books Biology 2016
Brill Online E-Books Biology Collection
Brill Online E-Books Children'S Leisure Activities In Russia
Brill Online E-Books Chinese Students' Monthly Online
Brill Online E-Books Classical Studies 2007
Brill Online E-Books Classical Studies 2008
Brill Online E-Books Classical Studies 2009
Brill Online E-Books Classical Studies 2010
Brill Online E-Books Classical Studies 2011
Brill Online E-Books Classical Studies 2012
Brill Online E-Books Classical Studies 2013
Brill Online E-Books Classical Studies 2014
Brill Online E-Books Classical Studies 2015
Brill Online E-Books Classical Studies 2016
Brill Online E-Books Classical Studies 2017
Brill Online E-Books Classical Studies 2019
Brill Online E-Books Classical Studies Collection
Brill Online E-Books Climate Change And Law Collection
Brill Online E-Books Codices Hugeniani Online
Brill Online E-Books Cold War Intelligence
Brill Online E-Books Colonial Period Korea Online
Brill Online E-Books Conrad Gessner'S Private Library Online
Brill Online E-Books Critical Editions Of The New Testament Online
Brill Online E-Books Cult Of Body
Brill Online E-Books Early Russian Cinema Online
Brill Online E-Books European History and Culture 1 2014
Brill Online E-Books European History and Culture 1 2015
Brill Online E-Books European History and Culture 1 2016
Brill Online E-Books European History and Culture 1 2017
Brill Online E-Books European History and Culture 1 2018
Brill Online E-Books European History and Culture 2 2014
Brill Online E-Books European History and Culture 2 2015
Brill Online E-Books European History and Culture 2 2016
Brill Online E-Books European History and Culture 2 2017

Brill Online E-Books European History and Culture 2 2018
Brill Online E-Books European History and Culture 2 2019
Brill Online E-Books European History and Culture 2007
Brill Online E-Books European History and Culture 2008
Brill Online E-Books European History and Culture 2009
Brill Online E-Books European History and Culture 2010
Brill Online E-Books European History and Culture 2011
Brill Online E-Books European History and Culture 2012
Brill Online E-Books European History and Culture 2013
Brill Online E-Books European History and Culture Collection
Brill Online E-Books French Revolutionary Opinions Online
Brill Online E-Books Girolamo Savonarola Online
Brill Online E-Books Global Oriental Collection
Brill Online E-Books Hebrew And Judeo-Arabic Printing In Baghdad
Brill Online E-Books Hebrew, Judeo-Arabic, And Marathi Jewish Printing In India Online
Brill Online E-Books Heinrich Bullinger'S Original Publications
Brill Online E-Books Historical Garden Design Online
Brill Online E-Books History Of Modern Russian And Ukrainian Art
Brill Online E-Books Human Rights and Humanitarian Law
Brill Online E-Books Human Rights and Humanitarian Law 2006
Brill Online E-Books Human Rights and Humanitarian Law 2007
Brill Online E-Books Human Rights and Humanitarian Law 2008
Brill Online E-Books Human Rights and Humanitarian Law 2009
Brill Online E-Books Human Rights and Humanitarian Law 2010
Brill Online E-Books Human Rights and Humanitarian Law 2011
Brill Online E-Books Human Rights and Humanitarian Law 2012
Brill Online E-Books Human Rights and Humanitarian Law 2013
Brill Online E-Books Human Rights and Humanitarian Law 2014
Brill Online E-Books Human Rights and Humanitarian Law 2015
Brill Online E-Books Human Rights and Humanitarian Law 2016
Brill Online E-Books Human Rights and Humanitarian Law 2017
Brill Online E-Books Human Rights and Humanitarian Law 2018
Brill Online E-Books Human Rights and Humanitarian Law 2019
Brill Online E-Books Humanities and Social Sciences 2014
Brill Online E-Books Humanities and Social Sciences 2015
Brill Online E-Books Humanities and Social Sciences 2016
Brill Online E-Books Humanities and Social Sciences 2017
Brill Online E-Books Humanities and Social Sciences Collection
Brill Online E-Books Hungarian Reformation Ebook Collection
Brill Online E-Books Imperial Russia'S Illustrated Press

Brill Online E-Books International Law 2006
Brill Online E-Books International Law 2007
Brill Online E-Books International Law 2008
Brill Online E-Books International Law 2009
Brill Online E-Books International Law 2010
Brill Online E-Books International Law 2011
Brill Online E-Books International Law 2012
Brill Online E-Books International Law 2013
Brill Online E-Books International Law 2014
Brill Online E-Books International Law 2015
Brill Online E-Books International Law 2016
Brill Online E-Books International Law 2017
Brill Online E-Books International Law 2018
Brill Online E-Books International Law 2019
Brill Online E-Books Israel'S Messenger Online
Brill Online E-Books Language and Linguistics 2011
Brill Online E-Books Language and Linguistics 2012
Brill Online E-Books Language and Linguistics 2013
Brill Online E-Books Language and Linguistics 2014
Brill Online E-Books Language and Linguistics 2015
Brill Online E-Books Language and Linguistics 2016-2017
Brill Online E-Books Language and Linguistics 2018
Brill Online E-Books Language and Linguistics Collection
Brill Online E-Books Latin American Anarchist And Labour Periodicals Online
Brill Online E-Books Literature and Cultural Studies 2007
Brill Online E-Books Literature and Cultural Studies 2008
Brill Online E-Books Literature and Cultural Studies 2009
Brill Online E-Books Literature and Cultural Studies 2010
Brill Online E-Books Literature and Cultural Studies 2011
Brill Online E-Books Literature and Cultural Studies 2012
Brill Online E-Books Literature and Cultural Studies 2013
Brill Online E-Books Literature and Cultural Studies 2014
Brill Online E-Books Literature and Cultural Studies 2015
Brill Online E-Books Literature and Cultural Studies 2016
Brill Online E-Books Literature and Cultural Studies 2017
Brill Online E-Books Literature and Cultural Studies 2018
Brill Online E-Books Literature and Cultural Studies 2019
Brill Online E-Books Middle East and Islamic Studies 2007
Brill Online E-Books Middle East and Islamic Studies 2008
Brill Online E-Books Middle East and Islamic Studies 2009

Brill Online E-Books Middle East and Islamic Studies 2010
Brill Online E-Books Middle East and Islamic Studies 2011
Brill Online E-Books Middle East and Islamic Studies 2012
Brill Online E-Books Middle East and Islamic Studies 2013
Brill Online E-Books Middle East and Islamic Studies 2014
Brill Online E-Books Middle East and Islamic Studies 2015
Brill Online E-Books Middle East and Islamic Studies 2016
Brill Online E-Books Middle East and Islamic Studies 2017
Brill Online E-Books Middle East and Islamic Studies 2018
Brill Online E-Books Middle East and Islamic Studies 2019
Brill Online E-Books Middle Eastern Manuscripts Online 1: Pioneer Orientalists
Brill Online E-Books Middle Eastern Manuscripts Online 3: Arabic Manuscripts From The Hungarian Academy
Brill Online E-Books Moses Maimonides, Unparalleled Editions Online
Brill Online E-Books North China Herald
Brill Online E-Books Philipp Melanchthon, Theologian And Humanist
Brill Online E-Books Popular Literature, Fiction And Songs In Russia
Brill Online E-Books Reformation In Heidelberg Online
Brill Online E-Books Reformed Protestantism: East Friesland And North West Germany Online
Brill Online E-Books Religious Studies Theology and Philosophy 2007
Brill Online E-Books Religious Studies Theology and Philosophy 2008
Brill Online E-Books Religious Studies Theology and Philosophy 2009
Brill Online E-Books Religious Studies Theology and Philosophy 2010
Brill Online E-Books Religious Studies Theology and Philosophy 2011
Brill Online E-Books Religious Studies Theology and Philosophy 2012
Brill Online E-Books Religious Studies Theology and Philosophy 2013
Brill Online E-Books Religious Studies Theology and Philosophy 2014
Brill Online E-Books Religious Studies Theology and Philosophy 2015
Brill Online E-Books Religious Studies Theology and Philosophy 2016
Brill Online E-Books Religious Studies Theology and Philosophy 2017
Brill Online E-Books Religious Studies Theology and Philosophy 2018
Brill Online E-Books Religious Studies, Theology and Philosophy Collection
Brill Online E-Books Richard Wagner Online
Brill Online E-Books Russian Avant-Garde, 1904-1946
Brill Online E-Books Russian Theater In The Early 20Th Century
Brill Online E-Books Russian-Ottoman Relations Part 1
Brill Online E-Books Russian-Ottoman Relations Part 2
Brill Online E-Books Russian-Ottoman Relations Part 3
Brill Online E-Books Russian-Ottoman Relations Part 4
Brill Online E-Books Screen And Stage
Brill Online E-Books Sixteenth Century Pamphlets Online / Flugschriften Online

Brill Online E-Books Slavonic Bibles
Brill Online E-Books Social Sciences 2007
Brill Online E-Books Social Sciences 2008
Brill Online E-Books Social Sciences 2009
Brill Online E-Books Social Sciences 2010
Brill Online E-Books Social Sciences 2011
Brill Online E-Books Social Sciences 2012
Brill Online E-Books Social Sciences 2013
Brill Online E-Books Social Sciences 2014
Brill Online E-Books Social Sciences 2015
Brill Online E-Books Social Sciences 2016
Brill Online E-Books Social Sciences 2017-1
Brill Online E-Books Social Sciences 2017-2
Brill Online E-Books Social Sciences 2018
Brill Online E-Books Social Sciences 2019
Brill Online E-Books Social Sciences Collection
Brill Online E-Books The Huguenots Online
Brill Online E-Books The Italian Reformation Online
Brill Online E-Books Translations Of The Peking Gazette Online
Brill Online E-Books U.S. Intelligence On Asia, 1945-1991
Brill Online E-Books Western Books On China Up To 1850 Online
Brill Online E-Books Western Travellers In The Islamic World Ebook Collection
Brill Online E-Books World Council Of Churches Online: World War Ii Era Records
Brill Online E-Books World Of Children - Artek Pioneer Camp Archives, 1944-1967 Online (Dfg Nationallizenzen)
Brill Online E-Books Yearbook Of The Imperial Theaters
Brill Online Journals
Brill Online Journals Archive Part 1
Brill Online Journals Archive Part 2
Brillonline Open Access Books
Brillonline Open Access Journals
KB+ BIBSAM BrillOnline Journals 2014-2016
KB+ BIBSAM BrillOnline Journals 2017-2019
KB+ BIBSAM BrillOnline Journals 2020
KB+ Brill:JISC Collections: Journals Archive:2013-2018
KB+ JISC Collections Brill Collection 2015
KB+ JISC Collections Brill Collection 2016
KB+ JISC Collections Brill Collection 2017
KB+ JISC Collections Brill Journals 2018
KB+ JISC Collections Brill Journals 2019
NESLi2 Brill Online Journals

Britannica Online Japan
British Editorial Society of Bone and Joint Surgery
British Herpetological Society Publications
British Institute of Radiology Journals
British Journal of Radiology - Archived collection
British Journal of Hospital Medicine
EThOS: Electronic Theses Online Service
EThOS: Electronic Theses Online Service - Abstract
EThOS: Electronic Theses Online Service Full Text
Broadcast Education Association Publications
Center For Digital Initiatives
BURA DSpace
British Standards Online
Builder Group Publications
Building Green
Business Expert Press eBooks
Berliner Wissenschafts-Verlag eLibrary
BWV eBooks
BWV Zeitschriften
Supreme Court Yearbook
CQ Almanac
CQ Global Researcher
CQ Magazine
CQ Supreme Court Collection
CQ Voting and Elections Collection
CAB Abstracts Summon Pilot
CAB Reviews Archive
Nutrition and Food Sciences Database
CABI Books Animal and Veterinary Science
CABI Descriptions of Fungi & Bacteria
CABI Distribution Maps of Plant Diseases
CABI Distribution Maps of Plant Pests
CABI eBook Archive
CABI eBook Current file
CABI eBooks Agriculture
CABI eBooks Environmental Sciences
CABI eBooks Human Health, Food & Nutrition
CABI eBooks Leisure & Tourism
CABI eBooks Plant Sciences
CABI Reviews

BACON - CAIRN - GLOBAL_COLLECTIFS-HUMANITES
BACON - CAIRN - GLOBAL_OUVRAGES-LETTRES-LINGUISTIQUE
BACON - CAIRN - GLOBAL_OUVRAGES-PHILOSOPHIE-RELIGIONS
BACON - CAIRN - GLOBAL_OUVRAGES-PSYCHANALYSE
BACON - CAIRN - GLOBAL_OUVRAGES-SANTEPUBLIQUE
BACON - CAIRN - GLOBAL_POCHES-LETTRES
Bacon CAIRN global collectif ecosocpol
Bacon CAIRN global ouvrages documentationhospitaliere
Bacon CAIRN global ouvrages ecosocpol
Bacon CAIRN global ouvrages general
Bacon CAIRN global ouvrages psychologiedelenfantetdeladolescent
Cairn eBooks Clinical Psychology
Cairn eBooks Education
CAIRN eBooks General
Cairn eBooks History, Geography
Cairn eBooks Humanities
Cairn eBooks Management
Cairn eBooks Political Science, Law
Cairn eBooks Psychology
Cairn eBooks Social Work
Cairn eBooks Sociology
Cairn Journal COUPERIN General
CAIRN Journals COUPERIN Psychology
CAIRN Journals Economy, Management, Finance
CAIRN Journals ECOSOCPOL
CAIRN Journals Education
CAIRN Journals General
Cairn Journals Geography and Environment
CAIRN Journals Healthcare
CAIRN Journals Humanities and Social Science
CAIRN Journals Psycho
CAIRN Journals Public health
CAIRN Journals Travail Social
Cairn Magazines Abridged
Cairn Magazines General
Cairn Open Access Journals
CAIRN Pocket Encyclopedias Economy, Management, Finance
CAIRN Pocket Encyclopedias General
CAIRN Pocket Encyclopedias History, Geography, Art
CAIRN Pocket Encyclopedias Philosophy, Religion

CAIRN Pocket Encyclopedias Political science, Law
CAIRN Pocket Encyclopedias Psychology
CAIRN Pocket Encyclopedias Que sais-je ?
CAIRN Pocket Encyclopedias Repères
CAIRN Pocket Encyclopedias Sociology, Ethnology, demography
CAIRN Pocket Encyclopedias Technical and Medical Sciences
Cairn Research General
Cairn Research Psychology
Cairn.info Encyclopédies de poche - Travail Social
Cairn.info Revues - Humanités
KB+ JISC Collections CAIRN Bouquet General Collection 2017-2019
KB+ JISC Collections CAIRN Economics Social And Political Sci Collection 2017-2019
KB+ JISC Collections CAIRN French Studies Collection 2017-2019
KB+ JISC Collections CAIRN Humanities And Social Sciences Collection 2017-2019
eScholarship Repository
Scholarly Commons
Apollo
Cambridge University Libguides
7titles Aletheia U
AletheaU7titles
Cambridge 2020 EBA for CollexPersee
Cambridge Books - Open Access
Cambridge Books Online - CollexPersee 2020 EBA
Cambridge Companions American Studies
Cambridge Companions Complete Collection
Cambridge Companions Online 2017 Collection
Cambridge Companions Online 2018 Collection
Cambridge Companions Online 2019 Collection
Cambridge Companions to Literature & Classics
Cambridge Companions to Music
Cambridge Companions to Philosophy, Religion and Culture
Cambridge Core All Books
Cambridge Core FID Politics 2016-2019
Cambridge Core Textbooks
Cambridge Core withdrawn books
Cambridge Core withdrawn journals
Cambridge EBA ebooks Complete Collection
Cambridge eBooks 2019 Science, Technology, and Medicine Collection, All eBooks
Cambridge eBooks 2020 Science, Technology, and Medicine Collection, All eBooks
Cambridge eBooks and Partner Presses All Books

Cambridge eBooks and Partner Presses All Books 2014
Cambridge eBooks and Partner Presses All Books 2016
Cambridge eBooks and Partner Presses All Books 2018
Cambridge eBooks and Partner Presses All Books 2019
Cambridge eBooks and Partner Presses All Books 2020
Cambridge eBooks and Partner Presses All Coursebooks
Cambridge eBooks and Partner Presses All Monographs
Cambridge eBooks and Partner Presses Anthropology 2017
Cambridge eBooks and Partner Presses Anthropology, All eBooks
Cambridge eBooks and Partner Presses Archaeology, All eBooks
Cambridge eBooks and Partner Presses Classical Studies 2017
Cambridge eBooks and Partner Presses Classical Studies, All eBooks
Cambridge eBooks and Partner Presses Computer Science 2017
Cambridge eBooks and Partner Presses Computer Science, All eBooks
Cambridge eBooks and Partner Presses Earth and Environmental Science, All eBooks
Cambridge eBooks and Partner Presses Economics, All eBooks
Cambridge eBooks and Partner Presses Engineering 2017
Cambridge eBooks and Partner Presses Engineering, All eBooks
Cambridge eBooks and Partner Presses Frontlist Collection 2015 to present
Cambridge eBooks and Partner Presses History 2017
Cambridge eBooks and Partner Presses Humanities 2017
Cambridge eBooks and Partner Presses Humanities and Social Sciences Collection, All eBooks
Cambridge eBooks and Partner Presses Humanities, All eBooks
Cambridge eBooks and Partner Presses Language and Linguistics 2017
Cambridge eBooks and Partner Presses Language and Linguistics, All eBooks
Cambridge eBooks and Partner Presses Law 2017
Cambridge eBooks and Partner Presses Law 2019
Cambridge eBooks and Partner Presses Law, All eBooks
Cambridge eBooks and Partner Presses Life Sciences 2017
Cambridge eBooks and Partner Presses Life Sciences, All eBooks
Cambridge eBooks and Partner Presses Literature 2017
Cambridge eBooks and Partner Presses Literature, All eBooks
Cambridge eBooks and Partner Presses Management 2017
Cambridge eBooks and Partner Presses Management, All eBooks
Cambridge eBooks and Partner Presses Mathematics 2017
Cambridge eBooks and Partner Presses Mathematics, All eBooks
Cambridge eBooks and Partner Presses Medicine 2017
Cambridge eBooks and Partner Presses Medicine, All eBooks
Cambridge eBooks and Partner Presses Music 2017
Cambridge eBooks and Partner Presses Music, All eBooks

Cambridge eBooks and Partner Presses Philosophy 2017
Cambridge eBooks and Partner Presses Philosophy, All eBooks
Cambridge eBooks and Partner Presses Physics and Astronomy 2017
Cambridge eBooks and Partner Presses Physics and Astronomy, All eBooks
Cambridge eBooks and Partner Presses Politics and International Relations 2017
Cambridge eBooks and Partner Presses Politics and International Relations, All eBooks
Cambridge eBooks and Partner Presses Psychology 2017
Cambridge eBooks and Partner Presses Psychology, All eBooks
Cambridge eBooks and Partner Presses Religion 2017
Cambridge eBooks and Partner Presses Religion, All eBooks
Cambridge eBooks and Partner Presses Science and Engineering 2017
Cambridge eBooks and Partner Presses Science and Engineering, All eBooks
Cambridge eBooks and Partner Presses Science, Technology, and Medicine Collection, All eBooks
Cambridge eBooks and Partner Presses Social Sciences 2017
Cambridge eBooks and Partner Presses Social Sciences, All eBooks
Cambridge eBooks and Partner Presses Sociology, All eBooks
Cambridge eBooks and Partner Presses Statistics and Probability 2017
Cambridge eBooks and Partner Presses Statistics and Probability, All eBooks
Cambridge eBooks Anthropology 2018
Cambridge eBooks Anthropology 2019
Cambridge eBooks Archaeology 2018
Cambridge eBooks Archaeology 2019
Cambridge eBooks Classical Studies 2018
Cambridge eBooks Classical Studies 2019
Cambridge eBooks Computer Science 2018
Cambridge eBooks Computer Science 2019
Cambridge eBooks Earth and Environmental Science 2018
Cambridge eBooks Earth and Environmental Science 2019
Cambridge eBooks Economics 2018
Cambridge eBooks Economics 2019
Cambridge eBooks Engineering 2018
Cambridge eBooks Frontlist 2019
Cambridge eBooks Frontlist 2019, excluding Law-Medicine
Cambridge eBooks Frontlist 2020
Cambridge eBooks History 2018
Cambridge eBooks History 2019
Cambridge eBooks Humanities 2018
Cambridge eBooks Humanities 2019
Cambridge eBooks Language and Linguistics 2018
Cambridge eBooks Language and Linguistics 2019

Cambridge eBooks Law 2018
Cambridge eBooks Law 2019
Cambridge eBooks Life Sciences 2018
Cambridge eBooks Life Sciences 2019
Cambridge eBooks Literature 2018
Cambridge eBooks Literature 2019
Cambridge eBooks Management 2018
Cambridge eBooks Management 2019
Cambridge eBooks Mathematics 2018
Cambridge eBooks Mathematics 2019
Cambridge eBooks Medicine 2018
Cambridge eBooks Medicine 2019
Cambridge eBooks Music 2018
Cambridge eBooks Music 2019
Cambridge eBooks Philosophy 2018
Cambridge eBooks Philosophy 2019
Cambridge eBooks Physics and Astronomy 2018
Cambridge eBooks Physics and Astronomy 2019
Cambridge eBooks Politics and International Relations 2018
Cambridge eBooks Politics and International Relations 2019
Cambridge eBooks Psychology 2018
Cambridge eBooks Psychology 2019
Cambridge eBooks Religion 2018
Cambridge eBooks Religion 2019
Cambridge eBooks Science and Engineering 2018
Cambridge eBooks Science and Engineering 2019
Cambridge eBooks Social Sciences 2018
Cambridge eBooks Social Sciences 2019
Cambridge eBooks Statistics and Probability 2018
Cambridge eBooks Statistics and Probability 2019
Cambridge Edition of the Works of Ben Jonson Online
Cambridge Elements 2018
Cambridge Handbooks Language and Linguistics, All eBooks
Cambridge Handbooks Psychology
Cambridge Handbooks, All eBooks
Cambridge Histories 2013
Cambridge Histories 2017
Cambridge Histories 2018
Cambridge Histories Online
Cambridge Histories Online 2012 and Preceeding

Cambridge Histories Online Archive to 2016
Cambridge Histories Online Complete
Cambridge JISC EBA ebooks Complete Collection
Cambridge Jnls 2017 CAUL Full Pckg Excl AAP Titles
Cambridge Journals 2016 Agriculture Package Standard UK
Cambridge Journals 2016 Biological Sci Package Standard UK
Cambridge Journals 2016 Business Package UK
Cambridge Journals 2017 CJDA Complete HSS Package
Cambridge Journals 2017 CJDA Complete Package
Cambridge Journals 2017 HSS Package Standard UK
Cambridge Journals 2017 N American Full Package
Cambridge Journals 2017 North American Full Package
Cambridge Journals 2017 SC-Tec Package Standard UK
Cambridge Journals 2017 STM Package Standard UK
Cambridge Journals 2018 BTAA - CJDA Select Subject Packages
Cambridge Journals 2018 CAUL Full package excluding AAP titles
Cambridge Journals 2018 CJDA Full Collection UK
Cambridge Journals 2018 HSS Package Standard UK
Cambridge Journals 2018 MedVet Package Standard UK
Cambridge Journals 2018 STM Package Standard UK
Cambridge Journals 2019 AAP Titles
Cambridge Journals 2019 CJDA Complete HSS Package Americas
Cambridge Journals 2019 CJDA Complete Package Americas
Cambridge Journals 2019 CJDA Complete STM Package Americas
Cambridge Journals 2019 CJDA Full Package UK
Cambridge Journals 2019 Full Package Standard Ams
Cambridge Journals 2019 Full Package Standard UK 19FULL1UK
Cambridge Journals 2019 HSS Package Standard Ams
Cambridge Journals 2019 HSS Package Standard UK 19HSS1UK
Cambridge Journals 2019 MedVet Package Standard UK 19MED1UK
Cambridge Journals 2019 OhioLINK Package
Cambridge Journals 2019 SANLiC 2017STATIC2019
Cambridge Journals 2019 STM Package Standard Ams
Cambridge Journals 2019 STM Package Standard UK 19STM1UK
Cambridge Journals 2020 Full Package
Cambridge Journals CAUL 2017 AAP Titles
Cambridge Journals CJDA Full Package UK 2019
Cambridge Journals Full Package Standard UK 2019
Cambridge Journals Online Archives (Eira)
Cambridge Library Collection Online

Cambridge Materials Research Society Journals
Cambridge University Press Journals Complete
Cambridge University Press Journals Digital Archive
Cambridge University Press Journals Digital Archive HSS
Cambridge University Press Journals Digital Archive STM
Cambridge University Press Journals Full Package Standard North America
Cambridge University Press Journals HSS
Cambridge University Press Journals STM
Cambridge University Press online books
Cambridge University Press Shakespeare Survey Online
Cambridge University Press Wholly Gold Open Access Journals
Complete Cambridge Elements
CRKN Cambridge University Press Journals
Dictionary of Irish Biography
DRAA Cambridge Journals Online-剑桥期刊库
JISC CJDA 2009 Part 1 HSS Collection
JISC CJDA 2009 STM Collection
JUSTICE Cambridge University Press Journals 2017
JUSTICE Cambridge University Press Journals HSS 2017
JUSTICE Cambridge University Press Journals STM 2017
KB+ BIBSAM Cambridge University Press Journals Online 2015-2017
KB+ Bibsam Cambridge University Press Journals Online 2018
KB+ Cambridge University Press: JISC Collections:Full Collection Digital Archives (STM and HSS)
KB+ JISC Collections Cambridge University Press full Collection 2015
KB+ JISC Collections Cambridge University Press Full Collection 2016
KB+ JISC Collections Cambridge University Press Full Collection 2017
KB+ JISC Collections Cambridge University Press Full Collection 2018
KB+ JISC Collections Cambridge University Press humanities And Social Sciences 2015
KB+ JISC Collections Cambridge University Press humanities And Social Sciences 2017
KB+ JISC Collections Cambridge University Press science Technology And Medicine 2015
KB+ JISC Collections Cambridge University Press science Technology And Medicine 2017
KB+ JISC Collections Cambridge University Press Science, Technology And Medicine Collection 2016
KB+ JISC Collections Cambridge University Press Science, Technology And Medicine Digital Archives
KB+ JISC Collections Cambridge University Press Social Science And Humanities Collection 2016
KB+ SHEDL Cambridge University Press Full Collection 2017
KB+ SHEDL Cambridge University Press: Full Collection 2017
NCL Cambridge Journals Digital Archive 2011
Nesli2 Cambridge University Press Journals
SHEDL Cambridge University Press Journals
Stahl's Essential Psychopharmacology Online

Canadian Aeronautics and Space Institute Publications
Canadian Anesthesiologists' Society
desLibris - Books
desLibris - CRKN purchase 2008
desLibris - Documents
desLibris - Guides de voyage Ulysse / Ulysses Travel Guides
desLibris - Irwin Law
Canadian Journal of Community Mental Health
Canadian Mathematical Society
Canadiana Online - Monographs
CRKN Open Access Journals List
NRC Research Press Journals
Canisius College LibGuides
Capella University LibGuides
DigiBib.Jeugd
Foreign Policy
AMICA Library Art Museum Images
David Rumsey Historical Map Collection
Farber Gravestone Collection
Japanese Historical Maps
National Palace Museum - Taipei
Casalini EEO Edición Española Online
Casalini EIO Italian Studies
Il Mulino eJournals
CASPUR - CIBER Publishing
International Journal of Dental Clinics
Cell Press
Cell Press Free Archives
KB+ JISC Collections Cell Press 2017-2019
CEDRAM Journals
Charleston Advisor
CRL Catalog
Centers for Disease Control and Prevention
Centers for Disease Control Web site
CEEOL Open Access Grey Literature
CEEOL: Open Access
Central and Eastern European Online Library - CEEOL Journals
Central and Eastern European Online Library - CEEOL Open Access Ebooks
Central Eastern European Online Library CEEOL Ebooks
Central Ornithology Publication Office

Centre for Analysis of Social Exclusion (CASE) Publications
CEPR Discussion Papers Online
BibCNRS
CFA Institute Publications
Black Studies Center
Chadwyck Black Studies Center Dissertations
Chadwyck Patrologia Latina
Gerritsen Collection
Historical Newspapers Online
International Index to Black Periodicals (IIBP)
Keris PAO Collection
MLA International Bibliography
Schomburg Studies on the Black Experience
Acta Sanctorum
Annual Bibliography of English Language and Literature
Archive Finder
ArchivesUSA
Black Abolitionist Papers
Black Studies Center: Black Literature Index
C19: Nineteenth Century Index
C19: Nineteenth Century Index (UK)
English Verse Drama
History Study Center: All Content
History Study Center: Historical Documents
History Study Center: Journals
History Study Center: Journals - International Clients
History Study Center: Maps & Reference
History Study Center: Multimedia
John Johnson Collection
KnowledgeNotes - UK Subscription
KnowledgeNotes - US Subscription
Literary Theory
Literature Online (LION) - Biographies
Luthers Werke
Paley Center Seminars
Patrologia Latina Database (via UK)
Queen Victoria’s Journals - Open Access version for UK and selected Commonwealth countries
Queen Victoria's Journals
Chalmers Publication Library (CPL)
Chandler-Gilbert Community College FoD

China Academic Conference Proceedings Database (Outside Mainland China Only)
China Online Journals Agriculture
China Online Journals Business Economics And Finance
China Online Journals Complete
China Online Journals Education Literature Art
China Online Journals Fundamental Sciences
China Online Journals Industrial Technology
China Online Journals Medicine And Health Sciences
China Online Journals Philosophy Politics Law
China Online Journals Social Sciences
China/Asia On Demand
LawInfoChina
北大法宝-法律法规检索系统
Chongqing University Open Access Journals
Chronicle of Higher Education
World Factbook
ciando eBooks
CiNii Complete
CityU Academic Repository
Bayle Corpus--Œuvres complètes
Bayle Corpus--oeuvres complètes
Bibliothèque de la Renaissance
Bibliothèque des Lettres
Classiques de l'argot et du jargon
Classiques Garnier Online
Classiques Jaunes en ligne
Colloques, congrès et conférences sur la Renaissance européenne
Corpus de la littérature médiévale des origines au 15e siècle
Corpus de la littérature narrative
Corpus de la première littérature francophone de l'Océan Indien, écrite et orale, des origines aux Indépendances
Corpus des dictionnaires de l'Académie française
Critique littéraire
Dictionnaire historique de l''ancien langage français depuis son origine jusqu''au siècle de Louis XIV
Dictionnaires des XVIe et XVIIe siècles
Dictionnaires des XVIe et XVIIe siècles
Dictionnaires et synthèses
Écrits sur l'art
Edmond Huguet, Dictionnaire de la langue française du 16e siècle
Émile Littré: Le Dictionnaire de la langue française
Encyclopédie Diderot et D'Alembert

Encyclopédie d'Yverdon
Encyclopédie d''Yverdon
Études de littérature des XXe et XXIe siècles
Études et essais sur la Renaissance
Études montaignistes
Études romantiques et dix-neuviémistes
Grand Corpus des dictionnaires
Grand Corpus des grammaires françaises des remarques et des traités
La Curne. Dictionnaire de l'ancien français
La Révolution et l'Empire
Larousse, Grand Dictionnaire universel du 19e s.
Littérature de l'Afrique noire
Masculin/féminin dans l'Europe moderne
Patrologia græca
Perspectives comparatistes
Recherches littéraires médiévales
Rencontres
Savoirs anciens et médiévaux
Textes de la Renaissance
Textes littéraires du Moyen Âge
Théorie de la littérature
OpenEdition Freemium
Clinical and Experimental Rheumatology (Online)
CLOCKSS
Co-Action Open Access Journals
CogPrints
Coimbra University Press Impactum Journals Free
Coimbra University Press Pombalina Books
Cold Spring Harbor Protocols
Archivo Digital
College Art Association (CAA) Reviews
Special Collections Research Center (SCRC) Database
Columbia International Affairs Online (CIAO)
Columbia International Affairs Online Books
Electronic Journals and Newspapers on Africa
Combined Arms Research Library Digital Library
Communication Institute for Online Scholarship
COS Funding Opportunities
COS Papers Invited
Compact Memory

COMPLIANCEDigital
Computing Reviews
Conseil International de la Langue Française
Ciencia y Tecnologia (ICYT)
Ciencias Sociales y Humanidades (ISOC)
Revistas Cientificas Del CSIC Free Journals
SINERGIE
Copernicus Publications
arXiv Computer Science
arXiv Mathematics
arXiv Nonlinear Science
arXiv Physics
arXiv Quantitative Biology
arXiv Statistics
arXiv.org
Core Historical Literature of Agriculture
Home Economics Journals
Corpus Montaigne
Council for Exceptional Children
CODESRIA Department of Publications and Dissemination
Council on Foreign Relations Publications
CQ Electronic Library
CQ Press Electronic Library
CQ Researcher Plus Archive
CQVIP
Advertising Age and Data Center
Cranfield Collection of E-Research - CERES
CRC Handbook of Chemistry and Physics
AGRICULTUREnetBASE
BIOMEDICALSCIENCEnetBASE
BIOSCIENCEnetBASE
BUSINESSnetBASE/MANAGEMENTnetBASE
CHEMICALENGINEERINGnetBASE
CHEMLIBnetBASE
CivilEngineeringnetBASE
COMPUTERSCIENCEnetBASE
CRC TELECOMMUNICATIONSnetBASE
CRCnetBASE Complete
ECONOMICSnetBASE
ElectricalEngineeringnetBASE

ENERGYANDCLEANTECHnetBASE
ENGnetBASE
ENVIROnetBASE
ENVIRONMENTALENGINEERINGnetBASE
ENVIRONMENTALSCIENCEnetBASE
ERGONOMICSnetBASE
FOODnetBASE
FORENSICnetBASE
GeneralENGINEERINGnetBASE
GEOnetBASE
HEALTHCAREnetBASE
HOMELANDSECURITYnetBASE
IndustrialENGINEERINGnetBASE
InfoSECURITYnetBASE
ITECHnetBASE
ITknowlegeBASE
LIFESCIENCEnetBASE
MATERIALSnetBASE
MATHnetBASE
MechanicalEngineeringnetBASE
MEDICINEnetBASE
MILITARYnetBASE
MiningENGINEERINGnetBASE
NANOnetBASE
NEUROSCIENCEnetBASE
NUTRITIONnetBASE
OCCUP-HEALTHandSAFETYnetBASE
PHARMACEUTICALnetBASE
PHYSICSnetBASE
POLYMERSnetBASE
PROJECTMANAGEMENTnetBASE
PublicADMINISTRATIONnetBASE
SCI-TECHnetBASE
STATSnetBASE
TAEBCnetBASE
TEXTILEnetBASE
VETnetBASE
WATERnetBASE
Credo Reference
Credo Reference: Social Sciences

Credo United States History Collection v4
CREDOreference Academic Core
CREDOreference All Content
CrossRef
Crystallography Journals
CSIRO Journals
JCPML Electronic Research Archives
Bibliovox Arts et Lettres
Bibliovox Economie Entreprise
Bibliovox Emploi Métiers Formation
Bibliovox Enfance Jeunesse
Bibliovox les Classiques
Bibliovox Libertinage et érotisme
Bibliovox Littérature Afrique Caraïbes
Bibliovox Livres en Anglais
Bibliovox Livres en espagnol
Bibliovox Sciences
Bibliovox Tourisme et Voyages
Bibliovox vie Pratique
International Action Sociale et Médico-Sociale
International Emploi, Métiers et Formation
International Gestion et Sciences économiques
International Pédagogie et Enseignement
International Santé Sciences de la Vie et Médecine
International Sciences Humaines et Sociales
International Sciences informatique et techniques
ScholarVox Management
ScholarVox U-BORDEAUX
ScholarVox U-BORDEAUX Emploi, métiers et formation
ScholarVox U-BORDEAUX Informatique
ScholarVox U-BORDEAUX Lectures loisirs
ScholarVox U-BORDEAUX Lettre
ScholarVox U-BORDEAUX Santé, Sciences de la Vie et Médecine
ScholarVox U-BORDEAUX Sciences de l'Éducation ESPE
ScholarVox U-BORDEAUX Sciences de l'ingénieur
ScholarVox U-BORDEAUX Sciences Economiques et de Gestion
ScholarVox U-BORDEAUX Sciences Humaines et Sociales
ScholarVox U-BORDEAUX Sciences Politiques
ScholarVox U-BORDEAUX STAPS
Scholarvox Université ESPE - Couperin

ScholarVox Université Informatique
ScholarVox Université Lectures loisirs
ScholarVox Université Lettres
ScholarVox Université Livres en espagnol
Scholarvox Université métiers - Couperin
Scholarvox Université Santé - Couperin
Scholarvox Université Science Humaines et Sociales - Couperin
ScholarVox Université Sciences - Couperin
ScholarVox Université Sciences Eco Gestion - Couperin
ScholarVox Université Sciences Politiques
ScholarVox Université STAPS
ScholarVox UNR-RA
Daat - Herzog College - דעת - מכון הרצוג
Dalloz Bibliothèque
Dana Press
Dankook Univeristy dCollection
Dankook Univeristy eBook
Darwinbooks
Data Trace E-Journals
DataCite
DataCite (Open Access)
African Studies Abstracts
Scientific American Surgery
DEEDI eReseach Archive (eRA)
FIS Bildung Literaturdatenbank
Dialnet
Digital Commons Online Journals
Digital Library of Georgia
Digital Repository @ Iowa State University Open Access
Orthotics & Prosthetics Virtual Library
Digitale Sammlungen
Digitale Sammlungen (Universitätsbibliothek Paderborn)
Collection Ocho y Medio of Cinema Studies
Digitalia eJournals
Digitalia Hispanica
Anthropos
DigitalNZ
Recercat
Disaster Recovery Journal
Indianjournals.com

DOAB Directory of Open Access Books
Doab Directory Open Access Books And Chapters Free
DOAJ Directory of Open Access Journals
Docuseek2 Complete Collection
Docuseek2 Essential Collection
Docuseek2 International Collection
Global Environmental Justice Collection
Dokumentenserver der FU Berlin
Thomist
Dove Press Free
Factiva Major News and Business Publications (Corporate)
Habelt Zeitschriften Online
Drexel Library Catalog
Drexel University LibGuides
iDEA: Drexel Libraries E-Repository and Archives
DTIC STINET
DTIC Technical Reports
DORAS
Duke Law Scholarship Repository
DukeSpace
DMJ100
Duke University Press
Duke University School of Law Journals
e-Duke All Books
e-Duke Books African American, African, and Black Diaspora Studies Collection Collection 2019
e-Duke Books African American, African, and Black Diaspora Studies Collection Collection 2020
e-Duke Books Anthropology Collection 2019
e-Duke Books Anthropology Collection 2020
e-Duke Books Art and Art History Collection 2018
e-Duke Books Art and Art History Collection 2019
e-Duke Books Art and Art History Collection 2020
e-Duke Books Asian Studies Collection 2018
e-Duke Books Asian Studies Collection 2019
e-Duke Books Asian Studies Collection 2020
e-Duke Books Gender Studies Collection 2016
e-Duke Books Gender Studies Collection 2017
e-Duke Books Gender Studies Collection 2018
e-Duke Books Gender Studies Collection 2019
e-Duke Books Gender Studies Collection 2020
e-Duke Books Latin American History Collection 2018

e-Duke Books Latin American History Collection 2019
e-Duke Books Latin American History Collection 2020
e-Duke Books Latin American Studies Collection 2016
e-Duke Books Latin American Studies Collection 2017
e-Duke Books Music and Sound Studies Collection 2017
e-Duke Books Music and Sound Studies Collection 2018
e-Duke Books Music and Sound Studies Collection 2019
e-Duke Books Music and Sound Studies Collection 2020
e-Duke Books Religious Studies Collection 2017
e-Duke Books Religious Studies Collection 2018
e-Duke Books Religious Studies Collection 2019
e-Duke Books Religious Studies Collection 2020
eDuke Books scholarly collection 2008
eDuke Books scholarly collection 2009
eDuke Books scholarly collection 2010
eDuke Books scholarly collection 2011
eDuke Books scholarly collection 2012
e-Duke Books Scholarly Collection 2013
e-Duke Books Scholarly Collection 2014
e-Duke Books Scholarly Collection 2015
e-Duke Books Scholarly Collection 2016
e-Duke Books Scholarly Collection 2017
e-Duke Books Scholarly Collection 2018
e-Duke Books Scholarly Collection 2019
eDuke Books scholarly collection 2020
eDuke Books scholarly collection Pre-2008 Archive
e-Duke Journals Scholarly Collection: Standard
e-Duke Journals Scholarly Collections: Expanded 2020
e-Duke Journals Scholarly Collections: Standard 2020
KB+ JISC Collections Duke University Press eBooks 2017 Scholarly Collection
KB+ JISC Collections Duke University Press eBooks Gender Studies 2017 Collection
KB+ JISC Collections Duke University Press eBooks Latin American Studies 2017 Collection
KB+ JISC Collections Duke University Press Expanded Collection 2015
KB+ JISC Collections Duke University Press Scholarly Collection 2017
KB+ Jisc Collections Duke University Press Scholarly Collection 2018
KB+ JISC Collections Duke University Press Scholarly Collection 2019
Dumas (Dépôt Universitaire de Mémoires Après Soutenance)
Duncker & Humblot eLibrary Books
Duncker & Humblot eLibrary eJournals
Dustbooks

Dustri-Verlag Online Publications
Earthquake Engineering Research Institute Publications
e-articles
Joyner Library Digital Collections
Complete List of Russian, Ukrainian, CIS and Baltic Titles
East View Russia Governmental Publications (UDB-GOV)
East View Russia Medicine and Public Health (UDB-MED)
East View Russia Voprosy istorii Complete Collection 1926-2004
East View Russian Social Sciences and Humanities Journals (UDB-EDU)
East View Universal Database of the Moscow University Herald
East View World News Connection
Literaturnaia Gazeta
Markov Processes and Related Fields
Nauka Online
Niva Digital Archive
TKN East View Century Journals Project Agriculture
TKN East View Century Journals Project Architecture, Energy, Traffic, Electromechanics
TKN East View Century Journals Project Chemistry, Metallurgy, Environment, Mine Industry
TKN East View Century Journals Project Complete
TKN East View Century Journals Project Economics & Management
TKN East View Century Journals Project Education & Social Sciences
TKN East View Century Journals Project Electronic Technology & Information Science
TKN East View Century Journals Project Literature, History, Philosophy
TKN East View Century Journals Project Mathematics, Physics, Mechanics, Astronomy
TKN East View Century Journals Project Medicine & Public Health
TKN East View Century Journals Project Politics, Military Affairs, Law
TKN East View Century Journals Project. Phase 1
TKN East View China Academic Journals (California Platform) Agriculture
TKN East View China Academic Journals (California Platform) Architecture, Energy, Traffic, Electromechanics, Etc
TKN East View China Academic Journals (California Platform) Chemistry, Metallurgy, Environment, Mine Industry
TKN East View China Academic Journals (California Platform) Economy & Management
TKN East View China Academic Journals (California Platform) Education & Social Sciences
TKN East View China Academic Journals (California Platform) Electronic Technology & Information Science
TKN East View China Academic Journals (California Platform) Literature, History, Philosophy
TKN East View China Academic Journals (California Platform) Mathematics, Physics, Mechanics, and Astronomy
TKN East View China Academic Journals (California Platform) Medicine & Public Health
TKN East View China Academic Journals (California Platform) Politics, Military Affairs, Law
TKN East View China Academic Journals Agriculture
TKN East View China Academic Journals Chemistry, Metallurgy
TKN East View China Academic Journals Complete

TKN East View China Academic Journals Complete (California Platform)
TKN East View China Academic Journals Economics & Management
TKN East View China Academic Journals Economics, Politics, Law
TKN East View China Academic Journals Education, Social Sciences
TKN East View China Academic Journals Electronics, Information Science
TKN East View China Academic Journals Industrial Technology & Engineering
TKN East View China Academic Journals Literature, History, Philosophy
TKN East View China Academic Journals Medicine, Health
TKN East View China Academic Journals Physics, Astronomy, Mathematics
Current Digest of Post-Soviet Press
Nauka Online
Pravda Digital Archive
Universal Database of Russian Leading Newspapers (Public Library Edition)
Universal Database of Russian Library and Information Sciences Publications
UDB-NN: AAASS NewsNet Archive
East View Press
Ebook Central Academic Complete
Ebook Central Academic Complete ANZ Edition
Ebook Central Academic Complete International Edition
Ebook Central Academic Complete UKI Edition
Ebook Central Academic Complete, China Edition
Ebook Central Academic Complete, Middle East Edition
Ebook Central Aerospace
Ebook Central Boydell & Brewer EBA Front List 2020
Ebook Central Business
Ebook Central Business Complete
Ebook Central Business Project Management
Ebook Central Canada Ebook Subscription / Collection Canadienne Globale
Ebook Central Chemical Engineering
Ebook Central Civil Engineering
Ebook Central College Complete
Ebook Central Computing
Ebook Central Corporate Engineering
Ebook Central Custom SME (Society of Manufacturing Engineers) Source™
Ebook Central Deutsche Sammlung
Ebook Central Education
Ebook Central Electronics Engineering
Ebook Central Employee Development
Ebook Central Engineering & Industrial Management
Ebook Central Engineering Core

Ebook Central Engineering Select
Ebook Central Engineering Systems
Ebook Central Environmental Engineering
Ebook Central Finance
Ebook Central Food Science Engineering
Ebook Central Health & Medicine
Ebook Central History
Ebook Central Human Resources
Ebook Central IGI Global InfoSci Books
Ebook Central IGI Global InfoSci Journals
Ebook Central Law
Ebook Central Leadership
Ebook Central Literature & Language
Ebook Central Materials Engineering
Ebook Central Mechanical Engineering
Ebook Central Medical Technology
Ebook Central Nordic
Ebook Central PACKT EBA Front List 2020
Ebook Central PALCI/SAGE EBA
Ebook Central Perpetual, DDA and Subscription Titles
Ebook Central Petroleum Engineering
Ebook Central Pharmaceuticals
Ebook Central Power Engineering
Ebook Central Pre 2015: Business & Economics
Ebook Central Pre 2015: Computers
Ebook Central Pre 2015: Education
Ebook Central Pre 2015: Engineering
Ebook Central Pre 2015: History & Political Sciences
Ebook Central Pre 2015: Humanities
Ebook Central Pre 2015: Interdisciplinary & Area Studies
Ebook Central Pre 2015: Language & Literature
Ebook Central Pre 2015: Law
Ebook Central Pre 2015: Life Sciences
Ebook Central Pre 2015: Medical
Ebook Central Pre 2015: Nursing & Allied Health
Ebook Central Pre 2015: Physical Sciences
Ebook Central Pre 2015: Psychology & Social Work
Ebook Central Pre 2015: Religion & Philosophy
Ebook Central Pre 2015: Sociology & Anthropology
Ebook Central Public Library Complete

Ebook Central Reference
Ebook Central Reference Research
Ebook Central Religion
Ebook Central Religion & Philosophy
Ebook Central Rowman & Littlefield EBA 2020
Ebook Central Sales & Marketing
Ebook Central Schools & Educators Complete
Ebook Central Science & Technology
Ebook Central Social Sciences
Ebook Central Spanish Language
Ebook Central Suncor Custom
Ebook Central SUNY EBA Front List 2020
Ebook Central Taylor & Francis EBA 10 Year Collection
Ebook Central Taylor & Francis EBA 20 Year Collection
Ebook Central The Arts
Ebook Central University Press
eLibro via Ebook Central Colección Español Completa - Spanish Complete
eLibro via Ebook Central Escuelas Secundarias - High School
Elibro via Ebook Central Premium
Poetry & Short Story Reference Center
Architectural Digest Magazine Archive
Bibliography of Asian Studies
Bloomberg Businessweek Digital Archive
British Nursing Index
Business Source Main Edition
Consumer Reports
eBook Business Collection
eBook EngineeringCore Collection
eBook Religion Collection
EBSCOhost Academic eBook Collection (North America)
EBSCOhost Academic Search Alumni Edition
EBSCOhost Academic Search Complete
EBSCOhost Academic Search Elite
EBSCOhost Academic Search Premier
EBSCOhost Academic Search Ultimate
EBSCOhost Advanced Placement Source
EBSCOhost African American Historical Serials
EBSCOhost Alt HealthWatch
EBSCOhost America History and Life with Full Text
EBSCOhost American Antiquarian Society Historical Periodicals Collection: Series 1

EBSCOhost American Antiquarian Society Historical Periodicals Collection: Series 2
EBSCOhost American Antiquarian Society Historical Periodicals Collection: Series 3
EBSCOhost American Antiquarian Society Historical Periodicals Collection: Series 4
EBSCOhost American Antiquarian Society Historical Periodicals Series: 5
EBSCOhost APA PsycARTICLES
EBSCOhost APA PsycBooks
EBSCOhost Applied Science & Technology Full Text
EBSCOhost Applied Science & Technology Source
EBSCOhost Applied Science & Technology Source Ultimate
EBSCOhost Art & Architecture Complete
EBSCOhost Art & Architecture Source
EBSCOhost Art Full Text
EBSCOhost Associates Programs Source
EBSCOhost Associates Programs Source Plus
EBSCOhost Atla Religion Database with AtlaSerials PLUS [LSDAR]
EBSCOhost AtlaSerials, Religion Collection [A6H]
EBSCOhost Australia New Zealand Reference Collection
EBSCOhost Biological and Agricultural Index Plus
EBSCOhost BioMedical Collection Corporate
EBSCOhost Biomedical Reference Collection: Basic
EBSCOhost Biomedical Reference Collection: Comprehensive
EBSCOhost Biomedical Reference Collection: Expanded
EBSCOhost Business Abstracts with Full Text
EBSCOhost Business Continuity & Disaster Recovery Reference Center
EBSCOhost Business Source Alumni Edition
EBSCOhost Business Source Complete
EBSCOhost Business Source Corporate
EBSCOhost Business Source Corporate Plus
EBSCOhost Business Source Elite
EBSCOhost Business Source Premier
EBSCOhost Business Source Ultimate
EBSCOhost Central & Eastern European Academic Source
EBSCOhost CINAHL
EBSCOhost CINAHL Complete
EBSCOhost CINAHL Plus
EBSCOhost CINAHL Plus with Full Text
EBSCOhost CINAHL with Full Text
EBSCOhost Communication & Mass Media Complete
EBSCOhost Communication Source
EBSCOhost Computer Source

EBSCOhost Computers and Applied Sciences Complete
EBSCOhost Corporate ResourceNet (CRN)
EBSCOhost Criminal Justice Abstracts with Full Text
EBSCOhost Dentistry & Oral Sciences Source
EBSCOhost Ebook Academic Collection - World Wide
EBSCOhost eBook Collection
EBSCOhost eBook Community College Collection
EBSCOhost eBook History Collection - North America
EBSCOhost eBook Nursing Collection
Ebscohost Ebooks Comprehensive Academic Collection (North America)
Ebscohost Ebooks University Press Collection (North America)
Ebscohost Ebooks University Press Collection Worldwide
EBSCOhost Econlit with Full Text
EBSCOhost Education Full Text
EBSCOhost Education Research Complete
EBSCOhost Education Source
EBSCOhost Electronic Journals Service
EBSCOhost Energy & Power Source
EBSCOhost Engineering Source
EBSCOhost Entrepreneurial Studies Source
EBSCOhost Environment Complete
EBSCOhost Film and Television Literature Index with Full Text
EBSCOhost Food Science Source
EBSCOhost French Business Source
EBSCOhost Fuente Academica
EBSCOhost Fuente Académica Premier
EBSCOhost General Science Full Text
EBSCOhost GreenFile
EBSCOhost Health Business FullTEXT
EBSCOhost Health Business FullTEXT Elite
EBSCOhost Health Policy Reference Center
EBSCOhost Health Source Nursing Academic Edition
EBSCOhost Historical Abstracts with Full Text
EBSCOhost Hospitality & Tourism Complete
EBSCOhost Humanities Full Text
EBSCOhost Humanities International Complete
EBSCOhost Humanities Source
EBSCOhost Humanities Source Ultimate
EBSCOhost Index to Legal Periodicals & Books Full Text
EBSCOhost International Security & Counter Terrorism Reference Center

EBSCOhost Legal Collection
EBSCOhost Legal Source
EBSCOhost LGBT Life Plus Full Text
EBSCOhost Library & Information Science Source
EBSCOhost Library Literature & Information Science Full Text
EBSCOhost Library, Information Science & Technology Abstracts with full text(LISTA)
EBSCOhost MasterFILE Elite
EBSCOhost MedicLatina
EBSCOhost MEDLINE Complete
EBSCOhost MEDLINE with Full Text
EBSCOhost MegaFILE
EBSCOhost Military and Government Collection
EBSCOhost National Review Archive
EBSCOhost Newspaper Source Plus
EBSCOhost Nursing and Allied Health: Basic
EBSCOhost Nursing and Allied Health: Comprehensive
EBSCOhost Nursing and Allied Health: Expanded
EBSCOhost OmniFile Full Text Mega
EBSCOhost OmniFile Full Text Select
EBSCOhost Philosopher’s Index with Full Text
EBSCOhost Political Science Complete
EBSCOhost Professional Development Collection
EBSCOhost Reader's Guide Full Text Mega
EBSCOhost Readers' Guide Full Text Select
EBSCOhost Regional Business News
EBSCOhost Rehabilitation & Sports Medicine Source
EBSCOhost Religion and Philosophy Collection
EBSCOhost Risk Management Reference Center
EBSCOhost Science & Technology Collection
EBSCOhost Small Business Reference Center
EBSCOhost Social Sciences Full Text
EBSCOhost SocINDEX with Full Text
EBSCOhost Sociological Collection
EBSCOhost Sociology Source Ultimate
EBSCOhost SPORTDiscus with Full Text
EBSCOhost STM Source
EBSCOhost Sustainability Reference Center
EBSCOhost Textile Technology Complete
EBSCOhost The Nation Archives
EBSCOhost The New Republic Archive

EBSCOhost The New Scientist Archive
EBSCOhost UK/Eire Reference Centre
EBSCOhost Vocational & Career Collection
EBSCOhost Vocational Studies Complete
EBSCOhost Vocational Studies Premier
ERIC
Fonte Academica
International Bibliography of the Social Sciences
Leadership & Management Learning Center
MEDLINE
Nation Archive Premium Edition
Newspaper Source
Nonprofit Organization Reference Center
RILM Abstracts of Music Literature with Full Text
Science Full Text Select (H.W. Wilson)
Shock & Vibration Digest
Sustainability Watch
Thematic Collection from AAS: Women's Periodicals of the 18th and 19th Century
Time Magazine Archive
Espace ÉTS (École de technologie supérieure)
Ecological Society of America Publications
Economic Commission for Latin America and the Caribbean (CEPAL) - Publications
Economist Intelligence Unit
Economist Newspaper
Ediciones Universidad de Salamanca Revistas Científicas
Edinburgh University Press
EUP Premium Subscription List
KB+ Jisc Collections Edinburgh University Press Complete Collection 2018
KB+ JISC Collections Edinburgh University Press Complete Collection 2019
KB+ SHEDL Edinburgh University Press Complete Collection 2015
KB+ SHEDL Edinburgh University Press Journals 2016-2019
Bibliothèque Numérique ENI
Editions ENI - vidéos
Mementis
Navis
Editorial Medica Panamericana
Bioflux Journals
Edizioni Minerva Medica
EDP Open
EDP Sciences

EDP Sciences Journal de Physique Archives
KB+ JISC Collections EDP Sciences 2016
KB+ JISC Collections EDP Sciences Astronomy 2016
KB+ JISC Collections EDP Sciences Engineering & Technology 2016
KB+ JISC Collections EDP Sciences Life Sciences 2016
KB+ JISC Collections EDP Sciences Mathematics & Computer Sciences 2016
KB+ JISC Collections EDP Sciences Physics 2016
Education Resources Information Center (ERIC)
Education Week
Edward Elgar Books
Edward Elgar Business 2010 and before
Edward Elgar Business 2011
Edward Elgar Business 2012
Edward Elgar Business 2013
Edward Elgar Business 2014
Edward Elgar Business 2015
Edward Elgar Business 2016
Edward Elgar Business 2017
Edward Elgar Business 2018
Edward Elgar Business 2020
Edward Elgar Business and Management 2011
Edward Elgar Business and Management Handbook Subscription 2014
Edward Elgar Business and Management Handbook Subscription 2015
Edward Elgar Business and Management Handbook Subscription 2016
Edward Elgar Business and Management Handbook Subscription 2017
Edward Elgar Business and Management Handbook Subscription 2018
Edward Elgar Economics 2010 and earlier
Edward Elgar Economics 2011
Edward Elgar Economics 2012
Edward Elgar Economics 2013
Edward Elgar Economics 2014
Edward Elgar Economics 2015
Edward Elgar Economics 2016
Edward Elgar Economics 2017
Edward Elgar Economics 2018
Edward Elgar Economics 2020
Edward Elgar Law 2010 and earlier
Edward Elgar Law 2011
Edward Elgar Law 2012
Edward Elgar Law 2013

Edward Elgar Law 2014
Edward Elgar Law 2015
Edward Elgar Law 2016
Edward Elgar Law 2017
Edward Elgar Law 2018
Edward Elgar Law 2020
Edward Elgar Publications
Edward Elgar Research Reviews
Edward Elgar Social & Political Science 2020
Edward Elgar Social and Political Science 2010 and earlier
Edward Elgar Social and Political Science 2011
Edward Elgar Social and Political Science 2012
Edward Elgar Social and Political Science 2013
Edward Elgar Social and Political Science 2014
Edward Elgar Social and Political Science 2015
Edward Elgar Social and Political Science 2016
Edward Elgar Social and Political Science 2017
Edward Elgar Social and Political Science 2018
Country Commerce
Country Report
Elektra
eLibro Arquitectura, Urbanismo y Diseño
eLibro Bellas Artes, Artes Visuales y Ciencias Semióticas
eLibro Cátedra
eLibro Cátedra España
eLibro Cengage
eLibro Ciencias Biológicas, Veterinarias y Silvoagropecuarias
eLibro Ciencias de la Información y de la Comunicación
eLibro Ciencias de la Salud
eLibro Ciencias Económicas y Administrativas
eLibro Ciencias Exactas y Naturales
eLibro Ciencias Sociales
eLibro Coleta Academica
eLibro Derecho
eLibro Ecoe
eLibro Ediciones de la U
eLibro Editorial Gedisa
eLibro Educación
eLibro Ficción
eLibro Grupo Patria-Larousse

eLibro Informática, Computación y Telecomunicaciones
eLibro Ingenierías y Tecnologías
eLibro Interés General
eLibro McGraw-Hill Especial
eLibro Pearson Educación
eLibro Psicología
elibro PUCE
eLibro Wolters Kluwer Health
E-LIS (E-prints in Library and Information Science)
Backfile Package - Chemical Engineering including Supplement 1 [YUC]
Backfile Package - Computer Science including Supplement 1 [YUR]
Backfile Package - High Energy/Nuclear Physics and Astronomy including Supplement 1 [YUH]
Backfile Package - Medicine and Dentistry including Supplements 1 and 2 [YKM]
ClinicalKey España Fisterra
eBook - Agricultural, Biological, and Food Sciences 2017 [EBCABS17]
eBook - Biochemistry, Genetics and Molecular Biology 2017 [EBCBGMB17]
eBook - Biomedical Science and Medicine 2017 [EBCBSM17]
eBook - Chemical Engineering 2017 [EBCCE17]
eBook - Chemistry 2017 [EBCC17]
eBook - Computer Science 2016 [EBCCS16]
eBook - Computer Science 2017 [EBCCS17]
eBook - Earth and Planetary Sciences 2016 [EBCEPS16]
eBook - Earth and Planetary Sciences 2017 [EBCEPS17]
eBook - Energy 2016 [EBCEN16]
eBook - Energy 2017 [EBCEN17]
eBook - Forensics and Security 2017 [EBCFOR17]
eBook - Immunology and Microbiology 2017 [EBCIM17]
eBook - Materials Science 2017 [EBCMS17]
eBook - Mathematics 2017 [EBCM17]
eBook - Psychology 2017 [EBCP17]
eBook - Social Sciences 2017 [EBCSS17]
Elsevier Ebook-Agricultural, Biological, and Food Sciences 2018
Elsevier Ebook-Biochemistry, Genetics and Molecular Biology 2018
Elsevier Ebook-Biomedical Science and Medicine 2018
Elsevier Ebook-Chemical Engineering 2018
Elsevier Ebook-Chemistry 2018
Elsevier Ebook-Computer Science 2018
Elsevier Ebook-Earth and Planetary Sciences 2018
Elsevier Ebook-Energy 2018
Elsevier Ebook-Forensics and Security 2018

Elsevier Ebook-Freedom Collection Books 2017
Elsevier Ebook-Freedom Collection Books 2018
Elsevier Ebook-Insights Library 2016: Life and Biomedical Sciences
Elsevier Ebook-Insights Library 2018: Chemistry, Molecular Sciences and Chemical Engineering
Elsevier Ebook-Insights Library 2018: Computer Science
Elsevier Ebook-Insights Library 2018: Earth Systems and Environmental Sciences
Elsevier Ebook-Insights Library 2018: Energy
Elsevier Ebook-Insights Library 2018: Engineering
Elsevier Ebook-Insights Library 2018: Food Science
Elsevier Ebook-Insights Library 2018: Health Sciences Corporate
Elsevier Ebook-Insights Library 2018: Life and Biomedical Sciences
Elsevier Ebook-Insights Library 2018: Materials Science and Materials Engineering
Elsevier Ebook-Insights Library 2018: Mathematics and Physics
Elsevier Ebook-Insights Library 2018: Neuroscience and Biobehavioral Psychology
Elsevier Ebook-Insights Library 2018: Social Sciences
Elsevier Ebook-Materials Science 2018
Elsevier Ebook-Mathematics 2018
Elsevier Ebook-Neuroscience 2018
Elsevier Ebook-Psychology 2018
Elsevier Ebook-Social Sciences 2018
Elsevier Fc Complete Collection Books 2018
Scopus
Elsevier ClinicalKey Australia Books
Elsevier ClinicalKey Books
Elsevier ClinicalKey Nursing Australia Books
Elsevier ClinicalKey Nursing Australia Journals
Elsevier ClinicalKey Student Foundation Medical English Language Dentistry Package
Elsevier ClinicalKey Student Foundation Medical English Language Review Package
Elsevier ClinicalKey Student Foundation Medical Spanish Package
Elsevier ClinicalKey Student Foundation Nursing English Language Part 1 Package
Elsevier ClinicalKey Student Foundation Nursing English Language Part 2 Package
Elsevier ClinicalKey Nursing Ebooks
CRKN Elsevier Academic Press Journals
CRKN Elsevier Additional Journals
eBook - Health Professions 2018 [EBCHP18]
eBook - Immunology and Microbiology 2018 [EBCIM18]
eBook - Physics and Astronomy 2018 [EBCPA18]
eBook - Veterinary Medicine 2018 [EBCVM18]
Elsevier Handbooks in Economics Series
Elsevier ScienceDirect Book Series Backfile Package - Agricultural and Biological Sciences

Elsevier ScienceDirect Book Series Backfile Package - All Subjects
Elsevier ScienceDirect Book Series Backfile Package - Biochemistry, Genetics and Molecular Biology
Elsevier ScienceDirect Book Series Backfile Package - Engineering
Elsevier ScienceDirect Book Series Backfile Package - Immunology and Microbiology
Elsevier ScienceDirect Book Series Backfile Package - Life Sciences
Elsevier ScienceDirect Book Series Backfile Package - Neuroscience
Elsevier ScienceDirect Book Series Backfile Package - Pharmacology, Toxicology and Pharmaceutical Science
Elsevier ScienceDirect Book Series Backfile Package - Physics and Astronomy
Elsevier ScienceDirect Book Series Backfile Package - Psychology
Elsevier ScienceDirect Book Series Package - Agricultural and Biological Sciences
Elsevier ScienceDirect Book Series Package - All Subjects
Elsevier ScienceDirect Book Series Package - Biochemistry, Genetics and Molecular Biology
Elsevier ScienceDirect Book Series Package - Chemistry
Elsevier ScienceDirect Book Series Package - Engineering
Elsevier ScienceDirect Book Series Package - Immunology and Microbiology
Elsevier ScienceDirect Book Series Package - Life Sciences
Elsevier ScienceDirect Book Series Package - Life Sciences (Legacy)
Elsevier ScienceDirect Book Series Package - Methods in Cell Biology (1964-1999)
Elsevier ScienceDirect Book Series Package - Methods in Cell Biology (2000-ongoing)
Elsevier ScienceDirect Book Series Package - Neuroscience
Elsevier ScienceDirect Book Series Package - Pharmacology, Toxicology and Pharmaceutical Science
Elsevier ScienceDirect Book Series Package - Physics and Astronomy
Elsevier ScienceDirect Book Series Package - Psychology
Elsevier ScienceDirect Book Series Package - Psychology (Legacy)
Elsevier ScienceDirect Book Series Package - Side Effects of Drugs Annual (1977-1999)
Elsevier ScienceDirect Book Series Package - Side Effects of Drugs Annual (2000-ongoing)
Elsevier ScienceDirect Book Series Package - Social Sciences
Elsevier ScienceDirect Books
Elsevier ScienceDirect Books Complete
Elsevier ScienceDirect Corporate Edition Book Collection
Elsevier ScienceDirect eBook - Agricultural and Biological Sciences (Legacy 1)
Elsevier ScienceDirect eBook - Agricultural and Biological Sciences (Legacy 2)
Elsevier ScienceDirect eBook - Agricultural and Biological Sciences 1995 - 2006
Elsevier ScienceDirect eBook - Agricultural and Biological Sciences 2007
Elsevier ScienceDirect eBook - Agricultural and Biological Sciences 2008
Elsevier ScienceDirect eBook - Agricultural and Biological Sciences 2009
Elsevier ScienceDirect eBook - Agricultural and Biological Sciences 2010
Elsevier ScienceDirect eBook - Agricultural and Biological Sciences 2011
Elsevier ScienceDirect eBook - Agricultural and Biological Sciences 2012
Elsevier ScienceDirect eBook - Agricultural and Biological Sciences 2013

Elsevier ScienceDirect eBook - Agricultural and Biological Sciences pre-2007
Elsevier ScienceDirect eBook - Agricultural and Biological Sciences Supplement pre-1995
Elsevier ScienceDirect eBook - Agricultural, Biological, and Food Sciences 2007
Elsevier ScienceDirect eBook - Agricultural, Biological, and Food Sciences 2007 - 2013
Elsevier ScienceDirect eBook - Agricultural, Biological, and Food Sciences 2008
Elsevier ScienceDirect eBook - Agricultural, Biological, and Food Sciences 2009
Elsevier ScienceDirect eBook - Agricultural, Biological, and Food Sciences 2010
Elsevier ScienceDirect eBook - Agricultural, Biological, and Food Sciences 2011
Elsevier ScienceDirect eBook - Agricultural, Biological, and Food Sciences 2012
Elsevier ScienceDirect eBook - Agricultural, Biological, and Food Sciences 2013
Elsevier ScienceDirect eBook - Agricultural, Biological, and Food Sciences 2014
Elsevier ScienceDirect eBook - Agricultural, Biological, and Food Sciences 2015
Elsevier ScienceDirect eBook - Agricultural, Biological, and Food Sciences 2016
Elsevier ScienceDirect eBook - Agricultural, Biological, and Food Sciences pre-2007
Elsevier ScienceDirect eBook - AGRICULTUREnetBASE
Elsevier ScienceDirect eBook - Arts and Humanities (Legacy 1)
Elsevier ScienceDirect eBook - Biochemistry, Genetics and Molecular Biology (Legacy 1)
Elsevier ScienceDirect eBook - Biochemistry, Genetics and Molecular Biology (Legacy 2)
Elsevier ScienceDirect eBook - Biochemistry, Genetics and Molecular Biology 1995 - 2006
Elsevier ScienceDirect eBook - Biochemistry, Genetics and Molecular Biology 2007
Elsevier ScienceDirect eBook - Biochemistry, Genetics and Molecular Biology 2008
Elsevier ScienceDirect eBook - Biochemistry, Genetics and Molecular Biology 2009
Elsevier ScienceDirect eBook - Biochemistry, Genetics and Molecular Biology 2010
Elsevier ScienceDirect eBook - Biochemistry, Genetics and Molecular Biology 2011
Elsevier ScienceDirect eBook - Biochemistry, Genetics and Molecular Biology 2012
Elsevier ScienceDirect eBook - Biochemistry, Genetics and Molecular Biology 2013
Elsevier ScienceDirect eBook - Biochemistry, Genetics and Molecular Biology 2014
Elsevier ScienceDirect eBook - Biochemistry, Genetics and Molecular Biology 2015
Elsevier ScienceDirect eBook - Biochemistry, Genetics and Molecular Biology 2016
Elsevier ScienceDirect eBook - Biomedical Science and Medicine (Legacy 1)
Elsevier ScienceDirect eBook - Biomedical Science and Medicine 2011
Elsevier ScienceDirect eBook - Biomedical Science and Medicine 2012
Elsevier ScienceDirect eBook - Biomedical Science and Medicine 2013
Elsevier ScienceDirect eBook - Biomedical Science and Medicine 2014
Elsevier ScienceDirect eBook - Biomedical Science and Medicine 2015
Elsevier ScienceDirect eBook - Biomedical Science and Medicine 2016
Elsevier ScienceDirect eBook - Biomedicine 2011
Elsevier ScienceDirect eBook - Biomedicine 2012
Elsevier ScienceDirect eBook - Biomedicine 2013
Elsevier ScienceDirect eBook - Biomedicine 2014

Elsevier ScienceDirect eBook - BIOSCIENCEnetBASE
Elsevier ScienceDirect eBook - Brazil Business, Science and Technology
Elsevier ScienceDirect eBook - Brazil Business, Science and Technology 2013
Elsevier ScienceDirect eBook - Chemical Engineering (Legacy 1)
Elsevier ScienceDirect eBook - Chemical Engineering (Legacy 2)
Elsevier ScienceDirect eBook - Chemical Engineering 1995 - 2006
Elsevier ScienceDirect eBook - Chemical Engineering 2007
Elsevier ScienceDirect eBook - Chemical Engineering 2008
Elsevier ScienceDirect eBook - Chemical Engineering 2009
Elsevier ScienceDirect eBook - Chemical Engineering 2010
Elsevier ScienceDirect eBook - Chemical Engineering 2011
Elsevier ScienceDirect eBook - Chemical Engineering 2012
Elsevier ScienceDirect eBook - Chemical Engineering 2013
Elsevier ScienceDirect eBook - Chemical Engineering 2014
Elsevier ScienceDirect eBook - Chemical Engineering 2015
Elsevier ScienceDirect eBook - Chemical Engineering 2016
Elsevier ScienceDirect eBook - Chemical Engineering pre-2007
Elsevier ScienceDirect eBook - Chemical Engineering Supplement pre-1995
Elsevier ScienceDirect eBook - Chemistry (Legacy 1)
Elsevier ScienceDirect eBook - Chemistry (Legacy 2)
Elsevier ScienceDirect eBook - Chemistry 1995 - 2006
Elsevier ScienceDirect eBook - Chemistry 2007
Elsevier ScienceDirect eBook - Chemistry 2008
Elsevier ScienceDirect eBook - Chemistry 2009
Elsevier ScienceDirect eBook - Chemistry 2010
Elsevier ScienceDirect eBook - Chemistry 2011
Elsevier ScienceDirect eBook - Chemistry 2012
Elsevier ScienceDirect eBook - Chemistry 2013
Elsevier ScienceDirect eBook - Chemistry 2014
Elsevier ScienceDirect eBook - Chemistry 2015
Elsevier ScienceDirect eBook - Chemistry 2016
Elsevier ScienceDirect eBook - Chemistry pre-2007
Elsevier ScienceDirect eBook - Chemistry Supplement pre-1995
Elsevier ScienceDirect eBook - CHEMLIBnetBASE
Elsevier ScienceDirect eBook - CLEANTECHnetBASE
Elsevier ScienceDirect eBook - Clinical Dentistry 2000-2010
Elsevier ScienceDirect eBook - Clinical Medicine 2000 - 2007
Elsevier ScienceDirect eBook - Clinical Medicine 2007
Elsevier ScienceDirect eBook - Clinical Medicine 2008
Elsevier ScienceDirect eBook - Clinical Medicine 2009

Elsevier ScienceDirect eBook - Clinical Medicine 2010
Elsevier ScienceDirect eBook - Clinical Medicine 2011
Elsevier ScienceDirect eBook - Clinical Medicine 2012
Elsevier ScienceDirect eBook - Clinical Medicine 2013
Elsevier ScienceDirect eBook - Clinical Medicine pre-2007
Elsevier ScienceDirect eBook - Elsevier Masson French Health Sciences Collection 2007 - 2010 (2)
Elsevier ScienceDirect eBook - Elsevier Masson French Health Sciences Collection 2007-2010
Elsevier ScienceDirect eBook - Elsevier Masson French Health Sciences Collection 2011
Elsevier ScienceDirect eBook - Elsevier Masson French Health Sciences Collection 2012
Elsevier ScienceDirect eBook - Elsevier Masson French Health Sciences Collection 2013
Elsevier ScienceDirect eBook - Elsevier Masson French Health Sciences Collection 2014
Elsevier ScienceDirect eBook - Elsevier Masson French Health Sciences Collection 2015
Elsevier ScienceDirect eBook - Elsevier Masson French Health Sciences Collection 2016
Elsevier ScienceDirect eBook - Engineering 2016
Elsevier ScienceDirect eBook - Engineering 2017
Elsevier ScienceDirect eBook - Engineering 2018
Elsevier ScienceDirect eBook - Environmental Science 2016
Elsevier ScienceDirect eBook - Environmental Science 2017
Elsevier ScienceDirect eBook - Environmental Science 2018
Elsevier ScienceDirect eBook - Finance 2016
Elsevier ScienceDirect eBook - Finance 2017
Elsevier ScienceDirect eBook - Finance 2018
Elsevier ScienceDirect eBook - Freedom Collection Books 2020
Elsevier ScienceDirect eBook - Health Professions 2000 - 2007
Elsevier ScienceDirect eBook - Health Professions 2007
Elsevier ScienceDirect eBook - Health Professions 2008
Elsevier ScienceDirect eBook - Health Professions 2009
Elsevier ScienceDirect eBook - Health Professions 2010
Elsevier ScienceDirect eBook - Health Professions 2011
Elsevier ScienceDirect eBook - Health Professions 2012
Elsevier ScienceDirect eBook - Health Professions 2013
Elsevier ScienceDirect eBook - Health Professions 2014
Elsevier ScienceDirect eBook - Health Professions 2015
Elsevier ScienceDirect eBook - Health Professions 2016
Elsevier ScienceDirect eBook - Health Professions pre-2007
Elsevier ScienceDirect eBook - Immunology and Microbiology 1995 - 2006
Elsevier ScienceDirect eBook - Immunology and Microbiology 2007
Elsevier ScienceDirect eBook - Immunology and Microbiology 2008
Elsevier ScienceDirect eBook - Immunology and Microbiology 2009
Elsevier ScienceDirect eBook - Immunology and Microbiology 2010

Elsevier ScienceDirect eBook - Immunology and Microbiology 2011
Elsevier ScienceDirect eBook - Immunology and Microbiology 2012
Elsevier ScienceDirect eBook - Immunology and Microbiology 2013
Elsevier ScienceDirect eBook - Immunology and Microbiology 2014
Elsevier ScienceDirect eBook - Immunology and Microbiology 2015
Elsevier ScienceDirect eBook - Immunology and Microbiology 2016
Elsevier ScienceDirect eBook - INDUSTRIALnetBASE
Elsevier ScienceDirect eBook - Information and Library Management 2009 - 2011
Elsevier ScienceDirect eBook - Information and Library Management 2012
Elsevier ScienceDirect eBook - Information and Library Management 2013
Elsevier ScienceDirect eBook - Information and Library Management 2014
Elsevier ScienceDirect eBook - Information and Library Management pre-2008
Elsevier ScienceDirect eBook - Information Management 2009 - 2011
Elsevier ScienceDirect eBook - Information Management 2012
Elsevier ScienceDirect eBook - Information Management 2013
Elsevier ScienceDirect eBook - Information Management pre-2008
Elsevier ScienceDirect eBook - INFOSECURITYNETBASE
Elsevier ScienceDirect eBook - Insights Library 2015: Computer Science
Elsevier ScienceDirect eBook - Insights Library 2015: Earth Systems and Environmental Sciences
Elsevier ScienceDirect eBook - Insights Library 2015: Energy
Elsevier ScienceDirect eBook - Insights Library 2015: Engineering
Elsevier ScienceDirect eBook - Insights Library 2015: Food Science
Elsevier ScienceDirect eBook - Insights Library 2015: Life and Biomedical Sciences
Elsevier ScienceDirect eBook - Insights Library 2015: Materials Science and Materials Engineering
Elsevier ScienceDirect eBook - Insights Library 2015: Mathematics and Physics
Elsevier ScienceDirect eBook - Insights Library 2015: Neuroscience and Biobehavioral Psychology
Elsevier ScienceDirect eBook - Insights Library 2015: Social Sciences
Elsevier ScienceDirect eBook - Insights Library 2016: Chemistry, Molecular Sciences and Chemical Engineering
Elsevier ScienceDirect eBook - Insights Library 2016: Computer Science
Elsevier ScienceDirect eBook - Insights Library 2016: Earth Systems and Environmental Sciences
Elsevier ScienceDirect eBook - Insights Library 2016: Energy
Elsevier ScienceDirect eBook - Insights Library 2016: Engineering
Elsevier ScienceDirect eBook - Insights Library 2016: Food Science
Elsevier ScienceDirect eBook - Insights Library 2016: Life and Biomedical Sciences
Elsevier ScienceDirect eBook - Insights Library 2016: Materials Science and Materials Engineering
Elsevier ScienceDirect eBook - Insights Library 2016: Mathematics and Physics
Elsevier ScienceDirect eBook - Insights Library 2016: Neuroscience and Biobehavioral Psychology
Elsevier ScienceDirect eBook - Insights Library 2016: Social Sciences
Elsevier ScienceDirect eBook - Insights Library 2017: Chemistry, Molecular Sciences and Chemical Engineering
Elsevier ScienceDirect eBook - Insights Library 2017: Computer Science

Elsevier ScienceDirect eBook - Insights Library 2017: Earth Systems and Environmental Sciences
Elsevier ScienceDirect eBook - Insights Library 2017: Energy
Elsevier ScienceDirect eBook - Insights Library 2017: Engineering
Elsevier ScienceDirect eBook - Insights Library 2017: Food Science
Elsevier ScienceDirect eBook - Insights Library 2017: Health Sciences Corporate
Elsevier ScienceDirect eBook - Insights Library 2017: Life and Biomedical Sciences
Elsevier ScienceDirect eBook - Insights Library 2017: Materials Science and Materials Engineering
Elsevier ScienceDirect eBook - Insights Library 2017: Mathematics and Physics
Elsevier ScienceDirect eBook - Insights Library 2017: Neuroscience and Biobehavioral Psychology
Elsevier ScienceDirect eBook - Insights Library 2017: Social Sciences
Elsevier ScienceDirect eBook - Materials Science 1995 - 2006
Elsevier ScienceDirect eBook - Materials Science 2007
Elsevier ScienceDirect eBook - Materials Science 2008
Elsevier ScienceDirect eBook - Materials Science 2009
Elsevier ScienceDirect eBook - Materials Science 2010
Elsevier ScienceDirect eBook - Materials Science 2011
Elsevier ScienceDirect eBook - Materials Science 2012
Elsevier ScienceDirect eBook - Materials Science 2013
Elsevier ScienceDirect eBook - Materials Science 2014
Elsevier ScienceDirect eBook - Materials Science 2015
Elsevier ScienceDirect eBook - Materials Science 2016
Elsevier ScienceDirect eBook - Materials Science pre-2007
Elsevier ScienceDirect eBook - Mathematics 1995 - 2006
Elsevier ScienceDirect eBook - Mathematics 2007
Elsevier ScienceDirect eBook - Mathematics 2008
Elsevier ScienceDirect eBook - Mathematics 2009
Elsevier ScienceDirect eBook - Mathematics 2010
Elsevier ScienceDirect eBook - Mathematics 2011
Elsevier ScienceDirect eBook - Mathematics 2012
Elsevier ScienceDirect eBook - Mathematics 2013
Elsevier ScienceDirect eBook - Mathematics 2014
Elsevier ScienceDirect eBook - Mathematics 2015
Elsevier ScienceDirect eBook - Mathematics 2016
Elsevier ScienceDirect eBook - Mathematics pre-2007
Elsevier ScienceDirect eBook - Medicine and Dentistry 1995 - 2006
Elsevier ScienceDirect eBook - Medicine and Dentistry 2007
Elsevier ScienceDirect eBook - Medicine and Dentistry 2008
Elsevier ScienceDirect eBook - Medicine and Dentistry 2009
Elsevier ScienceDirect eBook - Medicine and Dentistry 2010
Elsevier ScienceDirect eBook - Medicine and Dentistry 2011

Elsevier ScienceDirect eBook - MRW subscription package: Encyclopedia of Atmospheric Sciences
Elsevier ScienceDirect eBook - MRW subscription package: Encyclopedia of Biological Chemistry
Elsevier ScienceDirect eBook - MRW subscription package: Encyclopedia of Dairy Sciences
Elsevier ScienceDirect eBook - MRW subscription package: Encyclopedia of Food Microbiology
Elsevier ScienceDirect eBook - MRW subscription package: Encyclopedia of Forensic Sciences
Elsevier ScienceDirect eBook - MRW subscription package: Encyclopedia of Human Nutrition
Elsevier ScienceDirect eBook - MRW subscription package: Encyclopedia of Meat Sciences
Elsevier ScienceDirect eBook - MRW subscription package: Encyclopedia of Ocean Sciences
Elsevier ScienceDirect eBook - MRW subscription package: Encyclopedia of Quaternary Science
Elsevier ScienceDirect eBook - MRW subscription package: Encyclopedia of Spectroscopy and Spectrometry
Elsevier ScienceDirect eBook - MRW subscription package: Encyclopedia of the Neurological Sciences
Elsevier ScienceDirect eBook - MRW subscription package: Encyclopedia of Virology
Elsevier ScienceDirect eBook - MRW subscription package: International Encyclopedia of the Social & Behavioral Sciences
Elsevier ScienceDirect eBook - MRW subscription package: Treatise on Geochemistry
Elsevier ScienceDirect eBook - Neuroscience 1995 - 2006
Elsevier ScienceDirect eBook - Neuroscience 2007
Elsevier ScienceDirect eBook - Neuroscience 2008
Elsevier ScienceDirect eBook - Neuroscience 2009
Elsevier ScienceDirect eBook - Neuroscience 2010
Elsevier ScienceDirect eBook - Neuroscience 2011
Elsevier ScienceDirect eBook - Neuroscience 2012
Elsevier ScienceDirect eBook - Neuroscience 2013
Elsevier ScienceDirect eBook - Neuroscience 2014
Elsevier ScienceDirect eBook - Neuroscience 2015
Elsevier ScienceDirect eBook - Neuroscience 2016
Elsevier ScienceDirect eBook - Neuroscience 2017
Elsevier ScienceDirect eBook - Pharmacology, Toxicology and Pharmaceutical Science 2016
Elsevier ScienceDirect eBook - Pharmacology, Toxicology and Pharmaceutical Science 2017
Elsevier ScienceDirect eBook - Pharmacology, Toxicology and Pharmaceutical Science 2018
Elsevier ScienceDirect eBook - Physics and Astronomy 1995 - 2006
Elsevier ScienceDirect eBook - Physics and Astronomy 2007
Elsevier ScienceDirect eBook - Physics and Astronomy 2008
Elsevier ScienceDirect eBook - Physics and Astronomy 2009
Elsevier ScienceDirect eBook - Physics and Astronomy 2010
Elsevier ScienceDirect eBook - Physics and Astronomy 2011
Elsevier ScienceDirect eBook - Physics and Astronomy 2012
Elsevier ScienceDirect eBook - Physics and Astronomy 2013
Elsevier ScienceDirect eBook - Physics and Astronomy 2014
Elsevier ScienceDirect eBook - Physics and Astronomy 2015
Elsevier ScienceDirect eBook - Physics and Astronomy 2016

Elsevier ScienceDirect eBook - Physics and Astronomy pre-2007
Elsevier ScienceDirect eBook - Physics and Astronomy Supplement pre-1995
Elsevier ScienceDirect eBook - Plastics Engineering Hanser 2005 - 2011
Elsevier ScienceDirect eBook - Plastics Engineering Hanser 2013
Elsevier ScienceDirect eBook - Plastics Engineering Hanser 2014
Elsevier ScienceDirect eBook - Plastics Engineering Hanser 2015
Elsevier ScienceDirect eBook - Psychology 1995 - 2006
Elsevier ScienceDirect eBook - Psychology 2007
Elsevier ScienceDirect eBook - Psychology 2008
Elsevier ScienceDirect eBook - Psychology 2009
Elsevier ScienceDirect eBook - Psychology 2010
Elsevier ScienceDirect eBook - Psychology 2011
Elsevier ScienceDirect eBook - Psychology 2012
Elsevier ScienceDirect eBook - Psychology 2013
Elsevier ScienceDirect eBook - Psychology 2014
Elsevier ScienceDirect eBook - Psychology 2015
Elsevier ScienceDirect eBook - Psychology 2016
Elsevier ScienceDirect eBook - Psychology pre-2007
Elsevier ScienceDirect eBook - Psychology Supplement pre-1995
Elsevier ScienceDirect eBook - SciVerse Hightech Edition - eBook Collection
Elsevier ScienceDirect eBook - Specialty Medicine 2014
Elsevier ScienceDirect eBook - Specialty Medicine 2015
Elsevier ScienceDirect eBook - Specialty Medicine 2016
Elsevier ScienceDirect eBook - Veterinary Medicine 2007
Elsevier ScienceDirect eBook - Veterinary Medicine 2008
Elsevier ScienceDirect eBook - Veterinary Medicine 2009
Elsevier ScienceDirect eBook - Veterinary Medicine 2010
Elsevier ScienceDirect eBook - Veterinary Medicine 2011
Elsevier ScienceDirect eBook - Veterinary Medicine 2012
Elsevier ScienceDirect eBook - Veterinary Medicine 2013
Elsevier ScienceDirect eBook - Veterinary Medicine 2014
Elsevier ScienceDirect eBook - Veterinary Medicine 2015
Elsevier ScienceDirect eBook - Veterinary Medicine 2016
Elsevier ScienceDirect eBook - Veterinary Medicine pre-2007
Elsevier ScienceDirect eBook - Veterinary Science and Veterinary Medicine 2007
Elsevier ScienceDirect eBook - Veterinary Science and Veterinary Medicine 2008
Elsevier ScienceDirect eBook - William Andrew Chemical Engineering 2008
Elsevier ScienceDirect eBooks Freedom Collection 2012
Elsevier ScienceDirect eBooks Freedom Collection 2013
Elsevier ScienceDirect eBooks Freedom Collection 2014

Elsevier ScienceDirect eBooks Freedom Collection 2017
Elsevier ScienceDirect eBooks Freedom Collection 2019
Elsevier ScienceDirect FC Complete Collection Books 2008
Elsevier ScienceDirect FC Complete Collection Books 2009
Elsevier ScienceDirect FC Complete Collection Books 2010
Elsevier ScienceDirect FC Complete Collection Books 2011
Elsevier ScienceDirect FC Complete Collection Books 2015
Elsevier ScienceDirect FC Complete Collection Books 2016
Elsevier ScienceDirect FC Complete Collection Books 2017
Elsevier ScienceDirect Handbook Series Backfile Package: Handbooks in Economics Series
Elsevier ScienceDirect Handbook Series Backfile Package: Handbooks in Operations Research and Management Science
Elsevier ScienceDirect Handbook Series Package: Handbooks in Economics Series
Elsevier ScienceDirect Journals
Elsevier ScienceDirect Journals Complete
Elsevier ScienceDirect Textbooks
Elsevier SD Agricultural and Biological Sciences
Elsevier SD Biochemistry Genetics and Molecular Biology
Elsevier SD Book Series Package - Methods in Enzymology (1955-1999)
Elsevier SD Book Series Package - Methods in Enzymology (2000-ongoing)
Elsevier SD Business Management and Accounting
Elsevier SD Cell Press
Elsevier SD Chemical Engineering
Elsevier SD Chemistry
Elsevier SD College Edition Health & Life Sciences
Elsevier SD College Edition Physical Sciences
Elsevier SD College Edition Social & Behavioral Sciences
Elsevier SD Computer Science
Elsevier SD Corporate Edition
Elsevier SD Decision Sciences
Elsevier SD Earth and Planetary Sciences
Elsevier SD Economics Econometrics and Finance
Elsevier SD Ediciones Doyma
Elsevier SD Energy
Elsevier SD Engineering
Elsevier SD Environmental Science
Elsevier SD Freedom Collection
Elsevier SD Health Sciences
Elsevier SD Immunology and Microbiology
Elsevier SD Materials Science
Elsevier SD Mathematics

Elsevier SD Neuroscience
Elsevier SD Pharmacology Toxicology and Pharmaceutical Science
Elsevier SD Physics and Astronomy
Elsevier SD Psychology
Elsevier SD Reference Works
Elsevier SD Social Sciences
Elsevier SD Urban & Fischer
EZB-NALI5-00465 Elsevier Archive NL
KB+ BIBSAM Elsevier Cell Press 2014-2016
KB+ BIBSAM Elsevier Cell Press 2017
KB+ Bibsam Elsevier Cell Press 2018
KB+ BIBSAM Elsevier Cell Press Pta Package 2018
KB+ Bibsam Elsevier Journal Outside Freedom Collection 2018
KB+ BIBSAM Elsevier Journals Outside Freedom Collection 2014-2016
KB+ BIBSAM Elsevier Journals Outside Freedom Collection 2017
KB+ BIBSAM Elsevier Journals Outside Freedom Collection Pta Package 2018
KB+ BIBSAM Elsevier SD Freedom Collection 2017
KB+ Bibsam Elsevier SD Freedom Collection 2018
KB+ JISC Collections Elsevier Cell Press 2012-2016
KB+ JISC Collections Elsevier ScienceDirect Freedom Collection 2017-2021
KB+ JISC Collections Elsevier SD Agricultural And Biological Sciences 2012-2016
KB+ JISC Collections Elsevier SD Biochem Genetics And Molecular Biology 2012-2016
KB+ JISC Collections Elsevier SD Business Management And Accounting 2012-2016
KB+ JISC Collections Elsevier SD Chemical Engineering 2012-2016
KB+ JISC Collections Elsevier SD Chemistry 2012-2016
KB+ JISC Collections Elsevier SD Computer Science 2012-2016
KB+ JISC Collections Elsevier SD Decision Sciences 2014-2016
KB+ JISC Collections Elsevier SD Earth And Planetary Sciences 2012-2016
KB+ JISC Collections Elsevier SD Economics Econometrics And Finance 2012-2016
KB+ JISC Collections Elsevier SD Energy 2012-2016
KB+ JISC Collections Elsevier SD Engineering 2012-2016
KB+ JISC Collections Elsevier SD Environmental Science 2012-2016
KB+ JISC Collections Elsevier SD Freedom Collection 2012-2016
KB+ JISC Collections Elsevier SD Health Sciences 2012-2016
KB+ JISC Collections Elsevier SD Immunology And Microbiology 2012-2016
KB+ JISC Collections Elsevier SD Materials Science 2012-2016
KB+ JISC Collections Elsevier SD Mathematics 2012-2016
KB+ JISC Collections Elsevier SD Mathematics Core 2012-2016
KB+ JISC Collections Elsevier SD Neuroscience 2012-2016
KB+ JISC Collections Elsevier SD Nursing And Health Professions 2012-2016

KB+ JISC Collections Elsevier SD Pharmacology Toxicology And Pharmaceutics 2012-2016
KB+ JISC Collections Elsevier SD Physics And Astronomy 2012-2016
KB+ JISC Collections Elsevier SD Psychology 2012-2016
KB+ JISC Collections Elsevier SD Social Science 2012-2016
KB+ JISC Collections Elsevier SD Veterinary Science And Veterinary Medicine2012-2016
NESLI2 Elsevier ScienceDirect Freedom Collection
ScienceDirect (EiRA)
ScienceDirect German Medical Collection 2013 [EBCHSGER13]
ScienceDirect German Medical Collection 2014 [EBCHSGER14]
ScienceDirect German Medical Collection 2015 [EBCHSGER15]
ScienceDirect German Medical Collection 2016 [EBCHSGER16]
ScienceDirect German Medical Collection 2017 [EBCHSGER17]
Business, Management and Accounting including Supplement 1
Elsevier ScienceDirect Backfile Cell Press
Elsevier SD Backfile Agricultural
Elsevier SD Backfile Agriculture Supplement
Elsevier SD Backfile Allergology, Rheumatology and Immunology
Elsevier SD Backfile Anesthesiology, Pain Medicine, Emergency Medicine, Critical Care and Intensive Medicine
Elsevier SD Backfile Biochemistry
Elsevier SD Backfile Biochemistry Supplement
Elsevier SD Backfile Business
Elsevier SD Backfile Chemical Engineering
Elsevier SD Backfile Chemistry All
Elsevier SD Backfile Clinical Neurology
Elsevier SD Backfile Complete
Elsevier SD Backfile Computer
Elsevier SD Backfile Decision Sciences
Elsevier SD Backfile Dentistry Oral Surgery and Medicine
Elsevier SD Backfile Earth & Planetary Sciences
Elsevier SD Backfile Earth & Planetary Sciences Supplement
Elsevier SD Backfile Economics
Elsevier SD Backfile Economics Supplement
Elsevier SD Backfile Energy
Elsevier SD Backfile Engineering and Technology
Elsevier SD Backfile Environment Sciences Supplement
Elsevier SD Backfile Environmental Sciences
Elsevier SD Backfile Forensic Medicine, Pathology and Medical Technology
Elsevier SD Backfile Gastroenterology, Endocrinology, Diabetes and Metabolism
Elsevier SD Backfile General Medicine
Elsevier SD Backfile Hematology, Cardiology and Cardiovascular Medicine

Elsevier SD Backfile High Energy
Elsevier SD Backfile Immunology & Microbiology
Elsevier SD Backfile Immunology Supplement
Elsevier SD Backfile Inorganic Chemistry
Elsevier SD Backfile Inorganic Chemistry Supplement
Elsevier SD Backfile Materials Science
Elsevier SD Backfile Mathematics
Elsevier SD Backfile Mathematics Supplement
Elsevier SD Backfile Medicine and Dentistry
Elsevier SD Backfile Medicine and Dentistry Supplement 1
Elsevier SD Backfile Neuroscience
Elsevier SD Backfile Neuroscience Supplement
Elsevier SD Backfile Nursing and Health Professions
Elsevier SD Backfile Obstetrics, Gynecology and Women's Health
Elsevier SD Backfile Oncology
Elsevier SD Backfile Organic Chemistry
Elsevier SD Backfile Organic Chemistry Supplement
Elsevier SD Backfile Orthopedics, Sports Medicine and Rehabilitation
Elsevier SD Backfile Perinatology, Pediatrics and Child Health
Elsevier SD Backfile Pharmacology
Elsevier SD Backfile Pharmacology Supplement
Elsevier SD Backfile Physical and Analytical Chemistry
Elsevier SD Backfile Physical and Analytical Chemistry Supplement
Elsevier SD Backfile Physics
Elsevier SD Backfile Physics Supplement
Elsevier SD Backfile Psychiatry and Mental Health
Elsevier SD Backfile Psychology
Elsevier SD Backfile Psychology Supplement
Elsevier SD Backfile Public Health and Health Policy
Elsevier SD Backfile Radiology and Imaging
Elsevier SD Backfile Respiratory, Pulmonary and Infectious Diseases
Elsevier SD Backfile Social Science Supplement
Elsevier SD Backfile Social Sciences
Elsevier SD Backfile Surgery
Elsevier SD Backfile Veterinary
EMANI - Electronic Mathematics Archives Network Initiative
Embry-Riddle Aeronautical University (Daytona) Scholarly Commons
Embry-Riddle Aeronautical University ASASA
(TAEBC): Emerald, 2012
DRAA Emerald Management e-Journals 268

DRAA Emerald Management e-Journals 276
Emerald - Licence Nationale (ISTEX)
Emerald - SURFmarket
Emerald Accounting and Finance eJournal Collection
Emerald A-Z Current Journals
Emerald Backfiles
Emerald Backfiles-DRAA
Emerald Books Business Management And Economics Subscription
Emerald Books Transport
Emerald Built Environment eJournal Collection
Emerald Business, Management and Economics eBook Series Collection
Emerald Business, Management and Strategy eJournal Collection
Emerald Business, Management and Strategy eJournal Collection 2015
Emerald Complete Journals
Emerald Criminology / Forensic Psychology eJournal Collection
Emerald eBook Series-中區教學資源(Taiwan)
Emerald eCase Collection
Emerald Education eJournal Collection
Emerald eJournals Premier
Emerald Engineering Backfiles
Emerald Engineering eJournal Collection
Emerald Engineering, Computing & Technology Collection KERIS
Emerald Fulltext Archive Database (DFG Nationallizenzen)
Emerald Health and Social Care eJournal Collection
Emerald HR, Learning and Organization Studies eJournal Collection
Emerald Information and Knowledge Management eJournal Collection
Emerald Learning / Intellectual Disability eJournal Collection
Emerald Library Studies eJournal Collection
Emerald Management 110
Emerald Management 111
Emerald Management 120
Emerald Management 125
Emerald Management 140
Emerald Management 150 - CONCERT
Emerald Management 160
Emerald Management 175
Emerald Management 200
Emerald Management 40
Emerald Management 60
Emerald Management 80

Emerald Management 95
Emerald Management eJournals Collection
Emerald Management First 120
Emerald Management First 175
Emerald Management First 200
Emerald Management First 60
Emerald Management First Health and Social Care
Emerald Management First Public Sector Zone
Emerald Management Plus
Emerald Marketing eJournal Collection
Emerald Mental Health eJournal Collection
Emerald Open Access
Emerald Operations and Logistics and Quality eJournal Collection
Emerald Property Management & Built Environment eJournal Collection
Emerald Public Policy and Environmental Management eJournal Collection
Emerald Social Science eBook Series Collection
Emerald Specialist Collection KERIS
Emerald Tourism and Hospitality eJournal Collection
Emerald Vulnerable Groups eJournal Collection
Emerald:Backfile Collection:2013 - NESLI2
KB+ BIBSAM Emerald eJournals Premier 2014-2016
KB+ BIBSAM Emerald Management Plus Static 2014-2016
KB+ Eduserv Emerald Engineering 2015-2017
KB+ Eduserv Emerald Health And Social Care 2015-2017
KB+ Eduserv Emerald Library Studies 2015-2017
KB+ Eduserv Emerald Management 2015-2017 (150 Titles)
KB+ Eduserv Emerald Management 2015-2017 (175 Titles)
KB+ Eduserv Emerald Management Accounting Finance And Economics Collection 2015-2017
KB+ Eduserv Emerald Management Business Management And Strategy Collection 2015-2017
KB+ Eduserv Emerald Management Human Resources Learning And Organization Studies Collection 2015-2017
KB+ Eduserv Emerald Management Information And Knowledge Management Collection 2015-2017
KB+ Eduserv Emerald Management Marketing Collection 2015-2017
KB+ Eduserv Emerald Management Operations Logistics And Quality Collection 2015-2017
KB+ Eduserv Emerald Management Plus 2015-2017
KB+ Eduserv Emerald Management Premier Collection 2015-2017
KB+ Eduserv Emerald Management Property Management And Built Environment Collection 2015-2017
Emerald Emerging Markets Case Studies Collection
Transport eBook Collection
Emis
Britannica Library Adults (Australian)

Britannica Library Kids (Australian)
Britannica Library Teens (Australian)
Britannica Online Academic Edition
Britannica Online Public Library Edition
Britannica Online Public Library Edition for Kids
Britannica Online School Edition
Britannica Online School Edition: Compton''s
Britannica Premium Encyclopedia
Encyclopædia Britannica
Encyclopedia Britannica Academic Edition
Encyclopedia Britannica Elementary School Edition
Encyclopedia Britannica High School Edition
Encyclopedia Britannica Middle School Edition
Encyclopædia Universalis
Endocrine Society
Endocrine Society Journals
Entomological Society of Canada
Entomological Society of Ontario
Environment & Energy Publishing
E-Periodica Journals
Infoscience: EPF Lausanne
Equinox Publishing Journals
Freely Accessible Japanese Titles
ERIC
ERIC - Full Text Only (Discovery)
EricData 高等教育知識庫
Erudit Global AllJournals
Erudit Global Collection Culturelle - Cultural Collection
Erudit Open Access Journals
Executive Sciences Institute Publications
EStatement Studies
eTG complete Therapeutic Guidelines
ETH E-Collection
EuDML: The European Digital Mathematics Library
Eureka
EUR-Lex
EMIS University
European Commission - Economic and Financial Affairs
European Commission Environment Publications
European Commission Research Publications

European Commission-European Economy-Occasional Papers
European Library
Reading Europe
EMS Journals Archiv (DFG Nationallizenzen)
European Mathematical Society Books
European Mathematical Society Journals
esp@cenet
European Union Publications
Europeana Collections
EUREKA Academic Library
Evolutionary Ecology Research
eWIC Workshops in Computing
Exeley Publications
Expert Reviews
EZB-NALAS-00437 AAS Historical Periodicals 4 NL
Fachverlag der Verlagsgruppe Handelsblatt Zeitschriften
Factiva
African-American History Online
American History Online
American Indian History Online
Ancient and Medieval History Online
Modern World History Online
World Religions Online
F1000Research
Faulkner Advisory for IT Studies (FAITS)
FRASER publications
Federal Reserve Bank of Atlanta Publications
Federal Reserve Bank of Boston Publications
Federal Reserve Bank of Chicago Economic Research Publications
Federal Reserve Bank of Cleveland Economic Research & Data
Federal Reserve Bank of Minneapolis Economic Research Publications
Federal Reserve Bank of New York Research Publications
Federal Reserve Bank of Philadelphia Publications
Federal Reserve Bank of Richmond Research Publications
Federal Reserve Bank of San Francisco Publications
Federated Research Data Repository (FRDR)
Federation of American Societies for Experimental Biology
Films on Demand
Films on Demand Nursing Collection
Films on Demand: Nursing Video Collection

Films on Demand: World Cinema Video Collection
Financial Times
Firenze University Press Open Access Books
Firenze University Press Open Journals
Florida Atlantic University Digital Library
NOW Research Journals
AGRIS
FPUScholarWorks
Franz Steiner Verlag eLibrary
Fraunhofer-ePrints
Free E- Journals
Free Medical Journals
Freely Accessible Arts & Humanities Journals
Freely Accessible Social Science Journals
Freely Accessible Business Journals
Freely Accessible General Interest Journals
Freely Accessible Science Journals
French National Licences - Brill
French National Licences De Gruyter
French National Licences Elsevier
French National Licences IOP
French National Licences Nature
French National Licences Oxford University Press
French National Licences Royal Society of Chemistry
French National Licences Sage
French National Licences Wiley
OPUS FAU - Online publication system of Friedrich-Alexander-Universität Erlangen-Nürnberg
Frontiers in Bioscience Journal
Frontiers in
Frontline Medical Communications Journals
MEDES
Future Medicine Complete
Future Medicine Open Access
Future Science
Future Science Open Access
Academic OneFile (A&I only)
American Fur Company: America's First Business Monopoly
American History and Culture Online: Sabin Americana, 1500-1926
Archives of Sexuality and Gender: LGBTQ History and Culture Since 1940
Archives of Sexuality and Gender: LGBTQ History and Culture Since 1940, Part II

Archives Unbound<br>Contemporary Women''s Issues<br>Gale NewsVault<br>Gale Nineteenth Century Collections Online<br>Gale Nineteenth Century Collections Online: Asia and the West: Diplomacy and Cultural Exchange<br>Gale Nineteenth Century Collections Online: British Politics and Society<br>Gale Nineteenth Century Collections Online: British Theatre, Music, and Literature: High and Popular Culture<br>Gale Nineteenth Century Collections Online: Children's Literature and Childhood<br>Gale Nineteenth Century Collections Online: European Literature, 1790-1840: The Corvey Collection<br>Gale Nineteenth Century Collections Online: Mapping the World: Maps and Travel Literature<br>Gale Nineteenth Century Collections Online: Photography: The World Through the Lens<br>Gale Nineteenth Century Collections Online: Religion, Spirituality, Reform and Society<br>Gale Nineteenth Century Collections Online: Science, Technology and Medicine, 1780-1925<br>Gale Nineteenth Century Collections Online: Science, Technology, and Medicine: 1780-1925, Part II<br>Gale Nineteenth Century Collections Online: Women: Transnational Networks<br>InfoTrac Newsstand (A&I only)<br>International Herald Tribune Historical Archive 1887-2013<br>Legaltrac (A&I only)<br>Making of Modern Law: Foreign Primary Sources 1600-1970<br>Making of Modern Law: Foreign, Comparative and International Law, 1600-1926<br>Making of Modern Law: Primary Sources II, 1763–1970<br>Making of Modern Law: Primary Sources, 1620–1926<br>Making of Modern Law: Trials, 1600–1926<br>Making of the Modern World, Part III: 1890-1945<br>MLA International Bibliography<br>National Geographic Virtual Library<br>Nineteenth Century Collections Online<br>Nineteenth Century Collections Online: Asia and the West: Diplomacy and Cultural Exchange<br>Nineteenth Century Collections Online: British Politics and Society<br>Nineteenth Century Collections Online: British Theatre, Music, and Literature: High and Popular Culture<br>Nineteenth Century Collections Online: Children''s Literature and Childhood<br>Nineteenth Century Collections Online: Europe and Africa: Commerce, Christianity, Civilization, and Conquest<br>Nineteenth Century Collections Online: European Literature, 1790-1840: The Corvey Collection<br>Nineteenth Century Collections Online: Mapping the World: Maps and Travel Literature<br>Nineteenth Century Collections Online: Photography: The World Through the Lens<br>Nineteenth Century Collections Online: Religion, Spirituality, Reform and Society<br>Nineteenth Century Collections Online: Science, Technology and Medicine, 1780-1925<br>Nineteenth Century Collections Online: Science, Technology, and Medicine: 1780-1925, Part II<br>Nineteenth Century Collections Online: Women: Transnational Networks<br>Sur, 1931-1992

The Sunday Times Historical Archive, 1822-2006
19th Century UK Periodicals: Series 2 - Empire
Air & Space and Smithsonian Magazine 2011-Present
Air & Space and Smithsonian Magazine Archive (pre-2011)
Business Insights: Essentials
Business Insights: Global
Florida Newspaper Database
Gale Academic OneFile
Gale Academic OneFile Select
Gale Business: Entrepreneurship
Gale Cengage Academic ASAP
Gale Cengage Business ASAP
Gale Cengage Business ASAP International
Gale Cengage Custom Journals
Gale Cengage Custom Newspapers
Gale Cengage Economist Historical Archive
Gale Cengage Financial Times Historical Archive
Gale Cengage General Academic ASAP International
Gale Cengage General Business File ASAP International
Gale Cengage General Reference Center International
Gale Cengage Newsletters ASAP
Gale Cengage Nursing Resource Center
Gale Cengage Shakespeare Collection Periodicals
Gale Cengage The Times Digital Archive
Gale Cengage Virtual Reference Library - KERIS
Gale eBooks
Gale Eighteenth Century Collections Online I
Gale Eighteenth Century Collections Online II
Gale General OneFile
Gale Health and Wellness
Gale In Context: Biography
Gale In Context: Canada
Gale In Context: College
Gale In Context: Environmental Studies
Gale In Context: Global Issues
Gale In Context: High School
Gale In Context: Opposing Viewpoints
Gale In Context: Science
Gale In Context: U.S. History
Gale In Context: World History

Gale Interactive: Human Anatomy
Gale Literature Resource Center
Gale Literature: Dictionary of Literary Biography
Gale Literature: LitFinder
Gale Literature: Scribner Writer Series
Gale Literature: Twaynes Author Series
Gale Making of Modern Law: Legal Treatises, 1800-1926
Gale Making of the Modern World Part 1
Gale Making of the Modern World Part 2
Gale OneFile: Agriculture
Gale OneFile: Business
Gale OneFile: Communications and Mass Media
Gale OneFile: Computer Science
Gale OneFile: Contemporary Womens Issues
Gale OneFile: CPI.Q
Gale OneFile: Criminal Justice
Gale OneFile: Culinary Arts
Gale OneFile: Diversity Studies
Gale OneFile: Economics and Theory
Gale OneFile: Educator's Reference Complete
Gale OneFile: Entrepreneurship
Gale OneFile: Environmental Studies and Policy
Gale OneFile: Fine Arts
Gale OneFile: Gardening and Horticulture
Gale OneFile: Gender Studies
Gale OneFile: Health and Medicine
Gale OneFile: High School Edition
Gale OneFile: Home Improvement
Gale OneFile: Information Science
Gale OneFile: Informe Academico
Gale OneFile: Insurance and Liability
Gale OneFile: Leadership and Management
Gale OneFile: LegalTrac
Gale OneFile: Military and Intelligence
Gale OneFile: News
Gale OneFile: Nursing and Allied Health
Gale OneFile: Physical Therapy and Sports Medicine
Gale OneFile: Pop Culture Studies
Gale OneFile: Popular Magazines
Gale OneFile: Psychology

Gale OneFile: Religion and Philosophy
Gale OneFile: Science
Gale OneFile: U.S. History
Gale OneFile: Vocations and Careers
Gale OneFile: War and Terrorism
Gale OneFile: World History
Galegroup Times Literary Supplement Historical Archive
Illustrated London News Historical Archive (1842-2003)
KB+ JISC Cengage Academic OneFile 2016-2019
KB+ SHEDL Cengage The Times Digital Archive (1785-2014) 2020-2023
KB+ WHEEL Cengage The Times Digital Archive (1785-2008) 2014-2017
KB+ WHEEL Cengage The Times Digital Archive (1785-2014) 2020-2023
KB+ WHEEL CENGAGE The Times Digital Archive 1785-2010 And Rolling (2017-2020)
Literature Resource Center - Scribner Writers Module
Literature Resource Center - Twayne's Authors Module
National Geographic Magazine Archive
National Geographic Magazine Archive, 1888-1994
National Geographic Magazine Archive, 1995-Current
New York State Newspapers
Punch Historical Archive, 1841-1992
Sabin Americana: History of the Americas, 1500–1926
Smithsonian Collections Online
Telegraph Historical Archive, 1855-2000
The Independent Historical Archive
World Scholar: Latin America and the Caribbean Portal
Gallica Books Free
Gallica Periodicals
Gallica Arts
WISO - Die Datenbank für Hochschulen
wiso Fachzeitschriften Recht
wiso Fachzeitschriften Sozialwissenschaften
wiso Fachzeitschriften Technik
wiso Fachzeitschriften Wirtschaftswissenschaften
wiso journals psychology
GFMER Free Medical Journals
Geographical Association
Geological Society of America Special Papers
Lyell Collection Complete
Olms Online
Demokratizatsiya

Geosciences E-Journals
GeoScienceWorld
GeoScienceWorld eBooks Collection
KB+ JISC Collections Geoscienceworld Journals 2019
SEPM Society for Sedimentary Geology
Geotar eBooks
German Historical Institute Publications
GIGA Journal Family
GNL American Institute of Physics Digital Archive
GNL American Physical Society Digital Backfile Archive
GNL Brill Martinus Nijhoff eBooks Collection
GNL Cambridge University Press Journals Archive
GNL Central and Eastern European Online Library - CEEOL
GNL De Gruyter Berkeley Journals
GNL De Gruyter Online Journals
GNL Highwire Press American Physiological Society
GNL Karger eBooks Collection
GNL Karger Journals Archive
GNL Kluwer Law International Journals 2010
GNL Nature Archive
GNL SpringerLink Journals Archive
GNL Taylor and Francis Online Archives
GNL Thieme Connect Archive
SOFIS - Social Science Research Information System
SOLIS - Sozialwissenschaftliches Literaturinformationssystem
Sowiport
getAbstract
Ghent University Academic Bibliography
Google News Archive
KVINNSAM
Guangming Ribao (光明日报)
Griffith University DSpace
Synergies
Guilford Press
Reference Shelf
Hagley Digital Archives
HAL-SHS: Archive ouverte en Sciences de l'Homme et de la Société
HAL-SHS: Archive ouverte en Sciences de l'Homme et de la Société (Open Access)
Hamburg University Press Online-Zeitschriften
Hamburg University Press Programm

Hanz Zell Publications
Hanser eLibrary Books
Hanser Journals
Harvard Educational Publishing Group
Harvard University Library DASH
HathiTrust Digital Library
HathiTrust Digital Library Full View Outside the U.S.
HathiTrust Digital Library Full View U.S. Only
HathiTrust Digital Library Full View Worldwide
HBO Kennisbank
AMH Aged Care Companion
Australian Injectable Drugs Handbook (AIDH)
Australian Medicines Handbook
Australian Reference Content
eTG Complete
Interactive ECG
John Murtagh''s General Practice 4th Edition
John Murtagh''s Patient Education 5th Edition
John Murtagh''s Practice Tips 5th Edition
Medical Officers Handbook 8th Edition
Paediatric Pharmacopoeia 13th Edition
Paediatrics Manual
HeinOnline ABA Law Library Collection Periodicals
HeinOnline American Association of Law Libraries
HeinOnline American Bar Association Law Library
HeinOnline American Indian Law Collection
HeinOnline American Law Institute Library
HeinOnline Association of American Law Schools
HeinOnline Bar Journal Library
HeinOnline Canada Supreme Court reports
HeinOnline Criminal Justice & Criminology
HeinOnline European Centre for Minority Issues
HeinOnline Federal Register Library
HeinOnline Foreign and International Law Resources
HeinOnline Foreign Relations of the United States (FRUS)
HeinOnline GAO Reports and Comptroller General Decisions
HeinOnline Gun Regulation and Legislation in America
HeinOnline History of Bankruptcy: Taxation & Economic Reform in America, Part III
HeinOnline History Of Capital Punishment
HeinOnline History of International Law

HeinOnline Index to Foreign Legal Periodicals (IFLP)
HeinOnline Intellectual Property Law Collection
HeinOnline International Law Association Reports
HeinOnline Israel Law Reports
HeinOnline Kluwer Law International Journal Library
HeinOnline Law Journal Library
HeinOnline Legal Classics
HeinOnline New York Legal Research Library
HeinOnline Parker School of Foreign & Comparative Law Publications
HeinOnline Philip C. Jessup Library
HeinOnline Religion and the Law
HeinOnline Scottish Legal History
HeinOnline Selden Society Publications and the History of Early English Law
HeinOnline Session Laws
HeinOnline Slavery in America and the World: History, Culture & Law
HeinOnline Taxation & Economic Reform in America, Parts I & II, 1781-2010
HeinOnline Trends in Law Library Management and Technology
HeinOnline U.S. Federal Agency Documents, Decisions, and Appeals
HeinOnline U.S. International Trade Library
HeinOnline U.S. Presidential Library
HeinOnline U.S. Supreme Court Library
HeinOnline U.S. Treaties and Agreements Library
HeinOnline UN Law Collection
HeinOnline UNC Press Law Publications
HeinOnline Women and the Law (Peggy)
HeinOnline World Constitutions Illustrated
HeinOnline World Treaty Library
HeinOnline World Trials Library
Hellenic Academic Libraries Link
Apothesis
Hemispheric Institute of Performance and Politics Publications
Szold Institute - Publications - פרסומים - סאלד מכון
Szold Institute - Research Tools - מחקר כלי - סאלד מכון
Henry Stewart Talks
Henry Stewart Talks Biomedical & Life Sciences Collection
Henry Stewart Talks Business & Management Collection
American Heart Association
American Society For Horticultural Science
American Society for Nutrition
American Society of Tropical Medicine and Hygiene

Highwire Press AlphaMed Press
Highwire Press American Academy of Family Physicians
Highwire Press American Academy of Orthopaedic Surgeons
Highwire Press American Academy of Psychiatry and the Law
Highwire Press American Animal Hospital Association
Highwire Press American Association for Cancer Research
Highwire Press American Association for Clinical Chemistry
Highwire Press American Association of Critical-Care Nurses
Highwire Press American Association of Petroleum Geologists
Highwire Press American Board of Family Practice
Highwire Press American Dental Association
Highwire Press American Diabetes Association
Highwire Press American Journal of Science
Highwire Press American Osteopathic Association
Highwire Press American Podiatric Medical Association
Highwire Press American Registry of Professional Animal Scientists
Highwire Press American Society for Biochemistry and Molecular Biology
Highwire Press American Society for Clinical Pathology
Highwire Press American Society for Enology and Viticulture
Highwire Press American Society for Investigative Pathology
Highwire Press American Society for Microbiology
Highwire Press American Society for Pharmacology and Experimental Therapeutics
Highwire Press American Society of Health System Pharmacists
Highwire Press American Society of Nephrology
Highwire Press American Society of Neuroradiology
Highwire Press American Society of Plant Biologists
Highwire Press American Society of Radiologic Technologists
Highwire Press Annals of Family Medicine
Highwire Press Association of Clinical Scientists
Highwire Press Botanical Society of America
Highwire Press British Medical Journal Publishing Group
Highwire Press British Veterinary Association
Highwire Press Cleveland Clinic
Highwire Press Cold Spring Harbor Laboratory Press
Highwire Press College of Family Physicians of Canada
Highwire Press Company of Biologists
Highwire Press Dowden Health Media, Inc.
Highwire Press Environmental and Engineering Geophysical Society
Highwire Press European Association for Cardio-thoracic Surgery
Highwire Press European Respiratory Society

Highwire Press Ferrata Storti Foundation
Highwire Press Free
Highwire Press Fungal Biodiversity Centre (CBS)
Highwire Press Genetics Society of America
Highwire Press Harborside Press
Highwire Press International and American Associations of Dental Research
Highwire Press International Institute of Anticancer Research
Highwire Press Journal of Rheumatology Publishing Company Limited
Highwire Press Marine Biological Laboratory
Highwire Press Marshfield Clinic
Highwire Press Massachusetts Medical Society
Highwire Press Mineralogical Society of Great Britain and Ireland
Highwire Press Multimed Incorporated
Highwire Press Mycological Society of America
Highwire Press National Academy of Sciences
Highwire Press Parenteral Drug Association, Inc. (PDA)
Highwire Press Physiological Society
Highwire Press Poultry Science Association
Highwire Press Psychonomic Society Publications
Highwire Press Rockefeller University Press
Highwire Press Royal College of Psychiatrists
Highwire Press Seismological Society of America
Highwire Press Society for Experimental Biology and Medicine
Highwire Press Society for Leukocyte Biology
Highwire Press Society for Neuroscience
Highwire Press Society for Reproduction and Fertility
Highwire Press Society for the Study of Reproduction
Highwire Press Society of Economic Geologists, Inc.
Highwire Press Society of Interventional Radiology
Highwire Press Society of Nuclear Medicine
Highwire Press Soil and Water Conservation Society
Highwire Press Telos Press
Highwire Press University of Wisconsin Press
Highwire Press Wildlife Disease Association
Hindawi Publishing eBooks
Hindawi Publishing Journals
History of Earth Sciences Society
H-Net Reviews
Hobart and William Smith Colleges LibGuides
Hogrefe Journals

Hollywood Reporter
Hong Kong Journals Online
Hong Kong University Press
Human Kinetics
Huntington Digital Library
Hyper Article en Ligne (HAL)
Hyper Article en Ligne (HAL) (Open Access)
IHS Standards Expert
info4education (IHS Standards)
IBFD Online Publications
IBIMA Publishing Journals
IBISWorld
IBISWorld
IBISWorld Australia Industry Reports (ANZSIC)
IBISWorld Australian Industry Research Reports
IBISWorld Global Industry Reports
IBISWorld Global Industry Research
IBISWorld Industry Market Research
IBISWorld UK
IBISWorld with Global Reports
ICE Virtual Library Archive - Proceedings Collection
ICE Virtual Library Books
ICE Virtual Library Journals
KB+ Institution of Civil Engineers Publishing: JISC Collections:Virtual Library Archive (1836-2001)
KB+ JISC Collections Institution Of Civil Engineers Engineering Journals 2015
KB+ JISC Collections Institution Of Civil Engineers Engineering Journals 2017
KB+ JISC Collections Institution Of Civil Engineers Journals 2015
KB+ JISC Collections Institution Of Civil Engineers Journals 2017
ICLR Online
Inter-university Consortium for Political and Social Research
Idealonline online kütüphane - Ideal Cultural Publications
Idealonline online kütüphane - Journals
Idealonline online kütüphane - Medicine Books
Idealonline online kütüphane - Popular Magazines
Idealonline online kütüphane - Proceedings and Gift
Idunn.no
KB+ BIBSAM Universitesforlaget Idunn.no Journals 2013-2015
IEEE All-Society Periodicals Package (ASPP) 1998-Present
IEEE All-Society Periodicals Package (ASPP) 2005-Present
IEEE All-Society Periodicals Package (ASPP) 2010-Present

IEEE Articles On Demand
IEEE Communications Society
IEEE Computer Society Digital Library Journals
IEEE Computer Society Digital Library Proceedings
IEEE Conference Proceedings Archive
IEEE Elearning Library
IEEE Electronic Library (IEL)
IEEE Electronic Library (IEL) Conference Proceedings
IEEE Electronic Library (IEL) Journals
IEEE Journals Archive 1884-1954
IEEE Journals Archive 1884–1999
IEEE Journals Archive 1955–1964
IEEE Journals Archive 1965–1974
IEEE Journals Archive 1975–1984
IEEE Journals Archive 1985–1994
IEEE Journals Archive 1995–1999
IEEE Proceedings Order Plans (POP All) 1998-Present
IEEE Proceedings Order Plans (POP All) 2005-Present
IEEE Proceedings Order Plans (POP) 1998-Present
IEEE Proceedings Order Plans (POP) 2005-Present
IEEE Proceedings Order Plans POP 2010 – Present
IEEE Proceedings Order Plans POP All 2010 – Present
IEEE Spectrum Online
IEEE STEM 10 PLUS Journals Collection
IEEE STEM 25 PLUS Journals Collection
IEEE STEM 45 PLUS Journals Collection
IEEE Xplore All Conference Proceedings
IEEE Xplore All Conference Series
IEEE Xplore All Courses
IEEE Xplore All eBooks
IEEE Xplore All Journals
IEEE Xplore All Standards
IEEE Xplore Bell Labs Technical Journal
IEEE Xplore Communications Library
IEEE Xplore Computing Library
IEEE Xplore Conference Library Plus
IEEE Xplore Enterprise
IEEE Xplore IBM Journal of Research and Development
IEEE Xplore Journals Library Plus
IEEE Xplore MIT Press eBooks 2012

IEEE Xplore MIT Press eBooks 2012 & Prior
IEEE Xplore MIT Press eBooks 2013
IEEE Xplore MIT Press eBooks 2014
IEEE Xplore MIT Press eBooks 2015
IEEE Xplore MIT Press eBooks 2016
IEEE Xplore MIT Press eBooks Library—Computing & Engineering Collection
IEEE Xplore MIT Press Journals Library: Computing & Engineering Collection
IEEE Xplore Morgan & Claypool Synthesis eBooks Library
IEEE Xplore Morgan & Claypool Synthesis eBooks Library Collection Eight
IEEE Xplore Morgan & Claypool Synthesis eBooks Library Collection Five
IEEE Xplore Morgan & Claypool Synthesis eBooks Library Collection Four
IEEE Xplore Morgan & Claypool Synthesis eBooks Library Collection One
IEEE Xplore Morgan & Claypool Synthesis eBooks Library Collection Seven
IEEE Xplore Morgan & Claypool Synthesis eBooks Library Collection Six
IEEE Xplore Morgan & Claypool Synthesis eBooks Library Collection Three
IEEE Xplore Morgan & Claypool Synthesis eBooks Library Collection Two
IEEE Xplore Morgan & Claypool Synthesis eBooks Library Computer & Information Science Collection Eight
IEEE Xplore Morgan & Claypool Synthesis eBooks Library Computer & Information Science Collection Five
IEEE Xplore Morgan & Claypool Synthesis eBooks Library Computer & Information Science Collection Four
IEEE Xplore Morgan & Claypool Synthesis Ebooks Library Computer & Information Science Collection Nine
IEEE Xplore Morgan & Claypool Synthesis eBooks Library Computer & Information Science Collection One
IEEE Xplore Morgan & Claypool Synthesis eBooks Library Computer & Information Science Collection Seven
IEEE Xplore Morgan & Claypool Synthesis eBooks Library Computer & Information Science Collection Six
IEEE Xplore Morgan & Claypool Synthesis eBooks Library Computer & Information Science Collection Ten
IEEE Xplore Morgan & Claypool Synthesis eBooks Library Computer & Information Science Collection Three
IEEE Xplore Morgan & Claypool Synthesis eBooks Library Computer & Information Science Collection Two
IEEE Xplore Now Publishers Foundations and Trends Technology eBooks Library Engineering Collection 2018
IEEE Xplore Open Access Journals
IEEE Xplore POP ALL
IEEE Xplore Power & Energy Library
IEEE Xplore SMPTE Conferences
IEEE Xplore SMPTE Digital Library
IEEE Xplore SMPTE Journals
IEEE Xplore Wiley eBooks Library (IEEE Xplore)
IEEE Xplore Wiley Telecommunications eBooks 2018
IEEE Xplore Mit Press Ebooks 2017
IEEE-Wiley eBooks 2010 & Prior (IEEE Xplore)
IEEE-Wiley eBooks 2010 (IEEE Xplore)
IEEE-Wiley eBooks 2011 (IEEE Xplore)
IEEE-Wiley eBooks 2012 (IEEE Xplore)

IEEE-Wiley eBooks 2013 (IEEE Xplore)
IEEE-Wiley eBooks 2014 (IEEE Xplore)
IEEE-Wiley eBooks 2015 (IEEE Xplore)
IEEE-Wiley eBooks 2016 (IEEE Xplore)
IEEE-Wiley eBooks 2017 (IEEE Xplore)
IEEE-Wiley eBooks 2018 (IEEE Xplore)
MIT Press eBooks Library: Computing & Engineering Collection
Proceedings of the IEEE
IET Digital Library
IET Digital Library Ebooks
IET Digital Library Open Access
IG Publishing Siam
iG Publishing: A & C Black Publishers Ltd.
iG Publishing: American Library Association
iG Publishing: American Management Association
iG Publishing: Amsterdam University Press
iG Publishing: Anmol Publications
iG Publishing: ASM International
iG Publishing: Berghahn Books
iG Publishing: Berrett-Koehler Publishers, Inc.
iG Publishing: British Computer Society
iG Publishing: Business Expert Press
iG Publishing: Columbia University Press
iG Publishing: F.A. Davis Company
iG Publishing: Global Professional Publishing Ltd.
iG Publishing: Hart Publishing Ltd.
iG Publishing: Institute of Southeast Asian Studies
iG Publishing: International Engineering Consortium
iG Publishing: Kogan Page Ltd.
iG Publishing: Liverpool University Press
iG Publishing: Manchester University Press
iG Publishing: Maney Publishing
iG Publishing: Math Solutions Publications
iG Publishing: Momentum Press
iG Publishing: Multi-Science
iG Publishing: Nordic Institute of Asian Studies
iG Publishing: Princeton University Press
iG Publishing: Quintessence Publishing
iG Publishing: Radcliffe Publishing
iG Publishing: Royal Institute of British Architects

iG Publishing: Springer Publishing Company
iG Publishing: University of California Press
iG Publishing: University of Chicago Press
iG Publishing: University of Hawaii Press
Igaku Shoin Journals (医学書院雑誌)
IGDC Bibliographic Database - מאגר לחקר ההזדקנות
IGI Global Gateway
IGI Global infosci journals-communications and social science 2017
IGI Global InfoSci-Books
IGI Global infosci-books - copyright 2000
IGI Global infosci-books - copyright 2001
IGI Global infosci-books - copyright 2002
IGI Global infosci-books - copyright 2003
IGI Global infosci-books - copyright 2004
IGI Global infosci-books - copyright 2005
IGI Global infosci-books - copyright 2006
IGI Global infosci-books - copyright 2007
IGI Global infosci-books - copyright 2008
IGI Global infosci-books - copyright 2009
IGI Global infosci-books - copyright 2010
IGI Global infosci-books - copyright 2011
IGI Global infosci-books - copyright 2012
IGI Global infosci-books - copyright 2013
IGI Global infosci-books - copyright 2014
IGI Global infosci-books - copyright 2015
IGI Global infosci-books - copyright 2016
IGI Global infosci-books - copyright 2017
IGI Global infosci-books - copyright 2018
IGI Global infosci-books - copyright 2019
IGI Global infosci-business 2000 - 2013
IGI Global infosci-business 2014
IGI Global infosci-business 2015
IGI Global infosci-business 2016
IGI Global infosci-business 2017
IGI Global infosci-business 2018
IGI Global infosci-business 2019
IGI Global infosci-business administration and management technologies 2000 - 2013
IGI Global infosci-business administration and management technologies 2014
IGI Global infosci-business administration and management technologies 2015
IGI Global infosci-business administration and management technologies 2016

IGI Global infosci-business administration and management technologies 2017
IGI Global infosci-business administration and management technologies 2018
IGI Global infosci-business and enterprises collection 2016
IGI Global infosci-business and management collection 2016
IGI Global infosci-communications social science and healthcare 2000 - 2013
IGI Global infosci-communications social science and healthcare 2014
IGI Global infosci-communications social science and healthcare 2015
IGI Global infosci-communications social science and healthcare 2016
IGI Global infosci-communications social science and healthcare 2017
IGI Global infosci-communications social science and healthcare 2018
IGI Global infosci-computer science 2000 - 2013
IGI Global infosci-computer science 2014
IGI Global infosci-computer science 2015
IGI Global infosci-computer science 2016
IGI Global infosci-computer science 2017
IGI Global infosci-computer science 2018
IGI Global infosci-computer science collection 2016
IGI Global infosci-education and leadership collection 2016
IGI Global infosci-education-2000 - 2013
IGI Global infosci-education-2014
IGI Global infosci-education-2015
IGI Global infosci-education-2016
IGI Global infosci-education-2017
IGI Global infosci-education-2018
IGI Global infosci-engineering 2000 - 2013
IGI Global infosci-engineering 2014
IGI Global infosci-engineering 2015
IGI Global infosci-engineering 2016
IGI Global infosci-engineering 2017
IGI Global infosci-engineering collection 2016
IGI Global infosci-environmental science 2000 - 2013
IGI Global infosci-environmental science 2014
IGI Global infosci-environmental science 2015
IGI Global infosci-environmental science 2016
IGI Global infosci-environmental science 2017
IGI Global infosci-environmental science 2018
IGI Global infosci-environmental science collection 2016
IGI Global infosci-government 2000 - 2013
IGI Global infosci-government 2014
IGI Global infosci-government 2015

IGI Global infosci-government 2016
IGI Global infosci-government 2017
IGI Global InfoSci-Journals
IGI Global infosci-journals - copyright 2000
IGI Global infosci-journals - copyright 2001
IGI Global infosci-journals - copyright 2002
IGI Global infosci-journals - copyright 2003
IGI Global infosci-journals - copyright 2004
IGI Global infosci-journals - copyright 2005
IGI Global infosci-journals - copyright 2006
IGI Global infosci-journals - copyright 2007
IGI Global infosci-journals - copyright 2008
IGI Global infosci-journals - copyright 2009
IGI Global infosci-journals - copyright 2010
IGI Global infosci-journals - copyright 2011
IGI Global infosci-journals - copyright 2012
IGI Global infosci-journals - copyright 2013
IGI Global infosci-journals - copyright 2014
IGI Global infosci-journals - copyright 2015
IGI Global infosci-journals - copyright 2016
IGI Global infosci-journals - copyright 2017
IGI Global infosci-journals - copyright 2018
IGI Global infosci-library and information science 2000 - 2013
IGI Global infosci-library and information science 2014
IGI Global infosci-library and information science 2015
IGI Global infosci-library and information science 2016
IGI Global infosci-library and information science 2017
IGI Global infosci-library and information science 2018
IGI Global infosci-library collection 2016
IGI Global infosci-library science information studies and education 2000 - 2013
IGI Global infosci-library science information studies and education 2014
IGI Global infosci-library science information studies and education 2015
IGI Global infosci-library science information studies and education 2016
IGI Global infosci-library science information studies and education 2017
IGI Global infosci-library science information studies and education 2018
IGI Global infosci-media and communication collection 2016
IGI Global infosci-media and communications 2000 - 2013
IGI Global infosci-media and communications 2014
IGI Global infosci-media and communications 2015
IGI Global infosci-media and communications 2016

IGI Global infosci-media and communications 2017
IGI Global infosci-media and communications 2018
IGI Global infosci-medical - 2000 - 2010
IGI Global infosci-medical - 2011
IGI Global infosci-medical - 2012
IGI Global infosci-medical - 2013
IGI Global infosci-medical - 2014
IGI Global infosci-medical - 2015
IGI Global infosci-medical - 2016
IGI Global infosci-medical - 2017
IGI Global infosci-medical and healthcare collection 2016
IGI Global infosci-online teaching collection 2016
IGI Global infosci-science engineering and information technology 2000 - 2013
IGI Global infosci-science engineering and information technology 2014
IGI Global infosci-science engineering and information technology 2015
IGI Global infosci-science engineering and information technology 2016
IGI Global infosci-science engineering and information technology 2017
IGI Global infosci-science engineering and information technology 2018
IGI Global infosci-security and forensic collection 2016
IGI Global infosci-security and forensics 2000 - 2013
IGI Global infosci-security and forensics 2014
IGI Global infosci-security and forensics 2015
IGI Global infosci-security and forensics 2016
IGI Global infosci-security and forensics 2017
IGI Global infosci-security and forensics 2018
IGI Global infosci-social science 2000 - 2013
IGI Global infosci-social science 2014
IGI Global infosci-social science 2015
IGI Global infosci-social science 2016
IGI Global infosci-social science 2017
IGI Global infosci-social sciences collection 2016
InfoSci-Government and Law 2018
InfoSci-Medical, Healthcare, and Life Sciences 2018
InfoSci-Science and Engineering 2018
InfoSci-Social Sciences and Humanities 2018
InfoSci-Tec Adoption,Ethics,Human Computer Interaction eJournal Coll 2017
IHP - Index to Hebrew Periodicals - בעברית למאמרים חיפה מפתח
IHP - מפתח חיפה לכתבי עת אקדמיים ומחקריים
IHP - מפתח חיפה לכתבי עת מקצועיים
IHP - מפתח חיפה לכתבי עת שפיטים

IMD World Competitiveness Online
Risk.net
Inderscience Publishers
Indian Academy of Sciences
Digital Library of the Commons
Indiana University Mathematics Journal
Society for Ethnomusicology (SEM)
IUScholarWorks Journals
Ancient and Medieval History Online
Films on Demand
Films on Demand & Access Video on Demand (NC Live only)
Films On Demand Allied Health Video Collection
Films on Demand Archival Films & Newsreels Video Collection
Films On Demand Business & Economics Video Collection
Films On Demand Career & Technical Education Video Package
Films on Demand Careers & Job Search Video Collection
Films on Demand Family & Consumer Sciences Video Collection
Films on Demand Guidance & Counseling Video Collection
Films on Demand Health Video Collection
Films on Demand Humanities & Social Sciences Video Collection
Films On Demand Master Academic Video Package
Films on Demand Science & Mathematics Video Collection
Films on Demand Technical Education Video Collection
Films On Demand Veterinary Video Collection
Informa Healthcare Books
A+ Education (A&I content for Discovery)
Aboriginal and Torres Strait Islander Health Bibliography
AGIS Plus Text (A&I content for Discovery)
Agriculture and Natural Resources Index Archive (ANR-Index Archive)
Agriculture and Natural Resources Research
Agriculture and Natural Resources Research Archive (ANR-Research Archive)
AHRR: Australian Historic Records Register
AIM Management & Training Database (AIMMAT)
APEC Literature Database (APECLIT)
Archives and Records Management Literature in Australia and New Zealand (ARLIT)
ASIAN Resources: A Select Directory of Databases
Austguide
Australasian Medical Index
Australasian Military History Database (MIHILIST)
Australian Accounting & Taxation Database (AATD)

Australian Architecture Database
Australian Building Construction and Engineering Database (BUILD)
Australian Criminology Database
Australian Education Index
Australian Engineering Database (ENGINE)
Australian Family & Society Abstracts Database
Australian Federal Police Digest
Australian Heritage Bibliography (AHB)
Australian Industrial Relations Database (IREL)
Australian Interlibrary Resource Sharing Directory
Australian Library & Information Science Abstracts
Australian Nuclear Science & Technology Information (ANSTI)
Australian Public Affairs Full Text (A&I content for Discovery)
Australian Sport Database
Australian Taxation Abstracts Database (TAXABS)
Australian Tourism Index
Australian Transport Index (ATRI)
Australia''s National Geosciences, Minerals and Petroleum Reference Database (AESIS)
Australia''s Natural Resources Database (STREAMLINE)
Chronology of Australian Historic & Current Events (AUSCHRON)
Database on English Language Teaching for Adults in Australasia
Drug Database
EDGE
Edu TV
Environmental Abstracts (EVA)
Far North Queensland Collection (FNQ)
Giblin Working Papers Database (GIBLIN)
Great Barrier Reef Marine Park Province Database (REEF)
HIV/AIDS Database (HIVA)
Indigenous Australia
Indigenous Studies Bibliography (AIATSIS)
Informit A+ Education
Informit AGIS Plus Text
Informit Australian Public Affairs Full Text
Informit Business Collection
Informit Engineering Collection
Informit Families and Society Collection
Informit Health Collection
Informit Humanities & Social Sciences Collection
Informit Indigenous Collection

Informit Literature and Culture Collection
Informit New Zealand Collection
INTAN MAS
International Taxation Issues Database (INTAX)
Malaysia/Singapore/Brunei/ASEAN Database (BERITA)
Management and Environment Information (MEDGE)
MediaScan
Multicultural Australia and Immigration Studies
OMN Training Resources Database, Distributors (OMNDIST)
OMN Training Resources Database, Resources (OMNRES)
Rural and Remote Health Database (RURAL)
SCANfile
Science And Geography Education (SAGE)
Searchable Networked Intellectual Property Electronic Resource (SNIPER)
Serials in Australian Libraries (SIAL)
The Australian Chronicles: An Index to Sources of Australian Biography (CHRONICLES)
Threatened Species in Australia: A Select Bibliography (ENDANGER)
TVNews
Victorian Audit Library Information Service Database (VALISE)
Informs
INFORMS Journals Archive (1952-1997)
Medline
IngentaConnect (index/abstract)
IngentaConnect Bentham Science Publishing
IngentaConnect Bloomsbury Journals
IngentaConnect Felix Meiner Verlag
IngentaConnect Helmut Buske Verlag
IngentaConnect Henry Stewart Publications
IngentaConnect Intellect
IngentaConnect Journals
IngentaConnect Manchester University Press
IngentaConnect The Policy Press
Pascal-Francis
Institute of Arctic and Alpine Research
Food Technology
Institute of Mathematics Polish Academy of Sciences
INCE Digital Library
AAS-IOP Astronomy
AAS-IOP Astronomy Release 1
AAS-IOP Astronomy Release 2

BACON - Institute of Physics publishing IOP - COUPERIN_SCIENCE-EXTRA-COMPLETE
BACON - Institute of Physics publishing IOP - COUPERIN_STANDARD
CRKN Institute of Physics Journals Archive
CRKN Institute of Physics Journals Archive Update
CRKN Institute of Physics Journals Current
DRAA IOP Publishing Journals
Institute of Physics - Concise Physics
Institute of Physics - Expanding Physics
Institute of Physics - IOP eBooks
Institute of Physics AAS Titles
Institute of Physics Historic Archive Purchase
Institute of Physics Historic Archive Recent Purchase
Institute of Physics IOPscience extra
Institute of Physics Journals
Institute of Physics Open Access Journal Titles
Institute of Physics Sold Journal Titles
IOP Publishing CAUL Collection
IOP Publishing Concise Physics Release 1
IOP Publishing Concise Physics Release 2
IOP Publishing Concise Physics Release 3
IOP Publishing Concise Physics Release 4
IOP Publishing Concise Physics Release 5
IOP Publishing Concise Physics Release 6
IOP Publishing Expanding Physics Release 1
IOP Publishing Expanding Physics Release 2
IOP Publishing Expanding Physics Release 3
IOP Publishing Expanding Physics Release 5
IOP Publishing Expanding Physics Release 6
IOP Publishing Jordanian Public University Library System CoE Collection
IOP Publishing Physics World Discovery
IOP Science Platform
IOPscience (Open Access)
KB+ BIBSAM Institute Of Physics Journals 2014-2016
KB+ Jisc Collections Institute of Physics IOP Publishing Read & Publish agreement 2020-2023 reading list
KB+ JISC Collections Institute Of Physics IOP Science Extra 2017-2019
KB+ JISC Collections Institute Of Physics Journal Archive 1874-1998
KB+ JISC Collections Institute of Physics Journal Archive 1999-2006
KB+ JISC Collections Institute Of Physics Option 1 2015
KB+ JISC Collections Institute Of Physics Option 1 2016
KB+ JISC Collections Institute Of Physics Option 2 2015

KB+ JISC Collections Institute Of Physics Option 2 2016
KB+ JISC Institute of Physics Historical Archives
NESLi2 Institute of Physics Journals Option 1
NESLi2 Institute of Physics Journals Option 2
VIVA IOP Journals
IET 2013 Frontlist
IET 2015 Frontlist
IET Conference Publications by volume
SciTech eBooks Backlist
InTech Books Free
Intelecom Search Center
Past Masters Commons
Intellect Journals
Inter-American Development Bank
IARIA Journals
WHO/IARC Classification of Tumours
IAMCR Open Access Journals
SDI Online
International Developmental Research Center (IDRC) Digital Library
International Journal of Industrial Engineering
International Medical Press Online Journals
Balance of Payment Statistics
Direction of Trade Statistics Browser
Government Finance Statistics
IMF Books & Analytical Papers
IMF eLibrary Data
IMF Statistics
International Financial Statistics Online
International Monetary Fund (IMF)
International Monetary Fund Periodicals
World Economic Outlook Database
International Monetary Fund eLibrary
Regional Economic Outlook Reports
International Press
International Press - Pure Mathematics Journals
ISC Publications
International Society for Gerontechnology Publications
Ethnicity & disease
International Tables for Crystallography
International Union of Pure and Applied Chemistry

International Water Association Publishing - IWAP
Canadian Libraries
Internet Archive
Internet Scientific Publications
Inter-Research Science Center Journals
Institute of Physics:NESLI2:Science Extra Platform (Option 2):2012
Institute of Physics:NESLI2:Science Extra Platform (Option 2):2014
IOP Publishing Turpion Journals
IOS Press Books
IOS Press Journals
KB+ JISC Collections IOS Press 2015-2017
KB+ JISC Collections IOS Press 2018-2020
Israel Antiquities Authority
ARIEL - The Israel Review of Arts and Letters
ISTEX
Jan Szczepanski's Lists of OA-Journals. Historic Titles
IHS Jane’s Fighting Ships
IHS Jane’s Weapons: Ammunition
IHS Jane''s Aero Engines
IHS Jane''s Aircraft Component Manufacturers
IHS Jane''s airport review
IHS Jane''s All the World''s Aircraft: Unmanned
IHS Jane''s Country Risk Daily Report
IHS Jane''s Defence Industry
IHS Jane''s Defence Weekly
IHS Jane''s Flight Avionics
IHS Jane''s Helicopter Markets and Systems
IHS Jane''s Intelligence Review
IHS Jane''s International ABC Defence Directory
IHS Jane''s International Defence Review
IHS Jane''s Islamic affairs analyst
IHS Jane''s Missiles and Rockets
IHS Jane''s Navy International
IHS Jane''s Police & Homeland Security Equipment
IHS Jane''s Simulation & Training Systems
IHS Jane''s Space Systems & Industry
IHS Jane''s Terrorism & Insurgency Monitor
IHS Jane''s Terrorism Watch Report
IHS Jane''s weapons. Air launched
IHS Jane''s weapons. Infantry

IHS Jane''s weapons. Naval
IHS Jane''s Weapons: Strategic
IHS Jane''s World Air Forces
IHS Jane''s World Armies
IHS Jane''s World Defence Industry
IHS Jane''s World Navies
Jane's Amphibious and Special Forces
JTIC: Terrorism and Insurgency Centre
Heterocycles
医中誌Web
Japan Society of Applied Physics
Japan Times Archives
Journal of Clinical Orthodontics
Rambi - Index of Articles on Jewish Studies – רמבי
MeTeL Media
KB+ Jisc Collections Proquest Art and Architecture Archive 2017-2020
KB+ JISC Collections Proquest Literature Online
KB+ JISC Collections Proquest Periodicals Archive Online Collection 2 With Extension
KB+ JISC Collections Proquest Periodicals Archive Online Selection 2013-2018
John Benjamins Books
John Benjamins Journals
John Libbey EUROTEXT
Jordan Publishing Family Law Journals
Journal of Clinical Ethics
Journal of Clinical Pediatric Dentistry
Journal of Medical Insight
Journal of Philosophy
Journal of Studies on Alcohol and Drugs
Journal of Visualized Experiments
JoVE Behavior
JoVE Biochemistry
JoVE Bioengineering
JoVE Biology
JoVE Cancer Research
JoVE Chemistry
JoVE Core: Biology
JoVE Core: Social Psychology
JoVE Developmental Biology
JoVE Engineering
JoVE Environment

JoVE Genetics
JoVE Immunology and Infection
JoVE Lab Manual: Introductory Biology
JoVE Lab Manual: Introductory Chemistry
JoVE Medicine
JoVE Neuroscience
JoVE Open Access
JoVE Science Education
JoVE Science Education: Analytical Chemistry
JoVE Science Education: Basic Methods in Cellular and Molecular Biology
JoVE Science Education: Behavioral Science
JoVE Science Education: Biochemistry
JoVE Science Education: Bioengineering
JoVE Science Education: Biology I
JoVE Science Education: Biology II
JoVE Science Education: Cell Biology
JoVE Science Education: Chemical Engineering
JoVE Science Education: Cognitive Psychology
JoVE Science Education: Developmental Biology
JoVE Science Education: Developmental Psychology
JoVE Science Education: Earth Science
JoVE Science Education: Electrical Engineering
JoVE Science Education: Emergency Medicine and Critical Care
JoVE Science Education: Environmental Microbiology
JoVE Science Education: Environmental Science
JoVE Science Education: Experimental Psychology
JoVE Science Education: General Chemistry
JoVE Science Education: General Laboratory Techniques
JoVE Science Education: Genetics
JoVE Science Education: Inorganic Chemistry
JoVE Science Education: Lab Animal Research
JoVE Science Education: Lab Safety
JoVE Science Education: Mechanical Engineering
JoVE Science Education: Neuropsychology
JoVE Science Education: Neuroscience
JoVE Science Education: Nursing Skills
JoVE Science Education: Organic Chemistry
JoVE Science Education: Organic Chemistry II
JoVE Science Education: Physical Examinations I
JoVE Science Education: Physical Examinations II

JoVE Science Education: Physical Examinations III
JoVE Science Education: Physics I
JoVE Science Education: Physics II
JoVE Science Education: Sensation and Perception
JoVE Science Education: Social Psychology
JoVE Science Education: Biomedical Engineering
JoVE Science Education: Materials Engineering
JoVE Science Education: Structural Engineering
J-STAGE Free
J-STAGE Subscribe
Agricultural History Society
JSTOR 19th Century British Pamphlets
JSTOR Arts & Sciences X Current
JSTOR Arts & Sciences XI
JSTOR Arts & Sciences XII
JSTOR Arts & Sciences XIII
JSTOR Arts & Sciences XIV
JSTOR Arts & Sciences XV
JSTOR Arts and Sciences I
JSTOR Arts and Sciences II
JSTOR Arts and Sciences III
JSTOR Arts and Sciences IV
JSTOR Arts and Sciences IX
JSTOR Arts and Sciences V
JSTOR Arts and Sciences VI
JSTOR Arts and Sciences VII
JSTOR Arts and Sciences VIII
JSTOR Arts and Sciences X
JSTOR Asia Collection
JSTOR Biological Sciences Collection
JSTOR Books
JSTOR Books Archaeology
JSTOR Books Business And Economics
JSTOR Books EBA Pilot
JSTOR Books Education
JSTOR Books Film Studies
JSTOR Books History
JSTOR Books Language And Literature
JSTOR Books Law
JSTOR Books Music

JSTOR Books Open Access
JSTOR Books Philosophy
JSTOR Books Political Science
JSTOR Books Religion
JSTOR Books Sociology
JSTOR Books Spanish Collection
JSTOR Books Sustainability
JSTOR Business & Economics
JSTOR Business Collection
JSTOR Business II Collection
JSTOR Business III Collection
JSTOR Business IV Collection
JSTOR Complete
JSTOR Early Journal Content Free
Jstor Ebooks - Draa
Jstor Ebooks Biodiversity, Conservation & Ecology
Jstor Ebooks Botany
Jstor Ebooks Forestry, Ferns & Mosses
Jstor Ebooks Garden & Landscape Design
Jstor Ebooks Useful Plants
JSTOR Ecology & Botany II
JSTOR Ecology and Botany I
JSTOR Health & General Sciences
JSTOR Hebrew Journals
JSTOR Iberoamérica
JSTOR Ireland
JSTOR Jewish Studies Collection
JSTOR Journal Hosting Program
Jstor Journals Open Access
JSTOR Language & Literature Collection
JSTOR Life Sciences Collection
JSTOR Lives of Literature
JSTOR Mathematics & Statistics Legacy Collection
JSTOR Mathematics and Statistics
JSTOR Music Collection
JSTOR Music Legacy Collection
JSTOR Religion & Theology
JSTOR Security Studies
JSTOR Security Studies Extension
JSTOR Sustainability (journals and research reports)

JSTOR Unlimited Usage Book Titles
JSTOR Unlimited Usage DDA Book Titles
KB+ JISC Collections JSTOR Arts And Sciences I 2012-2015
KB+ JISC Collections JSTOR Arts And Sciences I Collections 2016-2017
KB+ JISC Collections JSTOR Arts And Sciences II 2012-2015
KB+ JISC Collections JSTOR Arts And Sciences II Collection 2016-2017
KB+ JISC Collections JSTOR Arts And Sciences Ii Collection 2017-2018
KB+ JISC Collections JSTOR Arts And Sciences III 2012-2015
KB+ JISC Collections JSTOR Arts And Sciences III Collection 2016-2017
KB+ JISC Collections JSTOR Arts And Sciences Iii Collection 2017-2018
KB+ JISC Collections JSTOR Arts And Sciences IV 2012-2015
KB+ JISC Collections JSTOR Arts And Sciences IV Collection 2016-2017
KB+ JISC Collections JSTOR Arts And Sciences Iv Collection 2017-2018
KB+ JISC Collections JSTOR Arts And Sciences IX 2012-2015
KB+ JISC Collections JSTOR Arts And Sciences IX Collection 2016-2017
KB+ JISC Collections JSTOR Arts And Sciences Ix Collection 2017-2018
KB+ JISC Collections JSTOR Arts And Sciences V 2012-2015
KB+ JISC Collections JSTOR Arts And Sciences V Collection 2016-2017
KB+ JISC Collections JSTOR Arts And Sciences VI 2012-2015
KB+ JISC Collections JSTOR Arts And Sciences VI Collection 2016-2017
KB+ JISC Collections JSTOR Arts And Sciences VII Collection 2016-2017
KB+ JISC Collections JSTOR Arts And Sciences VIII Collection 2016-2017
KB+ JISC Collections JSTOR Arts And Sciences X 2012-2015
KB+ JISC Collections JSTOR Arts And Sciences X Collection 2016-2017
KB+ JISC Collections JSTOR Arts And Sciences XI 2012-2015
KB+ JISC Collections JSTOR Arts And Sciences XI Collection 2016-2017
KB+ JISC Collections JSTOR Arts And Sciences XII 2012-2015
KB+ JISC Collections JSTOR Arts And Sciences XII Collection 2016-2017
KB+ JISC Collections JSTOR Arts And Sciences XIII Collections 2016-2017
KB+ JISC Collections JSTOR Arts And Sciences XIV Collection 2016-2017
KB+ JISC Collections JSTOR Arts And Sciences XV Collection 2016-2017
KB+ JISC Collections JSTOR Arts And Sciences Xv Collection 2017-2018
KB+ JISC Collections JSTOR Biological Sciences 2012-2015
KB+ JISC Collections JSTOR Biological Sciences Collection 2016-2017
KB+ JISC Collections JSTOR Business And Economics Collection 2016-2017
KB+ JISC Collections JSTOR Business I 2012-2015
KB+ JISC Collections JSTOR Business I Collection 2016-2017
KB+ JISC Collections JSTOR Business II 2012-2015
KB+ JISC Collections JSTOR Business II Collection 2016-2017
KB+ JISC Collections JSTOR Business III 2012-2015

KB+ JISC Collections JSTOR Business III Collection 2016-2017
KB+ JISC Collections JSTOR Business IV Collection 2016-2017
KB+ JISC Collections JSTOR Ecology And Botany 2012-2015
KB+ JISC Collections JSTOR Ecology And Botany I Collection 2016-2017
KB+ JISC Collections JSTOR Ecology And Botany II 2012-2015
KB+ JISC Collections JSTOR Ecology And Botany II Collection 2016-2017
KB+ JISC Collections JSTOR Health And General Sciences Collection 2016-2017
KB+ JISC Collections JSTOR Hebrew Collection 2015-2016
KB+ JISC Collections JSTOR Hebrew Collection 2016-2017
KB+ JISC Collections JSTOR Ireland Collection
KB+ JISC Collections JSTOR Jewish Studies Collection 2015-2016
KB+ JISC Collections JSTOR Jewish Studies Collection 2016-2017
KB+ JISC Collections JSTOR Language And Literature 2012-2015
KB+ JISC Collections JSTOR Language And Literature Collection 2016-2017
KB+ JISC Collections JSTOR Life Sciences 2012-2015
KB+ JISC Collections JSTOR Life Sciences Collection 2016-2017
KB+ JISC Collections JSTOR Mathematics And Statistics 2012-2015
KB+ JISC Collections JSTOR Mathematics And Statistics Enhanced Collection 2016-2017
KB+ JISC Collections JSTOR Mathematics And Statistics Legacy Collection 2016-2017
KB+ JISC Collections JSTOR Music 2012-2015
KB+ JISC Collections JSTOR Music Collection 2015-2016
KB+ JISC Collections JSTOR Music Enhanced Collection 2016
KB+ JISC Collections JSTOR Music Enhanced Collection 2016-2017
KB+ JISC Collections JSTOR Music Legacy Collection 2016-2017
KB+ JISC Collections JSTOR Religion And Theology Collection 2015-2016
KB+ JISC Collections JSTOR Religion And Theology Collection 2016-2017
KB+ JISC Collections Jstor Sustainability 2017-2018
Beltz Soziologie
Beltz Soziologie 2018
Beltz Soziologie Basis-Paket
Julius Beltz GmbH & Co. KG
Criterion Collection/Janus Films
MEF Collection
MEF Premium Collection
Bacon Karger Couperin Alljournals
Karger Books 2015
Karger Books 2017
Karger Books 2018-2019
Karger Books Series
Karger eBook Archive Collection German

Karger eBook Archive Collection Non-Series
Karger eBook Archive Non Serials Collection Deutsch
Karger eBook Archive Non Serials Collection English
Karger eBook Archive Serials Collection
Karger eBooks Collection
Karger eBooks Deutsch Collection
Karger Journal Archive Collection Biosciences
Karger Journal Archive Collection Internal Medicine 1
Karger Journal Archive Collection Internal Medicine 2
Karger Journal Archive Collection Neurology Neurobiology Neurosurgery
Karger Journal Archive Complete
Karger Journal Archive ORL, Ophthalmology, Dental Medicine, Obstetrics, Gynecology and Psychology, Psychiatry
Karger Journals Complete
Karger Journals Current Program
Karger Open Access Journals
KB+ BIBSAM Karger Medical And Scientific eJournals Complete 2014-2016
KB+ BIBSAM Karger Medical and Scientific Publishers 2017-2020
KB+ JISC Collections Karger Biosciences Journal Archive Collection
KB+ JISC Collections Karger Full Journal Archive Collection
KB+ JISC Collections Karger Internal Medicine I Archive Collection
KB+ JISC Collections Karger Internal Medicine Ii Archive Collection
KB+ JISC Collections Karger Medical And Scientific Full Collection 2015-2017
KB+ JISC Collections Karger Medical And Scientific Publishers Full Collection 2018-2020
KB+ JISC Collections Karger Neurology Neurobiology Neurosurgery Journal Archive
KB+ JISC Collections Karger ORL Ophthalmology Dental Medicine And Psychiatry Archive
Karger eBooks Collection 2020
SveMed+
KB+ Independent Scholarly Publishers Group - JISC Collection 2015-2017
KB+ Jisc Collections Company of Biologists Read and Publish Transitional Agreement Pilot 2020-2021
KB+ Jisc Collections European Respiratory Society European Respiratory Journal Read and Publish Transitional Agreement Pilot 2020-2021
KB+ Jisc Collections IWA Publishing IWAP Read and Publish Transitional Agreement Pilot 2020-2021
Keesing's World News Archive
Key Note Market Reports
KISTI Society
KB+ BIBSAM Kluwer Law Journals 2016-2018
KB+ BIBSAM Kluwer Law Journals 2019-2021
Kluwer Competition Law
Kluwer Law Online Journals
UpToDate
Kluwer International Encyclopedia of Laws

NARCIS
NARCIS: Datasets
Narcis: Open Access
NARCIS:Publications
AICHE/CCPS - Center for Chemical Process Safety
ASM on Knovel
Knovel Adhesives Coatings Sealants & Inks Academic
Knovel Adhesives, Coatings, Sealants & Inks Corporate
Knovel Aerospace Radar Technology Academic
Knovel Aerospace Radar Technology Corporate
Knovel ASM International Materials Collection Academic
Knovel ASM International Materials Collection Corporate
Knovel ASME Boiler and Pressure Vessel Code, Section II - Materials Academic
Knovel ASME Boiler and Pressure Vessel Code, Section II - Materials Corporate
Knovel Biochemistry, Biology & Biotechnology Academic
Knovel Biochemistry, Biology & Biotechnology Corporate
Knovel Center For Chemical Process Safety Premium Academic
Knovel Center For Chemical Process Safety Premium Corporate
Knovel Ceramics & Ceramic Engineering Academic
Knovel Ceramics & Ceramic Engineering Corporate
Knovel Chemical Resistance Database: Plastics and Elastomers Academic
Knovel Chemical Resistance Database: Plastics and Elastomers Corporate
Knovel Chemistry & Chemical Engineering Academic
Knovel Chemistry & Chemical Engineering Corporate
Knovel Civil Engineering & Construction Materials Academic
Knovel Civil Engineering & Construction Materials Corporate
Knovel Complete Academic
Knovel Complete Corporate
Knovel Composites Academic
Knovel Composites Corporate
Knovel Computer Hardware Engineering Academic
Knovel Computer Hardware Engineering Corporate
Knovel DIPPR Project 801 Academic
Knovel DIPPR Project 801 Corporate
Knovel Earth Sciences Academic
Knovel Earth Sciences Corporate
Knovel Electrical & Power Engineering Academic
Knovel Electrical & Power Engineering Corporate
Knovel Electronics & Semiconductors Academic
Knovel Electronics & Semiconductors Corporate

Knovel Engineering Management & Leadership Academic
Knovel Engineering Management & Leadership Corporate
Knovel Environment & Environmental Engineering Academic
Knovel Environment & Environmental Engineering Corporate
Knovel Fire Protection Engineering & Emergency Response Academic
Knovel Fire Protection Engineering & Emergency Response Corporate
Knovel Food Science Academic
Knovel Food Science Corporate
Knovel General Engineering & Project Administration Academic
Knovel General Engineering & Project Administration Corporate
Knovel Industrial Engineering & Operations Management Academic
Knovel Industrial Engineering & Operations Management Corporate
Knovel Knovel Polymer Matrix Composites Database Academic
Knovel Knovel Polymer Matrix Composites Database Corporate
Knovel Manufacturing Engineering Academic
Knovel Manufacturing Engineering Corporate
Knovel Marine Engineering Academic
Knovel Marine Engineering Corporate
Knovel Mechanics & Mechanical Engineering Academic
Knovel Mechanics & Mechanical Engineering Corporate
Knovel Metals & Metallurgy Academic
Knovel Metals & Metallurgy Corporate
Knovel Mining Engineering and Extractive Metallurgy Academic
Knovel Mining Engineering and Extractive Metallurgy Corporate
Knovel Nanotechnology Academic
Knovel Nanotechnology Corporate
Knovel Nondestructive Testing Academic
Knovel Nondestructive Testing Corporate
Knovel Oil & Gas Engineering Academic
Knovel Oil & Gas Engineering Corporate
Knovel Optics and Photonics Academic
Knovel Optics and Photonics Corporate
Knovel Pharmaceuticals Cosmetics & Toiletries Academic
Knovel Pharmaceuticals, Cosmetics & Toiletries Corporate
Knovel Plastics & Rubber Academic
Knovel Plastics & Rubber Corporate
Knovel Premium Titles Academic
Knovel Premium Titles Corporate
Knovel Process Design Control and Automation Academic
Knovel Process Design Control and Automation Corporate

Knovel Safety & Industrial Hygiene Academic
Knovel Safety & Industrial Hygiene Corporate
Knovel Software Engineering Academic
Knovel Software Engineering Corporate
Knovel Sustainable Energy and Development Academic
Knovel Sustainable Energy and Development Corporate
Knovel Textiles Academic
Knovel Textiles Corporate
Knovel Transportation Engineering Academic
Knovel Transportation Engineering Corporate
Knovel Welding Engineering & Materials Joining Academic
Knovel Welding Engineering & Materials Joining Corporate
Knovel, Academic, AICHE/CCPS - Center for Chemical Process Safety
Knovel, Academic, ASM International Materials Collection
Knovel, Academic, ASME Boiler and Pressure Vessel Code, Section II - Materials
Knovel, Academic, Chemical Resistance Database: Plastics and Elastomers
Knovel, Academic, DIPPR Project 801
Knovel, Academic, Knovel Polymer Matrix Composites Database
Knovel, Corporate, Adhesives, Coatings, Sealants & Inks
Knovel, Corporate, Aerospace & Radar Technology
Knovel, Corporate, AICHE/CCPS - Center for Chemical Process Safety
Knovel, Corporate, ASM International Materials Collection
Knovel, Corporate, ASME Boiler and Pressure Vessel Code, Section II - Materials
Knovel, Corporate, Biochemistry, Biology & Biotechnology
Knovel, Corporate, Ceramics & Ceramic Engineering
Knovel, Corporate, Chemical Resistance Database: Plastics and Elastomers
Knovel, Corporate, Chemistry & Chemical Engineering
Knovel, Corporate, Civil Engineering & Construction Materials
Knovel, Corporate, Composites
Knovel, Corporate, Computer Hardware Engineering
Knovel, Corporate, DIPPR Project 801
Knovel, Corporate, Earth Sciences
Knovel, Corporate, Electrical & Power Engineering
Knovel, Corporate, Electronics & Semiconductors
Knovel, Corporate, Engineering Management & Leadership
Knovel, Corporate, Environment & Environmental Engineering
Knovel, Corporate, Fire Protection Engineering & Emergency Response
Knovel, Corporate, Food Science
Knovel, Corporate, General Engineering & Project Administration
Knovel, Corporate, Industrial Engineering & Operations Management

Knovel, Corporate, Knovel Polymer Matrix Composites Database
New Zealand Index
Knowledge Unlatched Pilot Collection
Knowledge Unlatched Round 2
KU Select 2016
KU Select 2017
KU Select 2018 HSS Books Collection
KU Select 2018 STEM Books Collection
Delpher Boeken Basiscollectie (1781-1800)
Korea Information Science Society (KISS)
Korea Institute of Science and Technology Information (한국과학기술정보연구원)
Korea Science Citation Index
Korea Scholar
KoreaMed Synapse
KoreaMed Open Access
Korean Studies Information Service System (KISS) Journals - English
KoreaScience
Kotar - CET כותר - מטח
Kotar Journals - CET -כותר כתבי עת - מטח
New Zealand Geographic
LIBRIS
Kwantlen Digital Commons
Kyobo Scholar Journals
Kyoto University Repository
Lancet Medical Journals
Latin America and Caribbean social science virtual library: CLACSO
Latin American News Digest
Archives Historiques
Leadership Library - Yellow Books
Led on Line
Leeds Beckett University ePrints
PsychOpen
Leiden University Repository
Lenus The Irish Health Repository (Summon)
Leo S Olschki Publications
Publications of the Lepidopterists' Society
Lexis Advance Canada
Lexis Advance Quicklaw – add on Employment-Family-Real Estate
Lexis Advance Quicklaw – add on French
Lexis Advance Quicklaw – add on Securities-Corp-Comm

Lexis Advance Quicklaw Core
Lexis Advance Quicklaw Plus
LexisNexis Academic: Landmark Cases
LexisNexis Academic: Law Reviews
LexisNexis JurisClasseur
Nexis Advance UK
Nexis UK
Nexis Uni
Lexxion Zeitschriften
American Memory Images
Library of Congress Web Archives (LCWA)
Performing Arts Encyclopedia
Library Publishing Media Open Access Journals
Library Stack
LIBRAweb
LibriVox
Lincoln University Research Archive
Living Heritage Tikaka Tōku Iho
Linda Hall Digital Library
Lippincott Williams & Wilkins
Bacon Lippincott Williams And Wilkins Couperin Premier
Lirias (KU Leuven Association)
KB+ JISC Collections Liverpool University Press 2016-2018
Liverpool University Press
LOCKSS
LOCUS - SIAM's Online Journal Archive
London Review of Books
LSHTM Research Library
Longwoods Publishing
LORY (Lucerne Open Repository)
Loughborough University Institutional Repository
Loughborough University Institutional Repository Open Access
LSE PhD theses
LSE Research Online
Lucius & Lucius Zeitschriften
LUISS IR -- LUISSearch
LUISS IR -- LUISSThesis
Lynne Rienner Publishers Journals
Digital Commons @ Macalester College
MADOC Publikationsserver

KB+ BIBSAM Mark Allen Healthcare Journals 2016-2018
MA Education Intered
MA Education Intered Extra
MA Healthcare Complete
MA Healthcare Health Professionals
MA Healthcare InterMid
MA Healthcare InterNurse
MA Healthcare InterUK Vet
Magnes Press - The Hebrew University - מאגנס - העברית האוניברסיטה
Magnolia Press Serials
Maine Memory Network
UK Institutional Repository
Manchester University Press Journals - Open Access
Manchester University Research Explorer
MCCCD LibGuide
Transnational Dispute Management
Maruzen eBook Library
Maruzen eBook Library - Iwanami Subscription
EZB: Mary Ann Liebert
KB+ BIBSAM Mary Ann Liebert Publishers Journals 2014-2016
KB+ BIBSAM Mary Ann Liebert Publishers Journals 2017-2019
LiebertOnline Archives (Eira)
Mary Ann Liebert Online - Open Access
Mary Ann Liebert Publishers Journals
NERL Mary Ann Liebert Publishers Journals
Mary Ann Liebert Legacy Content
Mary Immaculate Research Repository Dspace
New England Journal of Medicine Archive
McFarland Journals
eScholarship@McGill
McGraw Hill Access Anesthesiology
McGraw Hill Access Pediatrics
McGraw-Hill AccessEmergency Medicine
McGraw-Hill AccessJAMAevidence
McGraw-Hill AccessMedicine
McGraw-Hill AccessPharmacy
McGraw-Hill AccessPhysiotherapy
McGraw-Hill AccessSurgery
McGraw-Hill's AccessEngineering
Access Medicine - Lange Educational Library

AccessObGyn
F.A. Davis PT Collection
Harrison Medicina
Harrisons Online
HemOnc Collection
McGraw Hill Education eBook Library - Medical 2015 Update Collection
McGraw-Hill AccessNeurology
McGraw-Hill OMMBID
McGraw-Hill Professional eBook Library - Medical: Handbooks
Murtagh Collection
McGraw Hill Ebook Library AP Study Guides
McGraw Hill Ebook Library Business Collection
McGraw Hill Ebook Library Business Skills
McGraw Hill Ebook Library Career Advice
McGraw Hill Ebook Library Computing
McGraw Hill Ebook Library DEMYSTIFIED Self-Teaching Guide: Education
McGraw Hill Ebook Library DEMYSTIFIED Self-Teaching Guide: Technical and Science
McGraw Hill Ebook Library DEMYSTIFIED Self-Teaching Guides: Business and Economics
McGraw Hill Ebook Library DEMYSTIFIED Self-Teaching Guides:Health Professions
McGraw Hill Ebook Library Entrepreneurship & Small Business Management
McGraw Hill Ebook Library Finance & Investing
McGraw Hill Ebook Library Human Resources & Training
McGraw Hill Ebook Library Leadership & Management
McGraw Hill Ebook Library Medical Collection
McGraw Hill Ebook Library Nursing
McGraw Hill Ebook Library Open University Press Collection
McGraw Hill Ebook Library Primary Care
McGraw Hill Ebook Library Process Management
McGraw Hill Ebook Library Professional Nursing
McGraw Hill Ebook Library Real Estate
McGraw Hill Ebook Library Sales & Marketing
McGraw Hill Ebook Library Schaum's Course Outlines
McGraw Hill Ebook Library School & Career Exams
McGraw Hill Ebook Library Student Study Aids Collection
McGraw Hill Ebook Library Understanding Public Health
McGraw Hill Ebook Library USMLE First Aid
McGraw-Hill's eBook Library First Aid Test Prep
AccessAnesthesiology Multimedia Video
AccessScience
JAMAevidence

JAMAevidence: RCE Education Guides
JAMAevidence: Care at the Close of Life
JAMAevidence: Rational Clinical Exam
JAMAevidence: Users'' Guides
Pharmacotherapy Principles and Practice
MECS Publisher
Media History Digital Library
韓國歷代文集叢書 (Korean Anthology in History)
Medical Journals
Medical Online Library
Medical*Online-E
Medical*Online E-Books Library (年間購読制)
MedicalFinder
MedicalFinder 法人サービス
MedicinesComplete
Medknow Open Access Journals
MedReviews Journals
Digital Archive Initiative Material Culture Review
Hoover's Online
Mergent International Company Data
Mergent Online
Mergent U.S. Company Data
Socialist Register
Mathematics in Engineering, Science & Aerospace (MESA)
Metapress American Art Therapy Association
Metapress American Association of Clinical Endocrinologists
Metapress American Pharmacists Association
Metapress Arizona State University School of Public Affairs
Metapress Association of Baccalaureate Social Work Program Directors
Metapress Canadian Meteorological and Oceanographic Society
Metapress Canadian Periodical for Community Studies
Metapress Chattagram Maa-O-Shishu Hospital Medical College
Metapress Chemistry Central Ltd
Metapress International Association of Yoga Therapists
Metapress Journals
Metapress Korean Physical Society
Metapress Laser Pages Publishing Ltd.
Metapress Max Weber Studies Office
Metapress Migration Letters
Metapress National Council for the Social Studies

Metapress Pavilion Journals
Metapress Practical Action Publishing
Metapress Psycke-Logo Press, Ltd.
Metapress Science from Israel
Metapress Scrivener Publishing
Metapress Shanghai University Press
Metapress The InnoVision Group
Metapress The Polymer Society of Korea
Metapress Université Catholique de Louvain and University of Leuven
Metapress Verlag C.H. Beck
Microbiology Society
Middlesex University Digital Repository
Mineralogical Society of America
Mineralogical Society of America:Master
MAUKDOP00000: Finance 18
MAUKDOS00000: Library Special
MABRAEE00000 - Brazil: Beauty Aids
MABRDMX00000 - Brazil: Food
MABRDNI00000 - Brazil: Lifestyle Specials
MABRGLO00000 - Brazil: Alimento
MABRGLP00000 - Brazil: Bebida
MACAFIN00000 - Canada: Finance Intelligence
MACAGLW00000 - Canada: Food: Canada
MACAGLX00000 - Canada: Drink: Canada
MACAGLY00000 - Canada: Lifestyles: Canada
MACAGLZ00000 - Canada: Retail: Canada
MACNAZC00000 - China: Financial Consumer
MACNCJR00000 - China: Technology
MACNDMG00000 - China: Travel
MACNDNC00000 - China: Household
MACNDNE00000 - China: Automotive
MACNDNF00000 - China: Lifestyles
MAEUAXC00000 - Europe: European Retail Briefing
MAEUDMT00000 - Europe: Retail Exposure
MAEUDNK00000 - Europe: Travel and Tourism Industry
MAEUTTA00000 - Europe: Travel and Tourism Analyst (TTA)
MAIEFIN00000 - Ireland: Finance Intelligence
MAIELSR00000 - Ireland: Leisure Intelligence
MAIEMKT00000 - Ireland: Market Intelligence
MAIERTL00000 - Ireland: Retail Intelligence

MAIESPC00000 - Ireland: Special Reports
MAIIDNB00000 - International: Beauty and Personal Care
MAIIEBO00000 - Academic International Reports
MAIIGAM00000 - International: Food & Drink
MAIIGIQ00000 - International: Household & Personal Care
MAIIRTL00000 - International: Retail Intelligence
MAUKDMN00000 - UK: Industrial Build (MBD)
MAUKDMO00000 - UK: Industrial Business (MBD)
MAUKDMP00000 - UK: Industrial Engineering (MBD)
MAUKDMS00000 - UK: Financial Specials
MAUKDNG00000 - UK: UK Retail Briefing
MAUKEBA00000 - UK: Retail Banking
MAUKECW00000 - UK: Travel Journals: International
MAUKEVC00000 - UK: Travel Industry
MAUKEVI00000 - UK: Investment
MAUKEVK00000 - UK: Financial Services Big Picture
MAUKEYB00000 - UK: Clothing and Footwear
MAUKGFZ00000 - UK: Household Care
MAUKGGA00000 - UK: Home Retailing
MAUKGGB00000 - UK: Retail
MAUKGLL00000 - UK: B2B Healthcare
MAUKGLM00000 - UK: Utilities and Energy
MAUKGMD00000 - UK: Financial Advice
MAUKGMF00000 - UK: Retail: Big Picture
MAUKGMM00000 - UK: Business - Technology: UK
MAUKGMN00000 - UK: Business: Finance - UK
MAUKGOT00000 - UK: Brands: Big Picture
MAUSDND00000 - US: Electronics
MAUSDNQ00000 - US: Retailing
MAUSEWV00000 - US: Transport and Transportation
MAUSFIN00000 - US: Finance Intelligence
MAUSGPA00000 - US: US Shopper
MAXXDMV00000 - Other: Country Reports (TTI)
Mintel Reports (Mintel Oxygen)
Mintel Automotive: UK
Mintel Beauty and Personal Care: Brazil
Mintel Beauty and Personal Care: China
Mintel Beauty and Personal Care: UK
Mintel Beauty and Personal Care: USA
Mintel Continental European Consumer Lifestyles: Europe

Mintel Drink: UK
Mintel Drink: USA
Mintel Finance Standard
Mintel Finance Standard Plus
Mintel Finance: USA
Mintel Financial Services - Insurance and Protection: UK
Mintel Financial Services - Intermediaries: UK
Mintel Financial Services - Investment and Savings: UK
Mintel Financial Services - Pensions and Retirement: UK
Mintel Food and Drink: Brazil
Mintel Food and Drink: China
Mintel Food: UK
Mintel Food: USA
Mintel Foodservice: Brazil
Mintel Foodservice: China
Mintel Foodservice: UK
Mintel Foodservice: USA
Mintel Health and Wellbeing: UK
Mintel Health and Wellbeing: USA
Mintel Household: Brazil
Mintel Household: USA
Mintel International Travel Series
Mintel Ireland: Industrial
Mintel Leisure and Entertainment: USA
Mintel Leisure: UK
Mintel Lifestyles: Brazil
Mintel Lifestyles: UK
Mintel Lifestyles: USA
Mintel MBD Industrial: UK
Mintel Media: UK
Mintel Mintel Reports: Ireland
Mintel Multicultural America: USA
Mintel Retail - E-Commerce: UK
Mintel Retail Exposure: International
Mintel Retail Intelligence: Europe
Mintel Retail: Brazil
Mintel Retail: China
Mintel Retailing and Apparel: USA
Mintel Technology: UK
Mintel Travel and Tourism Analyst (International)

Mintel Travel and Tourism: International
Mintel Travel: UK
Mintel Travel: USA
Miscellaneous eBooks
MIT Sloan Management Review
CogNet Library Journals
MIT Press Journals
Moazine
Mobilization
Mohr Siebeck Zeitschriften Online
Monash University EPress Journals
Montana Memory Project CONTENTdm Repository
Montana State University Digital Initiatives
Montana State University ScholarWorks
Synthesis Lectures on Human-Centered Informatics
Colloquium digital library of life sciences: Collection 3
Morgan & Claypool Biomedical Engineering Collection One
Morgan & Claypool Biomedical Engineering Collection Three
Morgan & Claypool Biomedical Engineering Collection Two
Morgan & Claypool Colloquium Collection 1
Morgan & Claypool Colloquium Collection 2
Morgan & Claypool Colloquium Collection 3
Morgan & Claypool Colloquium Collection 4
Morgan & Claypool Computer & Information Science Collection Eight
Morgan & Claypool Computer & Information Science Collection Five
Morgan & Claypool Computer & Information Science Collection Four
Morgan & Claypool Computer & Information Science Collection One
Morgan & Claypool Computer & Information Science Collection Seven
Morgan & Claypool Computer & Information Science Collection Six
Morgan & Claypool Computer & Information Science Collection Three
Morgan & Claypool Computer & Information Science Collection Two
Morgan & Claypool Digital Circuits Collection One
Morgan & Claypool Digital Circuits Collection Two
Morgan & Claypool Electromagnetics & Antennas Collection One
Morgan & Claypool General Engineering, Technology & Mathematics Collection One
Morgan & Claypool General Engineering, Technology & Mathematics Collection Three
Morgan & Claypool General Engineering, Technology & Mathematics Collection Two
Morgan & Claypool Signal Processing & Communications Collection One
Morgan & Claypool Signal Processing & Communications Collection Three
Morgan & Claypool Signal Processing & Communications Collection Two

Morgan & Claypool Synthesis Collection Eight
Morgan & Claypool Synthesis Collection Five
Morgan & Claypool Synthesis Collection Four
Morgan & Claypool Synthesis Collection One
Morgan & Claypool Synthesis Collection Seven
Morgan & Claypool Synthesis Collection Six
Morgan & Claypool Synthesis Collection Three
Morgan & Claypool Synthesis Collection Two
Morgan & Claypool Synthesis Digital Library of Engineering and Computer Science
Morgan Claypool Synthesis Lectures
Synthesis Collection Seven
Moshe Dayan Center - Tel Aviv University - מרכז משה דיין - אוניברסיטת תל אביב
MPublishing Journals
KB+ JISC Collections Multi-Science Publishing Full Collection 2015
Murdoch Research Repository
mybrary - Business
mybrary - Economics
mybrary - Law
Naace Journals
Narr Francke Attempto Verlag Zeitschriften und Jahrbücher
NASA Technical Reports Server
SAO/NASA Astrophysics Data System Historical Scans
SAO/NASA Astrophysics Data System Proceedings
Norske og nordiske tidsskriftsartikler (Norart)
National Academies Press
National Academy of Sciences (U.S.)
NABE Publications
National Association for the Teaching of English (NATE)
National Association of Geoscience Teachers Publications
National Bureau of Economic Research
National Bureau of Economic Research Publications
NBER
Tobacco Control Research Publications
National Chung Hsing University Institutional Repository - NCHUIR
TRAP@NCI
National Council of Teachers of English (NCTE)
ADEAC, a System of Digitalization and Exhibition for Archive Collections (デジタルアーカイブシステム ADEAC)
Agriculture, Forestry and Fisheries Research Information Technology Center (農林水産関係試験研究機関総合目録)
AKITA PREFECTURAL LIBRARY DIGITAL ARCHIVE (秋田県立図書館デジタルアーカイブ)
Aomori Prefectural Library Digital Archive (青森県立図書館デジタルアーカイブ)

Aozora Bunko (青空文庫)
CiNii Books
ColBase:Integrated Collection Database of the National Museums, Japan (ColBase:国立博物館所蔵品統合検索システム)
Collection (九大コレクション)
Cultural Heritage Online (文化遺産オンライン)
Digital Collections of Keio University Libraries (慶應義塾大学メディアセンター デジタルコレクション)
Digital library of Chiba Prefectural Central Library - Forest of material (資料の森（千葉県立中央図書館電子図書館）)
Fisheries Research Center Library and Archives - Digital Archive (水産総合研究センター図書資料デジタルアーカイブ)
Fukuoka Prefectural Library - Digital Library (福岡県立図書館 デジタルライブラリ)
Gifu Prefectural Library - Materials about Gifu prefecture & Maps (岐阜県図書館　岐阜県関係資料＋地図資料)
Hakodate City Central Library - Digital archive and gallery (函館市中央図書館所蔵デジタルアーカイブ　デジタル資料館)
Hiroshima Central City Library - Special Collections (広島市立中央図書館 特別集書)
Hokkaido Prefectural Library - Northern Materials Digital Library (北海道立図書館 北方資料デジタル・ライブラリー)
Ibaraki Prefectural Library - Digital Library (茨城県立図書館デジタルライブラリー)
Index to Laws & Regulations in early Meiji Japan (日本法令索引（明治前期編）)
Ishikawa Prefectural Library - Rare Material Gallery and Regional Material Library (石川県立図書館 貴重資料ギャラリー・地域資料ライブラリー)
Iwate Prefectural Library - Ihatov Iwate digital library (イーハトーブ岩手電子図書館（岩手県立図書館）)
Japanese Periodicals Index (NDL 雑誌記事索引)
Kanagawa Prefectural Library - Digital Archive (神奈川県立図書館 デジタルアーカイブ)
Kawasaki City Library Web Gallery (川崎市立図書館 Web ギャラリー)
Keio University Library - Digital Gallery (慶應義塾図書館デジタルギャラリー)
Komazawa University Digital Library (駒澤大学電子貴重書庫)
Mie Prefectural Library - Digital gallery of Japanese and Chinese Materials (三重県立図書館 和漢籍資料デ ジタル閲覧室)
Ministry of Agriculture, Forestry and Fisheries Digitalized Resource (農林水産省図書館・電子化図書一覧公開システム)
Miyagi Prefectural Library - WEB Forest of Wisdom (叡智の杜 WEB（宮城県図書館）)
Miyazaki Prefectural Library - Rare Books Digital Archive (宮崎県立図書館 貴重書デジタルアーカイブ)
Nagoya Tsurumai Central Library - Picture postcard collection of Nagoya (名古屋の絵葉書集（名古屋市鶴舞中央図書館）)
Nara Prefectural Library and Information Center - Digitized book and image exhibition gallery (奈良県立図書情報館 ふるさとデジタル化書籍＋絵図展示ギャラリー)
Nara Regional Historical Catalog (奈良県地域史料目録)
Nara Women''s University - Image database (奈良女子大学所蔵資料電子画像集)
National Diet Library - Electronic Exhibition (国立国会図書館電子展示会)
National Diet Library Online Public Access Catalog (NDL-OPAC)
NDL Digital Collections (Online publications) （国立国会図書館デジタルコレクション（電子書籍・電子雑誌）
NDL Digital Collections (国立国会図書館デジタルコレクション) 館内公開
NDL Search
Niigata Prefectural Library and Archives - Echigo Sado Digital Library (新潟県立図書館 新潟県立文書館越後佐渡デジタルライブラリー)
Okayama Municipal Central Library - Local document image collection (岡山市立中央図書館 郷土資料画像集)
Okinawa Prefectural Library "Valuable Materials Digital Archive" ("沖縄県立図書館『貴重資料デジタル書庫』")
Online catalog of reading room material related to Okinawa war (Not digitalized) (沖縄戦関係資料閲覧室 所蔵資料検索（未電子化）)

Online catalog of reading room material related to Okinawa war (沖縄戦関係資料閲覧室 所蔵資料検索)
Rare books image database of Osaka Prefectural Nakanoshima Library (大阪府立図書館　おおさかeコレクション)
Saitama Prefectural Library - Digital library (埼玉県立図書館 デジタルライブラリー)
Sapporo Municipal Central Library - Digital library (札幌市中央図書館デジタルライブラリー)
Shiga Prefectural Library - Ohmi Digital Historical Road (滋賀県立図書館 近江デジタル歴史街道)
Shizuoka Prefectural Central Library - Digital Library (静岡県立中央図書館 デジタルライブラリー)
Supreme Court of Japan OPAC (最高裁判所図書館 蔵書検索)
Tokushima Prefectural Library - Digital Library (徳島県立図書館 デジタルライブラリ)
Tottori Prefectural Library - Map Images (鳥取県立図書館所蔵絵図)
Toyama Prefectural Library - Gallery of rare pictures and books (富山県立図書館 古絵図・貴重書ギャラリー)
Union Catalog of Newspapers (新聞総合目録)
University of Tsukuba Library - Rare Books Collection (筑波大学附属図書館 貴重書コレクション)
UTokyo Academic Archives Portal (東京大学学術資産等アーカイブズポータル)
Yamagata Prefectural Library - World of lithograph of Takahashi Yuichi (高橋由一 石版画の世界（山形県立図書館）)
Yamanashi Digital Archive (山梨デジタルアーカイブ（山梨県立図書館）)
Yokohama City Central Library - Yokohama''s Memory (都市横浜の記憶（横浜市中央図書館）)
NFSC Portal for Journals
National Forum Journals (Free Journals)
National Geographic com
medIND: Biomedical Journals from India
JAIRO: Japanese Institutional Repositories Online (English)
JAIRO: Japanese Institutional Repositories Online (Japanese)
National Institute of Standards and Technology Virtual Library
National Journal Publications
Archived websites (1996 now)
Australian Periodical Publications 1840-1845
Digitised Newspapers and More
National Library of Australia Historic Australian Newspapers, 1803-1954
Trove
Trove Australian Thesis (Abstracts)
Trove Australian Thesis (Full Text - Open Access & Abstracts)
Trove Australian Thesis (Full Text - Open Access)
NLM Catalog
PubMed
Toxline
Index New Zealand (Open Access)
Papers Past
Publications New Zealand
doiSerbia
ANL - Narodni baze analytickeho popisu clanku (Articles from Czech Newspapers and Periodicals)

Manuscriptorium
Welsh Journals Online
NOAA Central Library Publications
CISTI Source
GEOSCAN
Korean Citation Index
Korean Research Memory (KRM)
National Science Teachers Association
National Catholic Bioethics Center
National University of Ireland Maynooth - CALM
National University of Ireland Maynooth Libguides
NUIM ePrints and eTheses Archive
Naturalis Biodiversity Center
KB+ JISC Collections Nature Publishing Group 2015
KB+ JISC Collections Nature Publishing Group 2016
KB+ JISC Collections Nature Publishing Group 2017
KB+ JISC Collections Nature Publishing Group 2018
KB+ JISC Collections Nature Publishing Group 2018 (Pre 2018 Subscribers)
Nature
Nature Free
Nature Partner Journals
Nature Precedings
Nature Publishing Group: NESLI2: 2014
North East Research Libraries Nature Research & Reviews
Calhoun: The NPS Institutional Archive
Naxos Music Library
Naxos Music Library Jazz
Naxos Spoken Word Library
Naxos Video Library
NBCLearn
JeugdLiteRom
LiteRom
LiteRom Wereldliteratuur
UittrekselBank
UittrekselBank Jeugd
Academia
Nepal Journals Online
JapanKnowledge Encyclopedia of Japan
JapanKnowledge Lib
JapanKnowledge 国史大辞典(Encyclopedia of Japanese History)

JapanKnowledge 現代用語の基礎知識(Encyclopedia of contemporary words)
JapanKnowledgeデジタル化学辞典(第2版)(Digital Dictionary of Chemistry(Second Edition))
JapanKnowledge世界大百科事典 (Sekai Daihyakkajiten - Heibonsha's World Encyclopedia)
JapanKnowledge世界文学大事典(The Shueisha Dictionary of World Literature)
JapanKnowledge情報・知識imidas(Innovative Multi-Information Dictionary, Annual Series)
JapanKnowledge文庫クセジュ ベストセレクション(Collection ≪Que sais-je?≫ Best Selections)
JapanKnowledge新版 歌舞伎事典(The Kabuki Dictionary: New Edition)
JapanKnowledge新版 能・狂言事典(The Noh and Kyogen Dictionary: New Edition)
JapanKnowledge新編 日本古典文学全集(The Complete Collection of Japanese Classical Literature, New Edition)
JapanKnowledge日本人名大辞典(The Biographical Dictionary of Japan)
JapanKnowledge日本国語大辞典(Shogakukan Unabridged Dictionary of the Japanese Language)
JapanKnowledge日本大百科全書(Complete Japanese Encyclopedia)
JapanKnowledge日本架空伝承人名事典 (Dictionary of Japanese Fictional and Legendary Characters)
JapanKnowledge日本歴史地名大系(Encyclopedia of Japanese Historical Place Names)
JapanKnowledge東洋文庫(Toyo Bunko (The Eastern Library))
JapanKnowledge法則の辞典(Dictionary of Laws of Natural Science)
JapanKnowledge法律用語辞典(第4版)(Dictionary of Legal Terms (4th Edition))
SPED理工系英和 (SPED Science and Technology English-Japanese Dictionary)
The ORIENTAL ECONOMIST1（1934-1945）
The ORIENTAL ECONOMIST2（1946-1952）
The ORIENTAL ECONOMIST3（1953-1963）
The ORIENTAL ECONOMIST4（1964-1973）
The ORIENTAL ECONOMIST5（1974-1985）
デジタル大辞泉 (Digital Dai-Ji-Sen)
デジタル大辞泉プラス (Digital Dai-ji-sen Plus)
プログレッシブ和英中辞典 (Progressive Japanese-English Dictionary)
プログレッシブ英和中辞典 (Progressive English-Japanese Dictionary)
プログレビジネス英語辞典 (Progressive Business English Dictionary)
ポケプロ中日辞典 (Pocket Progressive Chinese-Japanese Dictionary)
ポケプロ仏和辞典 (Pocket Progressive French-Japanese Dictionary)
ポケプロ伊和辞典 (Pocket Progressive Italian-Japanese Dictionary)
ポケプロ和仏辞典 (Pocket Progressive Japanese-French Dictionary)
ポケプロ和伊辞典 (Pocket Progressive Jaapnese-Italian Dictionary)
ポケプロ和独辞典 (Pocket Progressive Japanese-German Dictionary)
ポケプロ和西辞典 (Pocket Progressive Japanese-Spanish Dictionary)
ポケプロ日中辞典 (Pocket Progressive Japanese-Chinese Dictionary)
ポケプロ日韓辞典 (Pocket Progressive Japanese-Korean Dictionary)
ポケプロ独和辞典 (Pocket Progressive German-Japanese Dictionary)
ポケプロ西和辞典 (Pocket Progressive Spanish-Japanese Dictionary)
ポケプロ韓日辞典 (Pocket Progressive Korean-Japanese Dictionary)

ランダムハウス英和大辞典 (Random House English-Japanese Dictionary)
ロベール仏和大辞典 (Robert grand dictionnaire français-japonais)
人物叢書1 (Jinbutsu Sosho)
人物叢書2 (Jinbutsu Sosho)
人物叢書3 (Jinbutsu Sosho)
人物叢書4 (Jinbutsu Sosho)
人物叢書5 (Jinbutsu Sosho)
人物叢書6 (Jinbutsu Sosho)
伊和中辞典 (Shogakukan Italian-Japanese Dictionary, 2nd Edition)
例文仏教語大辞典(Dictionary of Buddhist Terminology)
全文全訳古語辞典 (Unabridged Ancient Japanese Dictionary)
医学英和辞典 (English-Japanese Dictionary of Medical Science)
和伊中辞典 (Shogakukan Japanese-Italian Dictionary, 2nd Edition)
和羅辞典 (Lexikon Japonicum - Latino)
図書館情報学用語辞典 (Toshokan Johogaku Yogojiten)
太陽1（明28-36）(Taiyo 1)
太陽2（明34-39）(Taiyo 2)
太陽3（明40-大1）(Taiyo 3)
字通 (Ji-Tsu)
岩波数学辞典 (Iwanami dictionary of mathematics)
弘文荘待賈古書目 (Kobunso Taika Koshomoku)
数え方の辞典 (Dictionary of Japanese Counting Words)
文芸倶楽部1（明28-明33）(Bungei Club 1)
文芸倶楽部2（明34-明39）(Bungei Club 2)
文芸倶楽部3（明40-大1）(Bungei Club 3)
文藝春秋 1 大12-昭7(1923-1932) (Bungeishunju Archives)
文藝春秋 2 昭8-16(1933-1941) (Bungeishunju Archives)
文藝春秋 3 昭17-25(1942-1950) (Bungeishunju Archives)
新編国歌大観 (Encyclopedia of Japanese Waka Poetry)
日本人物文献目録 (Biography of Japanese Biographies)
日本国勢図会 (Nihon kokusei zu-e)
日本方言大辞典 (Nihon Hogen Daijiten)
日本統計年鑑 (Japan statistical yearbook)
日本長期統計総覧 (Historical statistics of Japan)
東洋経済1（明28-45） (Weekly Toyo Keizai Digital Archives 1)
東洋経済2（大1-15） (Weekly Toyo Keizai Digital Archives 2)
東洋経済3（昭2-20） (Weekly Toyo Keizai Digital Archives 3)
東洋経済4（昭21-42） (Weekly Toyo Keizai Digital Archives 4)
東洋経済5（昭43-58） (Weekly Toyo Keizai Digital Archives 5)
東洋経済6（昭59-平11） (Weekly Toyo Keizai Digital Archives 6)

東洋経済7（平12-27） (Weekly Toyo Keizai Digital Archives 7)
校友会雑誌(Koyukai Zasshi)
独和大辞典〔第2版〕 (Grosses Deutsch-Japanisches Wörterbuch)
理化学英和辞典 (English-Japanese Dictionary of the Physical Science)
続々群書類従(Gunsho Ruiju 3)
続群書類従(Gunsho Ruiju 2)
羅和辞典 (Lexicon Latino-Japonicum)
美術新報 (Bijutsu Shinpo)
群書類従(Gunsho Ruiju)
西和中辞典 (Shogakukan Spanish-Japanese Dictionary, 2nd Edition)
角川古語大辞典 (Kadokawa Kogo Daijiten)
角川日本地名大辞典 (Kadokawa Great Dictionary of Japanese Place Names)
近代作家原稿集（樗陰旧蔵）(Kindai Sakka Genkoshu)
風俗画報(Fuzoku Gaho)
NetLibrary Lyrasis Shared Collection III
NetLibrary Lyrasis Shared Collection IV
NetLibrary Lyrasis Shared Collection IX
NetLibrary Lyrasis Shared Collection V
NetLibrary Lyrasis Shared Collection VI
NetLibrary Lyrasis Shared Collection VII
NetLibrary Lyrasis Shared Collection VIII
Networked Digital Library of Theses and Dissertations
NeuroScience Publishers
New Delhi Publishers
New England Journal of Medicine (EIRA)
New England Journal of Medicine Current
New York Public Library Digital Gallery
New York Public Library Picture Collection Online
New York Review of Books Archive
Newfound Press Books
Newnonmun
Black Authors, 1556-1922: Imprints from the Library Company of Philadelphia
Early American imprints. Series I, Supplement from the Library Company of Philadelphia, 1670-1800
NewsBank Acceda Noticias
NewsBank Access Business News
NewsBank Access Military, Gov, Defense
Newsbank Access World News
Newsbank Access World News Research Collection
Newsbank America's News
NewsBank America's News Magazines

Newsbank Early American Newspapers Series 1
Early American imprints. Series II, Supplement from the Library Company of Philadelphia, 1801-1819
U.S. Congressional Serial Set
MagazinePlus
NII-REO IEEE CSDL
NII-REO Kluwer Full
NII-REO Kluwer HSS
NII-REO Kluwer STM
NII-REO OUP Archive Full
NII-REO OUP Online
NII-REO Springer Online
Nikkei Asian Review
USNA Digital Collection
National Institute of Science Communication and Information Resources (NISCAIR)
NISO Publications
Nomos eLibrary
NORA - Norwegian Open Research Archives
Norden Publikationer (Norden Publications)
NPSIA Publications
Foundations and Trends E-Books
Foundations and Trends in Business and Economics
Foundations and Trends in Technology
Now Publishers Journals
BACON - American Institute of Physics AIP - COUPERIN_SOCIETIES-TITLES
NSTL 购买电子全文期刊
AIP期刊回溯库
WILEY期刊回溯库
NUMDAM Free
DBPIA - English
Nurimedia BookRail Books
Nurimedia DBPIA Journals
Nurimedia KRPIA
NZCER Journals Online
Oakland University OUR@Oakland
OAPEN Free
Avery Index to Architectural Periodicals
MEDLINE
OCLC FirstSearch ECO
OCUL Ebook Collection Brill Biblical

OCUL Ebook Collection Brill Classical
OCUL Ebook Collection Brill European
OCUL Ebook Collection Brill Law
OCUL Ebook Collection Brill Middle East
OCUL Ebook Collection Brill Religious
OCUL eBook Collection Duke
OCUL eBook Collection IEEE
OCUL eBook Collection Lippincott Williams and Wilkins
OCUL eBook Collection SPIE
OCUL eBook Collection University of Chicago
OCUL eBook Collection Wiley
OCUL Ebook CollectionBrill Social
ITU iLibrary
Nordic iLibrary
OECD Books and Papers
OECD Economic Surveys
OECD Economics iLibrary
OECD Education iLibrary
OECD Employment iLibrary
OECD Finance and Investment iLibrary
OECD Governance iLibrary
OECD iLibrary
OECD iLibrary Books Complete
OECD iLibrary Health Statistics
OECD iLibrary Statistics
OECD iLibrary Working Paper Series
OECD Social Issues/Migration/Health iLibrary
OECD Statistics
OECD Urban, Rural and Regional Development iLibrary
OECD/ITF Transport iLibrary
DOE Patents
OSTI.GOV
Ohio State University Press Knowledge Bank Free Collection During the COVID-19
Digital Resource Commons
OhioLINK Electronic Theses and Dissertations
Old City Publishing
Oncology Nursing Society
OnePetro
Open Book Publishers
OpenAIRE

Hypotheses
OpenEdition Books Complete
OpenEdition Freemium for Journals - Couperin
Openedition French Studies Bundle - Freemium
Openedition Journals Complete
OpenEdition Journals Freemium
OpenEdition Open Access and Freemium Journals Bundle
Openedition Open access Journals
OpenStax College
OpinionArchives
DRAA OSA E-Combination Package
DRAA OSA Optics Infobase
DRAA OSA Optics InfoBase Premium
OSA Digital Archive
The Optical Society Publishing Journals
The Optical Society Publishing Proceedings
Oral History Association Publications
O'Reilly Online Learning: Academic/Public Library Edition
O'Reilly Online Learning: Corporate Edition
O'Reilly Open Books
Commonwealth iLibrary
OECD Statistics
Ornithological Society of New Zealand Publications
Oslo Metropolitan University Open Digital Archive
OsloMet Fagarkivet
Otzar HaHochma - אוצר החכמה
Books@Ovid - American Nurses Association Collection 2017-2018 Purchase
Books@Ovid - Oncology Nursing Society Book Collection 2018 Purchase
Books@Ovid - Oncology Nursing Society Book Collection 2018 Subscription
Books@Ovid Purchase
Books@Ovid Purchase Complete
Books@Ovid Subscription
Books@Ovid Subscription Complete
British Nursing Index (BNI) (1985 to Present)
EconLit (Ovid)
EconLit (SilverPlatter UK)
ERIC (Ovid)
ERIC(SilverPlatter)
International Bibliography of the Social Sciences
JBI Complete Collection (JHOS-CS-I19)

Journals@Ovid
Journals@Ovid Complete
Journals@Ovid Emcare with LWW Nursing Journals 2017-2018
Journals@Ovid LWW Journal Definitive Archive Collection - Renewals Only
Journals@Ovid LWW Nursing and Health Professions Premier 2017 Revised
Journals@Ovid LWW Nursing and Health Professions Premier 2017-2018
Journals@Ovid LWW Total Access Collection 2018 minus Neurology
Journals@Ovid LWW Total Access Collection 2020 minus Neurology
Journals@Ovid LWW Total Access Collection 2020 minus Neurology N20
Journals@Ovid LWW Total Access Collection 2020 with Neurology
Journals@Ovid LWW Total Access Collection 2020 with Neurology N20
Journals@Ovid LWW Total Access Collection with Neurology
Journals@Ovid Open Access Journal Collection Rolling
MEDLINE (Ovid)
Ovid ADIS International Collection
Ovid ATLA RDB
Ovid CINAHL
Ovid Circulation Collection
Ovid Lippincott Williams & Wilkins Current Opinion Collection
Ovid Lippincott Williams & Wilkins High Impact Collection 2020
Ovid Lippincott Williams & Wilkins Journal Definitive Archive
Ovid Lippincott Williams & Wilkins Nursing Health Professions Premier 2014
Ovid Lippincott Williams & Wilkins Nursing Health Professions Premier 2016
Ovid Lippincott Williams & Wilkins Nursing Health Professions Premier 2017
Ovid Lippincott Williams & Wilkins Premier Collection 2008
Ovid Lippincott Williams & Wilkins Total Access Collection 2009
Ovid Lippincott Williams & Wilkins Total Access Collection 2010
Ovid Lippincott Williams & Wilkins Total Access Collection 2011
Ovid Lippincott Williams & Wilkins Total Access Collection 2012
Ovid Lippincott Williams & Wilkins Total Access Collection 2013
Ovid Lippincott Williams and Wilkins Journal Legacy Archive
Ovid Lippincott Williams and Wilkins Total Access Collection 2014
Ovid Lippincott Williams and Wilkins Total Access Collection 2015
Ovid Lippincott Williams and Wilkins Total Access Collection 2016
Ovid Lippincott Williams and Wilkins Total Access Collection 2017
Ovid LWW Medical Book Collection - No textbooks (2009)
Ovid LWW Medical Book Collection - No textbooks (2010)
Ovid LWW Medical Book Collection 2009
Ovid LWW Nursing and Health Professions Premier Collection 2009
Ovid Nursing Collection 1

Ovid Nursing Collection II: Lippincott Premier Nursing Journals
Ovid Nursing Community College Basic Journal Collection
Ovid Nursing Community College Extended Journal Collection
Ovid Nursing Full Text Plus Collection
Ovid Premier Collection
Ovid PsycARTICLES
Ovid PsycBooks
PAIS International (Ovid)
Sociological Abstracts (Ovid)
Oxford Art Online
Oxford Handbooks Online Archaeology
Oxford Handbooks Online Business & Management
Oxford Handbooks Online Classical Studies
Oxford Handbooks Online Complete
Oxford Handbooks Online Criminology and Criminal Justice
Oxford Handbooks Online Economics and Finance
Oxford Handbooks Online History
Oxford Handbooks Online Law
Oxford Handbooks Online Linguistics
Oxford Handbooks Online Literature
Oxford Handbooks Online Music
Oxford Handbooks Online Philosophy
Oxford Handbooks Online Political Science
Oxford Handbooks Online Psychology
Oxford Handbooks Online Religion
Oxford Medical Handbooks Online
Oxford Islamic Studies Online
Oxford Medicine Online
Oxford Music Online
Oxford Reference Library
Oxford Reference Premium Collection
Oxford Research Encyclopedias
Oxford Scholarly Editions Online
Oxford Scholarly Editions Online Early Seventeenth Century Drama
Oxford Scholarly Editions Online Early Seventeenth Century Poetry
Oxford Scholarly Editions Online Early Seventeenth Century Prose
Oxford Scholarly Editions Online Eighteenth Century Drama
Oxford Scholarly Editions Online Eighteenth Century Poetry
Oxford Scholarly Editions Online Eighteenth Century Prose
Oxford Scholarly Editions Online Latin Drama

Oxford Scholarly Editions Online Latin History
Oxford Scholarly Editions Online Latin Poetry
Oxford Scholarly Editions Online Latin Prose
Oxford Scholarly Editions Online Nineteenth Century Poetry
Oxford Scholarly Editions Online Nineteenth Century Prose
Oxford Scholarly Editions Online OSEO: 19th Century Prose
Oxford Scholarly Editions Online OSEO: Medieval Poetry
Oxford Scholarly Editions Online OSEO: Medieval Prose
Oxford Scholarly Editions Online Renaissance Drama
Oxford Scholarly Editions Online Renaissance Poetry
Oxford Scholarly Editions Online Renaissance Prose
Oxford Scholarly Editions Online Restoration Drama
Oxford Scholarly Editions Online Restoration Poetry
Oxford Scholarly Editions Online Restoration Prose
Oxford Scholarly Editions Online Romantics Poetry
Oxford Scholarly Editions Online Romantics Prose
Oxford Scholarly Editions Online Shakespeare
Oxford Scholarship - Oxford University Press: Biology
Oxford Scholarship - Oxford University Press: Business and Management
Oxford Scholarship - Oxford University Press: Classical Studies
Oxford Scholarship - Oxford University Press: Economics and Finance
Oxford Scholarship - Oxford University Press: History
Oxford Scholarship - Oxford University Press: Law
Oxford Scholarship - Oxford University Press: Linguistics
Oxford Scholarship - Oxford University Press: Literature
Oxford Scholarship - Oxford University Press: Mathematics
Oxford Scholarship - Oxford University Press: Music
Oxford Scholarship - Oxford University Press: Neuroscience
Oxford Scholarship - Oxford University Press: Palliative Care
Oxford Scholarship - Oxford University Press: Philosophy
Oxford Scholarship - Oxford University Press: Physics
Oxford Scholarship - Oxford University Press: Political Science
Oxford Scholarship - Oxford University Press: Psychology
Oxford Scholarship - Oxford University Press: Public Health and Epidemiology
Oxford Scholarship - Oxford University Press: Religion
Oxford Scholarship - Oxford University Press: Social Work
Oxford Scholarship - Oxford University Press: Sociology
Oxford Scholarship Online Complete
University Press Scholarship Online Anthropology
University Press Scholarship Online Archeology

University Press Scholarship Online Architecture
University Press Scholarship Online Art
University Press Scholarship Online Biology
University Press Scholarship Online Business And Management
University Press Scholarship Online Classics
University Press Scholarship Online Complete
University Press Scholarship Online Computer Science
University Press Scholarship Online Earth Sciences and Geography
University Press Scholarship Online Economics and Finance
University Press Scholarship Online Education
University Press Scholarship Online Environmental Science
University Press Scholarship Online Film Television and Radio
University Press Scholarship Online History
University Press Scholarship Online Information Science
University Press Scholarship Online Law
University Press Scholarship Online Linguistics
University Press Scholarship Online Literature
University Press Scholarship Online Mathematics
University Press Scholarship Online Music
University Press Scholarship Online Neuroscience
University Press Scholarship Online Palliative Care
University Press Scholarship Online Philosophy
University Press Scholarship Online Physics
University Press Scholarship Online Political Science
University Press Scholarship Online Psychology
University Press Scholarship Online Public Health and Epidemiology
University Press Scholarship Online Religion
University Press Scholarship Online Social Work
University Press Scholarship Online Society and Culture
University Press Scholarship Online Sociology
VIVA Oxford University Press Journals
VIVA University Press Scholarship Online Ebooks
AMA Manual of Style
American National Biography Online
Benezit Dictionary of Artists
CRKN Oxford University Press Current
DRAA Oxford Journals Collection 2015
DRAA Oxford Journals Humanities Collection-牛津现刊数据库人文科学分库
DRAA Oxford Journals Law Collection-牛津现刊数据库法律分库

DRAA Oxford Journals Life Sciences Collection-牛津现刊数据库生命科学分库
DRAA Oxford Journals Mathematics and Physical Sciences Collection-牛津现刊数据库数学和物理学分库
DRAA Oxford Journals Medicine Collection-牛津现刊数据库医学分库
DRAA Oxford Journals Social Sciences Collection-牛津现刊数据库社会科学分库
Grove Encyclopedia of Classical Art and Architecture
Investment Claims
IReL-KB Oxford Journals Online
JMLA/JPLA Oxford University Press 2018
JMLA/JPLA Oxford University Press 2020
JUSTICE Oxford University Press 2014
JUSTICE Oxford University Press 2017
KB+ BIBSAM Oxford University Press Journals (including new titles 2015-2018)
KB+ BIBSAM Oxford University Press Journals 2014 collection 2015-2018
KB+ BIBSAM Oxford University Press Journals 2016 collection 2017
KB+ Bibsam Oxford University Press Journals 2020 Collection 2020
KB+ JISC Collections Oxford University Press Archive and Archive Upgrade (2017-2020)
KB+ JISC Collections Oxford University Press Economics And Finance Collection 2015
KB+ JISC Collections Oxford University Press Economics and Finance Collection 2016
KB+ JISC Collections Oxford University Press Economics and Finance Collection 2017
KB+ JISC Collections Oxford University Press Full Collection 2017
KB+ JISC Collections Oxford University Press Full Collection 2018-2019
KB+ JISC Collections Oxford University Press Humanities And Social Sciences 2017
KB+ JISC Collections Oxford University Press Humanities And Social Sciences Collection 2015
KB+ JISC Collections Oxford University Press Humanities And Social Sciences Collection 2018
KB+ JISC Collections Oxford University Press Humanities Collection 2015
KB+ JISC Collections Oxford University Press Humanities Collection 2016
KB+ JISC Collections Oxford University Press Humanities Collection 2017
KB+ JISC Collections Oxford University Press Humanities Collection 2018
KB+ JISC Collections Oxford University Press Journals Collection Excluding New Titles 2015
KB+ JISC Collections Oxford University Press Journals Collection including 20 new titles 2016
KB+ JISC Collections Oxford University Press Journals Collection Including 25 New Titles 2015
KB+ JISC Collections Oxford University Press Law Collection 2015
KB+ JISC Collections Oxford University Press Law Collection 2016
KB+ JISC Collections Oxford University Press Law Collection 2017
KB+ JISC Collections Oxford University Press Law Collection 2018
KB+ JISC Collections Oxford University Press Life Sciences Collection 2015
KB+ JISC Collections Oxford University Press Life Sciences Collection 2016
KB+ JISC Collections Oxford University Press Life Sciences Collection 2017
KB+ JISC Collections Oxford University Press Life Sciences Collection 2018

KB+ JISC Collections Oxford University Press Mathematics & Physical Science Collection 2016
KB+ JISC Collections Oxford University Press Mathematics & Physical Science Collection 2018
KB+ JISC Collections Oxford University Press Mathematics And Physical Science 2017
KB+ JISC Collections Oxford University Press Mathematics And Physical Science Collection 2015
KB+ JISC Collections Oxford University Press Medicine Collection 2015
KB+ JISC Collections Oxford University Press Medicine Collection 2016
KB+ JISC Collections Oxford University Press Medicine Collection 2017
KB+ JISC Collections Oxford University Press Medicine Collection 2018
KB+ JISC Collections Oxford University Press Science Technology And Medicine 2017
KB+ JISC Collections Oxford University Press Science Technology And Medicine Collection 2015
KB+ JISC Collections Oxford University Press Science Technology And Medicine Collection 2018
KB+ JISC Collections Oxford University Press Social Sciences Collection 2015
KB+ JISC Collections Oxford University Press Social Sciences Collection 2016
KB+ JISC Collections Oxford University Press Social Sciences Collection 2017
KB+ JISC Collections Oxford University Press Social Sciences Collection 2018
KB+ Oxford University Press: JISC Collections:Archive and Archive Upgrade
KB+ WHEEL Oxford University Press Journals Collection 2015 (including 25 new titles) 2017
KB+ WHEEL/NESLI2 Oxford University Press Journals Collection 2014
Max Planck Encyclopedia of Public International Law
Oxford Biblical Studies Online
Oxford Bibliographies
Oxford Bibliographies Online : Anthropology
Oxford Bibliographies Online : Biblical Studies
Oxford Bibliographies Online : Childhood Studies
Oxford Bibliographies Online : Ecology
Oxford Bibliographies Online : International Law
Oxford Bibliographies Online : Military History
Oxford Bibliographies Online: Atlantic History
Oxford Bibliographies Online: Buddhism
Oxford Bibliographies Online: Cinema and Media Studies
Oxford Bibliographies Online: Classics
Oxford Bibliographies Online: Communication
Oxford Bibliographies Online: Criminology
Oxford Bibliographies Online: Hinduism
Oxford Bibliographies Online: International Relations
Oxford Bibliographies Online: Islamic Studies
Oxford Bibliographies Online: Latin American Studies
Oxford Bibliographies Online: Linguistics
Oxford Bibliographies Online: Medieval Studies
Oxford Bibliographies Online: Music

Oxford Bibliographies Online: Philosophy
Oxford Bibliographies Online: Political Science
Oxford Bibliographies Online: Psychology
Oxford Bibliographies Online: Public Health
Oxford Bibliographies Online: Renaissance and Reformation
Oxford Bibliographies Online: Sociology
Oxford Bibliographies Online: Victorian Literature
Oxford Classical Dictionary
Oxford Clinical Psychology
Oxford Competition Law
Oxford Constitutions of the World
Oxford Dictionary of National Biography
Oxford Digital Reference Shelf
Oxford English Dictionary
Oxford Handbooks Online 2012 Business and Management
Oxford Handbooks Online 2012 Classical Studies
Oxford Handbooks Online 2012 Economics and Finance
Oxford Handbooks Online 2012 Linguistics
Oxford Handbooks Online 2012 Political Science
Oxford Handbooks Online 2012 Psychology
Oxford Handbooks Online 2013 Criminology and Criminal Justice
Oxford Handbooks Online 2013 Political Science
Oxford Handbooks Online 2014 Business & Management
Oxford Handbooks Online 2014 History
Oxford Handbooks Online 2015 Business & Management
Oxford Handbooks Online 2015 History
Oxford Handbooks Online 2015 Music
Oxford Handbooks Online 2016 Business and Management
Oxford Handbooks Online 2016 Economics and Finance
Oxford Handbooks Online 2017 Law
Oxford Handbooks Online Foundation History Collection
Oxford Historical Treaties
Oxford Journals 2016 Current and Archive A-Z Collection
Oxford Journals 2017 Archive - IReL
Oxford Journals 2017 Current Collection with OpenAccess
Oxford Journals 2017 Open Access Collection
Oxford Journals 2018 Economics and Finance Collection
Oxford Journals 2018 Hss Collection
Oxford Journals 2018 Humanities Archive
Oxford Journals 2018 Humanities Collection

Oxford Journals 2018 Law Archive
Oxford Journals 2018 Law Collection
Oxford Journals 2018 Life Sciences Collection
Oxford Journals 2018 Mathematics and Physical Sciences Collection
Oxford Journals 2018 Medicine Archive
Oxford Journals 2018 Medicine Collection
Oxford Journals 2018 Open Access Titles Collection
Oxford Journals 2018 Policy Collection
Oxford Journals 2018 Religion and Philosophy Collection
Oxford Journals 2018 Science Archive
Oxford Journals 2018 Social Sciences Archive
Oxford Journals 2018 Social Sciences Collection
Oxford Journals 2018 Stm Collection
Oxford Journals 2020 Clinical Medicine
Oxford Journals 2020 Economics and Finance
Oxford Journals 2020 HSS
Oxford Journals 2020 Humanities
Oxford Journals 2020 Humanities Archive
Oxford Journals 2020 Law
Oxford Journals 2020 Law Archive
Oxford Journals 2020 Life Sciences
Oxford Journals 2020 Mathematics and Physical Sciences
Oxford Journals 2020 Medicine
Oxford Journals 2020 Medicine Archive
Oxford Journals 2020 Near Archive
Oxford Journals 2020 Policy
Oxford Journals 2020 Religion and Philosophy
Oxford Journals 2020 Science Archive
Oxford Journals 2020 Social Sciences
Oxford Journals 2020 STM
Oxford Journals Archive Collection
Oxford Journals A-Z Archive
Oxford Journals A-Z Collection
Oxford Journals Collection (EIRA)
Oxford Journals Current Collection
Oxford Journals Open Access Collection
Oxford Legal Research Library
Oxford Legal Research Library - Financial and Banking Law
Oxford Legal Research Library - International Commercial Arbitration
Oxford Reports on International Law

Oxford Scholarly Authorities on International Law
Oxford Scholarly Editions Online - Early Seventeenth Century Verse
Oxford University Press African American Studies Center
Oxford University Press Humanities Archive
Oxford University Press Journals
Oxford University Press Journals Collection - Near Archive KERIS
Oxford University Press Journals Current
Oxford University Press Journals Digital Archive
Oxford University Press Law Archive
Oxford University Press Law Trove
Oxford University Press Medicine Archive
Oxford University Press Open Access Books
Oxford University Press Science Archive
Oxford University Press Social Science Archive
Parry''s Consolidated Treaty Series
US Constitutional Law
WHEEL Oxford University Press
Who's Who / Who Was Who
Oxford Very Short Introductions: Arts And Humanities
Oxford Very Short Introductions: Complete
Oxford Very Short Introductions: History
Oxford Very Short Introductions: Law
Oxford Very Short Introductions: Literature
Oxford Very Short Introductions: Medicine And Health
Oxford Very Short Introductions: Philosophy
Oxford Very Short Introductions: Physics
Oxford Very Short Introductions: Politics
Oxford Very Short Introductions: Psychology
Oxford Very Short Introductions: Religion
Oxford Very Short Introductions: Science And Mathematics
Oxford Very Short Introductions: Social Sciences
Pagepress
PANGAEA
Paradigm Publishers Journals
Peeters Online Journals
Peking - pishu
Penn State Digital Library Collections
IK: Other Ways of Knowing
Pensiero Journals Open Access
Pensiero Scientifico Editore

Pensoft Open Access Journals
Aviation Week
PERSEE - Portail de revues scientifiques en sciences humaines et sociales
Pertanika Journals
Peter Lang ebooks
Pew Hispanic Center Site Search
journalism.org
Pew Forum on Religion & Public Life
Pew Global Attitudes Project
Pew Internet
Pew Research Center for the People & the Press
Pew Research Center Publications
Pew Social & Demographic Trends
Philosophy Documentation Center E-Collection
POIESIS: Philosophy Online Serials
AusDI Advanced
Physical Society of Japan (JPS)
Psychiatrist.com
PierOnline Books
PierOnline Journals
Plant Management Network
Industry Almanac eBooks
Plunkett Research Online
Biblioteka Wirtualna Nauki, Kolekcja Nauk Przyrodniczych
Polish Virtual Library of Science Mathematical Collection
PUCRS Institutional Repository
Revistas PUCP
PoPuPS
Portfolio Management Research
Portico Triggered Ebook For Participants
Portico Triggered Ejournal For Participants
Portico Triggered Ejournal Open Access
Biochemical Journal
Portland Press Electronic Journals
Practising Law Institute (PLI) PLI PLUS
Ernst Reinhardt E-Books
facultas.wuv und Maudrich Verlag
Ferdinand Schöningh
Herbert von Halem
Narr Francke Attempto Verlag E-Books

UVK E-Books
Vandenhoeck & Ruprecht
Verlag Barbara Budrich
W. Bertelsmann Verlag
Waxmann
WBG
Wilhelm Fink Verlag
Digital Collections at Princeton Theological Seminary
Department of Mathematics at Princeton University Publications
Digital Papers of Albert Einstein
Progress of Theoretical Physics
KB+ JISC Collections Project Euclid Prime 2016
KB+ JISC Collections Project Euclid Prime 2017
KB+ Jisc Collections Project Euclid Prime 2018
Lecture Notes in Logic - Monograph Series
Notre Dame Mathematical Lectures - Monograph Series
Proceedings of the Centre for Mathematics and its Applications
Project Euclid Complete
Project Euclid Direct
Project Euclid Open Access Journals
Project Euclid Prime
Project Gutenberg Online Catalog
Project HOPE
Booker T. Washington Papers
KB+ JISC Collections Project Muse Arts Collection 2017
KB+ Jisc Collections Project Muse Arts Collection 2018-2020
KB+ JISC Collections Project Muse Asian Studies Collection 2017
KB+ JISC Collections Project Muse Asian Studies Collection 2018-2020
KB+ Jisc Collections Project Muse Basic College Collection (2018-2020)
KB+ JISC Collections Project Muse Basic College Collection 2015-2017
KB+ Jisc Collections Project Muse Basic Research Collection (2018-2020)
KB+ JISC Collections Project Muse Basic Research Collection 2015-2017
KB+ JISC Collections Project Muse History Collection 2017
KB+ JISC Collections Project Muse History Collection 2018-2020
KB+ Jisc Collections Project Muse Humanities Collection (2018-2020)
KB+ JISC Collections Project Muse Humanities Collection 2015-2017
KB+ JISC Collections Project Muse Literature Core Collection 2017
KB+ JISC Collections Project Muse Literature Core Collection 2018-2020
KB+ JISC Collections Project Muse Literature Expanded Collection 2017
KB+ JISC Collections Project Muse Literature Expanded Collection 2018-2020

KB+ JISC Collections Project Muse Philosophy & Religion 2018-2020
KB+ JISC Collections Project Muse Philosophy Religion Collection 2017
KB+ Jisc Collections Project Muse Premium Collection (2018-2020)
KB+ Jisc Collections Project Muse Social Sciences Collection (2018-2020)
KB+ JISC Collections Project Muse Social Sciences Collection 2015-2017
KB+ Jisc Collections Project Muse Standard Collection (2018-2020)
KB+ JISC Collections Project Muse Standard Collection 2015-2017
Marx & Engels Collected Works
Project MUSE - Classic Cornell University Press Open Access Books
Project Muse All Journals
Project Muse American Literature
Project Muse Archive Complete Supplement
Project Muse Archive Complete Supplement 3
Project Muse Archive Complete Supplement 4
Project Muse Arts Collection
Project Muse Asian Studies
Project Muse Basic College Collection
Project Muse Basic Research Collection
Project Muse British and Irish Literature
Project Muse Classical and Medieval Studies
Project Muse Ebooks 2010-2012
Project Muse eBooks 2011
Project Muse eBooks 2013
Project Muse eBooks 2014
Project Muse eBooks 2015
Project Muse eBooks 2016
Project Muse eBooks 2017
Project Muse Ebooks Archive
Project Muse eBooks Supplement 2 2012
Project Muse eBooks Supplement 2 2013
Project Muse eBooks Supplement 2011
Project Muse eBooks Supplement 2013
Project Muse eBooks Supplement 2014
Project Muse Film, Theater and Performing Arts
Project Muse Global Cultural Studies
Project Muse History Collection
Project Muse Humanities Collection
Project Muse Literary Magazines
Project Muse Literary Studies (General)
Project Muse Literature Core Collection

Project Muse Literature Expanded Collection
Project MUSE Open Access Books
Project Muse Philosophy & Religion Collection
Project Muse Premium Collection
Project Muse Religious Studies
Project Muse Social Sciences Collection
Project Muse Standard Collection
UPCC Books - Single Title
UPCC Books 2011 Global Cultural Studies Collection Supplement
UPCC Books 2011 History Collection Supplement
UPCC Books 2011 Political Science and Policy Studies Collection Supplement
UPCC Books 2012 Complete Collection Supplement
UPCC Books 2016 Higher Education Collection
UPCC Books 2018 Higher Education
UPCC Books 2018 Literature
UPCC Books Archive Classics Collection Supplement
UPCC Books Archive Complete Collection Supplement
UPCC Books Archive Complete Foundation Supplement II
UPCC Books Archive Film, Theater and Performing Arts Collection Supplement
UPCC Books Archive Global Cultural Studies Collection Supplement
AAA: Architecture & Design
AAA: Art & Photography
ABI/INFORM Collection
ABI/INFORM Collection (Alumni)
ABI/INFORM Collection China
ABI/INFORM Dateline
ABI/INFORM Dateline (Alumni)
ABI/INFORM Global
ABI/INFORM Global (Alumni)
ABI/INFORM Professional Advanced
ABI/INFORM Professional Market Research
ABI/INFORM Professional Standard
ABI/INFORM Trade & Industry
ABI/INFORM Trade & Industry (Alumni)
Academic Video Online
Accounting, Tax & Banking Collection
Accounting, Tax & Banking Collection (Alumni)
Advanced Technologies & Aerospace Collection
Advanced Technologies & Aerospace Database
Advanced Technologies & Aerospace Index

Aerospace Database
African American Poetry
African Writers Series
African Writers Series (pre-September 2011 purchase)
Agricultural & Environmental Science Collection
Agricultural Science Collection
Agricultural Science Database
Alaska Newsstand
Alt-PressWatch
Alt-PressWatch (Alumni)
Aluminium Industry Abstracts
American Drama 1714-1915
American Periodicals from the Center for Research Libraries
American Periodicals Series
American Poetry
ANTE: Abstracts in New Technology & Engineering
APA PsycARTICLES
Applied Social Sciences Index & Abstracts (ASSIA)
Aqualine
Aquatic Science & Fisheries Abstracts (ASFA) Professional
Arizona Republic
Art & Architecture Archive Collection 1
Art & Architecture Archive Collection 2
Art, Design & Architecture Collection
ARTbibliographies Modern (ABM)
ARTbibliographies Modern (ABM) for DFG
Arts & Humanities Database
Arts Premium Collection
ASFA: Aquatic Sciences and Fisheries Abstracts
Asian & European Business Collection
Asian & European Business Collection (Alumni)
Asian Newsstream
Asian Newsstream (Alumni)
Atlanta Journal & Constitution
Australia & New Zealand Database
Australia & New Zealand Newsstream
Baltimore Sun
Baltimore Sun (Alumni)
Barron's
Bertolt Brechts Werke

Bibliografía de la Literatura Española
Biological Science Collection
Biological Science Database
Biological Science Index
Black Short Fiction and Folklore
Black Women Writers
Boston Globe
British Nursing Database
British Nursing Index
British Periodicals Collection I
British Periodicals Collection II
British Periodicals Collection III
British Periodicals Collection IV
British Periodicals Collection IV (China Edition)
Business Market Research Collection
Business Market Research Collection (Alumni)
Business Monitor International
Business Premium Collection
Business Premium Collection (Alumni)
Canadian Business & Current Affairs Database
Canadian Business & Current Affairs Database (Alumni)
Canadian Major Dailies
Canadian Newsstream
Canadian Poetry
Canadian Research Index
Career & Technical Education Database
Career & Technical Education Database (Alumni)
Caribbean Literature
Chemoreception Abstracts
Chicago Tribune
Chicago Tribune (Alumni)
Colonial State Papers
ComDisDome
Computer Science Database
Congressional Base
Congressional Indexes
Congressional Research Digital Collection 1979
Congressional Statutes at Large
Consumer Health Database
Consumer Health Database (Alumni)

Continental Europe Database
Copper Technical Reference Library
Coronavirus Research Database
Country Life Archive
Courier-Journal (Louisville)
Criminal Justice Database
Criminal Justice Database (Alumni)
Criminology Collection
DELNET Engineering & Technology Collection
DELNET Management Collection
DELNET Social Sciences & Humanities Collection
Des Moines Register
Design & Applied Arts Index (DAAI)
Detroit Free Press
Die Deutsche Lyrik in Reclams Universal-Bibliothek
Digital Complete Prospective 2014-
Digital National Security Archive - U.S. Nuclear Nonproliferation 2, Part I: From Atoms for Peace to the NPT, 1954-1968
Digital National Security Archive (DNSA)
Digital National Security Archive (DNSA): Afghanistan: The Making of U.S. Policy, 1973-1990
Digital National Security Archive (DNSA): Argentina, 1975-1980: The Making of U.S. Human Rights Policy
Digital National Security Archive (DNSA): Chile and the United States: U.S. Policy toward Democracy, Dictatorship, and Human Rights, 1970-1990
Digital National Security Archive (DNSA): China and the United States: From Hostility to Engagement, 1960-1998
Digital National Security Archive (DNSA): CIA Covert Operations II: The Year of Intelligence, 1975
Digital National Security Archive (DNSA): CIA Covert Operations III: From Kennedy to Nixon, 1961-1974
Digital National Security Archive (DNSA): CIA Covert Operations: From Carter to Obama, 1977-2010
Digital National Security Archive (DNSA): CIA Family Jewels Indexed
Digital National Security Archive (DNSA): Colombia and the United States: Political Violence, Narcotics, and Human Rights, 1948-2010
Digital National Security Archive (DNSA): Cuba and the U.S.: The Declassified History of Negotiations to Normalize Relations, 1959-2016
Digital National Security Archive (DNSA): Death Squads, Guerrilla War, Covert Ops, and Genocide: Guatemala and the United States, 1954-1999
Digital National Security Archive (DNSA): El Salvador: The Making of U.S. Policy, 1977-1984
Digital National Security Archive (DNSA): El Salvador: War, Peace, and Human Rights, 1980-1994
Digital National Security Archive (DNSA): Electronic Surveillance and the National Security Agency: From Shamrock to Snowden
Digital National Security Archive (DNSA): Iran: The Making of U.S. Policy, 1977-1980
Digital National Security Archive (DNSA): Iraqgate: Saddam Hussein, U.S. Policy and the Prelude to the Persian Gulf War, 1980-1994
Digital National Security Archive (DNSA): Japan and the United States: Diplomatic, Security, and Economic Relations, 1960-1976
Digital National Security Archive (DNSA): Japan and the United States: Diplomatic, Security, and Economic Relations, 1977-1992
Digital National Security Archive (DNSA): Japan and the United States: Diplomatic, Security, and Economic Relations, Part III, 1961-2000
Digital National Security Archive (DNSA): Mexico-United States Counternarcotics Policy, 1969-2013
Digital National Security Archive (DNSA): Nicaragua: The Making of U.S. Policy, 1978-1990
Digital National Security Archive (DNSA): Peru: Human Rights, Drugs and Democracy, 1980-2000

Digital National Security Archive (DNSA): Presidential Directives on National Security, Part I: From Truman to Clinton
Digital National Security Archive (DNSA): Presidential Directives on National Security, Part II: From Truman to George W. Bush
Digital National Security Archive (DNSA): South Africa: The Making of U.S. Policy, 1962-1989
Digital National Security Archive (DNSA): Soviet-U.S. Relations: The End of the Cold War, 1985-1991
Digital National Security Archive (DNSA): Targeting Iraq, Part 1: Planning, Invasion, and Occupation, 1997-2004
Digital National Security Archive (DNSA): Terrorism and U.S. Policy, 1968-2002
Digital National Security Archive (DNSA): The Berlin Crisis, 1958-1962
Digital National Security Archive (DNSA): The Cuban Missile Crisis Revisited: An International Collection, From Bay of Pigs to Nuclear Brink
Digital National Security Archive (DNSA): The Cuban Missile Crisis, 1962
Digital National Security Archive (DNSA): The Cuban Missile Crisis: 50th Anniversary Update
Digital National Security Archive (DNSA): The Iran-Contra Affair: The Making of a Scandal, 1983-1988
Digital National Security Archive (DNSA): The Kissinger conversations, supplement II: A verbatim record of U.S. diplomacy, 1969-1977
Digital National Security Archive (DNSA): The Kissinger Conversations, Supplement: A Verbatim Record of U.S. Diplomacy, 1969-1977
Digital National Security Archive (DNSA): The Kissinger Telephone Conversations: A Verbatim Record of U.S. Diplomacy, 1969-1977
Digital National Security Archive (DNSA): The Kissinger Transcripts: A Verbatim Record of U.S. Diplomacy, 1969-1977
Digital National Security Archive (DNSA): The National Security Agency: Organization and Operations, 1945-2009
Digital National Security Archive (DNSA): The Philippines: U.S. Policy During the Marcos Years, 1965-1986
Digital National Security Archive (DNSA): The President's Daily Brief: Kennedy, Johnson and the CIA
Digital National Security Archive (DNSA): The Soviet Estimate: U.S. Analysis of the Soviet Union, 1947-1991
Digital National Security Archive (DNSA): The U.S. Intelligence Community After 9/11
Digital National Security Archive (DNSA): The U.S. Intelligence Community: Organization, Operations and Management, 1947-1989
Digital National Security Archive (DNSA): The United States and the Two Koreas (1969-2000)
Digital National Security Archive (DNSA): The United States and the Two Koreas, Part II, 1969-2010
Digital National Security Archive (DNSA): U.S. Espionage and Intelligence, 1947-1996
Digital National Security Archive (DNSA): U.S. Intelligence and China: Collection, Analysis and Covert Action
Digital National Security Archive (DNSA): U.S. Intelligence on Weapons of Mass Destruction: From World War II to Iraq
Digital National Security Archive (DNSA): U.S. Military Uses of Space, 1945-1991
Digital National Security Archive (DNSA): U.S. Nuclear History, 1969-1976: Weapons, Arms Control, and War Plans in an Age of Strategic Parity
Digital National Security Archive (DNSA): U.S. Nuclear History: Nuclear Arms and Politics in the Missile Age, 1955-1968
Digital National Security Archive (DNSA): U.S. Nuclear Non-Proliferation Policy, 1945-1991
Digital National Security Archive (DNSA): U.S. Policy in the Vietnam War, Part I: 1954-1968
Digital National Security Archive (DNSA): U.S. Policy in the Vietnam War, Part II: 1969-1975
Digital National Security Archive (DNSA): U.S. Policy toward Iran: From the Revolution to the Nuclear Accord, 1978-2015
Digital U.S. Bills and Resolutions 2015
Digital U.S. Bills and Resolutions Prospective
Digital U.S. Bills and Resolutions, 1789-2013
Digital U.S. Bills and Resolutions, 2014-
Digital U.S. Bills and Resolutions, 2016
Digitale Bibliothek Deutscher Klassiker
Dissertations & Theses @ Acadia University

Dissertations & Theses @ Adelphi University
Dissertations & Theses @ Adler School of Professional Psychology
Dissertations & Theses @ Air Force Institute of Technology
Dissertations & Theses @ Alabama A&M University
Dissertations & Theses @ Alliant International University - NCCPL
Dissertations & Theses @ American University - WRLC
Dissertations & Theses @ Anderson University
Dissertations & Theses @ Andrews University
Dissertations & Theses @ Angelo State University
Dissertations & Theses @ Arcadia University
Dissertations & Theses @ Arizona State University
Dissertations & Theses @ Arkansas State University
Dissertations & Theses @ Arkansas Tech University
Dissertations & Theses @ Ashland University
Dissertations & Theses @ Auburn University
Dissertations & Theses @ Aurora University
Dissertations & Theses @ Austin Presbyterian Theological Seminary
Dissertations & Theses @ Australian National University
Dissertations & Theses @ Azusa Pacific University - SCELC
Dissertations & Theses @ Baker College
Dissertations & Theses @ Baker University
Dissertations & Theses @ Ball State University
Dissertations & Theses @ Barry University
Dissertations & Theses @ Baylor University Library
Dissertations & Theses @ Bellarmine University
Dissertations & Theses @ Binghamton University
Dissertations & Theses @ Biola University
Dissertations & Theses @ Boston College
Dissertations & Theses @ Boston University
Dissertations & Theses @ Bowling Green State University
Dissertations & Theses @ Bradley University
Dissertations & Theses @ Brandeis University
Dissertations & Theses @ Brown University
Dissertations & Theses @ Bryn Mawr College
Dissertations & Theses @ Caldwell University
Dissertations & Theses @ California Institute of Integral Studies - NCCPL
Dissertations & Theses @ California Institute of Technology
Dissertations & Theses @ California State University, Dominguez Hills
Dissertations & Theses @ California State University, Fullerton
Dissertations & Theses @ California State University, Long Beach

Dissertations & Theses @ California State University, Los Angeles
Dissertations & Theses @ Capella University
Dissertations & Theses @ Cardinal Stritch University
Dissertations & Theses @ Carleton University
Dissertations & Theses @ Carnegie Mellon University
Dissertations & Theses @ Case Western Reserve University
Dissertations & Theses @ Catholic University of America - WRLC
Dissertations & Theses @ Central Michigan University
Dissertations & Theses @ Chapman University
Dissertations & Theses @ Charles Univ - Karlova - CERGE
Dissertations & Theses @ Chestnut Hill College
Dissertations & Theses @ Chicago School of Professional Psychology
Dissertations & Theses @ Chinese University of Hong Kong
Dissertations & Theses @ Christopher Newport University
Dissertations & Theses @ City University of New York Graduate Center
Dissertations & Theses @ Claremont School of Theology
Dissertations & Theses @ Claremont University Consortium
Dissertations & Theses @ Clarion University
Dissertations & Theses @ Clark University
Dissertations & Theses @ Clarkson University
Dissertations & Theses @ Clemson University
Dissertations & Theses @ Cleveland State University
Dissertations & Theses @ College of Medicine - Mayo Clinic
Dissertations & Theses @ College of Saint Elizabeth
Dissertations & Theses @ College of St Mary''s
Dissertations & Theses @ Colorado School of the Mines
Dissertations & Theses @ Colorado State University
Dissertations & Theses @ Columbia University
Dissertations & Theses @ Concordia University
Dissertations & Theses @ Corcoran College of Art + Design
Dissertations & Theses @ Cornell University and the Weill Medical College
Dissertations & Theses @ Creighton University
Dissertations & Theses @ Dalhousie University
Dissertations & Theses @ Dallas Theological Seminary
Dissertations & Theses @ Dartmouth College
Dissertations & Theses @ DePaul University
Dissertations & Theses @ Dominican University College
Dissertations & Theses @ Dowling College
Dissertations & Theses @ Drew University
Dissertations & Theses @ Drexel University

Dissertations & Theses @ Duke University
Dissertations & Theses @ Duquesne University
Dissertations & Theses @ East Carolina University
Dissertations & Theses @ East Tennessee State University
Dissertations & Theses @ Eastern Michigan University
Dissertations & Theses @ Ecole de Technologie Superieure (ETS)
Dissertations & Theses @ Ecole Polytechnique de Montreal
Dissertations & Theses @ Edgewood College
Dissertations & Theses @ Emory University
Dissertations & Theses @ Emporia State University
Dissertations & Theses @ ESSEC Business School
Dissertations & Theses @ Fairleigh Dickinson University
Dissertations & Theses @ Fashion Institute of Technology
Dissertations & Theses @ Fielding Graduate University
Dissertations & Theses @ Florida A&M University - FCLA
Dissertations & Theses @ Florida Atlantic University - FCLA
Dissertations & Theses @ Florida Gulf Coast University - FCLA
Dissertations & Theses @ Florida Institute of Technology
Dissertations & Theses @ Florida International University - FCLA
Dissertations & Theses @ Florida State University - FCLA
Dissertations & Theses @ Fordham University
Dissertations & Theses @ Fuller Theological Seminary
Dissertations & Theses @ Gallaudet University - WRLC
Dissertations & Theses @ Gannon University
Dissertations & Theses @ Gardner-Webb
Dissertations & Theses @ George Mason University - WRLC
Dissertations & Theses @ George Washington University - WRLC
Dissertations & Theses @ Georgetown University - WRLC
Dissertations & Theses @ Georgia Institute of Technology
Dissertations & Theses @ Georgia Regents University
Dissertations & Theses @ Georgia Southern University
Dissertations & Theses @ Georgia State University
Dissertations & Theses @ Golden Gate University - SCELC
Dissertations & Theses @ Gonzaga University
Dissertations & Theses @ Grand Canyon University
Dissertations & Theses @ Grand Valley State University
Dissertations & Theses @ Hampton University
Dissertations & Theses @ Hartford Seminary
Dissertations & Theses @ Harvard University
Dissertations & Theses @ Hofstra University

Dissertations & Theses @ Hong Kong Baptist University
Dissertations & Theses @ Hong Kong Polytechnic University
Dissertations & Theses @ Hong Kong University of Science & Technology
Dissertations & Theses @ Hood College
Dissertations & Theses @ Howard University
Dissertations & Theses @ Illinois Institute of Technology
Dissertations & Theses @ Illinois State University
Dissertations & Theses @ Immaculata University
Dissertations & Theses @ Indiana State University
Dissertations & Theses @ Indiana University of Pennsylvania
Dissertations & Theses @ Indiana Wesleyan
Dissertations & Theses @ INSEAD
Dissertations & Theses @ Institute for Clinical Social Work
Dissertations & Theses @ Institute of Transpersonal Psychology - NCCPL
Dissertations & Theses @ Inter-American University
Dissertations & Theses @ Iowa State University
Dissertations & Theses @ James Madison University
Dissertations & Theses @ Johns Hopkins University
Dissertations & Theses @ Johnson & Wales University
Dissertations & Theses @ Kansas State University
Dissertations & Theses @ Kent State University
Dissertations & Theses @ King Fahd Univ of Petroleum & Minerals - Dhahran
Dissertations & Theses @ Kutztown University
Dissertations & Theses @ La Sierra University
Dissertations & Theses @ Lamar University Beaumont
Dissertations & Theses @ Lancaster Theological Seminary
Dissertations & Theses @ LaSalle University
Dissertations & Theses @ Laurentian University
Dissertations & Theses @ Lawrence Technological University
Dissertations & Theses @ Lehigh University
Dissertations & Theses @ Lehman College CUNY
Dissertations & Theses @ Lesley University
Dissertations & Theses @ Lewis & Clark College
Dissertations & Theses @ Liberty University
Dissertations & Theses @ Library and Archives Canada
Dissertations & Theses @ Loma Linda University
Dissertations & Theses @ Long Island University
Dissertations & Theses @ Louisiana State University Health Sciences Center - Shreveport
Dissertations & Theses @ Louisiana Tech University
Dissertations & Theses @ Loyola University

Dissertations & Theses @ Loyola University Chicago
Dissertations & Theses @ LSU Health Sciences Center - New Orleans
Dissertations & Theses @ Lutheran School of Theology
Dissertations & Theses @ Maharishi University of Management
Dissertations & Theses @ Marian University
Dissertations & Theses @ Marquette University
Dissertations & Theses @ Marshall University
Dissertations & Theses @ Marywood University
Dissertations & Theses @ Massachusetts School of Professional Psychology
Dissertations & Theses @ McGill University
Dissertations & Theses @ McMaster University
Dissertations & Theses @ Medical College of Ohio
Dissertations & Theses @ Medical College of Wisconsin
Dissertations & Theses @ Memorial University of Newfoundland
Dissertations & Theses @ Mercer University Atlanta
Dissertations & Theses @ Metropolitan State University
Dissertations & Theses @ MGH Institute of Health Professions
Dissertations & Theses @ Miami University
Dissertations & Theses @ MICA
Dissertations & Theses @ Michigan School of Professional Psychology
Dissertations & Theses @ Michigan State University - Fisheries and Wildlife
Dissertations & Theses @ Michigan Technological University
Dissertations & Theses @ Middle Tennessee State University
Dissertations & Theses @ Mills College
Dissertations & Theses @ Mississippi State University
Dissertations & Theses @ Missouri University of Science and Technology
Dissertations & Theses @ Montana State University
Dissertations & Theses @ Montana Tech of The University of Montana
Dissertations & Theses @ Morgan State University
Dissertations & Theses @ Mount Sinai School of Medicine
Dissertations & Theses @ New England Conservatory of Music
Dissertations & Theses @ New Jersey Institute of Technology
Dissertations & Theses @ New Mexico State University
Dissertations & Theses @ New Orleans Baptist Theological Seminary
Dissertations & Theses @ New York University
Dissertations & Theses @ North Carolina State University @ Raleigh
Dissertations & Theses @ North Dakota State University
Dissertations & Theses @ Northcentral University
Dissertations & Theses @ Northeastern University
Dissertations & Theses @ Northern Arizona University

Dissertations & Theses @ Northern Illinois University - IDAL
Dissertations & Theses @ Northern Kentucky University
Dissertations & Theses @ Northern Michigan University
Dissertations & Theses @ Northwestern University
Dissertations & Theses @ Nova Southeastern University
Dissertations & Theses @ Oakland University
Dissertations & Theses @ OGI School of Science & Engineering
Dissertations & Theses @ Ohio State University
Dissertations & Theses @ Ohio University
Dissertations & Theses @ Oklahoma State University - Stillwater
Dissertations & Theses @ Old Dominion University
Dissertations & Theses @ Oral Roberts University
Dissertations & Theses @ Oregon Health and Science University
Dissertations & Theses @ Oregon State University
Dissertations & Theses @ Our Lady of the Lake University
Dissertations & Theses @ Pace University
Dissertations & Theses @ Pacific Graduate School of Psychology - NCCPL
Dissertations & Theses @ Pacifica Graduate Institute
Dissertations & Theses @ Pepperdine University - SCELC
Dissertations & Theses @ Plymouth State University
Dissertations & Theses @ Point Park University
Dissertations & Theses @ Polytechnic University
Dissertations & Theses @ PORTALS
Dissertations & Theses @ Portland State University
Dissertations & Theses @ Prescott College
Dissertations & Theses @ Princeton Theological Seminary
Dissertations & Theses @ Princeton University
Dissertations & Theses @ Queen''s University at Kingston, Ontario
Dissertations & Theses @ Quinnipiac University
Dissertations & Theses @ Regent University
Dissertations & Theses @ Rensselaer Polytechnic Institute
Dissertations & Theses @ Rhode Island College
Dissertations & Theses @ Rice University
Dissertations & Theses @ Robert Morris University
Dissertations & Theses @ Roosevelt University
Dissertations & Theses @ Rosalind Franklin University of Medicine and Science
Dissertations & Theses @ Rowan University
Dissertations & Theses @ Royal Military College of Canada
Dissertations & Theses @ Royal Roads University
Dissertations & Theses @ Rush University

Dissertations & Theses @ Rutgers University
Dissertations & Theses @ Ryerson University
Dissertations & Theses @ Saint Joseph''s University
Dissertations & Theses @ Saint Louis University
Dissertations & Theses @ Saint Mary''s University (Canada)
Dissertations & Theses @ Salisbury University
Dissertations & Theses @ Salve Regina University
Dissertations & Theses @ Sam Houston State University
Dissertations & Theses @ San Francisco State University
Dissertations & Theses @ Santa Clara University
Dissertations & Theses @ Scripps Research Institute
Dissertations & Theses @ Seattle Pacific University
Dissertations & Theses @ Seattle University
Dissertations & Theses @ Semmelweis University
Dissertations & Theses @ Seton Hall University
Dissertations & Theses @ Shenandoah University
Dissertations & Theses @ Silver Lake College
Dissertations & Theses @ Simon Fraser University
Dissertations & Theses @ Sotheby''s Institute of Arts
Dissertations & Theses @ South Carolina State University
Dissertations & Theses @ South Dakota State University
Dissertations & Theses @ Southeast Missouri State University
Dissertations & Theses @ Southern Baptist Theological Seminary
Dissertations & Theses @ Southern Connecticut State University
Dissertations & Theses @ Southern Illinois University at Carbondale
Dissertations & Theses @ Southern Illinois University Edwardsville
Dissertations & Theses @ Southern Methodist University
Dissertations & Theses @ Southwest Minnesota State University
Dissertations & Theses @ Southwestern Baptist Theological Seminary
Dissertations & Theses @ Spalding University
Dissertations & Theses @ St Mary''s University (Texas)
Dissertations & Theses @ St. Ambrose University
Dissertations & Theses @ St. Mary''s University (Minnesota)
Dissertations & Theses @ Stanford University
Dissertations & Theses @ Stephen Austin State University
Dissertations & Theses @ Stevens Institute of Technology
Dissertations & Theses @ Sul Ross State University
Dissertations & Theses @ SUNY Albany
Dissertations & Theses @ SUNY Buffalo
Dissertations & Theses @ SUNY College of Environmental Science and Forestry

Dissertations & Theses @ SUNY Stony Brook
Dissertations & Theses @ Syracuse University
Dissertations & Theses @ Temple University
Dissertations & Theses @ Tennessee State University
Dissertations & Theses @ Texas A&M System
Dissertations & Theses @ Texas Christian University
Dissertations & Theses @ Texas Tech University
Dissertations & Theses @ Texas Woman''s University
Dissertations & Theses @ The City College of New York
Dissertations & Theses @ The College of St. Scholastica
Dissertations & Theses @ The Hong Kong Institute of Education
Dissertations & Theses @ The New School
Dissertations & Theses @ The Rockefeller University
Dissertations & Theses @ The University of Rochester
Dissertations & Theses @ The Weizmann Institute of Science
Dissertations & Theses @ The William Paterson University of New Jersey
Dissertations & Theses @ Thomas Jefferson University
Dissertations & Theses @ Touro
Dissertations & Theses @ Touro University International
Dissertations & Theses @ Trident University
Dissertations & Theses @ Trinity International University - IDAL
Dissertations & Theses @ Truman State University
Dissertations & Theses @ Tufts University
Dissertations & Theses @ Tulane University
Dissertations & Theses @ UMBC
Dissertations & Theses @ Union Institute & University
Dissertations & Theses @ United States International University
Dissertations & Theses @ Univ North Texas, Health Science Center
Dissertations & Theses @ Universidad de Cadiz
Dissertations & Theses @ Universidad de Castilla La Mancha
Dissertations & Theses @ Universidad de Cordoba
Dissertations & Theses @ Universidad de Deusto
Dissertations & Theses @ Universidad de Huelva
Dissertations & Theses @ Universidad de La Rioja
Dissertations & Theses @ Universidad de Las Palmas de Gran Canaria
Dissertations & Theses @ Universidad de Valladolid
Dissertations & Theses @ Universidad de Zaragoza
Dissertations & Theses @ Universidad Internacional de La Rioja
Dissertations & Theses @ Universidad Politecnica de Cartagena
Dissertations & Theses @ Universidad Politecnica de Valencia

Dissertations & Theses @ Universidad Politécnica Puerto Rico
Dissertations & Theses @ Universidad Pontificia Comillas
Dissertations & Theses @ Universidad Publica de Navarra
Dissertations & Theses @ Universidade de Aveiro
Dissertations & Theses @ Universitat de València
Dissertations & Theses @ Universite de Moncton
Dissertations & Theses @ Universite de Montreal
Dissertations & Theses @ Universite de Sherbrooke
Dissertations & Theses @ Universite du Quebec a Chicoutimi
Dissertations & Theses @ Universite du Quebec a Montreal
Dissertations & Theses @ Universite du Quebec a Trois-Rivieres
Dissertations & Theses @ Universite Laval
Dissertations & Theses @ University College Dublin
Dissertations & Theses @ University of Adelaide
Dissertations & Theses @ University of Akron
Dissertations & Theses @ University of Alabama
Dissertations & Theses @ University of Alabama at Birmingham
Dissertations & Theses @ University of Alabama in Huntsville
Dissertations & Theses @ University of Alaska Fairbanks
Dissertations & Theses @ University of Alberta
Dissertations & Theses @ University of Arizona
Dissertations & Theses @ University of Arkansas at Pine Bluff
Dissertations & Theses @ University of Arkansas Fayetteville
Dissertations & Theses @ University of Auckland
Dissertations & Theses @ University of Bridgeport
Dissertations & Theses @ University of British Columbia
Dissertations & Theses @ University of Calgary
Dissertations & Theses @ University of California
Dissertations & Theses @ University of Central Florida-FCLA
Dissertations & Theses @ University of Central Missouri
Dissertations & Theses @ University of Chicago
Dissertations & Theses @ University of Cincinnati
Dissertations & Theses @ University of Colorado at Denver
Dissertations & Theses @ University of Colorado System
Dissertations & Theses @ University of Colorado, Boulder
Dissertations & Theses @ University of Connecticut
Dissertations & Theses @ University of Dallas
Dissertations & Theses @ University of Dayton
Dissertations & Theses @ University of Delaware
Dissertations & Theses @ University of Denver

Dissertations & Theses @ University of Florida - FCLA
Dissertations & Theses @ University of Georgia
Dissertations & Theses @ University of Guelph
Dissertations & Theses @ University of Hartford
Dissertations & Theses @ University of Hawai`i at Manoa
Dissertations & Theses @ University of Hawaii
Dissertations & Theses @ University of Hong Kong
Dissertations & Theses @ University of Houston
Dissertations & Theses @ University of Houston - Clear Lake
Dissertations & Theses @ University of Idaho
Dissertations & Theses @ University of Illinois at Springfield
Dissertations & Theses @ University of Iowa
Dissertations & Theses @ University of Kansas
Dissertations & Theses @ University of Kentucky
Dissertations & Theses @ University of La Verne - SCELC
Dissertations & Theses @ University of Lethbridge
Dissertations & Theses @ University of Louisiana @ Monroe
Dissertations & Theses @ University of Louisiana at Lafayette
Dissertations & Theses @ University of Louisville
Dissertations & Theses @ University of Maine
Dissertations & Theses @ University of Manitoba
Dissertations & Theses @ University of Maryland in Baltimore
Dissertations & Theses @ University of Massachusetts @ Amherst
Dissertations & Theses @ University of Massachusetts at Lowell
Dissertations & Theses @ University of Melbourne
Dissertations & Theses @ University of Memphis
Dissertations & Theses @ University of Miami
Dissertations & Theses @ University of Michigan
Dissertations & Theses @ University of Mississippi
Dissertations & Theses @ University of Missouri - Columbia
Dissertations & Theses @ University of Missouri - Kansas City
Dissertations & Theses @ University of Missouri - St. Louis
Dissertations & Theses @ University of Nebraska - Lincoln
Dissertations & Theses @ University of Nebraska - Omaha
Dissertations & Theses @ University of Nebraska at Kearney
Dissertations & Theses @ University of Nevada Las Vegas
Dissertations & Theses @ University of Nevada Reno
Dissertations & Theses @ University of New Brunswick
Dissertations & Theses @ University of New Hampshire
Dissertations & Theses @ University of New Haven

Dissertations & Theses @ University of New Mexico
Dissertations & Theses @ University of New South Wales
Dissertations & Theses @ University of North Carolina at Chapel Hill
Dissertations & Theses @ University of North Carolina at Greensboro
Dissertations & Theses @ University of North Carolina Charlotte
Dissertations & Theses @ University of North Dakota
Dissertations & Theses @ University of North Florida - FCLA
Dissertations & Theses @ University of North Texas
Dissertations & Theses @ University of Northern British Columbia
Dissertations & Theses @ University of Northern Colorado
Dissertations & Theses @ University of Northern Iowa
Dissertations & Theses @ University of Notre Dame
Dissertations & Theses @ University of Oklahoma
Dissertations & Theses @ University of Oregon
Dissertations & Theses @ University of Ottawa
Dissertations & Theses @ University of Pennsylvania
Dissertations & Theses @ University of Phoenix
Dissertations & Theses @ University of Pittsburgh
Dissertations & Theses @ University of Prince Edward Island
Dissertations & Theses @ University of Puerto Rico - Mayaguez
Dissertations & Theses @ University of Puerto Rico - Rio Piedras
Dissertations & Theses @ University of Queensland
Dissertations & Theses @ University of Redlands
Dissertations & Theses @ University of Regina
Dissertations & Theses @ University of Rhode Island
Dissertations & Theses @ University of Richmond
Dissertations & Theses @ University of Saint Thomas
Dissertations & Theses @ University of San Diego
Dissertations & Theses @ University of San Francisco
Dissertations & Theses @ University of Sarasota
Dissertations & Theses @ University of Saskatchewan
Dissertations & Theses @ University of South Alabama
Dissertations & Theses @ University of South Carolina
Dissertations & Theses @ University of South Dakota
Dissertations & Theses @ University of South Florida - FCLA
Dissertations & Theses @ University of Southern California
Dissertations & Theses @ University of Southern Mississippi
Dissertations & Theses @ University of St. Thomas (Houston)
Dissertations & Theses @ University of Sydney
Dissertations & Theses @ University of Tennessee - Chattanooga

Dissertations & Theses @ University of Tennessee - Knoxville
Dissertations & Theses @ University of Tennessee Health Science Center
Dissertations & Theses @ University of Texas - Arlington
Dissertations & Theses @ University of Texas - Austin
Dissertations & Theses @ University of Texas - El Paso
Dissertations & Theses @ University of Texas - San Antonio
Dissertations & Theses @ University of Texas at Dallas
Dissertations & Theses @ University of Texas Health Science Center at Houston
Dissertations & Theses @ University of Texas Pan American
Dissertations & Theses @ University of Texas School of Dentistry at Houston
Dissertations & Theses @ University of Texas, School of Public Health
Dissertations & Theses @ University of the District of Columbia
Dissertations & Theses @ University of the Incarnate Word
Dissertations & Theses @ University of the Pacific
Dissertations & Theses @ University of the Sciences of Philadelphia
Dissertations & Theses @ University of Toledo
Dissertations & Theses @ University of Toronto
Dissertations & Theses @ University of Tulsa
Dissertations & Theses @ University of Utah
Dissertations & Theses @ University of Vermont
Dissertations & Theses @ University of Victoria
Dissertations & Theses @ University of Virginia
Dissertations & Theses @ University of Washington WCLP
Dissertations & Theses @ University of Waterloo
Dissertations & Theses @ University of West Florida - FCLA
Dissertations & Theses @ University of Western Ontario
Dissertations & Theses @ University of Wisconsin at Madison
Dissertations & Theses @ University of Wisconsin Milwaukee
Dissertations & Theses @ University of Wollongong
Dissertations & Theses @ University of Wyoming
Dissertations & Theses @ Utah State University
Dissertations & Theses @ Utica College
Dissertations & Theses @ Vanderbilt University
Dissertations & Theses @ Villanova University
Dissertations & Theses @ Virginia Commonwealth University
Dissertations & Theses @ Virginia Polytechnic Institute and State University
Dissertations & Theses @ Wake Forest University
Dissertations & Theses @ Washington State University WCLP
Dissertations & Theses @ Washington University in St. Louis
Dissertations & Theses @ Wayne State University

Dissertations & Theses @ Webster University
Dissertations & Theses @ Wesleyan University
Dissertations & Theses @ West Virginia University
Dissertations & Theses @ Western Michigan University
Dissertations & Theses @ Westminster Theological Seminary
Dissertations & Theses @ Wichita State University
Dissertations & Theses @ Widener University
Dissertations & Theses @ Wilfrid Laurier University
Dissertations & Theses @ Wilkes University
Dissertations & Theses @ Wilmington University
Dissertations & Theses @ Winebrenner Theological Seminary
Dissertations & Theses @ Xavier University
Dissertations & Theses @ Yale University
Dissertations & Theses @ York University
Dissertations and Theses @ CIC Institutions
Documents on British Policy Overseas
Early American Fiction 1789-1875
Early English Books Online
Early English Prose Fiction
Early European Books - Collection 1
Early European Books - Collection 10
Early European Books - Collection 11
Early European Books - Collection 12
Early European Books - Collection 13
Early European Books - Collection 14
Early European Books - Collection 15
Early European Books - Collection 16
Early European Books - Collection 17
Early European Books - Collection 18
Early European Books - Collection 2
Early European Books - Collection 3
Early European Books - Collection 4
Early European Books - Collection 5
Early European Books - Collection 6
Early European Books - Collection 7
Early European Books - Collection 8
Early European Books - Collection 9
Early European Books National Access Denmark - KBDK Collection
Early European Books National Access Italy - BNCF Collection
Early European Books National Access Netherlands - KBNL Collection

Early European Books Wellcome Trust Collection
Earth, Atmospheric & Aquatic Science Collection
Earth, Atmospheric & Aquatic Science Database
East & South Asia Database
East Europe, Central Europe Database
EconLit
Education Collection
Education Database
Education Database (Alumni)
Education Magazine Archive
Eighteenth-Century Fiction
Electronics & Communications Abstracts
eLibrary
Engineered Materials Abstracts
Engineering Basic PRO
Engineering Collection
Engineering Database
Engineering Index
Engineering Premium PRO
English Drama
English Poetry
English Poetry, Second Edition
Entertainment Industry Magazine Archive: Cinema, Film and Television (Part 1)
Entertainment Industry Magazine Archive: Cinema, Film and Television (Part 2)
Entertainment Industry Magazine Archive: Music, Radio and The Stage
Entrepreneurship Database
Entrepreneurship Database (Alumni)
Environmental Engineering Abstracts
Environmental Science Collection
Environmental Science Database
Environmental Science Index
ERIC
Ethnic NewsWatch
Ethnic NewsWatch (Alumni)
Ethnic NewsWatch: A History
Ethnic NewsWatch: A History (Alumni)
European Newsstream
European NewsstreamÂ (Alumni)
FIAF International Index to Film Periodicals
FIAF International Index to Film Periodicals Database

GenderWatch
GenderWatch (Alumni)
Genetics Abstracts (Online)
Global Breaking Newswires
Global News & ABI/Inform Professional
Global Newsstream
Global Newsstream (Alumni)
Goethes Werke
Hartford Courant
Hartford Courant (Alumni)
Health & Fitness Magazine Archive
Health & Medical Collection
Health & Medical Collection (Alumni)
Health and Safety Science Abstracts (Full archive)
Health Research Premium Collection
Health Research Premium Collection (Alumni)
Healthcare Administration Database
Healthcare Administration Database (Alumni)
Historic Literary Criticism
Historical Annual Reports
History Vault: African American Police League Records
History Vault: American Politics & Society from JFK to Watergate, 1960-1975
History Vault: American Politics in Early Cold War: Truman & Eisenhower Administrations, 1945-1961
History Vault: Black Freedom Struggle in C20: Federal Government Records
History Vault: Black Freedom Struggle in C20: Federal Government Records Supplement
History Vault: Black Freedom Struggle in C20: Organizational Records & Personal Papers, Part 1-a
History Vault: Black Freedom Struggle in C20: Organizational Records & Personal Papers, Part 1-b
History Vault: Black Freedom Struggle in C20: Organizational Records & Personal Papers, Part 2
History Vault: Confederate Military Manuscripts & Records of Union Generals & the Union Army
History Vault: Confidential U.S. State Department Central Files 1960-1969: Africa & Middle East
History Vault: Confidential U.S. State Department Central Files 1960-1969: Asia
History Vault: Confidential U.S. State Department Central Files 1960-1969: Europe & Latin America
History Vault: Creation of Israel: British Foreign Office Correspondence on Palestine & Transjordan
History Vault: FBI Confidential Files & Radical Politics in the U.S., 1945-1972
History Vault: Immigration Records of the INS, 1880-1930
History Vault: Japanese American Incarceration: Records of the War Relocation Authority, 1942-1946
History Vault: Labor Unions in the U.S., 1862-1974: Knights of Labor, AFL, CIO & AFL-CIO
History Vault: Law & Society Since the Civil War: American Legal Manuscripts from Harvard Law School
History Vault: Margaret Sanger Papers
History Vault: NAACP Papers: Board of Directors, Annual Conferences, Major Speeches, and National Staff Files, NAACP 1

History Vault: NAACP Papers: Branch Department, Branch Files and Youth Department Files, NAACP 6
History Vault: NAACP Papers: NAACP's Major Campaigns - Education, Voting, Housing, Employment, Armed Forces, NAACP 2
History Vault: NAACP Papers: NAACP's Major Campaigns - Legal Department Files, NAACP 4
History Vault: NAACP Papers: Special Subjects, NAACP 5
History Vault: NAACP Papers: The NAACP's Major Campaigns: Scottsboro, Anti-Lynching, Criminal, NAACP3
History Vault: Nazi Looted Art & Assets: Records on the Post-World War II Restitution Process
History Vault: New Deal and World War II: President Roosevelt's Office Files & Records of Federal Agencies
History Vault: OSS & State Department Intelligence and Research Reports, 1941-1961
History Vault: Pinkerton's National Detective Agency Records
History Vault: Progressive Era: Reform, Regulation, and Rights
History Vault: Progressive Era: Robert M. La Follette Papers
History Vault: Progressive Era: Voices of Reform
History Vault: Reconstruction & Military Government after the Civil War 1865-1877
History Vault: Records of the Children's Bureau, 1912-1969
History Vault: Revolutionary War & Early America: MHS Collections
History Vault: SDS, Vietnam Veterans Against the War & the Anti-Vietnam War Movement
History Vault: Slavery & the Law
History Vault: Slavery in Antebellum Southern Industries
History Vault: Socialist Party of America Papers
History Vault: Southern Life & African American History, 1775-1915: Plantations Records, Part 1
History Vault: Southern Life & African American History, 1775-1915: Plantations Records, Part 2
History Vault: Struggle for Women's Rights: Organizational Records, 1880-1990
History Vault: Thomas A. Edison Papers
History Vault: U.S. Diplomatic Post Records, 1914-1945
History Vault: U.S. Military Intelligence Reports, 1911-1944
History Vault: Vietnam War & American Foreign Policy, 1960-1975
History Vault: Women at Work during World War II: Rosie the Riveter & the Women's Army Corps
History Vault: Women's Studies Manuscript Collections from the Schlesinger Library
History Vault: Workers, Labor Unions, & the American Left in C20: Federal Records
History Vault: World War I: British Foreign Office Political Correspondence
History Vault: World War I: Records of the American Expeditionary Forces & Diplomacy
History Vault: World War II: U.S. Documents on Planning, Operations, Intelligence, Axis War Crimes...
History Vault: American Indians & the American West, 1809-1971
HistoryMakers
Hospital Premium Collection
Hospital Premium Collection (Alumni)
Humanities Index
India Database
Indianapolis Star
International Bibliography of Art (IBA)

International Bibliography of the Social Sciences (IBSS)
International Index to Music Periodicals (IIMP)
International Newsstream
International Newsstream (Alumni)
Irish Women Poets of the Romantic Period
J.P. Morgan Research
Kafkas Werke
Latin America & Iberia Database
Latin American Newsstream
Latin American Newsstream (Alumni)
Latin American Women Writers
Latino Literature: Poetry, Drama, and Fiction
Legislative Insight Digital Archive 2013
Legislative Insight Digital Archive 2014
Legislative Insight Digital Archive 2015
Legislative Insight Digital Archive 2016
Legislative Insight Digital Archive 2017
Legislative Insight Digital Archive 2018
Legislative Insight Major Laws
Legislative Insight Part A
Legislative Insight Part B
Legislative Insight Prospective 2013
LGBT Magazine Archive
Library & Information Science Abstracts (LISA)
Library & Information Science Collection
Library Science Database
Linguistics and Language Behavior Abstracts (LLBA)
Linguistics Collection
Linguistics Database
Literature Online (LION)
Literature Online (LION) – US Customers Only
Literature Online (LION) Premium
Literature Online (LION) Premium - US Customers Only
Los Angeles Times
Los Angeles Times (Alumni)
Market Research PRO
Materials Business File
Materials Research Database
Materials Science & Engineering Collection
Materials Science Collection

Materials Science Database
Materials Science Index
Medical Database
Medical Database (Alumni)
MEDLINE with Full Text
MEDLINE®
Men's Magazine Archive
METADEX
Meteorological & Geoastrophysical Abstracts
Middle East & Africa Database
Middle East & African Newsstream
Military Database
Military Database (Alumni)
Music & Performing Arts Collection
Music Periodicals Database
Natural Science Collection
New York Times
News PRO
Newsday
Newsday (Alumni)
Nineteenth-Century Fiction
Nursing & Allied Health Database
Nursing & Allied Health Database (Alumni)
Oceanic Abstracts
PAIS Index
Performing Arts Periodicals Database
Periodicals Archive Online Collection 0
Periodicals Archive Online Collection 1
Periodicals Archive Online Collection 1.2
Periodicals Archive Online Collection 10
Periodicals Archive Online Collection 2
Periodicals Archive Online Collection 2.2
Periodicals Archive Online Collection 3.1
Periodicals Archive Online Collection 3.2
Periodicals Archive Online Collection 3.3
Periodicals Archive Online Collection 3.4
Periodicals Archive Online Collection 4
Periodicals Archive Online Collection 5
Periodicals Archive Online Collection 6
Periodicals Archive Online Collection 7

Periodicals Archive Online Collection 8
Periodicals Archive Online Collection 9
Periodicals Archive Online English Literature Collection
Periodicals Archive Online Foundation Collection
Periodicals Archive Online Foundation Collection 2
Periodicals Archive Online Foundation Collection 3
Periodicals Archive Online Historical Studies Collection
Periodicals Archive Online History Collection
Periodicals Archive Online JISC Collection
Periodicals Archive Online JISC Collection 2
Periodicals Archive Online JSTOR Titles
Periodicals Archive Online Liberal Arts Collection 1
Periodicals Archive Online Liberal Arts Collection 2
Periodicals Archive Online Liberal Arts Collection 3
Periodicals Archive Online Liberal Arts Collection 4
Periodicals Archive Online Literary Studies Collection
Periodicals Archive Online Philosophy & Religion Collection
Periodicals Archive Online Spanish Language Collection
Periodicals Index Online
Periodicals Index Online Español
Pharma and Biotech Premium PRO
Pharmaceutical News Index
Pharmaceutical News Index (Alumni)
Philosophy Collection
Philosophy Database
Policy File Index
Political Science Database
Politics Collection
Pollution Abstracts
PRISMA Database
PRISMA Database with HAPI Index
ProQuest American Statistics PDF Service
ProQuest Biological Science Journals
ProQuest Central
ProQuest Central (Alumni)
ProQuest Central China
ProQuest Central Essentials
ProQuest Central Korea
ProQuest Central Student
ProQuest Central UK/Ireland

ProQuest Civil War Era
ProQuest Computer Science Journals
ProQuest Congressional and Executive Base
ProQuest Congressional Hearings Digital Collection Unpublished Hearings Collection A (House 1973-1979)
ProQuest Congressional Hearings Digital Collection Unpublished Hearings Collection B (House 1980, Senate 1985-1990)
ProQuest Congressional Hearings Digital Collection Unpublished Hearings Collection C (House 1981-1982, Senate 1991-1992)
ProQuest Congressional Hearings Digital Collection: Part A (1824-1979)
ProQuest Congressional Hearings Digital Collection: Part B (1980-2003)
ProQuest Congressional Hearings Digital Collection: Part C (2004-2010)
ProQuest Congressional Hearings Digital Collection: Part D (2011 forward)
ProQuest Congressional Hearings Digital Collection: Part D (2011)
ProQuest Congressional Hearings Digital Collection: Part E (2012)
ProQuest Congressional Hearings Digital Collection: Part F (2013)
ProQuest Congressional Hearings Digital Collection: Part G (2014)
ProQuest Congressional Hearings Digital Collection: Part H (2015)
ProQuest Congressional Hearings Digital Collection: Part I (2016)
ProQuest Congressional Hearings Digital Collection: Part J (2017)
ProQuest Congressional Hearings Digital Collection: Part K (2018)
ProQuest Congressional Hearings Digital Collection: Part L (2019)
ProQuest Congressional Hearings Digital Collection: Part M (2020)
ProQuest Congressional Record Permanent Digital Collection Part A (Includes Predecessors) (1789-1997)
ProQuest Congressional Record Permanent Digital Collection Part B (1998-2001)
ProQuest Congressional Record Permanent Digital Collection Part C (2002-2005)
ProQuest Congressional Record Permanent Digital Collection Part D (2006-2009)
ProQuest Congressional Research Digital Collection: Part A (1830-2003)
ProQuest Congressional Research Digital Collection: Part B (2004-2010)
ProQuest Congressional Research Digital Collection: Part C (2011 forward)
ProQuest Congressional Research Digital Collection: Part C (2011)
ProQuest Congressional Research Digital Collection: Part D (2012)
ProQuest Congressional Research Digital Collection: Part E (2013)
ProQuest Congressional Research Digital Collection: Part F (2014)
ProQuest Congressional Research Digital Collection: Part G (2015)
ProQuest Congressional Research Digital Collection: Part H (2016)
ProQuest Congressional Research Digital Collection: Part I (2017)
ProQuest Congressional Research Digital Collection: Part J (2018)
ProQuest Congressional Research Digital Collection: Part K (2019)
ProQuest Congressional: Digital U.S. Bills and Resolutions Prospective, 2016
ProQuest Congressional: U.S. Serial Set 2 Digital Collection, Part L (2018)
ProQuest Congressional: U.S. Serial Set 2 Digital Collection, Part M (2019)
ProQuest Congressional: U.S. Serial Set Digital Collection 1 (1789-1969)

ProQuest Congressional: U.S. Serial Set Digital Collection 2, Part A (1970-1979)
ProQuest Congressional: U.S. Serial Set Digital Collection 2, Part B (1980-1989)
ProQuest Congressional: U.S. Serial Set Digital Collection 2, Part C (1990-2003)
ProQuest Congressional: U.S. Serial Set Digital Collection 2, Part D (2004-2010)
ProQuest Congressional: U.S. Serial Set Digital Collection 2, Part E (2011 onwards)
ProQuest Congressional: U.S. Serial Set Digital Collection 2, Part E (2011)
ProQuest Congressional: U.S. Serial Set Digital Collection 2, Part F (2012)
ProQuest Congressional: U.S. Serial Set Digital Collection 2, Part G (2013)
ProQuest Congressional: U.S. Serial Set Digital Collection 2, Part H (2014)
ProQuest Congressional: U.S. Serial Set Digital Collection 2, Part I (2015)
ProQuest Congressional: U.S. Serial Set Digital Collection 2, Part J (2016)
ProQuest Congressional: U.S. Serial Set Digital Collection 2, Part K (2017)
ProQuest Congressional: U.S. Serial Set Digtal Collection 2, Part N (2020)
ProQuest Congressional: U.S. Serial Set Maps Digital Collection
ProQuest Congressional: Unpublished Hearings Digital Collection A (1973-1979)
ProQuest Congressional: Unpublished Hearings Digital Collection B (House 1980, Senate 1985-1990)
ProQuest Digital Complete Prospective 2016-forward
ProQuest Digital Complete Prospective 2017-forward
ProQuest Digital Complete Prospective 2018-forward
ProQuest Digital Complete Prospective 2019-forward
ProQuest Digital U.S. Bills and Resolutions 2017
ProQuest Digital U.S. Bills and Resolutions 2018
ProQuest Digital U.S. Bills and Resolutions 2019
ProQuest Dissertations & Theses A&I
ProQuest Dissertations & Theses Global
ProQuest Dissertations & Theses Global A&I: The Humanities and Social Sciences Collection
ProQuest Dissertations & Theses Global A&I: The Sciences and Engineering Collection
ProQuest Dissertations & Theses Global: The Humanities and Social Sciences Collection
ProQuest Dissertations & Theses Global: The Sciences and Engineering Collection
ProQuest Dissertations and Theses Professional
ProQuest Economist Intelligence Unit Country Reports Archive
ProQuest Executive Branch Documents, 1789-1932
ProQuest Executive Branch Documents, Part 2
ProQuest Executive Branch Documents, Part 3 (1940-1942)
ProQuest Executive Branch Documents, Part 4 (1943-1945)
ProQuest Executive Branch Documents, Part 5 (1946-1948)
ProQuest Executive Orders and Presidential Proclamations 1789-1979
ProQuest Executive Orders and Presidential Proclamations, 1789-Present
ProQuest Historic Digital US Bills & Resolutions 1789-1979
ProQuest Historical Newspapers: Alabama Collection

ProQuest Historical Newspapers: American Hebrew Jewish Messenger
ProQuest Historical Newspapers: Atlanta Daily World
ProQuest Historical Newspapers: Black Newspaper Collection
ProQuest Historical Newspapers: Boston Globe
ProQuest Historical Newspapers: Calgary Herald
ProQuest Historical Newspapers: Chicago Defender
ProQuest Historical Newspapers: Chicago Tribune
ProQuest Historical Newspapers: Chicago Tribune
ProQuest Historical Newspapers: Chicago Tribune (1849-2010)
ProQuest Historical Newspapers: Chinese Newspapers Collection
ProQuest Historical Newspapers: Cleveland Call and Post
ProQuest Historical Newspapers: Communist Historical Newspaper Collection
ProQuest Historical Newspapers: Dayton Daily News
ProQuest Historical Newspapers: Detroit Free Press
ProQuest Historical Newspapers: Detroit Free Press (1831-1922)
ProQuest Historical Newspapers: Detroit Free Press (1923-1999)
ProQuest Historical Newspapers: Hartford Courant
ProQuest Historical Newspapers: Hartford Courant (1764-1922)
ProQuest Historical Newspapers: Hartford Courant (1923-1990)
ProQuest Historical Newspapers: Indianapolis Star
ProQuest Historical Newspapers: Indianapolis Star (1903-1922)
ProQuest Historical Newspapers: Indianapolis Star (1923-2004)
ProQuest Historical Newspapers: International Collection
ProQuest Historical Newspapers: Jewish Advocate
ProQuest Historical Newspapers: Leader-Post
ProQuest Historical Newspapers: Leftist Newspapers and Periodicals
ProQuest Historical Newspapers: Los Angeles Sentinel
ProQuest Historical Newspapers: Los Angeles Times
ProQuest Historical Newspapers: Los Angeles Times (1881-1922)
ProQuest Historical Newspapers: Los Angeles Times (1881-2010)
ProQuest Historical Newspapers: Louisiana Collection
ProQuest Historical Newspapers: Louisville Courier Journal
ProQuest Historical Newspapers: Louisville Courier Journal (1830-1922)
ProQuest Historical Newspapers: Louisville Courier Journal (1923-2000)
ProQuest Historical Newspapers: Michigan Chronicle
ProQuest Historical Newspapers: Michigan Collection
ProQuest Historical Newspapers: Midland Newspapers
ProQuest Historical Newspapers: Minneapolis Star Tribune
ProQuest Historical Newspapers: Minneapolis Tribune
ProQuest Historical Newspapers: Mississippi Collection

ProQuest Historical Newspapers: Montreal Gazette
ProQuest Historical Newspapers: Nevada Collection
ProQuest Historical Newspapers: New York Amsterdam News
ProQuest Historical Newspapers: New York Collection
ProQuest Historical Newspapers: New York Tribune
ProQuest Historical Newspapers: New York Tribune / Herald Tribune
ProQuest Historical Newspapers: Newsday
ProQuest Historical Newspapers: Norfolk Journal and Guide
ProQuest Historical Newspapers: Ohio Collection
ProQuest Historical Newspapers: Ottawa Citizen
ProQuest Historical Newspapers: Pennsylvania Collection
ProQuest Historical Newspapers: Philadelphia Tribune
ProQuest Historical Newspapers: Pittsburgh Courier
ProQuest Historical Newspapers: Pittsburgh Post-Gazette
ProQuest Historical Newspapers: San Francisco Chronicle
ProQuest Historical Newspapers: South China Morning Post
ProQuest Historical Newspapers: St. Louis Post Dispatch
ProQuest Historical Newspapers: St. Louis Post Dispatch (1923-2003)
ProQuest Historical Newspapers: St. Louis Post-Dispatch (1874-1922)
ProQuest Historical Newspapers: St. Petersburg Times / Tampa Bay Times
ProQuest Historical Newspapers: The American Israelite
ProQuest Historical Newspapers: The Arizona Republican
ProQuest Historical Newspapers: The Arizona Republican (1890-1922)
ProQuest Historical Newspapers: The Arizona Republican (1923-2007)
ProQuest Historical Newspapers: The Atlanta Constitution
ProQuest Historical Newspapers: The Atlanta Constitution
ProQuest Historical Newspapers: The Austin American Statesman
ProQuest Historical Newspapers: The Baltimore Afro-American
ProQuest Historical Newspapers: The Baltimore Sun
ProQuest Historical Newspapers: The Baltimore Sun
ProQuest Historical Newspapers: The Boston Globe
ProQuest Historical Newspapers: The Boston Globe
ProQuest Historical Newspapers: The Christian Science Monitor
ProQuest Historical Newspapers: The Cincinnati Enquirer
ProQuest Historical Newspapers: The Cincinnati Enquirer (1841-1922)
ProQuest Historical Newspapers: The Cincinnati Enquirer (1923-2009)
ProQuest Historical Newspapers: The Globe and Mail
ProQuest Historical Newspapers: The Guardian and The Observer
ProQuest Historical Newspapers: The Guardian and The Observer
ProQuest Historical Newspapers: The Irish Times and The Weekly Irish Times

ProQuest Historical Newspapers: The Irish Times and The Weekly Irish Times
ProQuest Historical Newspapers: The Jerusalem Post
ProQuest Historical Newspapers: The Jewish Exponent
ProQuest Historical Newspapers: The Korea Times
ProQuest Historical Newspapers: The Nashville Tennessean
ProQuest Historical Newspapers: The Nashville Tennessean (1812-1922)
ProQuest Historical Newspapers: The Nashville Tennessean (1923-2002)
ProQuest Historical Newspapers: The New York Times
ProQuest Historical Newspapers: The New York Times
ProQuest Historical Newspapers: The New York Times (Alumni)
ProQuest Historical Newspapers: The New York Times with Index
ProQuest Historical Newspapers: The Philadelphia Inquirer
ProQuest Historical Newspapers: The Province
ProQuest Historical Newspapers: The Scotsman
ProQuest Historical Newspapers: The Scotsman
ProQuest Historical Newspapers: The Wall Street Journal
ProQuest Historical Newspapers: The Wall Street Journal
ProQuest Historical Newspapers: The Washington Post
ProQuest Historical Newspapers: The Washington Post (1877-1922)
ProQuest Historical Newspapers: Times Colonist
ProQuest Historical Newspapers: Times of India
ProQuest Historical Newspapers: Toronto Star
ProQuest Historical Newspapers: U.S. Jewish Newspaper Collection
ProQuest Historical Newspapers: U.S. Major Dailies
ProQuest Historical Newspapers: U.S. Metro Collection
ProQuest Historical Newspapers: U.S. Midwest Collection
ProQuest Historical Newspapers: U.S. North Central Collection
ProQuest Historical Newspapers: U.S. Northeast Collection
ProQuest Historical Newspapers: U.S. South Central Collection
ProQuest Historical Newspapers: U.S. Southeast Collection
ProQuest Historical Newspapers: U.S. West Collection
ProQuest Historical Newspapers: Vancouver Sun
ProQuest Historical Newspapers: Vermont Collection
ProQuest Historical Newspapers: Washington Collection
ProQuest Historical Newspapers: Windsor Star
ProQuest Historical Newspapers: Wisconsin Collection
ProQuest Historical Newspapers: Zeeland Record
ProQuest Indian Claims Insight
ProQuest International Statistics PDF Service
ProQuest Legislative Insight 1789-1979

ProQuest Legislative Insight 2018-forward
ProQuest Legislative Insight 2019
ProQuest Legislative Insight 2019-forward
ProQuest News Policy & Politics Magazine Archive
ProQuest Newsstand Professional
ProQuest One Business
ProQuest One Literature
ProQuest Pharma Collection
ProQuest Professional Education
ProQuest PsycBOOKS
ProQuest Recent Newspapers: Alabama Collection
ProQuest Recent Newspapers: American Banker
ProQuest Recent Newspapers: Barron's
ProQuest Recent Newspapers: Calgary Herald
ProQuest Recent Newspapers: Chicago Tribune
ProQuest Recent Newspapers: Delaware Collection
ProQuest Recent Newspapers: Detroit Free Press
ProQuest Recent Newspapers: Edmonton Journal
ProQuest Recent Newspapers: Florida Collection
ProQuest Recent Newspapers: Hartford Courant
ProQuest Recent Newspapers: Hawaii Collection
ProQuest Recent Newspapers: Indianapolis Star
ProQuest Recent Newspapers: Iowa Collection
ProQuest Recent Newspapers: Leader-Post
ProQuest Recent Newspapers: Los Angeles Times
ProQuest Recent Newspapers: Mississippi Collection
ProQuest Recent Newspapers: Montreal Gazette
ProQuest Recent Newspapers: National Post
ProQuest Recent Newspapers: Nevada Collection
ProQuest Recent Newspapers: New Jersey Collection
ProQuest Recent Newspapers: New York Collection
ProQuest Recent Newspapers: Newsday
ProQuest Recent Newspapers: North Carolina Collection
ProQuest Recent Newspapers: Ottawa Citizen
ProQuest Recent Newspapers: Pennsylvania Collection
ProQuest Recent Newspapers: Pittsburgh Post-Gazette
ProQuest Recent Newspapers: Saskatoon Star Phoenix
ProQuest Recent Newspapers: St. Louis Post-Dispatch
ProQuest Recent Newspapers: Star Tribune
ProQuest Recent Newspapers: Tampa Bay Times

ProQuest Recent Newspapers: The Atlanta Journal Constitution
ProQuest Recent Newspapers: The Baltimore Sun
ProQuest Recent Newspapers: The Boston Globe
ProQuest Recent Newspapers: The Cincinnati Enquirer
ProQuest Recent Newspapers: The Courier-Journal
ProQuest Recent Newspapers: The Guardian
ProQuest Recent Newspapers: The New York Times
ProQuest Recent Newspapers: The Philadelphia Inquirer
ProQuest Recent Newspapers: The Province
ProQuest Recent Newspapers: The Tennessean
ProQuest Recent Newspapers: The Vancouver Sun
ProQuest Recent Newspapers: The Wall Street Journal
ProQuest Recent Newspapers: The Washington Post
ProQuest Recent Newspapers: Times Colonist
ProQuest Recent Newspapers: Toronto Star
ProQuest Recent Newspapers: U.S. Major Dailies
ProQuest Recent Newspapers: U.S. Midwest Collection
ProQuest Recent Newspapers: U.S. North Central Collection
ProQuest Recent Newspapers: U.S. Northeast Collection
ProQuest Recent Newspapers: U.S. South Central Collection
ProQuest Recent Newspapers: U.S. Southeast Collection
ProQuest Recent Newspapers: U.S. West Collection
ProQuest Recent Newspapers: USA Today
ProQuest Recent Newspapers: Vermont Collection
ProQuest Recent Newspapers: Washington Collection
ProQuest Recent Newspapers: Windsor Star
ProQuest Statistical Abstract of the United States
ProQuest Statistical Abstracts of the World
ProQuest Statistical Business Content
ProQuest Statistical Insight - Tables
ProQuest Statistical Insight ASI Abstracts and Indexing
ProQuest Statistical Insight IIS Abstracts and Indexing
ProQuest Statistical Insight SRI Abstracts and Indexing
ProQuest Statistical Reference PDF Service
ProQuest Teacher Journals
ProQuest Visual Literacy
Psychology Database
Psychology Database (Alumni)
PTSDpubs
Public Health Database

Publicly Available Content Database
Religion Database
Religion Database (Alumni)
Religious Magazine Archive
Research Library
Research Library (Alumni)
Research Library China
Research Library Prep
Schillers Werke
Science Database
Science Database (Alumni)
SciTech Premium Collection
Scottish Women Poets of the Romantic Period
Screen Studies Collection
Senate Executive Documents and Reports
SIRS Discoverer
SIRS Issues Researcher
Social Science Database
Social Science Database (Alumni)
Social Science Premium Collection
Sociological Abstracts
Sociology Collection
Sociology Database
South and Southeast Asian Literature in English
Sports Medicine & Education Index
SS Low-Res Maps carto-bib
STEM Database
Supreme Court Insight 1933-1974
Supreme Court Insight 1975-1979
Supreme Court Insight 1975-2016
Supreme Court Insight 2017
Supreme Court Insight 2017-forward
Supreme Court Insight 2018
Supreme Court Insight 2019-2020 Term
Supreme Court Insight and Certiorari Denied Combined, 2019-forward
Supreme Court Insight Certiorari Denied, 2018-2019
Supreme Court Insight Certiorari Denied, 2018-forward
Supreme Court Insight Certiorari Denied, 2019_2020
Supreme Court Insight Certiorari Denied, Part 1, 1997-2017
Supreme Court Insight Certiorari Denied, Part 2, 1975-1996

Sustainability Science Abstracts
Teatro Español del Siglo de Oro
Technology Collection
The Annual Register: A Record of World Events
The Cecil Papers
The Christian Science Monitor
The Faber Poetry Library
The Globe and Mail
The GQ Archive
The Guardian
The Harper's Bazaar Archive
The Harper's Bazaar Archive (US edition only)
The Newsweek Archive
The Tennessean
The Times & The Sunday Times (London)
The Vogue Archive
The Vogue Italia Archive
The Wall Street Journal
The Washington Post
Toronto Star
TOXLINE
Trade PRO
Trench Journals and Unit Magazines of the First World War
Turkey Database
Twentieth Century American Poetry, Second Edition
Twentieth Century Drama
Twentieth-Century African American Poetry
Twentieth-Century American Poetry
Twentieth-Century English Poetry
U.K. Parliamentary Papers: House of Commons 18th Century (1688-1834)
U.K. Parliamentary Papers: House of Commons 19th Century (1801-1900)
U.K. Parliamentary Papers: House of Commons 20th Century (1901-2003/04 session)
U.K. Parliamentary Papers: House of Commons 21st Century, Module 1 (2004/05-2009/10 sessions)
U.K. Parliamentary Papers: House of Commons 21st Century, Module 2 (2010/12-2013/14 sessions)
U.K. Parliamentary Papers: House of Commons 21st Century, Module 3 (2015-2022)
U.K. Parliamentary Papers: House of Commons Hansard (1803-2005)
U.K. Parliamentary Papers: House of Commons Public Petitions to Parliament, 1833-1918
U.K. Parliamentary Papers: House of Lords, 1800-1910
U.S. Hispanic Newsstream
U.S. Major Dailies

U.S. Major Dailies (Alumni)
U.S. Midwest Newsstream
U.S. Newsstream
U.S. Newsstream (Alumni)
U.S. North Central Newsstream
U.S. Northeast Newsstream
U.S. Northeast Newsstream (Alumni)
U.S. South Central Newsstream
U.S. Southeast Newsstream
U.S. Statistical Abstract: 1878-1928
U.S. Statistical Abstract: 1929-1969
U.S. Statistical Abstract: 1970-2012
U.S. West Newsstream
UBM Computer Full Text
UK & Ireland Database
USA Today
Water Resources Abstracts
Women's Magazine Archive I
Women's Magazine Archive II
Women's Wear Daily Archive
Worldwide Political Science Abstracts
Youth and Popular Culture Magazine Archive
American Psychiatric Publishing Books and Guidelines
American Psychiatric Publishing Journals
Psychiatry Legacy Collection
Psychoanalytic Electronic Publishing Journals
Psychotherapy.net Exclusive Collection
Psychotherapy.net Full Collection
Psychotherapy.net Social Work Collection
Psychotherapy.net Standard Essential Collection
Psychotherapy.net Standard Expanded Collection
HOGREFE PsyJournals
Public Information Online
PLoS Biology
PLoS Currents
PLoS Medicine
Public Library of Science (PLoS) Journals Open Access
Publishers Weekly Digital Archive
PubMed Central
Pulsus

Purdue University Press Journals
Pushpa Publishing House journals
IBUK Libra
QScience Free
Queen's University QSpace
Quintessence Journals
Quintessenz Online Zeitschriften
R2 Digital Library
RACO Revistes Catalanes amb Accés Obert
Radiation Research Society Resources
Radiological Society of North America
Radiology Legacy Collection
RAND Publications
RAND Reports
Rapra Technology Journals
African History and Culture Imprints from the LCP (1540-1921)
Afro-Americana Imprints, 1535-1922
American Broadsides and Ephemera, Series I, 1760-1900
American Civil War Collection, 1860-1922: From the American Antiquarian Society
American Pamphlets Series I 1820-1922: From the New York Historical Society
American Slavery Collection, 1820-1922: From the American Antiquarian Society
Caribbean History and Culture, 1535-1920
Early American Imprints, Series I: Evans, 1639-1800
Early American Imprints, Series II: Shaw-Shoemaker, 1801-1819
Early American Imprints, Series II: Supplement from the American Antiquarian Society, 1801-1819
Joint Publications Research Service (JPRS) Reports, 1957-1994
U.S. Congressional Serial Set, 1817-1980
U.S. Congressional Serial Set, 1817-1980 (DFG Nationallizenzen)
U.S. Congressional Serial Set, 1817-1994
U.S. Congressional Serial Set, 1981-1994
Remedica Journals
Reprints Desk
RePEc
RePEc IDEAS
Research Repository UCD
ResearchOnline
Reserve Bank of Australia Bulletin
Revel@Nice
Revistas científicas del CSIC
Revistas Medicas Cubana

Rinton Press Journals
R2 Digital Library PDA Discoverable Titles
Rivisteweb
RMIT University Research Repository
Rock''s Backpages
Rocky Mountain Mathematics Consortium Journals
English Historical Documents
Routledge religion online
Architecture Media
Clinical Practice Guidelines
Royal College of Art Institutional Repository - Open Access
Royal College of Surgeons of England Publications
Royal Historical Society Bibliography
Royal Irish Academy Publications
PJ Online
AtoM @ RRU
DSpace @ RRU
KB+ BIBSAM Royal Society Publishing Journals 2014-2016
Analytical Science Collection
CRKN Royal Society of Chemistry Journals Gold
KB+ JISC Collections Royal Society Of Chemistry Gold 2016
KB+ JISC Collections Royal Society Of Chemistry Gold 2017-19 Collectin Option 1
KB+ JISC Collections Royal Society Of Chemistry Gold 2017-2019 Collection Option 3
KB+ Royal Society of Chemistry: JISC Collections:Journals Archive 2005-2007
NESLi2 Royal Society of Chemistry Gold Journals
Royal Society of Chemistry
Royal Society of Chemistry Archive
Royal Society of Chemistry eBook Collection 2007
Royal Society of Chemistry eBook Collection 2008
Royal Society of Chemistry eBook Collection 2009
Royal Society of Chemistry eBook Collection 2010
Royal Society of Chemistry eBook Collection 2011
Royal Society of Chemistry eBook Collection 2012
Royal Society of Chemistry eBook Collection 2013
Royal Society of Chemistry eBook Collection 2014
Royal Society of Chemistry eBook Collection 2015
Royal Society of Chemistry eBook Collection 2017
Royal Society of Chemistry eBook Collection 2018
Royal Society of Chemistry eBook Collection 2020
Royal Society of Chemistry eBook Collection Archive

Royal Society of Chemistry eBook Collection Complete
Royal Society of Chemistry Historical Collection
Royal Society of Chemistry Journals
Royal Society of Chemistry Journals Archive (1841-2004)
Royal Society Of Chemistry Package Gold
Royal Society of New Zealand Subscribe
Royal Society Open Access Journals
Royal Society Package S
Royal Society Publishing
KB+ BIBSAM Collections Royal College Of Nursing Journals 2017-2019
KB+ JISC Collections Royal College Of Nursing Archive
Royal College of Nursing (RCNi) Journals
Specialist Nursing Journals
Zhurnalnyĭ zal
RULA Digital Repository
Hirzel eLibrary
Sabinet African Journals Business & Finance Collection
Sabinet African Journals Core Collection
Sabinet African Journals Law Collection
Sabinet African Journals Medicine & Health Collection
Sabinet African Journals Religion Collection
Sabinet African Journals Science, Technology & Agriculture Collection
Sabinet African Journals Social Sciences & Humanities Collection
Sabinet Open Access Journals
African Journal Archive
SAE Aerospace Material Standards (Current)
SAE Aerospace Material Standards (Historical)
SAE Aerospace Standards (Current)
SAE Aerospace Standards (Historical)
SAE All Standards Current (GV, AMS, AS)
SAE All Standards Historical (GV, AMS, AS)
SAE EDGE™ Research Reports - 2019
SAE EDGE™ Research Reports - 2020
SAE EDGE™ Research Reports - 2021
SAE EDGE™ Research Reports - Full collection
SAE Ground Vehicle Standards (Current)
SAE Ground Vehicle Standards (Historical)
SAE Mobilus Technical Papers
SAE Technical Papers, 1998-Current
SAE Technical Papers, Back File A (1990 - 1997)

SAE Technical Papers, Back File B (1980-1989)
SAE Technical Papers, Back File C (1964-1979)
SAE Technical Papers, Back File D (1906-1963)
SAE International Journals
Sagamore Publishing Journals
Bacon Sage Publications Couperin Hss
Data Planet Statistical Datasets BASIC
Data Planet Statistical Datasets IMFUSERS
Data Planet Statistical Datasets Premium CHINA
Data Planet Statistical Datasets Premium EASI
Data Planet Statistical Datasets Premium IEM
Data Planet Statistical Datasets Premium INFOGROUP
Data-Planet Statistical Datasets
KB+ BIBSAM Sage Premier 2014-2016
KB+ BIBSAM Sage Premier 2017-2019
KB+ BIBSAM Sage The Royal Society Of Medicine Journals 2014-2016
KB+ BIBSAM Sage The Royal Society Of Medicine Journals 2017-2019
KB+ JISC Collections Sage IMechE Journals 2017-2018
KB+ JISC Collections Sage Premier 2015
KB+ JISC Collections SAGE Premier 2017-2018
KB+ Jisc Collections SAGE Premier 2019
KB+ JISC Collections Sage The Royal Society Of Medicine 2017-2018
KB+ WHEEL SAGE Premier 2016
KB+ WHEEL SAGE Premier 2017-2018
KESLI Premier Set
Sage Books And Reference Criminology Collection Supplement 2015
Sage Books And Reference Education Collection Supplement 2015
Sage Books And Reference Geography Collection Supplement 2015
Sage Books And Reference Health And Social Care Collection Supplement 2015
Sage Books And Reference Psychology Collection Supplement 2015
Sage Books And Reference Sociology Collection Supplement 2015
Sage Books Business And Management Collection Supplement 2015
Sage Books Counselling And Psychotherapy Collection Supplement 2015
Sage Books Criminology Collection Supplement 2015
Sage Books Education Collection Supplement 2015
Sage Books Geography Collection Supplement 2015
Sage Books Health And Social Care Collection Supplement 2015
Sage Books Media And Communication Collection Supplement 2015
Sage Books Psychology Collection Supplement 2015
Sage Books Sociology Collection Supplement 2015

SAGE Business Cases Essentials 2016-2018
SAGE Cardiology & Cardiovascular Medicine Subject Collection
SAGE CBUC 2020
SAGE Clinical Medicine 2020
SAGE Clinical Medicine Package 2007
SAGE Clinical Medicine Package 2008
SAGE Clinical Medicine Package 2009
SAGE Clinical Medicine Package 2010
SAGE Clinical Medicine Package 2011
SAGE Clinical Medicine Package 2012
SAGE Clinical Medicine Package 2013
SAGE Clinical Medicine Package 2014
SAGE Clinical Medicine Package 2015
SAGE Clinical Medicine Package 2016
SAGE Clinical Medicine Package 2017
SAGE Clinical Medicine Package 2018
SAGE Clinical Medicine Package 2019
SAGE Clinical Medicine Shallow Backfile 2 2020
SAGE Clinical Medicine Shallow Backfile 2017
SAGE Clinical Medicine Shallow Backfile 2020 Purchase
SAGE CNPeReading Journals
SAGE Collections Plus 2020
SAGE Communication and Media Studies Collection
SAGE Communication FTC
SAGE Communication Subject Collection Backfile Purchase
SAGE Community College Subject Collection
SAGE Community College Subject Collection Backfile
SAGE Complete
Sage Complete Books Collection Supplement 2015
SAGE Complete Books, Reference and Navigator Collection 2017
SAGE Complete Books, Reference and Navigator Collection 2018
Sage Complete Deep Backfile 2016
SAGE Complete Deep Backfile 2020
SAGE Complete Deep Backfile Purchase 2007
SAGE Complete Deep Backfile Purchase 2008
SAGE Complete Deep Backfile Purchase 2009
SAGE Complete Deep Backfile Purchase 2010
SAGE Complete Deep Backfile Purchase 2011
SAGE Complete Deep Backfile Purchase 2012
SAGE Complete Deep Backfile Purchase 2013

SAGE Complete Deep Backfile Purchase 2014
SAGE Complete Deep Backfile Purchase 2015
SAGE Complete Deep Backfile Purchase 2017
SAGE Complete Deep Backfile Purchase 2018
SAGE CQ Press Annual Collection
SAGE CQ Press Annual Collection 2017
SAGE CQ Press Annual Collection 2018
SAGE CQ Press Congress Collection
SAGE CQ Press Historic Documents
SAGE CQ Press Political Handbook of the World
SAGE CQ Press Politics in America
SAGE CQ Press Public Affairs Collection
SAGE CQ Press Researcher Online
Sage CQ Press Supreme Court Yearbook
SAGE CQ Press Vital Statistics on American Politics
SAGE CQ Press Washington Information Directory
SAGE CQ Press Weekly
SAGE Criminology Collection
SAGE Criminology FTC
Sage CRKN Collection
SAGE CSIC Cross Access Package 2008
SAGE Deep Backfile Clinical Medicine 2020
SAGE Deep Backfile Clinical Medicine Purchase 2012
SAGE Deep Backfile Clinical Medicine Purchase 2013
SAGE Deep Backfile Clinical Medicine Purchase 2014
SAGE Deep Backfile Clinical Medicine Purchase 2015
SAGE Deep Backfile Clinical Medicine Purchase 2016
SAGE Deep Backfile Clinical Medicine Purchase 2017
SAGE Deep Backfile Clinical Medicine Purchase 2018
SAGE Deep Backfile Health Sciences 2020
SAGE Deep Backfile Health Sciences Purchase 2012
SAGE Deep Backfile Health Sciences Purchase 2013
SAGE Deep Backfile Health Sciences Purchase 2014
SAGE Deep Backfile Health Sciences Purchase 2015
SAGE Deep Backfile Health Sciences Purchase 2016
SAGE Deep Backfile Health Sciences Purchase 2017
SAGE Deep Backfile Health Sciences Purchase 2018
SAGE Deep Backfile Health Sciences Purchase 2019
SAGE Deep Backfile HSS 2020
SAGE Deep Backfile HSS Purchase 2011

SAGE Deep Backfile HSS Purchase 2012
SAGE Deep Backfile HSS Purchase 2013
SAGE Deep Backfile HSS Purchase 2014
SAGE Deep Backfile HSS Purchase 2015
SAGE Deep Backfile HSS Purchase 2016
SAGE Deep Backfile HSS Purchase 2017
SAGE Deep Backfile HSS Purchase 2018
SAGE Deep Backfile STM 2020
SAGE Deep Backfile STM Purchase 2008
SAGE Deep Backfile STM Purchase 2009
SAGE Deep Backfile STM Purchase 2010
SAGE Deep Backfile STM Purchase 2011
SAGE Deep Backfile STM Purchase 2012
SAGE Deep Backfile STM Purchase 2013
SAGE Deep Backfile STM Purchase 2014
SAGE Deep Backfile STM Purchase 2015
SAGE Deep Backfile STM Purchase 2016
SAGE Deep Backfile STM Purchase 2017
SAGE Deep Backfile STM Purchase 2018
SAGE Deep Backfile Upgrade 2020
SAGE Deep Backfile Upgrade Purchase 2008
SAGE Deep Backfile Upgrade Purchase 2009
SAGE Deep Backfile Upgrade Purchase 2010
SAGE Deep Backfile Upgrade Purchase 2011
SAGE Deep Backfile Upgrade Purchase 2012
SAGE Deep Backfile Upgrade Purchase 2013
SAGE Deep Backfile Upgrade Purchase 2014
SAGE Deep Backfile Upgrade Purchase 2015
SAGE Deep Backfile Upgrade Purchase 2016
SAGE Deep Backfile Upgrade Purchase 2017
SAGE Deep Backfile Upgrade Purchase 2018
SAGE Education Collection
SAGE Education FTC
SAGE Education Subject Collection Backfile Purchase
SAGE Engineering Custom Collection
SAGE Full-Text Collections
SAGE Health and Medicine Custom Collection
SAGE Health Practice & Services Subject Collection
SAGE Health Practice & Services Subject Collection Backfile Purchase
SAGE Health Science Shallow Backfile 2 2020

SAGE Health Sciences 2020
SAGE Health Sciences Backfile Upgrade Purchase 2017
SAGE Health Sciences Backfile Upgrade Purchase 2018
SAGE Health Sciences Package 2008
SAGE Health Sciences Package 2009
SAGE Health Sciences Package 2010
SAGE Health Sciences Package 2011
SAGE Health Sciences Package 2012
SAGE Health Sciences Package 2013
SAGE Health Sciences Package 2014
SAGE Health Sciences Package 2015
SAGE Health Sciences Package 2016
SAGE Health Sciences Package 2017
SAGE Health Sciences Package 2018
SAGE Health Sciences Package 2019
Sage Health Sciences Package Without RSM 2015
SAGE Health Sciences Shallow Backfile 2017
SAGE Health Sciences without RSM 2020
SAGE HR Package Backfile
SAGE HR Subject Collection
SAGE HSS 2020
Sage HSS Backfile 2016
SAGE HSS Backfile Upgrade 2020
SAGE HSS Backfile Upgrade Purchase 2013
SAGE HSS Backfile Upgrade Purchase 2014
SAGE HSS Backfile Upgrade Purchase 2015
SAGE HSS Backfile Upgrade Purchase 2016
SAGE HSS Backfile Upgrade Purchase 2017
SAGE HSS Backfile Upgrade Purchase 2018
SAGE HSS Backfile Upgrade Purchase 2019
SAGE HSS Package 2007
SAGE HSS Package 2008
SAGE HSS Package 2009
SAGE HSS Package 2010
SAGE HSS Package 2011
SAGE HSS Package 2012
SAGE HSS Package 2013
SAGE HSS Package 2014
SAGE HSS Package 2015
SAGE HSS Package 2017

SAGE HSS Package 2018
SAGE HSS Package 2019
SAGE HSS Shallow Backfile 2 2020
SAGE HSS Shallow Backfile 2017
SAGE Humanities and Social Science Backfile Package 2008
SAGE Humanities and Social Science Backfile Package 2009
SAGE Humanities and Social Science Backfile Package 2010
SAGE IMechE Archive
SAGE IMechE Complete Collection With Archive
SAGE IMechE Complete Collection without Archive
SAGE IMechE Journals - NESLI2 2012
SAGE IMechE Proceedings Collection
SAGE IMechE Proceedings Collection With Archive
SAGE IMechE Shallow Backfile
SAGE JOURNALS HSS 2016
SAGE Journals Medico-Legal Subject Collection Backfile
Sage Journals Online Deep Backfile (DFG Nationallizenzen)
Sage Journals Open Access Journals
SAGE Knowledge A-Z (All Titles)
SAGE Knowledge Books and Reference
SAGE Knowledge Books Collection
SAGE Knowledge Business And Management Collection
SAGE Knowledge Counselling And Psychotherapy Collection
Sage Knowledge Cq Press Annual Collection 2016
SAGE Knowledge Criminology Collection
SAGE Knowledge Education Collection
SAGE Knowledge Geography Collection
SAGE Knowledge Health And Social Care Collection
SAGE Knowledge Media And Communication Collection
SAGE Knowledge Politics And International Relations Collection
SAGE Knowledge Psychology Collection
Sage Knowledge Reference 2000-2011 Handbook Backlist Collection
SAGE Knowledge Reference Collection
SAGE Knowledge Sociology Collection
SAGE Knowledge Video
SAGE Knowledge Video - Business & Management Collection
SAGE Knowledge Video - Counseling & Psychotherapy Collection
SAGE Knowledge Video - Education Collection
SAGE Knowledge Video - Media & Communication Collection
SAGE Knowledge Video - Politics & International Relations Collection

SAGE Knowledge Video - Psychology Collection
SAGE Knowledge Video: Business and Management
SAGE Knowledge Video: Education
SAGE Knowledge Video: Political Science & International Relations Collection 2020
SAGE Management & Org Studies FTC
SAGE Management & Org Studies Subject Collection
SAGE Management & Organization Studies Subject Collect Backfile Purchase
SAGE Management and Organisation Studies Collection
SAGE Materials Science and Engineering Collection
SAGE Materials Science and Engineering Collection without I Mech E
SAGE Materials Science FTC
SAGE Materials Science Subject Collection without iMechE
SAGE Medico-Legal Subject Collection
SAGE Medico-Legal Subject Collection Backfile Purchase
SAGE Mental Health Subject Collection
SAGE Neurology Subject Collection
SAGE Neurology Subject Collection Backfile Purchase
SAGE Nursing & Health Sciences FTC
SAGE Nursing & Public Health Subject Collection Backfile Purchase
SAGE Nursing and Public Health Collection
SAGE Oncology Subject Collection
SAGE Oncology Subject Collection Backfile Purchase
SAGE Open Access 2014 - NESLI2
SAGE Orthopaedics & Sports Medicine Subject Collection Backfile Purchase
SAGE Orthopaedics and Sports Medicine Collection
SAGE Palliative Medicine & Chronic Care Subject Collect Backfile Purchase
SAGE Palliative Medicine & Chronic Care Subject Collection
SAGE Pediatrics Subject Collection
SAGE Pediatrics Subject Collection Backfile Purchase
SAGE Pharmacology & Biomedical Subject Collection
SAGE Pharmacology & Biomedical Subject Collection Backfile Purchcase
SAGE Politics & Int Relations Subject Collection
SAGE Politics & International Relations Subject Coll Backfile Purchase
SAGE Politics and International Relations Collection
SAGE Premier 2006
SAGE Premier 2007
SAGE Premier 2007 Without Aera
SAGE Premier 2008
SAGE Premier 2009
SAGE Premier 2010

SAGE Premier 2011
SAGE Premier 2012
Sage Premier 2012 - SURFMarket
SAGE Premier 2013
SAGE Premier 2014
SAGE Premier 2015
SAGE Premier 2015 - SURFMarket
Sage Premier 2016
Sage Premier 2017
SAGE Premier 2018
SAGE Premier 2019
SAGE Premier 2020
SAGE Psychology Collection
SAGE Psychology FTC
SAGE Psychology Subject Collection Backfile Purchase
SAGE Reference 2000-2010 Handbook Backlist Collection
Sage Reference Online 2005-2008 Handbook Upgrade Collection
SAGE Reference Online 2007 Encyclopedia Collection
SAGE Reference Online 2008 Encyclopedia Collection
SAGE Reference Online 2009 Encyclopedia Collection
SAGE Reference Online 2010 Encyclopedia Collection
SAGE Reference Online 2011 Encyclopedia Collection
SAGE Reference Online 2011 Green Series Bundle
SAGE Reference Online 2011 Handbook Collection
SAGE Reference Online 2012 Encyclopedia Collection
Sage Reference Online 2012 Handbook Collection
SAGE Reference Online 2013 Encyclopedia Collection
Sage Reference Online 2014 Encyclopedia Collection
Sage Reference Online 2015 Encyclopedia Collection
Sage Reference Online 2016 Encyclopedia Collection
SAGE Reference Online Backlist Encyclopedia Collection
SAGE Reference Online Community College and Law Library Bundle
Sage Reference Online Complete
SAGE Reference Online Handbook Collection 2009
SAGE Reference Online Handbook Collection 2010
SAGE Reference Online Handbook Collection 2013
Sage Reference Online Handbook Collection 2014
Sage Reference Online Handbook Collection 2015
SAGE Religion Subject Collection
SAGE Religion Subject Collection Backfile Purchase

SAGE Research Methods Cases Part I
SAGE Research Methods Cases Part II
SAGE Research Methods Cases: Medicine and Health
SAGE Research Methods Core
SAGE Research Methods Datasets Part I
SAGE Research Methods Datasets Part II
SAGE Research Methods Entire Platform
SAGE Research Methods Podcasts
SAGE Research Methods Subject Collection
SAGE Research Methods Subject Collection Backfile Purchase
SAGE Research Methods Video I
SAGE Research Methods Video: Practical Research and Academic Skills
Sage Royal Society of Medicine
SAGE Royal Society of Medicine Open Access Journals - NESLI2 2013
SAGE RSM package Subject Collection
SAGE RSM package Subject Collection Backfile Purchase
SAGE Shallow Backfile 2 2020
SAGE Shallow Backfile 2020
SAGE Shallow Backfile Package 2014
SAGE Shallow Backfile Package 2015
SAGE Shallow Backfile Package 2017
SAGE Shallow Backfile Package DFG 2014
SAGE Shallow Backfile Package with RSM 2014
SAGE Sociology Collection
SAGE Sociology FTC
SAGE Sociology Subject Collection Backfile Purchase
SAGE STM 2020
SAGE STM Backfile Upgrade Purchase 2013
SAGE STM Backfile Upgrade Purchase 2014
SAGE STM Backfile Upgrade Purchase 2015
SAGE STM Backfile Upgrade Purchase 2016
SAGE STM Backfile Upgrade Purchase 2017
SAGE STM Backfile Upgrade Purchase 2018
SAGE STM Package 2008
SAGE STM Package 2009
SAGE STM Package 2010
SAGE STM Package 2011
SAGE STM Package 2012
SAGE STM Package 2013
SAGE STM Package 2014

SAGE STM Package 2015
SAGE STM Package 2016
SAGE STM Package 2017
SAGE STM Package 2018
SAGE STM Package 2019
SAGE STM Shallow Backfile 2 2020
SAGE STM Shallow Backfile 2017
SAGE STM Without RSM
SAGE STM without RSM 2020
SAGE Symposium Journals
SAGE Urban Studies & Planning FTC
SAGE Urban Studies & Planning Subject Collection Backfile Purchase
SAGE Urban Studies And Planning Collection
Eighties in America
Fifties in America
Nineties in America
Salem Health
Salem History
Salem Science
Seventies in America
Sixties in America
Twenties in America
Schattauer ebooks
Schattauer Scientific Journals
LLMC-Digital
Scholars Portal Books: APA PsycBooks 2015-2016
Scholars Portal Books: APA PsycBooks 2016-2017
Scholars Portal Books: APA PsycBooks 2017-2018
Scholars Portal Books: APA PsycBooks 2018-2019
Scholars Portal Books: APA PsycBooks xxxx-2015
Scholars Portal Books: Business Expert Press 2010
Scholars Portal Books: Business Expert Press 2011
Scholars Portal Books: Business Expert Press 2012
Scholars Portal Books: Business Expert Press 2013
Scholars Portal Books: Business Expert Press 2014
Scholars Portal Books: Business Expert Press 2015
Scholars Portal Books: Business Expert Press 2016
Scholars Portal Books: Business Expert Press 2017
Scholars Portal Books: Business Expert Press 2018
Scholars Portal Books: Business Expert Press 2019

Scholars Portal Books: Business Expert Press 2020
Scholars Portal Books: Cambridge Books Online 2012
Scholars Portal Books: Cambridge Books Online 2013
Scholars Portal Books: Cambridge Books Online 2014
Scholars Portal Books: Cambridge Books Online 2015
Scholars Portal Books: Cambridge Books Online 2016
Scholars Portal Books: Cambridge Books Online 2017
Scholars Portal Books: Cambridge Books Online 2018
Scholars Portal Books: Cambridge Books Online xxxx-2011
Scholars Portal Books: Cambridge Companions 0000-2017
Scholars Portal Books: Cambridge Companions 2018
Scholars Portal Books: Cambridge Online 2018
Scholars Portal Books: De Gruyter - Harvard 2011
Scholars Portal Books: De Gruyter - Harvard 2012
Scholars Portal Books: De Gruyter - Harvard 2013
Scholars Portal Books: Duke 1983-2009
Scholars Portal Books: Duke 2010
Scholars Portal Books: Duke 2011
Scholars Portal Books: Duke 2012
Scholars Portal Books: Duke 2013
Scholars Portal Books: Duke 2014
Scholars Portal Books: Duke 2015
Scholars Portal Books: Duke 2016
Scholars Portal Books: Duke 2017
Scholars Portal Books: Duke 2018
Scholars Portal Books: Duke 2019
Scholars Portal Books: Edward Elgar Publishing
Scholars Portal Books: Elsevier
Scholars Portal Books: Emerald 2000-2004
Scholars Portal Books: Emerald 2005-2010
Scholars Portal Books: Emerald xxxx-1999
Scholars Portal Books: IEEE-MIT 2014
Scholars Portal Books: IEEE-MIT 2015
Scholars Portal Books: IEEE-MIT 2016
Scholars Portal Books: IEEE-MIT 2017
Scholars Portal Books: IEEE-MIT 2018
Scholars Portal Books: IEEE-Wiley 1974-2011
Scholars Portal Books: IEEE-Wiley 2012
Scholars Portal Books: IEEE-Wiley 2013
Scholars Portal Books: IEEE-Wiley 2014

Scholars Portal Books: IEEE-Wiley 2015
Scholars Portal Books: IEEE-Wiley 2016
Scholars Portal Books: IEEE-Wiley 2017
Scholars Portal Books: IEEE-Wiley 2018
Scholars Portal Books: IEEE-Wiley 2018
Scholars Portal Books: IEEE-Wiley 2019
Scholars Portal Books: IET - Scitech 2013
Scholars Portal Books: IET - Scitech 2014
Scholars Portal Books: IET - Scitech 2015
Scholars Portal Books: IET 1979-2007
Scholars Portal Books: IET 2008
Scholars Portal Books: IET 2009
Scholars Portal Books: IET 2010
Scholars Portal Books: IET 2011
Scholars Portal Books: IET 2012
Scholars Portal Books: IET 2013
Scholars Portal Books: IET 2014
Scholars Portal Books: IET 2015
Scholars Portal Books: IET 2016
Scholars Portal Books: Informa Healthcare
Scholars Portal Books: Karger Chemical Immununology and Allergy 1936-1996
Scholars Portal Books: Karger Non-Series 1997-
Scholars Portal Books: Karger Series 1997-
Scholars Portal Books: LWW
Scholars Portal Books: Morgan and Claypool Colloquium 1
Scholars Portal Books: Morgan and Claypool Colloquium 2
Scholars Portal Books: Morgan and Claypool Colloquium 3
Scholars Portal Books: Morgan and Claypool Colloquium 4
Scholars Portal Books: Morgan and Claypool Synthesis 1
Scholars Portal Books: Morgan and Claypool Synthesis 2
Scholars Portal Books: Morgan and Claypool Synthesis 3
Scholars Portal Books: Morgan and Claypool Synthesis 4
Scholars Portal Books: Morgan and Claypool Synthesis 5
Scholars Portal Books: Morgan and Claypool Synthesis 6
Scholars Portal Books: Morgan and Claypool Synthesis 7
Scholars Portal Books: OpenEdition Ottawa University
Scholars Portal Books: Palgrave 2012-2013
Scholars Portal Books: Patron Driven Acquisition 2010
Scholars Portal Books: Patron Driven Acquisition 2011
Scholars Portal Books: SPIE 1989-2010

Scholars Portal Books: SPIE 2011
Scholars Portal Books: SPIE 2012
Scholars Portal Books: SPIE 2013
Scholars Portal Books: SPIE 2014
Scholars Portal Books: SPIE 2015
Scholars Portal Books: SPIE 2016
Scholars Portal Books: SPIE 2017
Scholars Portal Books: Springer 2005-2008
Scholars Portal Books: Springer 2009
Scholars Portal Books: Springer 2010
Scholars Portal Books: Springer 2011
Scholars Portal Books: Springer 2012
Scholars Portal Books: Springer-Behavioral Science 2013
Scholars Portal Books: Springer-Behavioral Science 2014
Scholars Portal Books: Springer-Behavioral Science 2015
Scholars Portal Books: Springer-Behavioral Science and Psychology 2016
Scholars Portal Books: Springer-Behavioral Science and Psychology 2017
Scholars Portal Books: Springer-Biomedical and Life Sciences 2013
Scholars Portal Books: Springer-Biomedical and Life Sciences 2014
Scholars Portal Books: Springer-Biomedical and Life Sciences 2015
Scholars Portal Books: Springer-Biomedical and Life Sciences 2016
Scholars Portal Books: Springer-Biomedical and Life Sciences 2017
Scholars Portal Books: Springer-Business and Economics 2013
Scholars Portal Books: Springer-Business and Economics 2014
Scholars Portal Books: Springer-Business and Economics 2015
Scholars Portal Books: Springer-Business and Management 2016
Scholars Portal Books: Springer-Business and Management 2017
Scholars Portal Books: Springer-Chemistry and Material Science 2013
Scholars Portal Books: Springer-Chemistry and Material Science 2014
Scholars Portal Books: Springer-Chemistry and Material Science 2015
Scholars Portal Books: Springer-Chemistry and Material Science 2016
Scholars Portal Books: Springer-Chemistry and Material Science 2017
Scholars Portal Books: Springer-Computer Science 2013
Scholars Portal Books: Springer-Computer Science 2014
Scholars Portal Books: Springer-Computer Science 2015
Scholars Portal Books: Springer-Computer Science 2016
Scholars Portal Books: Springer-Computer Science 2017
Scholars Portal Books: Springer-Earth and Environmental Science 2013
Scholars Portal Books: Springer-Earth and Environmental Science 2014
Scholars Portal Books: Springer-Earth and Environmental Science 2015

Scholars Portal Books: Springer-Earth and Environmental Science 2016
Scholars Portal Books: Springer-Earth and Environmental Science 2017
Scholars Portal Books: Springer-Economics and Finance 2016
Scholars Portal Books: Springer-Economics and Finance 2017
Scholars Portal Books: Springer-Education 2016
Scholars Portal Books: Springer-Education 2017
Scholars Portal Books: Springer-Energy 2013
Scholars Portal Books: Springer-Energy 2014
Scholars Portal Books: Springer-Energy 2015
Scholars Portal Books: Springer-Energy 2016
Scholars Portal Books: Springer-Energy 2017
Scholars Portal Books: Springer-Engineering 2013
Scholars Portal Books: Springer-Engineering 2014
Scholars Portal Books: Springer-Engineering 2015
Scholars Portal Books: Springer-Engineering 2016
Scholars Portal Books: Springer-Engineering 2017
Scholars Portal Books: Springer-History 2016
Scholars Portal Books: Springer-History 2017
Scholars Portal Books: Springer-Humanities 2013
Scholars Portal Books: Springer-Humanities 2014
Scholars Portal Books: Springer-Humanities 2015
Scholars Portal Books: Springer-Law and Criminology 2016
Scholars Portal Books: Springer-Law and Criminology 2017
Scholars Portal Books: Springer-Math And Statistics 2013
Scholars Portal Books: Springer-Math And Statistics 2014
Scholars Portal Books: Springer-Math And Statistics 2015
Scholars Portal Books: Springer-Math And Statistics 2016
Scholars Portal Books: Springer-Math And Statistics 2017
Scholars Portal Books: Springer-Medicine 2013
Scholars Portal Books: Springer-Medicine 2014
Scholars Portal Books: Springer-Medicine 2015
Scholars Portal Books: Springer-Medicine 2016
Scholars Portal Books: Springer-Medicine 2017
Scholars Portal Books: Springer-Physics and Astronomy 2013
Scholars Portal Books: Springer-Physics and Astronomy 2014
Scholars Portal Books: Springer-Physics and Astronomy 2015
Scholars Portal Books: Springer-Physics and Astronomy 2016
Scholars Portal Books: Springer-Physics and Astronomy 2017
Scholars Portal Books: Springer-Political Science and International Studies 2016
Scholars Portal Books: Springer-Political Science and International Studies 2017

Scholars Portal Books: Springer-Professional and Applied Computing 2013
Scholars Portal Books: Springer-Professional and Applied Computing 2014
Scholars Portal Books: Springer-Professional and Applied Computing 2015
Scholars Portal Books: Springer-Professional and Applied Computing 2016
Scholars Portal Books: Springer-Professional and Applied Computing 2017
Scholars Portal Books: Springer-Protocols 1980-2009
Scholars Portal Books: Springer-Protocols 2010
Scholars Portal Books: Springer-Protocols 2011
Scholars Portal Books: Springer-Protocols 2013
Scholars Portal Books: Springer-Protocols 2014
Scholars Portal Books: Springer-Protocols 2015
Scholars Portal Books: Springer-Religion and Philosophy 2017
Scholars Portal Books: Springer-Social Sciences 2016
Scholars Portal Books: Springer-Social Sciences 2017
Scholars Portal Books: University of Chicago Press 0000-2011
Scholars Portal Books: University of Chicago Press 2012
Scholars Portal Books: University of Chicago Press 2013
Scholars Portal Books: University of Chicago Press 2014
Scholars Portal Books: University of Chicago Press 2015
Scholars Portal Books: University of Chicago Press 2016
Scholars Portal Books: University of Chicago Press 2017
Scholars Portal Books: University of Chicago Press 2018
Scholars Portal Books: Wiley 2006-2009
Scholars Portal Books: Wiley 2010
Scholars Portal Books: Wiley 2011
Scholars Portal Books: Wiley 2012
Scholars Portal Books: Wiley 2013
Scholars Portal Books: Wiley 2014
Scholars Portal Books: Wiley 2015
Scholars Portal Books: Wiley 2016
Scholars Portal Books: Wiley 2017
Scholars Portal Books: Wiley 2018
Scholars Portal Books: Wiley 2020
Scholars Portal Books: Wiley xxxx-2005
Scholars Portal Journals: Elsevier Backfile - Agricultural, and Biological Sciences
Scholars Portal Journals: Elsevier Backfile - Agricultural, and Biological Sciences Supplement 1
Scholars Portal Journals: Elsevier Backfile - Anesthesiology Pain Medicine
Scholars Portal Journals: Elsevier Backfile - Biochemistry, Genetics and Molecular Biology
Scholars Portal Journals: Elsevier Backfile - Biochemistry, Genetics and Molecular Biology Supplement 1
Scholars Portal Journals: Elsevier Backfile - Business, Management and Accounting

Scholars Portal Journals: Elsevier Backfile - Business, Management and Accounting Supplement 1
Scholars Portal Journals: Elsevier Backfile - Cell Press
Scholars Portal Journals: Elsevier Backfile - Chemical Engineering
Scholars Portal Journals: Elsevier Backfile - Chemical Engineering Supplement 1
Scholars Portal Journals: Elsevier Backfile - High Energy/Nuclear Physics and Astronomy
Scholars Portal Journals: Elsevier Backfile - Organic Chemistry
Scholars Portal Journals: Elsevier Backfile - Physical and Analytical Chemistry
Scholars Portal Journals: Elsevier Backfile: Clinical Neurology
Scholars Portal Journals: Elsevier Backfile: Computer Science
Scholars Portal Journals: Elsevier Backfile: Computer Science Supplement 1
Scholars Portal Journals: Elsevier Backfile: Decision Sciences
Scholars Portal Journals: Elsevier Backfile: Dentisty, Oral Surgery and Medicine
Scholars Portal Journals: Elsevier Backfile: Earth Planetary Sciences
Scholars Portal Journals: Elsevier Backfile: Earth Planetary Sciences Supplement 1
Scholars Portal Journals: Elsevier Backfile: Economics, Econometrics and Finance
Scholars Portal Journals: Elsevier Backfile: Economics, Econometrics and Finance Supplement 1
Scholars Portal Journals: Elsevier Backfile: Energy Power
Scholars Portal Journals: Elsevier Backfile: Engineering Technology
Scholars Portal Journals: Elsevier Backfile: Environmental Science
Scholars Portal Journals: Elsevier Backfile: Environmental Science Supplement 1
Scholars Portal Journals: Elsevier Backfile: Gastroenterology, Endocrinology and Diabetes Metabolism
Scholars Portal Journals: Elsevier Backfile: General Medicine
Scholars Portal Journals: Elsevier Backfile: Imunology and Microbiology Supplement 1
Scholars Portal Journals: Elsevier Backfile: Inorganic Chemistry
Scholars Portal Journals: Elsevier Backfile: Inorganic Chemistry Supplement 1
Scholars Portal Journals: Elsevier Backfile: Materials Science
Scholars Portal Journals: Elsevier Backfile: Mathematics
Scholars Portal Journals: Elsevier Backfile: Medicine Dentistry
Scholars Portal Journals: Elsevier Backfile: Medicine Dentistry Supplement 1
Scholars Portal Journals: Elsevier Backfile: Medicine Dentistry Supplement 2
Scholars Portal Journals: Elsevier Backfile: Neuroscience
Scholars Portal Journals: Elsevier Backfile: Neuroscience Supplement 1
Scholars Portal Journals: Elsevier Backfile: Nursing Health Professions
Scholars Portal Journals: Elsevier Backfile: Obstetrics, Gynecology and Women's Health
Scholars Portal Journals: Elsevier Backfile: Organic Chemistry Supplement 1
Scholars Portal Journals: Elsevier Backfile: Orthopedics Sports Medicine Rehabilitation
Scholars Portal Journals: Elsevier Backfile: Perinatology, Pediatrics and Child Health
Scholars Portal Journals: Elsevier Backfile: Pharmacology, Toxicology and Pharmaceutics
Scholars Portal Journals: Elsevier Backfile: Pharmacology, Toxicology and Pharmaceutics Supplement 1
Scholars Portal Journals: Elsevier Backfile: Physical and Analytical Chemistry Supplement 1

Scholars Portal Journals: Elsevier Backfile: Physics General
Scholars Portal Journals: Elsevier Backfile: Physics General Supplement 1
Scholars Portal Journals: Elsevier Backfile: Psychiatry and Mental Health
Scholars Portal Journals: Elsevier Backfile: Psychology
Scholars Portal Journals: Elsevier Backfile: Psychology Supplement 1
Scholars Portal Journals: Elsevier Backfile: Public Health Policy
Scholars Portal Journals: Elsevier Backfile: Social Science
Scholars Portal Journals: Elsevier Cell Press
Scholars Portal Journals: Elsevier Current
Scholars Portal Journals: Emerald
Scholars Portal Journals: ICE Archive
Scholars Portal Journals: IEEE
Scholars Portal Journals: Informa Health
Scholars Portal Journals: IOP
Scholars Portal Journals: IOP 2013 Archive Update
Scholars Portal Journals: Kluwer Law
Scholars Portal Journals: Maney Publishing Archive
Scholars Portal Journals: Sage
Scholars Portal Journals: SPIE
Scholars Portal Journals: Taylor & Francis Archive - Anthropology
Scholars Portal Journals: Taylor & Francis Archive - Arts and Humanities
Scholars Portal Journals: Taylor & Francis Archive - Behavioral Science
Scholars Portal Journals: Taylor & Francis Archive - Business, Management and Economics
Scholars Portal Journals: Taylor & Francis Archive - Chemistry
Scholars Portal Journals: Taylor & Francis Archive - Criminology and Law
Scholars Portal Journals: Taylor & Francis Archive - Education
Scholars Portal Journals: Taylor & Francis Archive - Engineering, Computing and Technology
Scholars Portal Journals: Taylor & Francis Archive - Environment and Agriculture
Scholars Portal Journals: Taylor & Francis Archive - Mathematics and Statistics
Scholars Portal Journals: Taylor & Francis Archive - Media, Cultural and Communication Studies
Scholars Portal Journals: Taylor & Francis Archive - Physics
Scholars Portal Journals: Taylor & Francis Archive - Politics, Internatinal Relations & Area Studies
Scholars Portal Journals: Taylor & Francis Archive - Public Health
Scholars Portal Journals: Taylor & Francis Archive - Sociology and Related Disciplines
Scholars Portal Journals: Taylor & Francis Archive - Sport, Leisure and Tourism
Scholars Portal Journals: Taylor & Francis Archive - Strategic, Defence & Security Studies
Scholars Portal Journals: Thieme
Scholars Portal University Of Chicago Press Direct
Scholastic Education PLUS
Schweizerbart Science Publications

SciELO
SciELO Argentina
SciELO Brazil
SciELO Chile
SciELO Colombia
SciELO Costa Rica
SciELO Cuba
SciELO eBooks
SciELO Ecuador
SciELO Mexico
SciELO Open Access Books
SciELO Paraguay
SciELO Peru
SciELO Portugal
SciELO Public Health
SciELO Social Sciences
SciELO Spain
SciELO Uruguay
SciELO Venezuela
Science Alert Free
Science and Engineering Research Support Society
Science and Technology Facilities Council (STFC) IR -- ePubs
Science Printers and Publishers Online Medical Journals
De Gruyter Open
TÜBİTAK Scientific Journals
Scientific American
Scientific.net Journals
SCOAP3 Journals
Sean Kingston Publishing Journals
Digital Commons @ SPU
Sejong University
SELADOC Complete Collection
SELADOC Full Text Collection
Bibliography of Linguistic Literature - Bibliographie Linguistischer Literatur
SHEDL Intellect
Community Justice Portal
全国遺跡報告総覧
Ship Index
SIAM Society for Industrial and Applied Mathematics
SIAM Society for Industrial and Applied Mathematics Books

Single Journals
Leadership Development Channel
Skillsoft Books Summaries
Slack Incorporated
Smithsonian Digital Repository
SSRN eLibrary
L'Année philologique
Société Mathematique de France
Société Rencesvals
Society for Applied Anthropology
SIAM ebook
SID (Society for Information Display) Publications
Society of American Archivists Publications
Society of Exploration Geophysicists Journals
SME Journals
Society of Petroleum Engineers
SORA Searchable Ornithological Research Archive
La Habana Elegante
Central University Libraries (CUL) Digital Collections
SMU Business Libguide
SMU Digital Repository
SMU Law Libguide
SMU LibGuides
SMU Theology Libguide
Southern Poverty Law Center (SPLC) Publications
Spandidos
SPIE Digital Library - JUSTICE
SPIE journals
KB+ BIBSAM SPIE Digital Library 2014-2016
SPIE Digital Library (Proceedings Series)
SPIE Digital Library eBooks
SPIE Digital Library Journals
SPIE Digital Library Proceedings
SPIE Open Access Publications
Spiegel Wissen
Spiral
International Historical Statistics
The New Palgrave: A Dictionary of Economics
Adis Journals & Newsletters
BioMed Central Journals Complete

BioMed Central Open Access Free
Canadian Research Knowledge Network SpringerLink Archive
Canadian Research Knowledge Network SpringerLink Current
CARLI SpringerLink Journals
CDL SpringerLink Journals 2016
CIC SpringerLink Journals
Colby, Bates and Bowdoin (CBB) SpringerLink Journals
CommunityPlus SpringerLink Journals 2016
DRAA Springer Online Journals
DRAA Springer Online Journals Biomedical and Life Sciences
DRAA Springer Online Journals Humanities and Social Sciences
DRAA Springer Online Journals Technology and Engineering
EZB-NALI2-00462 Springer Archive NL
FinELib SpringerLink Contemporary Journals
GWLA SpringerLink Journals
GWLA SpringerLink Upgrade collection
IReL SpringerLink Journals 2016
JANUL PULC SpringerLink Journals 2009
Japan Medical Library Association SpringerLink Journals 2016
JMLA SpringerLink Journals 2014
JPLA SpringerLink Journals 2017
JUSTICE SpringerLink Online Journals Archive
JUSTICE SpringerLink Online Journals Current 2018
KB+ Bibsam Springer Compact 2016-2018
KB+ BIBSAM Springer eJournals 2015-2017
KB+ JISC Collections Springer Books Professional and Applied Computing 2015 Collection
KB+ JISC Collections Springer Compact 2016-2018
KB+ JISC Collections Springer Compact 2019-2021
KB+ JISC Collections Springer eBooks Biomedical and Life Sciences 2015 Collection
KB+ JISC Collections Springer eBooks Chemistry and Materials Science 2015 Collection
KB+ JISC Collections Springer eBooks Earth and Environmental Science 2015 Collection
KB+ JISC Collections Springer eBooks Energy 2015 Collection
KB+ JISC Collections Springer eBooks Engineering 2015 Collection
KB+ JISC Collections Springer eBooks Mathematics and Statistics 2015 Collection
KB+ JISC Collections Springer eBooks Medicine 2015 Collection
KB+ JISC Collections Springer eBooks Physics and Astronomy 2015 Collection
KB+ JISC Collections Springer Institutional Agreement:2013-2015
KB+ SHEDL JISC Collections Springer Closed Consortium Agreement 2013-2015
KB+ SHEDL Springer Compact 2016-2017
KB+ SHEDL Springer Journals Adis

MCLS SpringerLink Journals
NCAL TONS SpringerLink Journals 2016
Nesli2 Springer Institutional Agreement 2014
NESLi2 SpringerLink Journals Option 1
NESLi2 SpringerLink Journals Option 2
North East Research Libraries (NERL) Springer Journals
North East Research Libraries (NERL) Springer Journals TONS 2016
North East Research Libraries (NERL) Springer TONS 2010-2014 Journals
OhioLINK SpringerLink Journals
Palgrave Connect eBooks Business & Management Collections
Palgrave Connect eBooks Economics & Finance Collections
Palgrave Connect eBooks Education Collections
Palgrave Connect eBooks History Collections
Palgrave Connect eBooks Language & Linguistics Collections
Palgrave Connect eBooks Literature & Performing Arts Collections
Palgrave Connect eBooks Literature Collections
Palgrave Connect eBooks Media & Culture Collections
Palgrave Connect eBooks Political & International Studies Collections
Palgrave Connect eBooks Religion & Philosophy Collections
Palgrave Connect eBooks Social & Cultural Studies Collections
Palgrave Connect eBooks Social Sciences Collections
Palgrave Connect eBooks Theatre & Performance Collections
SANLIC SpringerLink Journals
SHEDL SpringerLink Journals 2013
Springer Advances in Biochemical Engineering/Biotechnology eBooks
Springer Advances in Polymer Science eBooks
Springer Advances in Solid State Physics eBooks
Springer Behavioral Science and Psychology eBooks 2016 English/International
Springer Behavioral Science and Psychology eBooks 2017 English/International
Springer Behavioral Science and Psychology eBooks 2018 English/International
Springer Behavioral Science and Psychology eBooks 2019 English/International
Springer Behavioral Science and Psychology eBooks 2020 English/International
Springer Behavioral Science eBooks 2005 English/International
Springer Behavioral Science eBooks 2006 English/International
Springer Behavioral Science eBooks 2007 English/International
Springer Behavioral Science eBooks 2010 English/International
Springer Behavioral Science eBooks 2012 English/International
Springer Behavioral Science eBooks 2013 English/International
Springer Behavioral Science eBooks 2015 English/International
Springer Biomedical and Life Sciences eBooks 2005 English/International

Springer Biomedical and Life Sciences eBooks 2006 English/International
Springer Biomedical and Life Sciences eBooks 2008 English/International
Springer Biomedical and Life Sciences eBooks 2009 English/International
Springer Biomedical and Life Sciences eBooks 2010 English/International
Springer Biomedical and Life Sciences eBooks 2011 English/International
Springer Biomedical and Life Sciences eBooks 2012 English/International
Springer Biomedical and Life Sciences eBooks 2013 English/International
Springer Biomedical and Life Sciences eBooks 2015 English/International
Springer Biomedical and Life Sciences eBooks 2017 English/International
Springer Biomedical and Life Sciences eBooks 2018 English/International
Springer Biomedical and Life Sciences eBooks 2019 English/International
Springer Biomedical and Life Sciences eBooks 2020 English/International
Springer Book Archive - Alle Themen
Springer Book Archive - Alle Themen 1990-1999
Springer Book Archive - Alle Themen 2000-2004
Springer Book Archive - Alle Themen before 1990
Springer Book Archive - Behavioral Sciences
Springer Book Archive - Biomedicine & Life Sciences
Springer Book Archive - Business & Economics
Springer Book Archive - Business & Economics 1990-1999
Springer Book Archive - Business & Economics 2000-2004
Springer Book Archive - Business & Economics before 1990
Springer Book Archive - Chemistry & Material Science 1990-1999
Springer Book Archive - Chemistry & Material Science 2000-2004
Springer Book Archive - Chemistry & Material Science before 1990
Springer Book Archive - Collection
Springer Book Archive - Collection 1990-1999
Springer Book Archive - Collection 2000-2004
Springer Book Archive - Collection before 1990
Springer Book Archive - Computer Science
Springer Book Archive - Computer Science 1990-1999
Springer Book Archive - Computer Science 2000-2004
Springer Book Archive - Computer Science before 1990
Springer Book Archive - Earth & EnvironmentalScience
Springer Book Archive - Earth & EnvironmentalScience 1990-1999
Springer Book Archive - Earth & EnvironmentalScience 2000-2004
Springer Book Archive - Earth & EnvironmentalScience before 1990
Springer Book Archive - Engineering
Springer Book Archive - Engineering 1990-1999
Springer Book Archive - Engineering 2000-2004

Springer Book Archive - Engineering before 1990
Springer Book Archive - Geistes-, Sozial- und Rechtswissenschaften 1990-1999
Springer Book Archive - Geistes-, Sozial- und Rechtswissenschaften 2000-2004
Springer Book Archive - Geistes-, Sozial- und Rechtswissenschaften before 1990
Springer Book Archive - Humanities, Social Science & Law
Springer Book Archive - Humanities, Social Science & Law 1990-1999
Springer Book Archive - Humanities, Social Science & Law 2000-2004
Springer Book Archive - Humanities, Social Science & Law before 1990
Springer Book Archive - Mathematics
Springer Book Archive - Mathematics 1990-1999
Springer Book Archive - Mathematics 2000-2004
Springer Book Archive - Mathematics before 1990
Springer Book Archive - Medicine
Springer Book Archive - Medicine 1990-1999
Springer Book Archive - Medicine 2000-2004
Springer Book Archive - Medicine before 1990
Springer Book Archive - Medizin
Springer Book Archive - Medizin 1990-1999
Springer Book Archive - Medizin 2000-2004
Springer Book Archive - Medizin before 1990
Springer Book Archive - Naturwissenschaften
Springer Book Archive - Naturwissenschaften 1990-1999
Springer Book Archive - Naturwissenschaften 2000-2004
Springer Book Archive - Naturwissenschaften before 1990
Springer Book Archive - Physics and Astronomy
Springer Book Archive - Physics and Astronomy 1990-1999
Springer Book Archive - Physics and Astronomy 2000-2004
Springer Book Archive - Physics and Astronomy before 1990
Springer Book Archive - Professional Computing and Web Design
Springer Book Archive - Professional Computing and Web Design 1990-1999
Springer Book Archive - Professional Computing and Web Design 2000-2004
Springer Book Archive - Professional Computing and Web Design before 1990
Springer Book Archive - Technik & Informatik
Springer Book Archive - Technik & Informatik 1990-1999
Springer Book Archive - Technik & Informatik 2000-2004
Springer Book Archive - Technik & Informatik before 1990
Springer Book Archive - Wirtschaftswissenschaften 1990-1999
Springer Book Archive - Wirtschaftswissenschaften 2000-2004
Springer Book Archive - Wirtschaftswissenschaften before 1990
Springer Business and Economics eBooks 2009 English/International

Springer Business and Economics eBooks 2010 English/International
Springer Business and Economics eBooks 2013 English/International
Springer Business and Economics eBooks 2014 English/International
Springer Business and Management eBooks 2016 English/International
Springer Business and Management eBooks 2017 English/International
Springer Business and Management eBooks 2018 English/International
Springer Business and Management eBooks 2019 English/International
Springer Business and Management eBooks 2020 English/International
Springer Chemistry and Materials Science eBooks 2005 English/International
Springer Chemistry and Materials Science eBooks 2007 English/International
Springer Chemistry and Materials Science eBooks 2008 English/International
Springer Chemistry and Materials Science eBooks 2009 English/International
Springer Chemistry and Materials Science eBooks 2010 English/International
Springer Chemistry and Materials Science eBooks 2011 English/International
Springer Chemistry and Materials Science eBooks 2013 English/International
Springer Chemistry and Materials Science eBooks 2015 English/International
Springer Chemistry and Materials Science eBooks 2017 English/International
Springer Chemistry and Materials Science eBooks 2018 English/International
Springer Chemistry and Materials Science eBooks 2019 English/International
Springer Chemistry and Materials Science eBooks 2020 English/International
Springer Communications in Computer and Information Science
Springer Computer Science eBooks 2018 English/International
Springer Computer Science eBooks 2019 English/International
Springer Computer Science eBooks 2020 English/International
Springer Earth and Environmental Science eBooks 2006 English/International
Springer Earth and Environmental Science eBooks 2007 English/International
Springer Earth and Environmental Science eBooks 2008 English/International
Springer Earth and Environmental Science eBooks 2010 English/International
Springer Earth and Environmental Science eBooks 2013 English/International
Springer Earth and Environmental Science eBooks 2016 English/International
Springer Earth and Environmental Science eBooks 2017 English/International
Springer Earth and Environmental Science eBooks 2018 English/International
Springer Earth and Environmental Science eBooks 2019 English/International
Springer Earth and Environmental Science eBooks 2020 English/International
Springer ebooks – Licence Nationale France
Springer eBooks (EIRA)
Springer Economics and Finance eBooks 2016 English/International
Springer Economics and Finance eBooks 2017 English/International
Springer Economics and Finance eBooks 2018 English/International
Springer Economics and Finance eBooks 2019 English/International

Springer Economics and Finance eBooks 2020 English/International
Springer Education eBooks 2017 English/International
Springer Education eBooks 2018 English/International
Springer Education eBooks 2019 English/International
Springer Education eBooks 2020 English/International
Springer Energy eBooks 2013 English/International
Springer Energy eBooks 2014 English/International
Springer Energy eBooks 2015 English/International
Springer Energy eBooks 2017 English/International
Springer Energy eBooks 2018 English/International
Springer Energy eBooks 2019 English/International
Springer Energy eBooks 2020 English/International
Springer Engineering eBooks 2005 English/International
Springer Engineering eBooks 2006 English/International
Springer Engineering eBooks 2007 English/International
Springer Engineering eBooks 2010 English/International
Springer Engineering eBooks 2011 English/International
Springer Engineering eBooks 2013 English/International
Springer Engineering eBooks 2014 English/International
Springer Engineering eBooks 2016 English/International
Springer Engineering eBooks 2017 English/International
Springer Engineering eBooks 2018 English/International
Springer Engineering eBooks 2019 English/International
Springer Engineering eBooks 2020 English/International
Springer English/International eBooks 2005 - Full Set
Springer English/International eBooks 2006 - Full Set
Springer English/International eBooks 2009 - Full Set
Springer English/International eBooks 2011 - Full Set
Springer English/International eBooks 2012 - Full Set
Springer English/International eBooks 2013 - Full Set
Springer English/International eBooks 2017 - Full Set
Springer English/International eBooks 2018 - Full Set
Springer English/International eBooks 2020 - Full Set
Springer Erziehungswissenschaft und Soziale Arbeit eBooks 2020 - German Language
Springer Geistes - und Sozialwissenschaften eBooks 2006 - German Language
Springer Geistes - und Sozialwissenschaften eBooks 2010 - German Language
Springer Geistes - und Sozialwissenschaften eBooks 2012 - German Language
Springer Geistes - und Sozialwissenschaften eBooks 2013 - German Language
Springer Geisteswissenschaften eBooks 1941 - German Language
Springer Geisteswissenschaften eBooks 1948 - German Language

Springer Geisteswissenschaften eBooks 1949 - German Language
Springer Geisteswissenschaften eBooks 1950 - German Language
Springer Geisteswissenschaften eBooks 1951 - German Language
Springer Geisteswissenschaften eBooks 1952 - German Language
Springer Geisteswissenschaften eBooks 1953 - German Language
Springer Geisteswissenschaften eBooks 1954 - German Language
Springer Geisteswissenschaften eBooks 1955 - German Language
Springer Geisteswissenschaften eBooks 1956 - German Language
Springer Geisteswissenschaften eBooks 1957 - German Language
Springer Geisteswissenschaften eBooks 1958 - German Language
Springer Geisteswissenschaften eBooks 1959 - German Language
Springer Geisteswissenschaften eBooks 1960 - German Language
Springer Geisteswissenschaften eBooks 1961 - German Language
Springer Geisteswissenschaften eBooks 1962 - German Language
Springer Geisteswissenschaften eBooks 1963 - German Language
Springer Geisteswissenschaften eBooks 1964 - German Language
Springer Geisteswissenschaften eBooks 1965 - German Language
Springer Geisteswissenschaften eBooks 1966 - German Language
Springer Geisteswissenschaften eBooks 1967 - German Language
Springer Geisteswissenschaften eBooks 1968 - German Language
Springer Geisteswissenschaften eBooks 1969 - German Language
Springer Geisteswissenschaften eBooks 1970 - German Language
Springer Geisteswissenschaften eBooks 1971 - German Language
Springer Geisteswissenschaften eBooks 1972 - German Language
Springer Geisteswissenschaften eBooks 1973 - German Language
Springer Geisteswissenschaften eBooks 1974 - German Language
Springer Geisteswissenschaften eBooks 1975 - German Language
Springer Geisteswissenschaften eBooks 1976 - German Language
Springer Geisteswissenschaften eBooks 1977 - German Language
Springer Geisteswissenschaften eBooks 1978 - German Language
Springer Geisteswissenschaften eBooks 1979 - German Language
Springer Geisteswissenschaften eBooks 1980 - German Language
Springer Geisteswissenschaften eBooks 1981 - German Language
Springer Geisteswissenschaften eBooks 1982 - German Language
Springer Geisteswissenschaften eBooks 1983 - German Language
Springer Geisteswissenschaften eBooks 1984 - German Language
Springer Geisteswissenschaften eBooks 1985 - German Language
Springer Geisteswissenschaften eBooks 1986 - German Language
Springer Geisteswissenschaften eBooks 1987 - German Language
Springer Geisteswissenschaften eBooks 1988 - German Language

Springer Geisteswissenschaften eBooks 1989 - German Language
Springer Geisteswissenschaften eBooks 1990 - German Language
Springer Geisteswissenschaften eBooks 1991 - German Language
Springer Geisteswissenschaften eBooks 1992 - German Language
Springer Geisteswissenschaften eBooks 1993 - German Language
Springer Geisteswissenschaften eBooks 1994 - German Language
Springer Geisteswissenschaften eBooks 1995 - German Language
Springer Geisteswissenschaften eBooks 1996 - German Language
Springer Geisteswissenschaften eBooks 1997 - German Language
Springer Geisteswissenschaften eBooks 1998 - German Language
Springer Geisteswissenschaften eBooks 1999 - German Language
Springer Geisteswissenschaften eBooks 2000 - German Language
Springer Geisteswissenschaften eBooks 2001 - German Language
Springer Geisteswissenschaften eBooks 2002 - German Language
Springer Geisteswissenschaften eBooks 2003 - German Language
Springer Geisteswissenschaften eBooks 2004 - German Language
Springer Geisteswissenschaften eBooks 2005 - German Language
Springer Geisteswissenschaften eBooks 2006 - German Language
Springer Geisteswissenschaften eBooks 2007 - German Language
Springer Geisteswissenschaften eBooks 2008 - German Language
Springer Geisteswissenschaften eBooks 2009 - German Language
Springer Geisteswissenschaften eBooks 2010 - German Language
Springer Geisteswissenschaften eBooks 2011 - German Language
Springer Geisteswissenschaften eBooks 2012 - German Language
Springer Geisteswissenschaften eBooks 2013 - German Language
Springer Geisteswissenschaften eBooks 2014 - German Language
Springer Geisteswissenschaften eBooks 2015 - German Language
Springer Geisteswissenschaften eBooks 2016 - German Language
Springer Geisteswissenschaften eBooks 2017 - German Language
Springer Geisteswissenschaften eBooks 2018 - German Language
Springer Geisteswissenschaften eBooks 2019 - German Language
Springer Geisteswissenschaften eBooks 2020 - German Language
Springer German Language eBooks 2005 - Full Set
Springer German Language eBooks 2006 - Full Set
Springer German Language eBooks 2007 - Full Set
Springer German Language eBooks 2008 - Full Set
Springer German Language eBooks 2009 - Full Set
Springer German Language eBooks 2010 - Full Set
Springer German Language eBooks 2011 - Full Set
Springer German Language eBooks 2012 - Full Set

Springer German Language eBooks 2013 - Full Set
Springer German Language eBooks 2014 - Full Set
Springer German Language eBooks 2015 - Full Set
Springer German Language eBooks 2016 - Full Set
Springer German Language eBooks 2017 - Full Set
Springer German Language eBooks 2018 - Full Set
Springer German Language eBooks 2020 - Full Set
Springer Global Palgrave Book Archive - Political Science & International Studies Collection
Springer Handbook of Environmental Chemistry eBooks
Springer History eBooks 2016 English/International
Springer History eBooks 2017 English/International
Springer History eBooks 2018 English/International
Springer History eBooks 2019 English/International
Springer History eBooks 2020 English/International
Springer Humanities and Social Science eBook Collection 2016 English/International
Springer Humanities and Social Science eBook Collection 2017 English/International
Springer Humanities and Social Science eBook Collection 2018 English/International
Springer Humanities and Social Science eBook Collection 2020 English/International
Springer Humanities, Social Sciences and Law eBooks 2005 English/International
Springer Humanities, Social Sciences and Law eBooks 2006 English/International
Springer Humanities, Social Sciences and Law eBooks 2007 English/International
Springer Humanities, Social Sciences and Law eBooks 2008 English/International
Springer Humanities, Social Sciences and Law eBooks 2010 English/International
Springer Humanities, Social Sciences and Law eBooks 2011 English/International
Springer Humanities, Social Sciences and Law eBooks 2012 English/International
Springer Humanities, Social Sciences and Law eBooks 2013 English/International
Springer Humanities, Social Sciences and Law eBooks 2014 English/International
Springer Humanities, Social Sciences and Law eBooks 2015 English/International
Springer IFIP Advances in Information and Communication Technology
Springer Intelligent Technologies and Robotics eBooks 2019 English/International
Springer Intelligent Technologies and Robotics eBooks 2020 English/International
Springer Journals – Licence Nationale France
Springer Journals (EIRA)
Springer Law and Criminology eBooks 2017 English/International
Springer Law and Criminology eBooks 2018 English/International
Springer Law and Criminology eBooks 2019 English/International
Springer Law and Criminology eBooks 2020 English/International
Springer Lecture Notes in Business Information Processing
Springer Lecture Notes in Computer Science eBooks
Springer Lecture Notes in Control and Information Sciences eBook

Springer Lecture Notes in Earth System Sciences eBooks
Springer Lecture Notes in Mathematics eBooks
Springer Lecture Notes in Physics eBooks
Springer Lecture Notes of the Institute for Computer Sciences, Social Informatics and Telecommunications Engineering
springer link books english complete
springer link books german archive
springer link books german complete
Springer Literature, Cultural and Media Studies eBooks 2016 English/International
Springer Literature, Cultural and Media Studies eBooks 2017 English/International
Springer Literature, Cultural and Media Studies eBooks 2018 English/International
Springer Literature, Cultural and Media Studies eBooks 2020 English/International
Springer Mathematics and Statistics eBooks 2006 English/International
Springer Mathematics and Statistics eBooks 2014 English/International
Springer Mathematics and Statistics eBooks 2015 English/International
Springer Mathematics and Statistics eBooks 2016 English/International
Springer Mathematics and Statistics eBooks 2017 English/International
Springer Mathematics and Statistics eBooks 2018 English/International
Springer Mathematics and Statistics eBooks 2019 English/International
Springer Mathematics and Statistics eBooks 2020 English/International
Springer Medicine eBooks 2006 English/International
Springer Medicine eBooks 2007 English/International
Springer Medicine eBooks 2008 English/International
Springer Medicine eBooks 2009 English/International
Springer Medicine eBooks 2010 English/International
Springer Medicine eBooks 2013 English/International
Springer Medicine eBooks 2015 English/International
Springer Medicine eBooks 2018 English/International
Springer Medicine eBooks 2019 English/International
Springer Medicine eBooks 2020 English/International
Springer Medizin eBooks 2006 - German Language
Springer Medizin eBooks 2008 - German Language
Springer Medizin eBooks 2009 - German Language
Springer Medizin eBooks 2013 - German Language
Springer Medizin eBooks 2017 - German Language
Springer Natur - und Basiswissenschaften incl. Mathematik eBooks 2005 - German Language
Springer Natur - und Basiswissenschaften incl. Mathematik eBooks 2006 - German Language
Springer Natur - und Basiswissenschaften incl. Mathematik eBooks 2007 - German Language
Springer Natur - und Basiswissenschaften incl. Mathematik eBooks 2008 - German Language
Springer Natur - und Basiswissenschaften incl. Mathematik eBooks 2009 - German Language
Springer Natur - und Basiswissenschaften incl. Mathematik eBooks 2011 - German Language

Springer Natur - und Basiswissenschaften incl. Mathematik eBooks 2014 - German Language
Springer Natur - und Basiswissenschaften incl. Mathematik eBooks 2015 - German Language
Springer Natur - und Basiswissenschaften incl. Mathematik eBooks 2017 - German Language
Springer Natur - und Basiswissenschaften incl. Mathematik eBooks 2020 - German Language
Springer Nature Book Archives Millennium (2000-2004)
Springer Nature German Automotive
Springer Nature LALC Springer Ebooks & Books Series Consortium Collection
Springer Nature LALC Springer Protocols 2016-2018 Consortium Collection
Springer Nature OA Free Journals
Springer Nature Optimum LYRASIS 2017 Collection
Springer Nature Spanish National Consortium 2018 Collection
Springer Palgrave Macmillan Journals
Springer Physics and Astronomy eBooks 2005 English/International
Springer Physics and Astronomy eBooks 2008 English/International
Springer Physics and Astronomy eBooks 2009 English/International
Springer Physics and Astronomy eBooks 2011 English/International
Springer Physics and Astronomy eBooks 2012 English/International
Springer Physics and Astronomy eBooks 2013 English/International
Springer Physics and Astronomy eBooks 2014 English/International
Springer Physics and Astronomy eBooks 2015 English/International
Springer Physics and Astronomy eBooks 2016 English/International
Springer Physics and Astronomy eBooks 2017 English/International
Springer Physics and Astronomy eBooks 2018 English/International
Springer Physics and Astronomy eBooks 2019 English/International
Springer Physics and Astronomy eBooks 2020 English/International
Springer Political Science and International Studies eBooks 2017 English/International
Springer Political Science and International Studies eBooks 2018 English/International
Springer Political Science and International Studies eBooks 2019 English/International
Springer Political Science and International Studies eBooks 2020 English/International
Springer Professional and Applied Computing eBooks 2006 English/International
Springer Professional and Applied Computing eBooks 2007 English/International
Springer Professional and Applied Computing eBooks 2008 English/International
Springer Professional and Applied Computing eBooks 2009 English/International
Springer Professional and Applied Computing eBooks 2010 English/International
Springer Professional and Applied Computing eBooks 2013 English/International
Springer Professional and Applied Computing eBooks 2015 English/International
Springer Professional and Applied Computing eBooks 2017 English/International
Springer Professional and Applied Computing eBooks 2018 English/International
Springer Professional and Applied Computing eBooks 2019 English/International
Springer Professional and Applied Computing eBooks 2020 English/International

Springer Progress in Colloid and Polymer Science eBooks
Springer Psychologie eBooks 2015 - German Language
Springer Psychologie eBooks 2016 - German Language
Springer Psychologie eBooks 2017 - German Language
Springer Psychologie eBooks 2018 - German Language
Springer Psychologie eBooks 2020 - German Language
Springer Religion and Philosophy eBooks 2017 English/International
Springer Religion and Philosophy eBooks 2018 English/International
Springer Religion and Philosophy eBooks 2019 English/International
Springer Religion and Philosophy eBooks 2020 English/International
Springer Russian Library of Science
Springer Series in Optical Sciences eBooks
Springer Social Sciences eBooks 2017 English/International
Springer Social Sciences eBooks 2018 English/International
Springer Social Sciences eBooks 2019 English/International
Springer Social Sciences eBooks 2020 English/International
Springer Sozialwissenschaften und Recht eBooks 2016 - German Language
Springer Sozialwissenschaften und Recht eBooks 2017 - German Language
Springer Sozialwissenschaften und Recht eBooks 2018 - German Language
Springer STM eBook Collection 2016 English/International
Springer STM eBook Collection 2017 English/International
Springer STM eBook Collection 2018 English/International
Springer STM eBook Collection 2020 English/International
Springer Structure and Bonding eBooks
Springer Studies in Computational Intelligence eBooks
Springer Studies in Fuzziness and Soft Computing eBooks
Springer Technik & Informatik eBooks 2005 - German Language
Springer Technik & Informatik eBooks 2007 - German Language
Springer Technik & Informatik eBooks 2008 - German Language
Springer Technik & Informatik eBooks 2009 - German Language
Springer Technik & Informatik eBooks 2010 - German Language
Springer Technik & Informatik eBooks 2011 - German Language
Springer Technik & Informatik eBooks 2012 - German Language
Springer Technik & Informatik eBooks 2013 - German Language
Springer Technik & Informatik eBooks 2015 - German Language
Springer Technik & Informatik eBooks 2019 - German Language
Springer Technik & Informatik eBooks 2020 - German Language
Springer Topics in Current Chemistry eBooks
Springer Topics in Heterocyclic Chemistry eBooks
Springer Topics in Organometallic Chemistry eBooks

Springer Tracts in Advanced Robotics eBooks
Springer Tracts in Modern Physics eBooks
Springer Understanding Complex Systems eBooks
Springer Wirtschaftswissenschaften eBooks 2007 - German Language
Springer Wirtschaftswissenschaften eBooks 2008 - German Language
Springer Wirtschaftswissenschaften eBooks 2010 - German Language
Springer Wirtschaftswissenschaften eBooks 2013 - German Language
Springer Wirtschaftswissenschaften eBooks 2015 - German Language
Springer Wirtschaftswissenschaften eBooks 2018 - German Language
Springer Wirtschaftswissenschaften eBooks 2020 - German Language
SpringerLINK - UNIPA 2016
SpringerLINK Archive - CEIRC
SpringerLink Books Architecture And Design 2005
SpringerLink Books Architecture And Design 2006
SpringerLink Books Architecture And Design 2007
SpringerLink Books Architecture And Design 2008
SpringerLink Books Architecture Design
SpringerLink Books Business And Economics 2017
SpringerLink Books Business and Economics German
SpringerLink Books Computer Science and Engineering German
SpringerLink Books Computer Science and Engineering German Archive
SpringerLink Books Computer Science without Lecture Notes 2005
SpringerLink Books Computer Science without Lecture Notes 2006
SpringerLink Books Computer Science without Lecture Notes 2010
SpringerLink Books Computer Science without Lecture Notes 2011
SpringerLink Books Computer Science without Lecture Notes 2013
SpringerLink Books Computer Science without Lecture Notes 2014
SpringerLink Books Computer Science Without Lecture Notes 2015
SpringerLink Books Computer Science Without Lecture Notes 2016
SpringerLink Books Computer Science Without Lecture Notes 2017
SpringerLink Books Energy
SpringerLink Books Engineering
SpringerLink Books Humanities Social Sciences and Law Archive
SpringerLink Books Humanities, Social Science German
SpringerLink Books J.B. Metzler Humanities German
SpringerLink Books Lecture Notes In Computer Science 2015
SpringerLink Books Lecture Notes In Computer Science 2016
SpringerLink Books Lecture Notes In Computer Science 2017
SpringerLink Books Lecture Notes In Computer Science 2018
SpringerLink Books Lecture Notes In Computer Science Archive

SpringerLink Books Lecture Notes In Mathematics 2015
SpringerLink Books Lecture Notes In Mathematics 2016
SpringerLink Books Lecture Notes In Physics 2015
SpringerLink Books Lecture Notes In Physics 2017
SpringerLink Books Lecture Notes In Physics 2018
SpringerLink Books Life Science & Basic Disciplines German
SpringerLink Books Psychology German
SpringerLink Chinese Library of Science
SpringerLINK ebooks - Current Topics in Microbiology and Immunology (Contemporary)
SpringerLINK ebooks - J.B. Metzler Humanities 2020 German Language
SpringerLink ebooks - Mathematics and Statistics (2013)
SpringerLink FLVC Journals Collection
SpringerLink Health & Hospitals Cancer Journals 2016
SpringerLink Health & Hospitals General Hospitals Journals 2016
SpringerLink Health & Hospitals Paediatrics Journals 2016
SpringerLink Historical Archives Behavioral Sciences
SpringerLink Historical Archives Biomedical and Life Sciences
SpringerLink Historical Archives Business and Economics
SpringerLink Historical Archives Chemistry and Materials Science
SpringerLink Historical Archives Engineering
SpringerLink Historical Archives Mathematics
SpringerLink Historical Archives Medicine
SpringerLink Historical Archives Physics and Astronomy
SpringerLink Lecture Notes in Computer Science 2005
SpringerLink Lecture Notes in Computer Science 2006
SpringerLink Lecture Notes in Computer Science 2007
SpringerLink Lecture Notes in Computer Science 2008
SpringerLink Lecture Notes in Computer Science 2009
SpringerLink Lecture Notes in Computer Science 2010
SpringerLink Lecture Notes in Computer Science 2011
SpringerLink Lecture Notes in Computer Science 2012
SpringerLink Lecture Notes in Computer Science 2013
SpringerLink Lecture Notes In Computer Science 2014
SpringerLINK Lecture Notes in Computer Science 2020
SpringerLink Lecture Notes in Mathematics 2005
SpringerLink Lecture Notes in Mathematics 2006
SpringerLink Lecture Notes in Mathematics 2007
SpringerLink Lecture Notes in Mathematics 2008
SpringerLink Lecture Notes in Mathematics 2009
SpringerLink Lecture Notes in Mathematics 2010

SpringerLink Lecture Notes in Mathematics 2011
SpringerLink Lecture Notes in Mathematics 2012
SpringerLink Lecture Notes in Mathematics 2013
SpringerLink Lecture Notes In Mathematics 2014
SpringerLink Lecture Notes in Mathematics 2017
SpringerLink Lecture Notes in Mathematics 2018
SpringerLINK Lecture Notes in Mathematics 2020
SpringerLink Lecture Notes in Physics 2005
SpringerLink Lecture Notes in Physics 2006
SpringerLink Lecture Notes in Physics 2007
SpringerLink Lecture Notes in Physics 2008
SpringerLink Lecture Notes in Physics 2009
SpringerLink Lecture Notes in Physics 2010
SpringerLink Lecture Notes in Physics 2011
SpringerLink Lecture Notes in Physics 2012
SpringerLink Lecture Notes in Physics 2013
SpringerLink Lecture Notes In Physics 2014
SpringerLINK Lecture Notes in Physics 2020
SpringerLink Online Journals Archive Complete
SpringerLink Open Access eBooks
SpringerLINK Optimum LYRASIS 2017 Journal collection
SpringerLink Protocols 2014
SpringerLink Series
SpringerNature CAUL
SpringerNature Complete eBooks
SpringerNature Complete eBooks Archive pre-2005
SpringerNature Complete eBooks Contemporary 2005+
SpringerNature Complete Journals
SpringerNature Complete Protocols
SpringerNature French eBook collection
SpringerNature French eBook collection Medical Sciences
SpringerNature French eBook collection Natural Sciences
SpringerNature Full Italian eBook collection
SpringerNature Hospitals and Health - Biomedicine - Books - Archive
SpringerNature Hospitals and Health - Biomedicine - Books - Contemporary
SpringerNature Hospitals and Health - Internal Medicine, Dermatology - Books - Archive
SpringerNature Hospitals and Health - Internal Medicine, Dermatology - Books - Contemporary
SpringerNature Hospitals and Health - Nephrology - Books - Archive
SpringerNature Hospitals and Health - Nephrology - Books - Contemporary
SpringerNature Hospitals and Health - Neurology - Books - Archive

SpringerNature Hospitals and Health - Neurology - Books - Contemporary
SpringerNature Hospitals and Health - Nuclear Medicine - Books - Archive
SpringerNature Hospitals and Health - Nuclear Medicine - Books - Contemporary
SpringerNature Hospitals and Health - Oncology & Hematology - Books - Archive
SpringerNature Hospitals and Health - Oncology & Hematology - Books - Contemporary
SpringerNature Hospitals and Health - Orthopedics - Books - Archive
SpringerNature Hospitals and Health - Orthopedics - Books - Contemporary
SpringerNature Hospitals and Health - Pathology - Books - Archive
SpringerNature Hospitals and Health - Pathology - Books - Contemporary
SpringerNature Hospitals and Health - Pharmacology & Toxicology - Books - Archive
SpringerNature Hospitals and Health - Pharmacology & Toxicology - Books - Contemporary
SpringerNature Hospitals and Health - Public Health - Books - Archive
SpringerNature Hospitals and Health - Public Health - Books - Contemporary
SpringerNature Hospitals and Health - Radiology - Books - Archive
SpringerNature Hospitals and Health - Radiology - Books - Contemporary
SpringerNature Hospitals and Health - Surgery & Anesthesiology - Books - Archive
SpringerNature Hospitals and Health - Surgery & Anesthesiology - Books - Contemporary
SpringerNature Hospitals and Health - Urology & Gynecology - Books - Archive
SpringerNature Hospitals and Health - Urology & Gynecology - Books - Contemporary
SpringerNature Industry Sector - Aerospace - Archive
SpringerNature Industry Sector - Aerospace - Contemporary
SpringerNature Industry Sector - Automotive - Archive
SpringerNature Industry Sector - Automotive - Contemporary
SpringerNature Industry Sector - Biotechnology - Archive
SpringerNature Industry Sector - Biotechnology - Contemporary
SpringerNature Industry Sector - Chemical Manufacturing - Archive
SpringerNature Industry Sector - Chemical Manufacturing - Contemporary
SpringerNature Industry Sector - Consumer Packaged Goods - Archive
SpringerNature Industry Sector - Consumer Packaged Goods - Contemporary
SpringerNature Industry Sector - Electronics - Archive
SpringerNature Industry Sector - Electronics - Contemporary
SpringerNature Industry Sector - Energy, Utilities & Environment - Archive
SpringerNature Industry Sector - Energy, Utilities & Environment - Contemporary
SpringerNature Industry Sector - Engineering - Archive
SpringerNature Industry Sector - Engineering - Contemporary
SpringerNature Industry Sector - Finance, Business & Banking - Archive
SpringerNature Industry Sector - Finance, Business & Banking - Contemporary
SpringerNature Industry Sector - Health & Hospitals - Archive
SpringerNature Industry Sector - Health & Hospitals - Contemporary
SpringerNature Industry Sector - IT & Software - Archive

SpringerNature Industry Sector - IT & Software - Contemporary
SpringerNature Industry Sector - Law - Archive
SpringerNature Industry Sector - Law - Contemporary
SpringerNature Industry Sector - Materials & Steel - Archive
SpringerNature Industry Sector - Materials & Steel - Contemporary
SpringerNature Industry Sector - Oil & Gas - Archive
SpringerNature Industry Sector - Oil & Gas - Contemporary
SpringerNature Industry Sector - Pharma - Archive
SpringerNature Industry Sector - Pharma - Contemporary
SpringerNature Industry Sector - Telecommunications - Archive
SpringerNature Industry Sector - Telecommunications - Contemporary
SpringerNature Italian eBook collection Humanities and Social Sciences
SpringerNature Italian eBook collection Mathematics, Statistics and Natural Sciences
SpringerNature Italian eBook collection Medical and Life Sciences
SpringerNature J.B. Metzler Humanities eBooks 2005
SpringerNature J.B. Metzler Humanities eBooks 2006
SpringerNature J.B. Metzler Humanities eBooks 2007
SpringerNature J.B. Metzler Humanities eBooks 2008
SpringerNature J.B. Metzler Humanities eBooks 2009
SpringerNature J.B. Metzler Humanities eBooks 2010
SpringerNature J.B. Metzler Humanities eBooks 2011
SpringerNature J.B. Metzler Humanities eBooks 2012
SpringerNature J.B. Metzler Humanities eBooks 2013
SpringerNature J.B. Metzler Humanities eBooks 2014
SpringerNature J.B. Metzler Humanities eBooks 2015
SpringerNature J.B. Metzler Humanities eBooks 2016
SpringerNature J.B. Metzler Humanities eBooks 2017
SpringerNature J.B. Metzler Humanities eBooks 2018
SpringerNature J.B. Metzler Humanities eBooks 2019
SpringerNature J.B. Metzler Humanities eBooks 2020
SpringerNature J.B. Metzler Humanities eBooks Contemporary (2005+)
SpringerNature LYRASIS 2018 Consortium Collection
SpringerNature Palgrave Business & Management Collection 2005
SpringerNature Palgrave Business & Management Collection 2006
SpringerNature Palgrave Business & Management Collection 2007
SpringerNature Palgrave Business & Management Collection 2008
SpringerNature Palgrave Business & Management Collection 2009
SpringerNature Palgrave Business & Management Collection 2010
SpringerNature Palgrave Business & Management Collection 2011
SpringerNature Palgrave Business & Management Collection 2012

SpringerNature Palgrave Business & Management Collection 2013
SpringerNature Palgrave Business & Management Collection 2014
SpringerNature Palgrave Business & Management Collection 2015
SpringerNature Palgrave Economics & Finance Collection 2005
SpringerNature Palgrave Economics & Finance Collection 2006
SpringerNature Palgrave Economics & Finance Collection 2007
SpringerNature Palgrave Economics & Finance Collection 2008
SpringerNature Palgrave Economics & Finance Collection 2009
SpringerNature Palgrave Economics & Finance Collection 2010
SpringerNature Palgrave Economics & Finance Collection 2011
SpringerNature Palgrave Economics & Finance Collection 2012
SpringerNature Palgrave Economics & Finance Collection 2013
SpringerNature Palgrave Economics & Finance Collection 2014
SpringerNature Palgrave Economics & Finance Collection 2015
SpringerNature Palgrave Education Collection 2007
SpringerNature Palgrave Education Collection 2008
SpringerNature Palgrave Education Collection 2009
SpringerNature Palgrave Education Collection 2010
SpringerNature Palgrave Education Collection 2011
SpringerNature Palgrave Education Collection 2012
SpringerNature Palgrave Education Collection 2013
SpringerNature Palgrave Education Collection 2014
SpringerNature Palgrave Education Collection 2015
SpringerNature Palgrave History Collection 2005
SpringerNature Palgrave History Collection 2006
SpringerNature Palgrave History Collection 2007
SpringerNature Palgrave History Collection 2008
SpringerNature Palgrave History Collection 2009
SpringerNature Palgrave History Collection 2010
SpringerNature Palgrave History Collection 2011
SpringerNature Palgrave History Collection 2012
SpringerNature Palgrave History Collection 2013
SpringerNature Palgrave History Collection 2014
SpringerNature Palgrave History Collection 2015
SpringerNature Palgrave Intern. Relations & Development Collection 2010
SpringerNature Palgrave Intern. Relations & Development Collection 2011
SpringerNature Palgrave Intern. Relations & Development Collection 2012
SpringerNature Palgrave Intern. Relations & Development Collection 2013
SpringerNature Palgrave Language & Linguistics Collection 2005
SpringerNature Palgrave Language & Linguistics Collection 2006

SpringerNature Palgrave Language & Linguistics Collection 2007
SpringerNature Palgrave Language & Linguistics Collection 2008
SpringerNature Palgrave Language & Linguistics Collection 2009
SpringerNature Palgrave Language & Linguistics Collection 2010
SpringerNature Palgrave Language & Linguistics Collection 2011
SpringerNature Palgrave Language & Linguistics Collection 2012
SpringerNature Palgrave Language & Linguistics Collection 2013
SpringerNature Palgrave Language & Linguistics Collection 2014
SpringerNature Palgrave Language & Linguistics Collection 2015
SpringerNature Palgrave Literature & Performing Arts Collection 2005
SpringerNature Palgrave Literature & Performing Arts Collection 2006
SpringerNature Palgrave Literature & Performing Arts Collection 2007
SpringerNature Palgrave Literature & Performing Arts Collection 2008
SpringerNature Palgrave Literature & Performing Arts Collection 2009
SpringerNature Palgrave Literature & Performing Arts Collection 2010
SpringerNature Palgrave Literature & Performing Arts Collection 2012
SpringerNature Palgrave Literature & Performing Arts Collection 2013
SpringerNature Palgrave Literature & Performing Arts Collection 2014
SpringerNature Palgrave Literature Collection 2005
SpringerNature Palgrave Literature Collection 2006
SpringerNature Palgrave Literature Collection 2007
SpringerNature Palgrave Literature Collection 2008
SpringerNature Palgrave Literature Collection 2009
SpringerNature Palgrave Literature Collection 2010
SpringerNature Palgrave Literature Collection 2011
SpringerNature Palgrave Literature Collection 2012
SpringerNature Palgrave Literature Collection 2013
SpringerNature Palgrave Literature Collection 2014
SpringerNature Palgrave Literature Collection 2015
SpringerNature Palgrave Media & Culture Collection 2005
SpringerNature Palgrave Media & Culture Collection 2006
SpringerNature Palgrave Media & Culture Collection 2007
SpringerNature Palgrave Media & Culture Collection 2008
SpringerNature Palgrave Media & Culture Collection 2009
SpringerNature Palgrave Media & Culture Collection 2010
SpringerNature Palgrave Media & Culture Collection 2011
SpringerNature Palgrave Media & Culture Collection 2012
SpringerNature Palgrave Media & Culture Collection 2013
SpringerNature Palgrave Media & Culture Collection 2014
SpringerNature Palgrave Media & Culture Collection 2015

SpringerNature Palgrave Political & Intern. Studies Collection 2005
SpringerNature Palgrave Political & Intern. Studies Collection 2006
SpringerNature Palgrave Political & Intern. Studies Collection 2007
SpringerNature Palgrave Political & Intern. Studies Collection 2008
SpringerNature Palgrave Political & Intern. Studies Collection 2009
SpringerNature Palgrave Political & Intern. Studies Collection 2010
SpringerNature Palgrave Political & Intern. Studies Collection 2011
SpringerNature Palgrave Political & Intern. Studies Collection 2012
SpringerNature Palgrave Political & Intern. Studies Collection 2013
SpringerNature Palgrave Political & Intern. Studies Collection 2014
SpringerNature Palgrave Political & Intern. Studies Collection 2015
SpringerNature Palgrave Political Science Collection 2008
SpringerNature Palgrave Political Science Collection 2010
SpringerNature Palgrave Political Science Collection 2011
SpringerNature Palgrave Political Science Collection 2012
SpringerNature Palgrave Religion & Philosophy Collection 2005
SpringerNature Palgrave Religion & Philosophy Collection 2006
SpringerNature Palgrave Religion & Philosophy Collection 2007
SpringerNature Palgrave Religion & Philosophy Collection 2008
SpringerNature Palgrave Religion & Philosophy Collection 2009
SpringerNature Palgrave Religion & Philosophy Collection 2010
SpringerNature Palgrave Religion & Philosophy Collection 2011
SpringerNature Palgrave Religion & Philosophy Collection 2012
SpringerNature Palgrave Religion & Philosophy Collection 2013
SpringerNature Palgrave Religion & Philosophy Collection 2014
SpringerNature Palgrave Religion & Philosophy Collection 2015
SpringerNature Palgrave Social & Cultural Studies Collection 2005
SpringerNature Palgrave Social & Cultural Studies Collection 2006
SpringerNature Palgrave Social & Cultural Studies Collection 2007
SpringerNature Palgrave Social & Cultural Studies Collection 2008
SpringerNature Palgrave Social & Cultural Studies Collection 2009
SpringerNature Palgrave Social & Cultural Studies Collection 2011
SpringerNature Palgrave Social & Cultural Studies Collection 2012
SpringerNature Palgrave Social & Cultural Studies Collection 2014
SpringerNature Palgrave Social & Cultural Studies Collection 2015
SpringerNature Palgrave Social Sciences Collection 2005
SpringerNature Palgrave Social Sciences Collection 2006
SpringerNature Palgrave Social Sciences Collection 2007
SpringerNature Palgrave Social Sciences Collection 2008
SpringerNature Palgrave Social Sciences Collection 2009

SpringerNature Palgrave Social Sciences Collection 2010
SpringerNature Palgrave Social Sciences Collection 2011
SpringerNature Palgrave Social Sciences Collection 2012
SpringerNature Palgrave Social Sciences Collection 2013
SpringerNature Palgrave Social Sciences Collection 2014
SpringerNature Palgrave Social Sciences Collection 2015
SpringerNature Palgrave Theatre & Performance Collection 2009
SpringerNature Palgrave Theatre & Performance Collection 2010
SpringerNature Palgrave Theatre & Performance Collection 2011
SpringerNature Palgrave Theatre & Performance Collection 2012
SpringerNature Palgrave Theatre & Performance Collection 2013
SpringerNature Palgrave Theatre & Performance Collection 2014
SpringerNature Palgrave Theatre & Performance Collection 2015
SpringerNature Tsus Journals 2018
Springer在线回溯数据库
VHLC SpringerLink Journals 2016
Virginia Group SpringerLink Journals
WiLS Springer Journal Package
Springer Publishing Connect
SpringerLink Books - AutoHoldings
SpringerLink Journals - AutoHoldings
Sri Lanka Journals Online
SSOAR (Social Science Open Access Repository)
St. Jerome Journals
E-Dissertationen der Universität Hamburg
eDoc.ViFaPol
Conservation OnLine
Digital Collections @ Stanford (SULAIR)
STAT!Ref
PALMM Textual Collections
Math-Net.Ru (free access)
SIPRI Yearbook
Electronic Journal System of STPI
Open SUNY Textbooks
SUNY Geneseo KnightScholar
Surry County Digital Heritage
Susquehanna University CONTENTdm
SwePub
SwePub Articles
SwePub Artistic

SWEPUB Blekinge Tekniska Högskola
SwePub Book
SwePub Book Chapter
SwePub Conference
SwePub Editorial
SWEPUB Ersta Sköndal högskola
SWEPUB Försvarshögskolan
SWEPUB Freely available online
SWEPUB Gymnastik- och idrottshögskolan
SWEPUB Högskolan Dalarna
SWEPUB Högskolan i Borås
SWEPUB Högskolan i Gävle
SWEPUB Högskolan i Halmstad
SWEPUB Högskolan i Jönköping
SWEPUB Högskolan i Skövde
SWEPUB Högskolan Kristianstad
SWEPUB Högskolan Väst
SWEPUB Karlstads universitet
SWEPUB Kungliga Tekniska Högskolan
SWEPUB Linköpings universitet
SWEPUB Linnéuniversitetet
SWEPUB Lunds universitet
SWEPUB Mälardalens högskola
SWEPUB Malmö högskola
SWEPUB Mittuniversitetet
SWEPUB Nordiska Afrikainstitutet
SWEPUB Örebro universitet
SwePub Other
SwePub Patent
SwePub Reports
SwePub Review
SWEPUB Södertörns högskola- SwePub
SWEPUB Sophiahemmet Högskola
SWEPUB Stockholms universitet
SwePub Thesis
SWEPUB Umeå universitet
SWEPUB Uppsala universitet
Cambridge University Press Journals (Swiss National Licence)
De Gruyter Online Journals (Swiss National Licence)
Oxford University Press Journals (Swiss National Licence)

Springer Journals (Swiss National Licence)
TAEBDC ABC-Clio eBooks 2010
TAEBDC ABC-Clio eBooks 2011
TAEBDC ABC-Clio eBooks 2012
TAEBDC Abc-Clio eBooks 2014
TAEBDC Books@Ovid 2009
TAEBDC Books@Ovid 2010
TAEBDC Books@Ovid 2011
TAEBDC Books@Ovid 2012
TAEBDC Books@Ovid 2013
TAEBDC Books@Ovid 2015
TAEBDC Cambridge Books Online (CBO) 2008
TAEBDC Cambridge Books Online (CBO) 2009
TAEBDC Cambridge Books Online (CBO) 2010
TAEBDC Cambridge Books Online (CBO) 2011
TAEBDC Columbia University Press eBooks 2009
TAEBDC Columbia University Press eBooks 2014
TAEBDC CRC netBase eBooks 2014
TAEBDC De Gruyter eBooks 2014
TAEBDC EBL 2015
TAEBDC EBL 2016
TAEBDC Ebrary eBooks 2009
TAEBDC Ebrary eBooks 2010
TAEBDC Ebrary eBooks 2011
TAEBDC Ebrary eBooks 2012
TAEBDC Elsevier ScienceDirect Books 2013
TAEBDC Elsevier ScienceDirect Books 2014
TAEBDC Elsevier ScienceDirect Books 2016
TAEBDC Elsevier ScienceDirect eBooks 2009
TAEBDC Elsevier ScienceDirect eBooks 2010
TAEBDC Elsevier ScienceDirect eBooks 2011
TAEBDC Elsevier ScienceDirect eBooks 2012
TAEBDC Emerald eBooks 2011
TAEBDC Emerald eBooks 2014
TAEBDC IGI Global InfoSci Books 2011
TAEBDC IGI Global InfoSci Books 2012
TAEBDC IGI Global InfoSci Books 2013
TAEBDC IGI Global InfoSci EBooks 2014
TAEBDC Informa Healthcare eBooks 2011
TAEBDC Informa Healthcare eBooks 2012

TAEBDC IOS eBooks 2008
TAEBDC IOS eBooks 2009
TAEBDC IOS eBooks 2010
TAEBDC IOS eBooks 2011
TAEBDC IOS eBooks 2012
TAEBDC IOS eBooks 2013
TAEBDC IOS eBooks 2014
TAEBDC IOS eBooks 2015
TAEBDC IOS eBooks 2016
TAEBDC Jstor Books 2014
TAEBDC Jstor Books 2015
TAEBDC Karger eBooks 2011
TAEBDC Karger eBooks 2012
TAEBDC Karger eBooks 2013
TAEBDC Library & eBooks 2011
TAEBDC McGraw-Hill eBooks 2010
TAEBDC McGraw-Hill eBooks 2012
TAEBDC MyiLibrary eBooks 2008
TAEBDC MyiLibrary eBooks 2009
TAEBDC NetLibrary eBooks 2008
TAEBDC NetLibrary eBooks 2009
TAEBDC Oxford Scholarship Online (OSO) eBooks 2008
TAEBDC Oxford Scholarship Online (OSO) eBooks 2009
TAEBDC Oxford Scholarship Online (OSO) eBooks 2010
TAEBDC Oxford Scholarship Online (OSO) eBooks 2012
TAEBDC Oxford Scholarship Online eBooks 2013
TAEBDC Oxford Scholarship Online eBooks 2014
TAEBDC Project Muse eBooks 2013
TAEBDC Sage eBooks 2014
TAEBDC Sage eBooks 2015
TAEBDC Springer eBooks 2008
TAEBDC Springer eBooks 2009
TAEBDC Springer eBooks 2010
TAEBDC Springer eBooks 2011
TAEBDC Springer eBooks 2012
TAEBDC Springer eBooks 2014
TAEBDC Springer eBooks 2015
TAEBDC Springer eBooks 2016
TAEBDC Taylor & Francis eBooks 2010
TAEBDC Taylor & Francis eBooks 2011

TAEBDC Taylor & Francis eBooks 2014
TAEBDC Taylor & Francis eBooks 2015
TAEBDC Wiley eBooks 2013
TAEBDC Wiley eBooks 2016
TAEBDC Woodhead eBooks 2013
TAEBDC Woodhead eBooks 2014
TAEBDC World Scientific eBooks 2012
TAEBDC World Scientific eBooks 2013
TAEBDC World Scientific eBooks 2014
Taipei Veterans General Hospital Journals
Medieval and Early Modern Sources Online (MEMSO)
Tax Notes
Routledge Handbooks Online Archaeology & Classics 2018
Routledge Handbooks Online Asian Studies 2018
Routledge Handbooks Online Business & Economics 2018
Routledge Handbooks Online Communication, Journalism, Media and Culture 2018
Routledge Handbooks Online Complete
Routledge Handbooks Online Criminal Justice & Criminology 2018
Routledge Handbooks Online Education 2018
Routledge Handbooks Online Environment & Sustainability 2018
Routledge Handbooks Online Foreign Languages 2018
Routledge Handbooks Online Geography 2018
Routledge Handbooks Online History 2018
Routledge Handbooks Online Law 2018
Routledge Handbooks Online Linguistics 2018
Routledge Handbooks Online Literature 2018
Routledge Handbooks Online Politics 2018
Routledge Handbooks Online Psychology 2018
Routledge Handbooks Online Religion 2018
Routledge Handbooks Online Security Studies 2018
Routledge Handbooks Online Sociology 2018
Routledge Handbooks Online Sport & Leisure 2018
Taiwan Academic E-Books Consortium (TAEBC) Taylor & Francis eBooks
Taylor & Francis Business & Management eBooks
Taylor & Francis eBooks Archeology
Taylor & Francis eBooks Asian Studies
Taylor & Francis eBooks Complete
Taylor & Francis eBooks English Language and Linguistics
Taylor & Francis eBooks Health Studies
Taylor & Francis eBooks Law

Taylor & Francis eBooks Media and Communication
Taylor & Francis eBooks Middle East
Taylor & Francis eBooks Psychology
Taylor & Francis eBooks Security
Taylor & Francis eBooks Social Work
Taylor & Francis eBooks Sport
Taylor & Francis Economics eBooks
Taylor & Francis Education eBooks
Taylor & Francis Geography eBooks
Taylor & Francis History eBooks
Taylor & Francis Literature eBooks
Taylor & Francis Philosophy eBooks
Taylor & Francis Politics & International Relations eBooks
Taylor & Francis Sociology eBooks
VIVA Taylor & Francis Evidence-Based Ebooks
Bacon Taylor&Francis Couperin Ssh-Backfiles-And-Frontlist
Bacon Taylor&Francis Couperin St-Backfiles-And-Frontlist
Bloomsbury Taylor & Francis Visual Collection 2015
CAUL Taylor & Francis Ceased Titles Package 2019
CAUL Taylor & Francis Journals
Cogent OA
IReL-KB Taylor and Francis
KB+ BIBSAM Taylor & Francis Informa Healthcare Journals 2014-2016
KB+ BIBSAM Taylor & Francis Informa Healthcare Journals 2017-2019
KB+ Bibsam Taylor & Francis Medical Library 2015 Collection 2018-2020
KB+ Bibsam Taylor & Francis Medical Library 2018 Collection 2018-2020
KB+ Bibsam Taylor & Francis Ssh + S&T Library 2015 Collection 2018-2020
KB+ Bibsam Taylor & Francis Ssh + S&T Library 2018 Collection 2018-2020
KB+ BIBSAM Taylor & Francis SSH And S&T Upgrade 2017
KB+ BIBSAM Taylor Francis Journals 2015-2017
KB+ BIBSAM Taylor Francis Medical Package Upgrade 2017
KB+ JISC Collections Bloomsbury Taylor & Francis Full Collection 2015
KB+ JISC Collections Maney Full Digital Archive 2015-2017
KB+ JISC Collections Maney Health Sciences Digital Archive 2015-2017
KB+ JISC Collections Maney Materials Science And Engineering Digital Archive 2015-2017
KB+ JISC Collections Taylor & Francis Science and Technology Expanded 2016-2017
KB+ JISC Collections Taylor & Francis Social Science and Humanities Expanded 2016-2017
KB+ JISC Collections Taylor And Francis Combined Social Science And Humanities And Science And Technology 2018-2020
KB+ JISC Collections Taylor And Francis Medical Library 2018-2020
KB+ JISC Collections Taylor And Francis Science And Technology 2018-2020

KB+ JISC Collections Taylor And Francis Social Science And Humanities 2018-2020
KB+ JISC Collections Taylor Francis Full Collection 2015-2017
KB+ JISC Collections Taylor Francis Science And Technology Collection 2015-2017
KB+ JISC Collections Taylor Francis Social Science And Humanities Collection 2015-2017
KB+ JISC Collections: Taylor & Francis: Geography, Planning, Urban and Environment Online Archive
KB+ Taylor & Francis:Jisc Collections:Full Collection:Expanded:2016-2017
Royal Society of New Zealand free
Taylor & Francis Allied & Public Health Archive 2017
Taylor & Francis Anthropology & Archaeology Online Archive
Taylor & Francis Archaeology, Anthropology and Heritage
Taylor & Francis Arts & Humanities Online Archive
Taylor & Francis Arts & Humanities Online Collection
Taylor & Francis Behavioral Collection
Taylor & Francis Behavioral Online Archive
Taylor & Francis Biological, Earth & Environment Science Archive 2017
Taylor & Francis Biological, Earth, Environmental & Food Sciences
Taylor & Francis Business & Management Collection
Taylor & Francis Business, Management and Economics Archive 2009
Taylor & Francis Business, Management and Economics Online Archive
Taylor & Francis Chemistry Collection
Taylor & Francis Chemistry Online Archive
Taylor & Francis Clinical Psychiatry & Neuroscience Archive 2017
Taylor & Francis Criminology and Law Collection
Taylor & Francis Criminology and Law Online Archive
Taylor & Francis CRKN Medical
Taylor & Francis CRKN Science and Technology
Taylor & Francis CRKN Social Science and Humanities
Taylor & Francis Current Content Access
Taylor & Francis eBooks Open Access
Taylor & Francis Education Collection
Taylor & Francis Education Online Archive
Taylor & Francis Engineering, Computing & Technology Collection
Taylor & Francis Engineering, Computing & Technology Online Archive
Taylor & Francis Environment & Agriculture Archive
Taylor & Francis Expert Opinion Modern Archive
Taylor & Francis Expert Review Modern Archive
Taylor & Francis FRESH Combined Package
Taylor & Francis FRESH Medical
Taylor & Francis FRESH Science & Technology
Taylor & Francis FRESH Social Science & Humanities

Taylor & Francis Geography, Planning, Urban and Environment Collection
Taylor & Francis Geography, Planning, Urban and Environment Online Archive
Taylor & Francis Group Mathematics & Statistics Collection
Taylor & Francis Group Mathematics & Statistics Online Archive
Taylor & Francis Journals Complete
Taylor & Francis Library & Information Science Collection
Taylor & Francis Library & Information Science Collection Archive
Taylor & Francis Media, Cultural & Communication Studies Online Archive
Taylor & Francis Media, Culture & Communication Studies Collection
Taylor & Francis Medical Classic Archive
Taylor & Francis Medical Library
Taylor & Francis Medical Modern Archive
Taylor & Francis Medical New Launch Trial (Volume 1 & 2)
Taylor & Francis Medicine and Dentistry Archive 2017
Taylor & Francis Online 2020 - CAUL
Taylor & Francis Online ADF Bespoke Collection 2020
Taylor & Francis Online ANZ Medical Bespoke Collection 2020
Taylor & Francis Online Ceased Titles 2020 - CAUL
Taylor & Francis Online DHB Medical Bespoke Collection 2020
Taylor & Francis Online Open Access 2020 - CAUL
Taylor & Francis Online Perpetual Access 2019 - CAUL
Taylor & Francis Online RMH Bespoke Collection 2020
Taylor & Francis Online VHLC Medical Bespoke Collection 2020
Taylor & Francis Online William Angliss Bespoke Collection 2020
Taylor & Francis Open Access
Taylor & Francis Pharmaceutical Science & Toxicology Archive 2017
Taylor & Francis Physics Collection
Taylor & Francis Physics Online Archive
Taylor & Francis Politics International Relations & Area Studies Collection
Taylor & Francis Politics, International Relations & Area Studies Online Archive
Taylor & Francis Psychology Archive 2017
Taylor & Francis Public Health & Social Care Online Archive
Taylor & Francis Science & Technology New Launch Trial (Volume 1 & 2)
Taylor & Francis Science and Technology Library
Taylor & Francis Social Science & Humanities New Launch Trial (Volume 1 & 2)
Taylor & Francis Social Science & Humanities with Science & Technology
Taylor & Francis Social Science and Humanities Library
Taylor & Francis Sociology & Related Disciplines Collection
Taylor & Francis Sociology & Related Disciplines Online Archive
Taylor & Francis Sport, Leisure & Tourism Collection

Taylor & Francis Sport, Leisure & Tourism Online Archive
Taylor & Francis Strategic Defence & Security Studies Archive
Taylor & Francis Strategic, Defence & Securities Studies Collection
TDX - Theses and Dissertations Online
Tech Science Press
TI Global - Agroalimentaire
TI Global - All Titles
TI Global - Automatique et Ingénierie Système
TI Global - Bioprocédés
TI Global - Bois Verre Céramique et Textile
TI Global - Bruit et vibrations
TI Global - Chimie Verte
TI Global - Conception et Production
TI Global - Constantes physico-chimiques
TI Global - Conversion de l’énergie électrique
TI Global - Corrosion et vieillissement
TI Global - Documents Numériques Gestion de Contenu
TI Global - Droit et Organisation Générale de la Construction
TI Global - Éco-conception et Innovation Responsable
TI Global - Élaboration et Recyclage des Métaux
TI Global - Électronique
TI Global - Emballages
TI Global - Environnement
TI Global - Étude et Propriétés des Métaux
TI Global - Fonctions et composants mécaniques
TI Global - Formulation
TI Global - Froid industriel
TI Global - Frottement, Usure et Lubrification
TI Global - Génie nucléaire
TI Global - Innovations technologiques
TI Global - Instrumentation et méthodes de mesure
TI Global - La Construction Responsable
TI Global - Le second oeuvre et l'équipement du bâtiment
TI Global - Le traitement du signal et ses applications
TI Global - Les superstructures du bâtiment
TI Global - Logistique
TI Global - Machines hydrauliques, aérodynamiques et thermiques
TI Global - Maintenance
TI Global - Management et ingénierie de l'innovation
TI Global - Management Industriel

TI Global - Matériaux fonctionnels
TI Global - Mathématiques
TI Global - Mécanique des sols et géotechnique
TI Global - Médicaments et produits pharmaceutiques
TI Global - Mesures et tests électroniques
TI Global - Mesures mécaniques et dimensionnelles
TI Global - Mesures physiques
TI Global - Métier : Ingénieur territorial
TI Global - Métier : Responsable bureau d’étude-conception
TI Global - Métier : Responsable environnement
TI Global - Métier : Responsable qualité
TI Global - Métier : Responsable risque chimique
TI Global - Mise en forme des métaux et fonderie
TI Global - Nanosciences et nanotechnologies
TI Global - Opérations unitaires. Génie de la réaction chimique
TI Global - Optique Photonique
TI Global - Physique Chimie
TI Global - Physique énergétique
TI Global - Plastiques et composites
TI Global - Qualité et sécurité au laboratoire
TI Global - Réseaux électriques et applications
TI Global - Réseaux Télécommunications
TI Global - Ressources énergétiques et stockage
TI Global - Robotique
TI Global - Sécurité des systèmes d'information
TI Global - Sécurité et gestion des risques
TI Global - Systèmes aéronautiques
TI Global - Systèmes ferroviaires
TI Global - Techniques d'analyse
TI Global - Techniques du bâtiment : le second oeuvre et les lots techniques
TI Global - Techniques du bâtiment : l'enveloppe du bâtiment
TI Global - Techniques du bâtiment : préparer la construction
TI Global - Technologies biomédicales
TI Global - Technologies de l'eau
TI Global - Technologies logicielles Architectures des systèmes
TI Global - Technologies radars et applications
TI Global - Thermique industrielle
TI Global - Traçabilité
TI Global - Traitements des métaux
TI Global - Transport fluvial et maritime

TI Global - Travail des matériaux - Assemblage
TI Global - Travaux publics et infrastructures
TI Global - Véhicule et mobilité du futur
TI Global - Vieillissement, pathologies et réhabilitation du bâtiment
TI Global - Contro´le non destructif
Techno Press
TUMS Electronic Journals
Temple University ContentDM
International journal of engineering education
Termedia Journals
Terrapub Journals
Test Prep Review
Stat!Ref (National Library Alliance)
Digital Commons @ The Texas Medical Center
ThinkTech
Textiles Intelligence
The Chemical Society of Japan Free
The Chemical Society of Japan Subscribed
Freedonia Focus Industry Market Research Reports
The Historical Association
PolyU Institutional Research Archive
IET Digital Library - eBooks 2014
The Journal of Bone and Joint Surgery
NCPEA Publications
Bacon Thieme Publishing Group Couperin Chimie
Bacon Thieme Publishing Group Couperin Medical
KB+ BIBSAM Thieme eBook Library 2014-2016
KB+ JISC Collections Georg Thieme Verlag Chemistry Collection 2015
KB+ JISC Collections Georg Thieme Verlag Chemistry Collection 2017
KB+ JISC Collections Georg Thieme Verlag Medicine Collection 2015
KB+ JISC Collections Georg Thieme Verlag Medicine Collection 2017
KB+ Jisc Collections Georg Thieme Verlag Medicine Collection 2018
KB+ Jisc Collections Georg Thieme Verlag Transitional Open Access Chemistry Collection 2020-2021
Thieme Clinical Collections
Thieme Clinical Collections 2015
Thieme Connect
Thieme Connect Free
Thieme E-Book Library Klinik und Praxis
Thieme E-Book Library Studium und Lehre
Thieme E-Book Library Veterinärmedizin

Thieme EbookLibrary
Thieme E-Library
Thieme eRef
Thieme MedOne Neurosurgery
Thieme MedOne Radiology
Thieme MedOne Spine
Thieme-Connect
Thieme-Connect Backfiles
Thomas Land Publishers Inc.
Arts & Humanities Citation Index
MEDLINE
Science Citation Index Expanded
Social Sciences Citation Index
Web of Science Complete
Web of Science - Arts & Humanities Citation Index - 1989
Web of Science - Arts & Humanities Citation Index - 1990
Web of Science - Arts & Humanities Citation Index - 1991
Web of Science - Arts & Humanities Citation Index - 1992
Web of Science - Arts & Humanities Citation Index - 1993
Web of Science - Arts & Humanities Citation Index - 1994
Web of Science - Arts & Humanities Citation Index - 1995
Web of Science - Arts & Humanities Citation Index - 1996
Web of Science - Arts & Humanities Citation Index - 1997
Web of Science - Arts & Humanities Citation Index - 1998
Web of Science - Arts & Humanities Citation Index - 1999
Web of Science - Arts & Humanities Citation Index - 2000
Web of Science - Arts & Humanities Citation Index - 2001
Web of Science - Arts & Humanities Citation Index - 2002
Web of Science - Arts & Humanities Citation Index - 2003
Web of Science - Arts & Humanities Citation Index - 2004
Web of Science - Arts & Humanities Citation Index - 2005
Web of Science - Arts & Humanities Citation Index - 2006
Web of Science - Arts & Humanities Citation Index - 2007
Web of Science - Arts & Humanities Citation Index - 2008
Web of Science - Arts & Humanities Citation Index - 2009
Web of Science - Arts & Humanities Citation Index - 2010
Web of Science - Arts & Humanities Citation Index - 2011
Web of Science - Arts & Humanities Citation Index - 2012
Web of Science - Arts & Humanities Citation Index - 2013
Web of Science - Arts & Humanities Citation Index - 2014

Web of Science - Arts & Humanities Citation Index - 2015
Web of Science - Arts & Humanities Citation Index - 2016
Web of Science - Arts & Humanities Citation Index - 2017
Web of Science - Arts & Humanities Citation Index - 2018
Web of Science - Arts & Humanities Citation Index - 2019
Web of Science - Arts & Humanities Citation Index - 2020
Web of Science - Arts & Humanities Citation Index - 2021
Web of Science - Science Citation Index Expanded - 1989
Web of Science - Science Citation Index Expanded - 1990
Web of Science - Science Citation Index Expanded - 1991
Web of Science - Science Citation Index Expanded - 1992
Web of Science - Science Citation Index Expanded - 1993
Web of Science - Science Citation Index Expanded - 1994
Web of Science - Science Citation Index Expanded - 1995
Web of Science - Science Citation Index Expanded - 1996
Web of Science - Science Citation Index Expanded - 1997
Web of Science - Science Citation Index Expanded - 1998
Web of Science - Science Citation Index Expanded - 1999
Web of Science - Science Citation Index Expanded - 2000
Web of Science - Science Citation Index Expanded - 2001
Web of Science - Science Citation Index Expanded - 2002
Web of Science - Science Citation Index Expanded - 2003
Web of Science - Science Citation Index Expanded - 2004
Web of Science - Science Citation Index Expanded - 2005
Web of Science - Science Citation Index Expanded - 2006
Web of Science - Science Citation Index Expanded - 2007
Web of Science - Science Citation Index Expanded - 2008
Web of Science - Science Citation Index Expanded - 2009
Web of Science - Science Citation Index Expanded - 2010
Web of Science - Science Citation Index Expanded - 2011
Web of Science - Science Citation Index Expanded - 2012
Web of Science - Science Citation Index Expanded - 2013
Web of Science - Science Citation Index Expanded - 2014
Web of Science - Science Citation Index Expanded - 2015
Web of Science - Science Citation Index Expanded - 2016
Web of Science - Science Citation Index Expanded - 2017
Web of Science - Science Citation Index Expanded - 2018
Web of Science - Science Citation Index Expanded - 2019
Web of Science - Science Citation Index Expanded - 2020
Web of Science - Science Citation Index Expanded - 2021

Web of Science - Social Sciences Citation Index - 1989
Web of Science - Social Sciences Citation Index - 1990
Web of Science - Social Sciences Citation Index - 1991
Web of Science - Social Sciences Citation Index - 1992
Web of Science - Social Sciences Citation Index - 1993
Web of Science - Social Sciences Citation Index - 1994
Web of Science - Social Sciences Citation Index - 1995
Web of Science - Social Sciences Citation Index - 1996
Web of Science - Social Sciences Citation Index - 1997
Web of Science - Social Sciences Citation Index - 1998
Web of Science - Social Sciences Citation Index - 1999
Web of Science - Social Sciences Citation Index - 2000
Web of Science - Social Sciences Citation Index - 2001
Web of Science - Social Sciences Citation Index - 2002
Web of Science - Social Sciences Citation Index - 2003
Web of Science - Social Sciences Citation Index - 2004
Web of Science - Social Sciences Citation Index - 2005
Web of Science - Social Sciences Citation Index - 2006
Web of Science - Social Sciences Citation Index - 2007
Web of Science - Social Sciences Citation Index - 2008
Web of Science - Social Sciences Citation Index - 2009
Web of Science - Social Sciences Citation Index - 2010
Web of Science - Social Sciences Citation Index - 2011
Web of Science - Social Sciences Citation Index - 2012
Web of Science - Social Sciences Citation Index - 2013
Web of Science - Social Sciences Citation Index - 2014
Web of Science - Social Sciences Citation Index - 2015
Web of Science - Social Sciences Citation Index - 2016
Web of Science - Social Sciences Citation Index - 2017
Web of Science - Social Sciences Citation Index – 2018
Web of Science - Social Sciences Citation Index – 2019
Web of Science - Social Sciences Citation Index – 2020
Web of Science - Social Sciences Citation Index – 2021
Westlaw UK Journals (Full Text & Abstracts)
Westlaw UK Journals (Full Text)
Journals@UrMEL
Tibetan and Himalayan Digital Library Journals
Tidsskrift.dk Open Access Free
Tímarit
Tire Society Publications

TKC Law Library Journals
TOHO University Books
Hitotsubashi Business Review (一橋ビジネスレビュー)
Kinyu Business (The Financial Business Review) (金融ビジネス)
Think!
Toyo Keizai Tokei Geppo (Statistics Monthly) (統計月報)
Weekly Toyo Keizai (週刊東洋経済)
Transportation Research Record
Times Higher Education
Turcademy
Catalog of U.S. Government Publications (CGP)
FDsys - Government Publications
Federal Depository Library Program
Ubiquity Partner Network - Journals
Ubiquity Press Books (Open Access)
UCL Discovery
UCLA Chicano Studies Research Center
UCLA American Indian Studies Center
UCLA Asian American Studies Center Press
UDN eBooks 電子書
InCT Kennisbank
ULAKBIM - Mühendislik ve Temel Bilimler Veri Tabani
ULAKBIM - Turk Sosyal Bilimler Veri Tabani
ULAKBIM - Türk Tıp Veri Tabani
ULAKBIM - Yaşam Bilimleri Veri Tabani
Relief Central
uCentral
unipub Repository
UniSA Library Open Journal Systems Free
UniSA Research Outputs Repository
Unitec Research Bank
Sinica Sinoweb (經典人文學刊庫)
Office of Fair Trading Publications
United Kingdom Serials Group
UN iLibrary
United Nations Juridical Yearbook
United States Pharmacopeia and the National Formulary USP-NF
REDALyC
Universidad Complutense Madrid
UCrea

Universidade do Porto Institutional Repository All Content
Universidade do Porto Institutional Repository Open Access
Universita degli Studi di Milano Bicocca
Archives Italiennes de Biologie - a Journal of Neuroscience
ELEA
UAB Digital Repository
Diposit Digital de la Universitat de Barcelona
DUGiDocs (Universitat de Girona)
DUGiFonsEspecials (Universitat de Girona)
DUGiMedia (Universitat de Girona)
Universität Konstanz Institional Repository (KOPS)
Universitat Oberta de Catalunya E-Journals
RiuNet (Universitat Politècnica de València)
Repositori Digital de la UPF (Universitat Pompeu Fabra)
Digital Dissertations (Universitätsbibliothek der LMU Muenchen)
Open Access LMU
Bibliographie der deutschen Sprach- und Literaturwissenschaft (BDSL)
Heidelberger Historische Bestände - Zeitschriften und Zeitungen
Elektronische Zeitschriftenbibliothek - Frei zugängliche E-Journals
Université de Liège - Open Repository and Bibliography (ORBI)
Constellation (Université du Québec à Chicoutimi)
Digital Library and Archives Electronic Journals
Aberdeen University Research Archive
University of Adelaide Press (Open Access)
University of Arizona Institutional Repository
Opus: University of Bath's Eprints IR
Bergen Open Research Archive (BORA)
UBIRA ePapers (University of Birmingham)
UBIRA eTheses (University of Birmingham)
University of Boras DSpace
cIRcle: UBC's dSpace IR
Digital Collections: UBC's ContentDM IR
University of Calgary Press - Open access eBooks
University of Calgary Press Journals
Mathematical Sciences Publishers
Hispanic American Periodicals Index (HAPI Online)
KB+ JISC Collections University Of California Press Complete Collection 2018
KB+ JISC Collections University Of California Press Complete Collection 2019
KB+ JISC Collections University Of California Press Complete Plus Collection 2018
KB+ JISC Collections University Of California Press Complete Plus Collection 2019

KB+ JISC Collections University Of California Press Premium Collection 2018
KB+ JISC Collections University Of California Press Premium Plus Collection 2018
Luminos
Thesaurus Linguae Graecae
University of California Press Journals
Archive of Popular American Music
Chicago Manual of Style Online
University of Chicago Press Journals
University of Chicago Press Journals (Current Content)
ROAR (University of East London Repository)
Databáze VŠKP
University of Edinburgh dspace
Chemical Engineering Documents
University of Guelph Theses and Dissertations
Houston journal of mathematics
University of Huddersfield LibGuides
University of Huddersfield Library Catalogue
University of Huddersfield Repository
University of Illinois Press
Iowa Research Online
Kent Academic Repository
UKnowledge
UL PDA
University of Limerick dspace
British History Online
Digital Commons @ UMaine
Cross Currents
Deep Blue Data at the University of Michigan
Digital Library Production Service (DLPS)
digitalculturebooks
Making of America Books
Making of America Journals (Michigan)
Open Humanities Press
Abraham Lincoln Association Serials
UMPEBC UMPEBC University of Michigan Press eBooks 2014
UMPEBC University of Michigan Press eBooks
UMPEBC University of Michigan Press eBooks 2011 & Prior
UMPEBC University of Michigan Press eBooks 2012
UMPEBC University of Michigan Press eBooks 2013
UMPEBC University of Michigan Press eBooks 2015

UMPEBC University of Michigan Press eBooks 2016
UMPEBC University of Michigan Press eBooks 2017
UMPEBC University of Michigan Press eBooks 2018
UMPEBC University of Michigan Press eBooks 2019
UMPEBC University of Michigan Press eBooks Open Access
Open Textbook Catalog
University of Minnesota Digital Conservancy
Estuaries Research Federation
Maureen and Mike Mansfield Library CONTENTdm Respository
Digital Commons @ University of Nebraska - Lincoln
Internet Center for Wildlife Damage Management
Journal of Latino and Latin American Studies
UNLV Digital Collections
Electronic Text Centre - University of New Brunswick
Latin America Data Base
UNSWorks (University of New South Wales)
Portal to Texas History
University of North Texas Digital Library
SHAREOK Repository
Otago University Research Archive
School of Business ePrints
Te Tumu ePrints
University of Pennsylvania Press Journals
Archive of European Integration (AEI)
D-Scholarship@Pitt: Institutional Repository of the University of Pittsburgh
D-Scribe Digital Publishing
Études Ricoeuriennes / Ricoeur Studies
Industry Studies Working Papers
International Journal of Telerehabilitation
Revista Iberoamericana
University Library System, University of Pittsburgh: D-Scribe Collections
University of Pittsburgh Aphasiology Archive
University of Pittsburgh Digital Research Library
University of Pittsburgh Minority Health and Health Equity Archive
University of Pittsburgh Philosophy of Science
UQ Library - Digitised Materials - UQ staff and students only
University Of Queensland Press
University of Richmond UR Scholarship Repository
University of Rochester Digital Collections
University of Rochester LibGuides

University of Rochester Library Catalog
ARROW
Scholar Commons
ONE Archives at the University of Southern California Book Collection
Shoah Foundation Institute Visual History Archive
University of Southern California Health Sciences Catalog
University of Southern California: Norris LibGuides
University of Southern California: Wilson LibGuides
USC Databases
USC Digital Library
USC Libraries LibGuides
USC Libraries Special Collections
University of Southern Denmark PURE
Surrey Research Insight
Sydney eScholarship Repository
University of Texas at Arlington Dspace
University of Texas at Arlington LibGuides
Archive of Indigenous Languages of Latin America
American Literary Translators Association Publications
UTPB Library Catalog
University of Texas Press Journals
University of the Arts London Research Online
International Journal of Developmental Biology
University of Toronto T-Space
University of Toronto Press
ScholarWorks @ UVM
University of Victoria Digital Collections
UVic Abacus Dataverse Network
UWIC DSpace
Cardiff University, New Readings
University of Warwick Electronic Law Journals
University of Waterloo UWSpace
University of Wisconsin Digital Collections Center
Wolverhampton Intellectual Repository and E-theses (WIRE)
Internet archaeology
ZORA
ZORA (Open Access)
Digitální knihovna Univerzity Pardubice (dspace)
Die digitale Landesbibliothek Oberösterreich (Upper Austrian Federal State Library)
Alvin, Uppsala University Library

Uppsala University DiVA
USAE Engineer Research and Development Center Reports
USPTO Issued Patents
USPTO Published Applications
UTB studi-e-book
Utrecht University Repository
Van Duuren Ebooks
VIUSpace
Vandenhoeck & Ruprecht Journals
Vandenhoeck & Ruprecht: Kritisch-exegetischer Kommentar über das Neue Testament
Vandenhoeck And Ruprecht Complete
Vandenhoeck And Ruprecht Formen Der Erinnerung
Vandenhoeck And Ruprecht Schriften Des Hannah-Arendt-Instituts
V&R Unipress Journals
Vanderbilt University Institutional Repository
Vathek Publishing
VCU Scholars Compass
Budrich Journals
Verlag Österreich eLibrary
Vetus Testamentum Supplements Online, Supplement 2014
Victoria and Albert Museum Research & Conservation Publications
VLeBooks
Vrije Universiteit
Wageningen Academic Publishers eBooks
Wageningen Academic Publishers Journals
Wageningen Academic Publishers Open Access
Wake Forest Law Publications
Walden University Journals
COVID-19 Free Access eBooks
De Gruyter bepress ResearchNow - CAUL
De Gruyter Complete Journal Package 2020
De Gruyter Complete Journal Package 2020 in English
De Gruyter Complete Journal Package HSS 2020
De Gruyter Complete Journal Package HSS 2020 in English
De Gruyter Complete Journal Package STM 2020
De Gruyter Complete Journal Package STM 2020 in English
De Gruyter EBA Package: All Backlist eBooks
De Gruyter eBooks Architecture and Design 2016
De Gruyter eBooks Art 2016
De Gruyter eBooks Classical and Ancient Near Eastern Studies 2016

de Gruyter eBooks Complete
De Gruyter eBooks Economics 2016
De Gruyter eBooks Engineering, Computer Sciences 2016
De Gruyter eBooks History 2016
De Gruyter eBooks Law 2016
De Gruyter eBooks Library and Information Science 2016
De Gruyter eBooks Linguistics 2016
De Gruyter eBooks Literary Cultural and Area Studies 2016
De Gruyter eBooks Mathematics 2016
De Gruyter eBooks Medicine and Life Sciences 2016
De Gruyter eBooks Philosophy 2016
De Gruyter eBooks Physics, Chemistry, Industrial Chemistry, Materials Sciences, Geosciences 2016
De Gruyter eBooks Social Sciences 2016
De Gruyter eBooks Theology and Religious Studies, Jewish Studies 2016
De Gruyter e-Journal HSS Collection 2020 KERIS
De Gruyter Journal Package Biology, Chemistry, Geosciences 2020
De Gruyter Journal Package Classical Studies, History 2020
De Gruyter Journal Package Law 2020
De Gruyter Journal Package Library and Information Science, Library Reference 2020
De Gruyter Journal Package Linguistics, Literature 2020
De Gruyter Journal Package Mathematics, Physics, Engineering 2020
De Gruyter Journal Package Medicine 2020
De Gruyter Journal Package Philosophy, Theology, Judaism, Religion 2020
De Gruyter Journal Package Politics, Economics, Sociology 2020
De Gruyter Journals Biology Chemistry Geosciences 2018
De Gruyter Journals Classical Studies History 2018
De Gruyter Journals HSS All Languages 2018
De Gruyter Journals HSS All Languages 2019
De Gruyter Journals Law 2018
De Gruyter Journals Library And Information Science Library Reference 2018
De Gruyter Journals Linguistics Literature 2018
De Gruyter Journals Mathematics Physics Engineering 2018
De Gruyter Journals Medicine 2018
De Gruyter Journals Philosophy Theology Judaism Religion 2018
De Gruyter Journals Politics Economics Sociology 2018
De Gruyter Journals STM All Languages 2018
De Gruyter Journals STM All Languages 2019
De Gruyter Journals STM English 2018
De Gruyter Online Journals
De Gruyter PDA Package: All Databases and eBooks

De Gruyter PDA Package: All eBook Content, incl. OWV/AV
De Gruyter PDA Package: All eBooks and english HSS
De Gruyter PDA Package: All eBooks english STM
De Gruyter PDA Package: All English Language eBooks
De Gruyter PDA Package: All Humanities eBooks and Databases
De Gruyter PDA Package: All Humanities eContent, incl.OWV/AV
De Gruyter PDA Package: All SSH eBooks
De Gruyter PDA Package: All STM eBook
De Gruyter PDA Package: All STM eBooks and Databases
De Gruyter PDA Package: All STM eContent, incl. OWV/AV
De Gruyter PDA Package: Columbia Pilot Project 2016
De Gruyter PDA Package: Harvard Pilot Project 2016
De Gruyter PDA Package: Princeton Pilot Project 2016
De Gruyter PDA Package:Complete Content Collection, incl. OWV/AV
KB+ Bibsam De Gruyter Complete Collection 2018-2020
KB+ BIBSAM De Gruyter Journals 2015
KB+ JISC Collections De Gruyter Complete Collection 2016-2018
KB+ JISC Collections De Gruyter Complete Package English Titles 1826-2014
KB+ JISC Collections De Gruyter Journal Archive 1826-2012
KB+ JISC Collections De Gruyter Library And Information Science 1826-2014
KB+ JISC Collections De Gruyter Linguistics Literature Collection 1826-2014
KB+ JISC Collections De Gruyter Mathematics Physics Engineering 1826-2014
KB+ JISC Collections De Gruyter Medicine Collection 1826-2014
KB+ JISC Collections De Gruyter Philosophy Theology Judaism Religion 1826-2014
KB+ JISC Collections De Gruyter Science Technical And Medical 1826-2014
KB+ JISC Collections De Gruyter SSH English Titles 1826-2014
Oldenbourg Verlagsgruppe Zeitschriften
Walter de Gruyter Reference Global eBooks Science, Technology and Medicine
Walter De Gruyter: Open Access eBooks
Walter De Gruyter: Open Access Journals
Academic Conferences in China (Arts/Humanities/Social Sciences) (Mainland China Only)
China Local Gazetteers
China Online Journals (COJ)
Dissertations of China (Mainland China Only)
WANFANG Data Centre
学位论文 (Dissertations of China) (Outside Mainland China Only)
WARC.com
Leyburn Electronic Government Documents
Washington and Lee University Scholarly Commons
Washington Post

Wellesley College Digital Repository
Thomson Reuters Westlaw Campus Research
Westlaw Asia
Westlaw China
WestlawNext Campus Research Law
WestlawNext Campus Research Law + News
WestlawNext Campus Research Law + News + Business
White Rose Research Online
Whiting & Birch
Wichtig Editore
Wikipedia
Wilderness Medical Society Journals
Wildlife Online
Cochrane Library
Cochrane Library Open Aceess
COVID-19: effective options for quitting smoking during the pandemic - Open Access
COVID-19: evidence relevant to critical care - Open Access
COVID-19: infection control and prevention measures - Open Access
COVID-19: regional anaesthesia to reduce drug use in anaesthesia and avoid aerosol generation
CRKN Wiley Blackwell Backfiles
CRKN Wiley Online Library
KB+ BIBSAM Wiley Database Model 2016-2018
KB+ BIBSAM Wiley Online Library Custom Collection 2014-2015
KB+ JISC Collections Wiley Online Library Full Collection 2018
KB+ Jisc Collections Wiley Online Library Full Collection 2019
KB+ JISC Collections Wiley Online Library Full Collection:2015-2017
KB+ JISC Collections Wiley Online Library Medicine and Nursing 2015-2017
KB+ JISC Collections Wiley Online Library Medicine and Nursing Collection 2018
KB+ JISC Collections Wiley Online Library Smaller Collection 2018
KB+ JISC Collections Wiley Online Library Smaller Collection:2015-2017
KB+ JISC Collections Wiley Online Library SSH Collection 2015-2017
KB+ JISC Collections Wiley Online Library SSH Collection 2018
KB+ JISC Collections Wiley Online Library STM Collection 2015-2017
KB+ JISC Collections Wiley Online Library STM Collection 2018
KB+ SHEDL Wiley Full Collection 2015-2017
KB+ SHEDL Wiley Online Library Full Collection 2018
KB+ WHEEL Wiley Online Library Full Collection 2015-2017
NESLI2 Wiley Online Library Full Collection:2012-2014
NESLi2 Wiley Online Library SSH Collection 2012-2014
NESLi2 Wiley Online Library STM Collection 2012-2014

VIVA Wiley Perpetual Access Journals
Wiley Frontlist All Obook 2014 to Present
Wiley Frontlist All Obook 2015
Wiley Frontlist All Obook 2016
Wiley Frontlist All Obook 2018
Wiley Frontlist Obook Business 2015-2016
Wiley Frontlist Obook Business 2016
Wiley Frontlist Obook Chemistry 2015-2016
Wiley Frontlist Obook Chemistry 2016
Wiley Frontlist Obook Engineering 2015-2016
Wiley Frontlist Obook Engineering 2016
Wiley Frontlist Obook Health Science 2015-2016
Wiley Frontlist Obook Health Science 2016
Wiley Frontlist Obook Humanities 2015-2016
Wiley Frontlist Obook Humanities 2016
Wiley Frontlist Obook Life Science 2015-2016
Wiley Frontlist Obook Life Science 2016
Wiley Frontlist Obook Math & Statistics 2015-2016
Wiley Frontlist Obook Math & Statistics 2016
Wiley Frontlist Obook Physical Science Engineering 2018
Wiley Frontlist Obook Social Sciences 2015-2016
Wiley Frontlist Obook Social Sciences 2016
Wiley Frontlist Obook Veterinary Science 2015-2016
Wiley Frontlist Obook Veterinary Science 2016
Wiley Not in Any Collection 2018
Wiley Online Book 2010 to Present
Wiley Online Book 2011 to Present
Wiley Online Library (EIRA)
Wiley Online Library 2010 Full Collection
Wiley Online Library 2011 Full Collection
Wiley Online Library 2012 Full Collection
Wiley Online Library 2013 Full Collection
Wiley Online Library 2014 Full Collection
Wiley Online Library AGU 2016
Wiley Online Library AGU 2017
Wiley Online Library AGU Backfiles
Wiley Online Library AGU Free Content
Wiley Online Library AGU Journal Collection
Wiley Online Library All Frontlist Titles 2019
Wiley Online Library All Journals

Wiley Online Library Analytical Science Backfiles
Wiley Online Library Anesthesia Intensive Care Backfiles
Wiley Online Library Anthrosource
Wiley Online Library Aquaculture and Fish Sciences Backfiles
Wiley Online Library Backfiles Complete
Wiley Online Library Backlist Obook AGU 2017
Wiley Online Library Backlist Obook Business 2017
Wiley Online Library Backlist Obook Chemistry 2017
Wiley Online Library Backlist Obook Engineering 2017
Wiley Online Library Backlist Obook German Language 2017
Wiley Online Library Backlist Obook Health Sciences 2017
Wiley Online Library Backlist Obook Humanities 2017
Wiley Online Library Backlist Obook Life Sciences 2017
Wiley Online Library Backlist Obook Math & Stat 2017
Wiley Online Library Backlist Obook Social Sciences 2017
Wiley Online Library Backlist Obook Veterinary 2017
Wiley Online Library Biology backfiles
Wiley Online Library Biotechnology, Biochemistry, and Biophysics Backfiles
Wiley Online Library Business and Management Backfiles
Wiley Online Library Cardiology Backfiles
Wiley Online Library Cell and Developmental Biology Backfiles
Wiley Online Library Chemistry Backfiles
Wiley Online Library Chemistry Societies Backfiles
Wiley Online Library Chemistry Societies II Backfiles
Wiley Online Library Civil Engineering Backfiles
Wiley Online Library Communications Technology, Electrical and Systems Engineering Backfiles
Wiley Online Library Computer Science Backfiles
Wiley Online Library Current Protocols
Wiley Online Library Database Model 2016
Wiley Online Library Database Model 2017
Wiley Online Library Database Model 2018
Wiley Online Library Database Model 2019
Wiley Online Library Database Model 2020
Wiley Online Library Dentistry backfiles
Wiley Online Library Dermatology Backfiles
Wiley Online Library Economics, Finance and Accounting Backfiles
Wiley Online Library Education Backfiles
Wiley Online Library Food Science Backfiles
Wiley Online Library Free Content
Wiley Online Library Frontlist 2017

Wiley Online Library Full Collection 2017
Wiley Online Library Full Collection 2018
Wiley Online Library Full Collection 2019
Wiley Online Library Full Collection 2020
Wiley Online Library Gastroenterology and Hepatology Backfiles
Wiley Online Library General Medicine Backfiles
Wiley Online Library Genetics Backfiles
Wiley Online Library Geography Backfiles
Wiley Online Library History & Archaeology backfiles
Wiley Online Library Infectious Diseases Backfiles
Wiley Online Library Journal of Pathology Backfiles
Wiley Online Library Journals 2016 - CAUL
Wiley Online Library Journals Frontfile Complete
Wiley Online Library Language and Linguistics Backfiles
Wiley Online Library Law backfiles
Wiley Online Library Literature, Cultural Studies and Art Backfiles
Wiley Online Library Material Science Backfiles
Wiley Online Library Mathematics Backfiles
Wiley Online Library Medicine and Nursing 2011 Collection
Wiley Online Library Medicine and Nursing Collection 2012
Wiley Online Library Medicine and Nursing Collection 2013
Wiley Online Library Medicine and Nursing Collection 2018
Wiley Online Library Medicine and Nursing Collection 2020
Wiley Online Library Microbiology backfiles
Wiley Online Library Natural Sciences and Taxonomy Backfiles
Wiley Online Library Neurology, Neurosurgery and Pain Medicine Backfiles
Wiley Online Library Neuroscience Backfiles
Wiley Online Library Novartis Foundation Symposia
Wiley Online Library Numerical Engineering Backfiles
Wiley Online Library Nursing backfiles
Wiley Online Library Oncology Backfiles
Wiley Online Library Online Books
Wiley Online Library Open Access
Wiley Online Library Opt-In Titles
Wiley Online Library PALCI Backfiles Collection
Wiley Online Library Pharmaceutical and Medicinal Chemistry
Wiley Online Library Pharmacology and Toxicology Backfiles
Wiley Online Library Philosophy backfiles
Wiley Online Library Physical Science & Engineering Frontlist 2019
Wiley Online Library Physics

Wiley Online Library Physics and Astronomy Backfiles
Wiley Online Library Pilot 2013
Wiley Online Library Pilot 2014
Wiley Online Library Pilot 2015
Wiley Online Library Politics backfiles
Wiley Online Library Polymer Backfiles
Wiley Online Library Psychology Backfiles
Wiley Online Library Radiology Backfiles
Wiley Online Library Religion and Theology Backfiles
Wiley Online Library Science Technology and Medicine Collection 2017
Wiley Online Library Science Technology and Medicine Collection 2018
Wiley Online Library Science, Technology and Mathematics Collection 2020
Wiley Online Library Social Science and Humanities Collection 2016
Wiley Online Library Social Science and Humanities Collection 2017
Wiley Online Library Social Science and Humanities Collection 2018
Wiley Online Library Social Science and Humanities Collection 2020
Wiley Online Library Sociology Backfiles
Wiley Online Library SSH 2013
Wiley Online Library STM 2011
Wiley Online Library STM 2013
Wiley Online Library Surgery Backfiles
Wiley Online Library Transplantation Backfiles
Wiley Online Library Tribology Backfiles
Wiley Online Library UBCM Agriculture
Wiley Online Library UBCM all Online Books
Wiley Online Library UBCM Business
Wiley Online Library UBCM Chemistry 2000 to Present
Wiley Online Library UBCM Chemistry through 1999
Wiley Online Library UBCM Earth & Environmental
Wiley Online Library UBCM Engineering
Wiley Online Library UBCM German Language
Wiley Online Library UBCM Humanities
Wiley Online Library UBCM Life Science
Wiley Online Library UBCM Math & Statistics
Wiley Online Library UBCM Medicine and Nursing
Wiley Online Library UBCM Physical Science
Wiley Online Library UBCM Psychology
Wiley Online Library UBCM Social Behavioral
Wiley Online Library UBCM Veterinary Science
Wiley Online Library Urology Backfiles

Wiley Online Library Veterinary Backfiles
Wiley Online Major Reference Works
Wiley-Blackwell Encyclopedia of Literature
Wiley-Blackwell Online Books - BTAA Consortium 2010
Wiley Online Library Hematology Backfiles
Wiley-Blackwell Encyclopedia of Life Sciences
Wiso eBooks
Wiso Plus Standard
MedKnow Publications
Wolters Kluwer Open Health
Women's Wear Daily
Open Knowledge Repository
World Bank Data Catalog
World Development Indicators (WDI)
World Bank E-Library Journals
World Bank E-Library Policy Research Working Papers
World Bank E-Library Publications
World Book Advanced
World Book Online Info Finder
World Book Online Reference Center
World Book Student
WHO Digital Library
World Meteorological Organization Publications
World Scientific Archives
World Scientific eBooks
World Scientific E-Textbooks
World Scientific Journals
World Scientific Journals (Tsinghua Mirror)
World Scientific Open
Textile Magazines
CEABA Chemische Technik und Biotechnologie (CEAB)
ESTEC technology for environment and sustainability (ESTE)
TEMA - Technik und Management
Xavier University of Louisiana's Digital Archives
Yale University School of Medicine News & Publications
YIVO Encyclopedia of Jews in Eastern Europe
York Digital Journals
ECONIS
EconStor
Zentralblatt MATH

Chemistry of Plant Raw Material
AraBase
AraBase (Full Text Only- for Discovery)
Dissertations
Dissertations (Full Text Only- for Discovery)
EcoLink Arabic Database
EcoLink Arabic Database (Full Text Only- for Discovery)
EduSearch Arabic Database
EduSearch Arabic Database (Full Text Only- for Discovery)
HumanIndex
HumanIndex (Full Text Only- for Discovery)
IslamicInfo
IslamicInfo (Full Text Only- for Discovery)
中国学术会议论文全文数据库(China Academic Conference Proceedings Database, CACP)
中国学术期刊（网络版）
HUSCAP: Hokkaido University Collection of Scholarly and Academic Papers
国家哲学社会科学学术期刊数据库 (NSSD)
日経BP記事検索サービス　大学版
青空文庫

Table S2. MEDLINE and CHINAL.

Collection ID	Collection Name	DBID	Interface	Provider Coverage (metadata indexed directly from provider)
6,1324E+17	EBSCOhost MEDLINE Complete	SV3	EBSCOhost	No
6,11E+17	EBSCOhost MEDLINE with Full Text	EMB	EBSCOhost	No
6,1139E+15	MEDLINE	ECM	EBSCOhost	Yes
6,114E+15	Medline	CGR	Infotrieve, Inc	Yes
6,1139E+15	MEDLINE	CUY	OCLC	Yes
6,1139E+15	MEDLINE (Ovid)	CVF	Ovid	Yes
6,1496E+17	MEDLINE with Full Text	PSQYO	ProQuest	No
6,1139E+15	MEDLINE®	7X8	ProQuest	Yes
6,1139E+15	MEDLINE	EIF	Thomson Reuters	Yes
6,11E+17	EBSCOhost CINAHL	ECI	EBSCOhost	No
6,1345E+17	EBSCOhost CINAHL Complete	04C	EBSCOhost	No
6,11E+17	EBSCOhost CINAHL Plus	ECP	EBSCOhost	No
6,11E+17	EBSCOhost CINAHL Plus with Full Text	ECT	EBSCOhost	No
6,11E+17	EBSCOhost CINAHL with Full Text	ECF	EBSCOhost	No
6,1111E+16	Ovid CINAHL	AXR	Ovid	Yes
